# Author Correction: Switching imidazole reactivity by dynamic control of tautomer state in an allosteric foldamer

**DOI:** 10.1038/s41467-024-53247-9

**Published:** 2024-10-29

**Authors:** David P. Tilly, Jean-Paul Heeb, Simon J. Webb, Jonathan Clayden

**Affiliations:** 1 School of Chemistry, University of Bristol, Cantock’s Close, Bristol, BS8 1TS UK; 2 Department of Chemistry, University of Manchester, Oxford Road, Manchester, M13 9PL UK

Correction to: *Nature Communications* 10.1038/s41467-023-38339-2, published online 8 May 2023

In the Acknowledgements section of this article the grant number relating to Programme Grant Molecular Robotics given for Jonathan Clayden and Simon J. Webb was incorrectly given as EP/P027067 and should have been EP/P027067/1. Equally, the grant number relating to Bristol EPSRC Centre for Doctoral Training in Technology-Enhanced Chemical Synthesis for Jean-Paul Heeb was incorrectly given as EP/S024107 and should have been EP/S024107/1. The original article has been updated.

