# Supplementary material for: Switching imidazole reactivity by dynamic control of tautomer state in an allosteric foldamer
Source: Nat Commun. 2023 May 8;14:2647. doi: 10.1038/s41467-023-38339-2 (PMC10167260; doi:10.1038/s41467-023-38339-2)
Supplement: Supplementary file 1 — Supplementary Information [file 41467_2023_38339_MOESM1_ESM.pdf]

Supplementary Information

Switching imidazole reactivity by dynamic control of tautomer state in an allosteric foldamer

David P. Tilly[1,2]*, Jean-Paul Heeb[1], Simon J. Webb[2], Jonathan Clayden[1]*

[1] School of Chemistry, University of Bristol, Cantock's Close, Bristol BS8 1TS, UK

E-mail: j.clayden@bristol.ac.uk

[2] Department of Chemistry, University of Manchester, Manchester M13 9PL

E-mail: david.tilly@manchester.ac.uk

Contents

I. Supplementary Notes 5

1. General Information 5

II. Supplementary Methods 6

2. General synthetic schemes 6

2.1 Synthesis of compound **1** 6

2.2 Synthesis of compound **2** 6

2.3 Synthesis of compound **3** 7

3. Experimental procedures and characterisation 8

3.1. Synthesis of compound **1** 8

Compound **1b**. 8

Compound **1c** 8

Compound **1da** 9

Compound **1d** 9

Compounds **1ea** and **1eb** 10

Compound **1fb** 11

Compound **1fa** 12

Mixture of compounds **1fa** and **1fb** 12

Compound **1** 13

3.2. Synthesis of compound **2** 13

Compound **2b** 13

Compound **2c**. 14

Compounds **2da** and **2db** 15

Compound **2ea** 15

Compound **2**. 16

3.3. Synthesis of compound **3** 16

Compound **3e**. 17

Compound **3f**. 17

Compound **3g**. 18

Compound **3**. 18

3.4. Synthesis of control compounds **S15-S17** 19

Compound **SI5** 19

Compound **SI6** 19

Compound **SI7** 20

III. Supplementary Figures

4. NMR spectra of synthesised compounds 21

IV. Supplementary Discussion 61

5. NMR spectroscopy studies 61

5.1. Compound **1** 61

5.1.1. Assignment of ^{1}H and ^{13}C signals 61

5.1.2. Dilution study of compound **1** in CD_2Cl_2 at 25 °C 66

5.1.3. Variable temperature ^{1}H NMR study (25 to -40 °C) 68

5.1.4. Titration of compound **1** with tetrabutylammonium chloride in CD_2Cl_2 at 25 °C 70

5.1.5. NMR signal assignment of compound **1** mixed with 2 equivalents of tetrabutylammonium chloride in CD_2Cl_2 at 25 °C 74

5.1.6. Variable temperature NMR of compound **1** in the presence of 2 equivalents of tetrabutylammonium chloride 76

5.2. Compound **2** 78

5.2.1. Assignment of ^{1}H and ^{13}C NMR signals 78

5.2.2. DFT calculations on compound **2** 84

5.2.3. Titration of compound **2** with tetrabutylammonium chloride in CD_2Cl_2 at 25 °C 84

5.3. Compound **3** 86

5.3.1. Assignment of ^{1}H and ^{13}C NMR signals 86

5.3.2. Identification of hydrogen bond directionality preference 90

5.3.3. Titration of compound **3** with tetrabutylammonium chloride in CD_2Cl_2 at 25 °C 90

5.3.4. Titration of compound **3** with tetrabutylammonium iodide in CD_2Cl_2 at 25 °C 97

5.3.5. Titration of compound **3** with tetrabutylammonium bromide in CD_2Cl_2 at 25 °C 100

5.3.6. Titration of compound **3** with tetrabutylammonium nitrate in CD_2Cl_2 at 25 °C 103

5.4. Control NMR experiments on diurea **SI5** and tris(urea) **SI6** 106

5.4.1. Titration of diurea **SI5** with tetrabutylammonium chloride in CD_2Cl_2 at 25 °C 106

5.4.2. Titration of compound **SI6** with tetrabutylammonium chloride in CD_2Cl_2 at 25 °C 107

6. Deprotonation of 2,4-dinitrophenol 109

6.1. Titration of 2,4-dinitrophenol with imidazole in dry dichloromethane 109

6.1.1. UV-visible spectroscopy titration of 2,4-dinitrophenol with imidazole 109

6.1.2. Control experiments 110

6.2. Titration of 2,4-dinitrophenol with compound **1** in dry dichloromethane 111

6.2.1. UV-visible spectroscopy titration of 2,4-dinitrophenol with compound **1** 111

6.2.2. Control experiments 113

6.3. Titration of 2,4-dinitrophenol with compound **2** in dry dichloromethane 113

6.3.1. UV-visible spectroscopy titration of 2,4-dinitrophenol with compound **2** 113

6.3.2. Control experiment: UV absorbance of compound **2** 115
6.4. Titration of 2,4-dinitrophenol and compound **1** with tetrabutylammonium chloride 115
6.4.1. UV-visible spectroscopy titration 115
6.4.2. Control experiments 117
6.5. Time course studies of 2,4-dinitrophenol deprotonation in dichloromethane 118
6.5.1. Between 2,4-dinitrophenol and imidazole 118
6.5.2. Between 2,4-dinitrophenol and compound **1** 119
6.5.3. Between 2,4-dinitrophenol and compound **1** in the presence of tetrabutylammonium chloride 120
7. Nucleophilic additions to bis(4-nitrophenyl)carbonate 121
7.1. Reaction of bis(4-nitrophenyl)carbonate with imidazole in dry dichloromethane 121
7.1.1. Reaction of bis(4-nitrophenyl)carbonate with imidazole, monitored by UV visible spectrometry 121
7.1.2. UV visible spectroscopy control experiments 122
7.1.3. Reaction of bis(4-nitrophenyl)carbonate with imidazole monitored by NMR spectroscopy and mass spectrometry 124
7.2. Reaction of bis(4-nitrophenyl)carbonate with compound **1** monitored by UV visible spectroscopy 131
7.3. Reaction of bis(4-nitrophenyl)carbonate with compound **2** 132
7.3.1. Reaction of bis(4-nitrophenyl)carbonate with **2** monitored by UV-visible spectroscopy 132
7.3.2. Reaction of bis(4-nitrophenyl)carbonate with compound **2** monitored by NMR spectroscopy and mass spectrometry 133
7.4. Reaction of bis(4-nitrophenyl)carbonate and compound **1** in the presence of tetrabutylammonium chloride 137
7.4.1. UV-visible spectroscopy monitoring of bis(4-nitrophenyl)carbonate reacting with **1** in the presence of tetrabutylammonium chloride 137
7.4.2. Mass spectrometric study to identify reaction products 140
7.4.3. Control experiments 140
7.4.4. Time course studies of the addition to bis(4-nitrophenyl)carbonate 144
V. Supplementary References 151

I. Supplementary Notes

1. General Information

All reactions were performed under a nitrogen atmosphere. All reagents and chemicals were obtained from chemical suppliers and used without further purification. Anhydrous solvents were dispensed under nitrogen from a solvent purification system (Innovative Technologies PureSolve PS-MP-5) or were purchased from commercial suppliers.

Thin-layer chromatography (TLC) was performed using pre-coated plates (Macherey-Nagel Polygram SIL G/UV254). Visualisation was achieved by way of UV light (at 254 nm), and staining with either potassium permanganate, phosphomolybdic acid (in ethanol), or ninhydrin (in ethanol) as stains. Stained TLC plates were heated for visualization. Flash column chromatography was carried out either manually using Fluorochem 60 silica (40-60 µm particle size) or using an automated Biotage® Isolera Spektra Four with gradient elution on pre-packed silica gel Biotage® SNAP Ultra columns or ZIP Sphere columns.

Nuclear Magnetic Resonance spectra (^{1}H NMR and ^{13}C NMR) were recorded on Bruker Nano 400, Jeol ECS 400 or Bruker Avance III HD 500 Cryo with 5 mm DCH 13C–1H/D Cryo Probe (500 MHz) spectrometers. Low temperature experiments were recorded on a Jeol ECS 300 spectrometer. Some of the 2D NOESY experiments were recorded on a Bruker Avance III HD 700 (1.7mm micro-cryo) spectrometer when stated. All NMR characterisation experiments were performed at 25 °C and 1 atm unless otherwise stated. Chemical shifts (δ) are quoted in parts per million (ppm) relative to the specified deuterated solvent. Spectra were calibrated using the residual solvent peaks for $CDCl_3$ (δH: 7.26 ppm; δC: 77.16 ppm), CD_2Cl_2 (δH: 5.32 ppm; δC: 53.84 ppm) and $(CD_3)_2SO$ (δH: 2.50 ppm; δC: 39.52 ppm) as appropriate. Coupling constants (*J*) are quoted in Hz and are rounded to the nearest 0.1 Hz. Splitting patterns are abbreviated to singlet (s), doublet (d), triplet (t), quartet (q), multiplet (m) or some combination thereof.

Infrared spectra were recorded on a Bruker FT IR Alpha P spectrometer with samples applied as neat films. Absorptions maxima (ν_{max}) of interest are quoted in wavenumbers as ν in cm^{-1} for the most intense bands.

High resolution spectrometry experiments (HR-MS) were recorded by staff at the University of Bristol on a Synapt G2S Waters for nanospray experiments, MicrOTOFII Bruker Daltonics and Orbitrap Elite Thermo Scientific for electrospray ionisation experiments.

Melting points were measured on a Stuart SMP10 melting point apparatus and are uncorrected.

II. Supplementary Methods

2. General synthetic schemes

2.1 Synthesis of compound 1

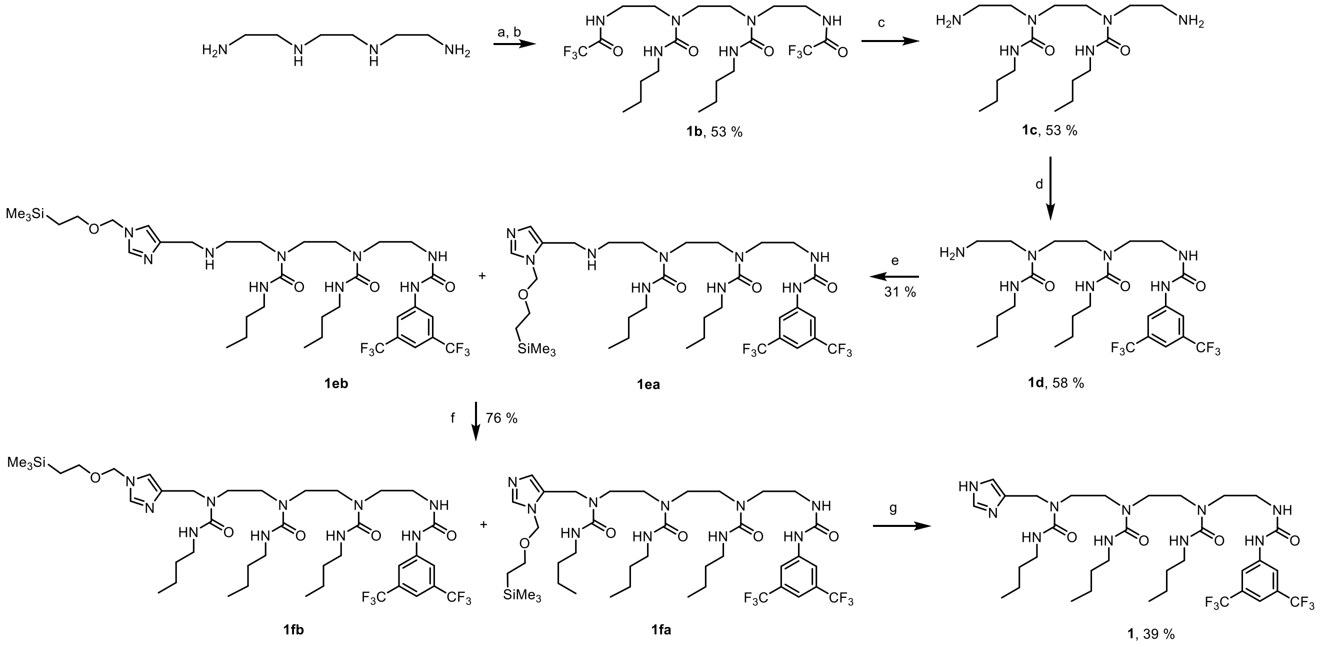

Supplementary Figure 1. Synthesis of compound **1**. Reagents and conditions: (a) CF_3CO_2Et (2 equiv.), MeOH, -78 °C to RT; (b) ^nBuNCO (4 equiv.), CH_2Cl_2, 40 °C, 16 h.; (c) NaOH aq. 0.2 M (2.2 equiv.), MeOH, 35 °C, 12 h.; (d) 3,5-$(CF_3)_2PhNCO$ (0.8 equiv.), CH_2Cl_2, RT, 12 h.; (e) RCHO (1 equiv.), CH_2Cl_2, RT, 12 h. then $NaBH(OAc)_3$ (1.12 equiv.), RT, 18 h.; (f) ^nBuNCO (1.1 equiv.), CH_2Cl_2, RT, 18 h.; (g) TBAF (3.4 equiv.), THF, 55 °C, 72h.

2.2 Synthesis of compound 2

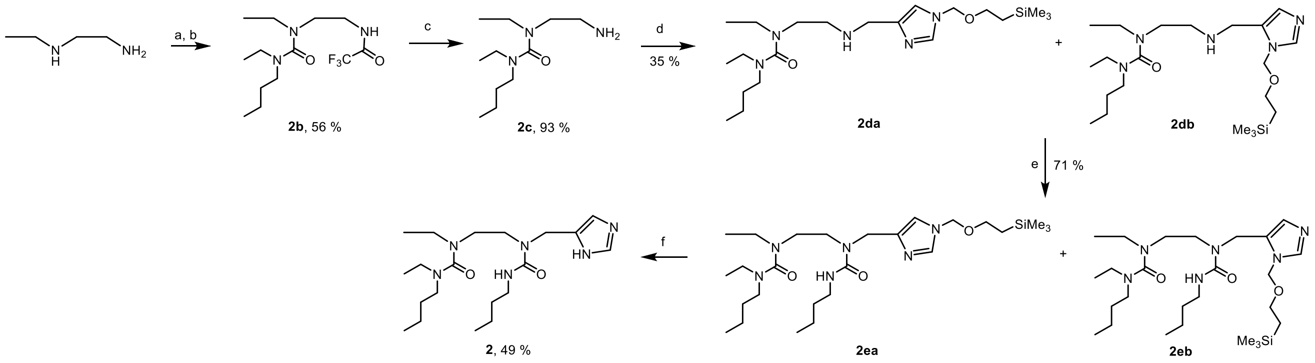

Supplementary Figure 2. Synthesis of compound **2** Reagents and conditions: (a) CF_3CO_2Et (1 equiv.), MeOH, -78 °C to RT, 4h.; (b) ROCOCl (1 equiv.), pyridine (1 equiv.), CH_2Cl_2, RT, 48 h.; (c) NaOH aq. 0.2 M (1.3 equiv.), MeOH, 35 °C, 16 h.; (d) RCHO (1 equiv.), $NaBH(OAc)_3$ (1 equiv.), CH_2Cl_2, RT, 12 h.; (e) ^nBuNCO (1.1 equiv.), CH_2Cl_2, RT, 16 h.; (f) TBAF (1.2 equiv.), THF, 55 °C, 48h.

2.3 Synthesis of compound **3**

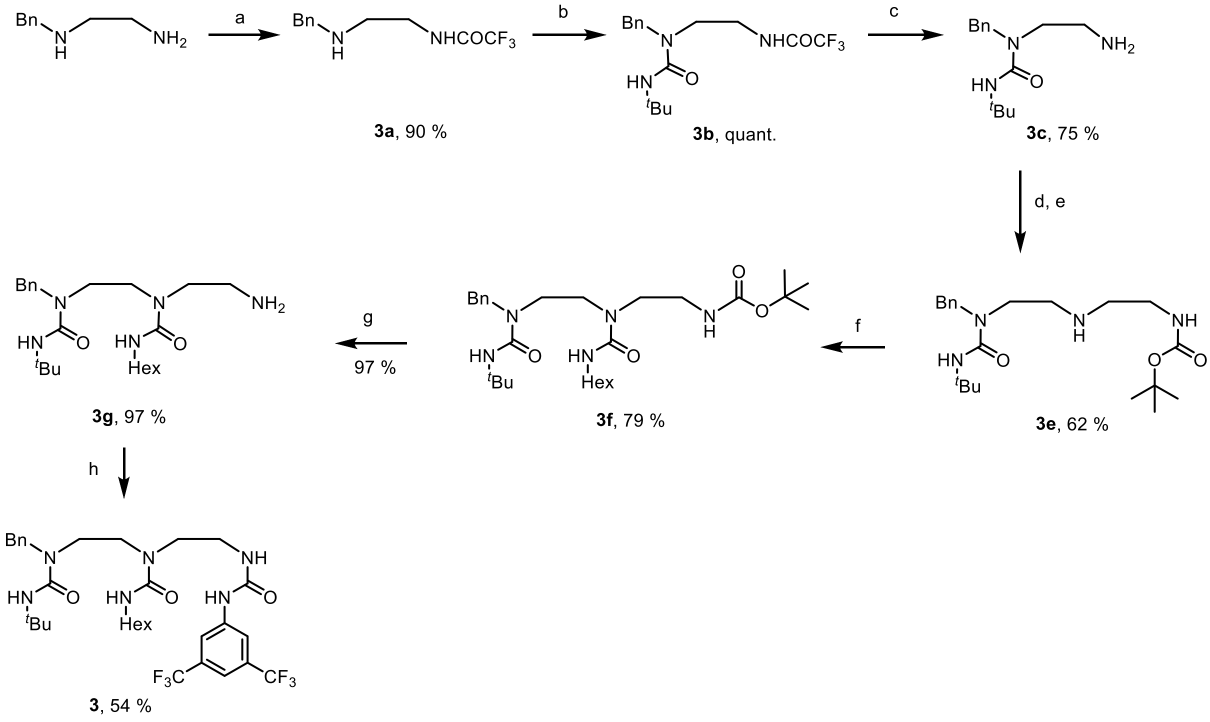

Supplementary Figure 3. Synthesis of compound **3**. Reagents and conditions: Compounds **3a-c** were made as reported.[1] (a) CF_3CO_2Et (1 equiv.), MeOH, -78 °C to RT; (b) ^{t}BuNCO (1.2 equiv.), 1,2-DCE, 50 °C; (c) NaOH aq. 0.2 M (1 equiv.), MeOH, 40 °C, 12 h.; (d) *N*-Boc-2-aminoacetaldehyde (1.5 equiv.), MeOH, RT, 12 h.; (e) $NaBH_4$ (3 equiv.), RT, 5 h.; (f) ^{n}HexNCO (1.5 equiv.), CH_2Cl_2, RT, 12 h.; (g) CF_3CO_2H (18 equiv.), CH_2Cl_2, RT, 12 h.; (h) 3,5-$(CF_3)_2$PhNCO (1.1 equiv.), CH_2Cl_2, RT, 16 h.

3. Experimental procedures and characterisation

3.1. Synthesis of compound **1**

Compound **1b**.

N,N'-bis(trifluoroacetyl)-*N'',N'''*-*bis*(*n*-butylcarbamoyl)triethylenetetramine

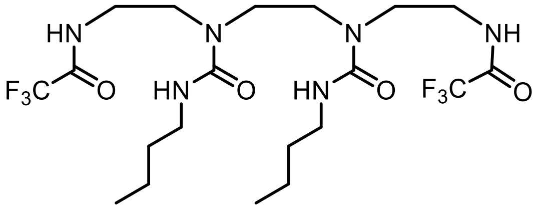

A solution of triethylenetetramine (4 mL, 26.8 mmol, 1.00 equiv, 1.6 M) in anhydrous MeOH was cooled to -78 °C then ethyl trifluoroacetate (7.7 mL, 53.6 mmol, 1 equiv. per primary amine) was added dropwise over 30 minutes. The resultant solution was stirred for 12 hours while allowing the temperature to rise to 20 °C. The mixture was concentrated under reduced pressure then purified using silica gel flash chromatography (eluent: 100 % MeOH). The *N,N'*-*bis*(trifluoroacetyl)triethylenetetramine **1b** was obtained as a colourless solid. The resulting *bis*-trifluoroacetamide (475 mg, 1.4 mmol) was dissolved in anhydrous CH_2Cl_2 to a concentration of 0.5M, then n-butyl isocyanate (630 µL, 5.6 mmol) was added to the mixture and the resultant solution was stirred for 16 hours at 40 °C. After concentration under reduced pressure, the residue was purified using silica gel flash chromatography (eluent: CH_2Cl_2:acetone:MeOH 70:20:5) then precipitation from hot $CHCl_3$ to give the title compound (395 mg, 0.73 mmol, 53 %) as a white solid. **^{1}H NMR** (400 MHz, DMSO-d_6) δ 9.49 – 9.26 (s, 2H, 2 × N*H*COCF$_3$), 6.62– 6.37 (t, *J* = 5.2 Hz, 2H, 2 × N*H*CH$_{2\ butyl}$), 3.32 – 3.29 (m, 4H, 2 × C*H*$_2$N), 3.29 – 3.23 (m, 4H, 2 × C*H*$_2$N), 3.22 – 3.16 (m, 4H, 2 × C*H*$_2$N), 3.06 – 2.95 (td, *J* = 7.0, 5.3 Hz, 4H, 2 × C*H*$_2$NH$_{butyl}$), 1.32 – 1.45 (m, 4H, 2 × C*H*$_{2\ butyl}$), 1.32 – 1.18 (m, 4H, 2 × C*H*$_{2\ butyl}$), 0.91 – 0.80 (t, *J* = 7.3 Hz, 6H, 2 × C*H*$_{3\ butyl}$). **^{13}C NMR** (101 MHz, DMSO d_6) δ 157.7 (2C, 2 × *C*=O), 156.4 (q, *J* = 36.0 Hz, 2C, 2 × *C*=OCF$_3$), 115.9 (q, *J* = 288.2 Hz, 2C, 2 × *C*F$_3$), 45.9 (2C, 2 × *C*H$_2$N), 45.7 (2C, 2 × *C*H$_2$N), 40.3 (2C, 2 × *C*H$_{2\ butyl}$), 38.6 (2C, 2 × *C*H$_2$N), 31.8 (2C, 2 × *C*H$_{2\ butyl}$), 19.5 (2C, 2 × *C*H$_{2\ butyl}$), 13.7 (2C, 2 × *C*H$_{3\ butyl}$). **FTIR (neat)** ν_{max} = 3244, 2960, 2935, 2875, 1707, 1624, 1540, 1210, 1183, 1156. **HR – MS** (ESI, positive ion mode) – *m/z* for $[C_{20}H_{34}F_6N_6O_4+Na]^+$ 559.2438, observed 559.2438. **MP** 159 °C.

Compound **1c**

N'',N'''-di(n-butylcarbamoyl)triethylenetetramine

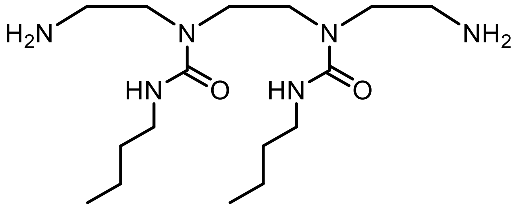

To a solution of *N,N'*-*bis*(trifluoroacetyl)- *N'',N'''*-di(n-butylcarbamoyl)triethylenetetramine **1b** (340 g, 0.63 mmol, 0.10 M in MeOH) was added 2.2 equiv. of an aqueous solution of sodium hydroxide (0.2 M). The resultant mixture was stirred for 12 hours at 35 °C. The methanol was removed under reduced pressure, the aqueous layer was extracted using a mixture ($CHCl_3$: *i*PrOH 3:1), the combined organic extracts concentrated under reduced pressure. The residue was purified using silica gel flash chromatography (eluent gradient from CH_2Cl_2: MeOH 90:10 to neat MeOH) to give the

product (113 mg, 0.33 mmol, 53 %) as a white solid. **^{1}H NMR** (400 MHz, $CDCl_3$) δ 6.81 – 6.55 (t, *J* = 5.2 Hz, 2H, 2 × N*H*CH_2 $_{Butyl}$), 3.32 – 3.15 (m, 8H, 4 × C*H*$_2$), 3.15 – 3.05 (td, *J* = 7.2, 5.2 Hz, 4H, 2 × C*H*$_2$NH), 3.04 – 2.90 (m, 4H, 2 × NH_2), 2.87 – 2.71 (t, *J* = 5.7 Hz, 4H, 2 × C*H*$_2$N), 1.48 – 1.33 (m, 4H, 2 × C*H*$_{2\ butyl}$), 1.33 – 1.16 (m, 4H, 2 × C*H*$_{2\ butyl}$), 0.89 – 0.75 (m, 6H, 2 × C*H*$_{3\ butyl}$). **^{13}C NMR** (101 MHz, $CDCl_3$) δ 159.6 (2C, *C*=O), 50.9 (2C, 2 × *C*H_2N), 46.6 (2C, 2 × *C*H_2N), 40.9 (2C, 2 × *C*H_2N), 40.6 (2 × *C*H_2NH_{butyl}),, 32.2 (2C, 2 × $CH_{2\ butyl}$), 20.2 (2C, 2 × *C*$H_{2\ butyl}$), 13.8 (2C, 2 × *C*$H_{3\ butyl}$). **FTIR (neat)** ν_{max} = 3284, 2957, 2931, 2871, 1628, 1551, 1276 cm^{-1}. **HR – MS** (ESI, positive ion mode) – *m/z* for $[C_{16}H_{36}N_6O_2+Na]^+$ 3367.2792, observed 367.2775. **MP** 143 °C.

Compound **1da**

N,N'-bis(3,5-*bis*(trifluoromethyl)phenylcarbamoyl)-*N'',N'''*-bis(n-butylcarbamoyl)triethylenetetramine

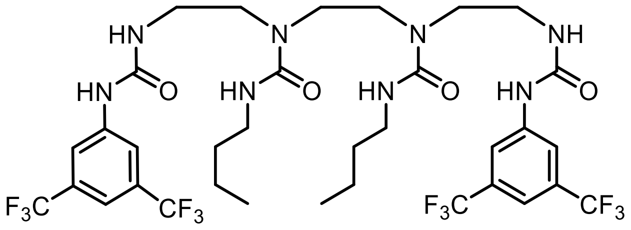

To a solution of *N'',N'''*-di(n-butylcarbamoyl)triethylenetetramine **1c** (120 mg, 0.35 mmol) in anhydrous CH_2Cl_2 (8 mL) was added dropwise a solution of *bis*(trifluoromethyl)phenyl isocyanate (48 µL, 0.28 mmol, 0.8 equiv.) in CH_2Cl_2 (1 mL) then the mixture was stirred for 12 hours at 20 °C. After concentration under reduced pressure, the residue was purified using silica gel flash chromatography (gradient CH_2Cl_2:MeOH 90:10 to 0:100) to afford *N,N'-bis*(3,5-*bis*(trifluoromethyl)phenylcarbamoyl)-*N'',N'''-bis*(n-butylcarbamoyl)triethylenetetramine **1da** (36 mg, 0.0.042 mmol, 15 %) as a solid and *N*-(3,5-*bis*(trifluoromethyl)phenylcarbamoyl)- *N'',N'''*-di(n-butylcarbamoyl)triethylenetetramine **1d** (96 mg, 0.16 mmol, 58 %) as an oil. **^{1}H NMR** (400 MHz, $CDCl_3$) δ 9.01 – 8.60 (s, 2H, 2 × N*H*Ar), 7.97 – 7.75 (m, 4H, 4 × C*H*Ar), 7.46 – 7.39 (s, 2H, 2 × C*H*Ar), 6.83 – 6.58 (m, 2H, 2 × NH_{butyl}), 6.51 – 6.30 (s, 2H, 2 × N*H*CH_2), 3.50 – 3.33 (m, 12H, 6 × CH_2), 3.25 – 3.18 (m, 4H, 2 × CH_2), 1.58 – 1.47 (m, 4H, 2 × CH_2), 1.36 – 1.26 (m, 4H, 2 × CH_2), 0.90 – 0.78 (m, 6H, 2 × CH_3). **^{13}C NMR** (101 MHz, MeOD) δ 160.5 (2C, 2 × *C*=O), 157.8 (2C, 2 × *C*=O), 143.4 (*C*$_{Ar}$), 133.1 (q, *J* = 33.0 Hz, 2C, *C*$_{Ar}$), 124.8 (q, *J* = 271.8 Hz, 2C, 2 × *C*F_3), 119.0 (2C, 2 × CH_{Ar}), 115.52 (2C, 2 × *C*H_{Ar}), 48.4 (2C, 2 × *C*H_2N), 47.5 (2C, 2 × *C*H_2N), 41.7 (2C, 2 × *C*H_2), 39.8 (2C, 2 × *C*H_2N), 33.3 (2C, 2 × *C*H_2), 21.1 (2C, 2 × *C*H_2), 14.1 (2C, 2 × *C*H_3). **FTIR (neat)** ν_{max} = 3310, 3094, 2929, 1622, 1554, 1385, 1277, 1133 cm^{-1}. **HR – MS** (ESI, positive ion mode) – *m/z* for $[C_{34}H_{42}F_{12}N_8O_4+H]^+$ 855.3210, observed 855.3197. **MP** 193-195 °C.

Compound **1d**

N-(3,5-*bis*(trifluoromethyl)phenylcarbamoyl)- *N'',N'''*-di(n-butylcarbamoyl)triethylenetetramine

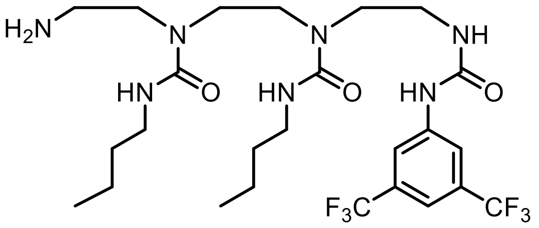

^{1}H NMR (400 MHz, $CDCl_3$) δ 9.13 – 8.84 (s, 1H, N*H*$_{Ar}$), 7.95 – 7.75 (s, 2H, 2 × C*H*$_{Ar}$), 7.38 – 7.30 (s, 1H, C*H*$_{Ar}$), 6.98 – 6.86 (t, 1H, N*H*$_{Butyl}$), 6.85 – 6.62 (d, 2H, N*H*$_{Butyl}$, N*H*CH_2), 3.36 – 3.22 (m, 8H, 4 × C*H*$_2$), 3.20 – 3.12 (m, 4H, 2 × C*H*$_2$), 3.11 – 3.04

(m, 2H, CH_2), 2.84 – 2.74 (m, 2H, CH_2), 1.48 – 1.35 (m, 4H, 2 × CH_2), 1.30 – 1.20 (m, 4H, 2 × CH_2), 0.86 – 0.73 (dt, 6H, 2 × CH_3). **^{13}C NMR** (101 MHz, $CDCl_3$) δ 160.7 (C=O), 159.2 (C=O), 156.3 (C=O), 141.8 (C_{Ar}), 132.0 (q, J = 33.1 Hz, 2C, 2 × C_{Ar}) 123.5 (q, 2C, 2 × CF_3 J = 272.7 Hz,), 117.9 (2C, 2 × CH_{Ar}), 114.8 (q, J = 4.2 Hz, CH_{Ar}), 52.4 (CH_2), 48.2 (CH_2), 47.3 (CH_2), 46.6 (CH_2), 41.6 (CH_2), 41.2 (CH_2), 40.6 (CH_2), 39.2 (CH_2NH_2), 32.2 ($CH_{2\ butyl}$), 31.9 ($CH_{2\ butyl}$), 20.3 ($CH_{2\ butyl}$), 20.2 ($CH_{2\ butyl}$), 13.9 ($CH_{3\ butyl}$), 13.8 ($CH_{3\ butyl}$). **FTIR (neat)** ν_{max} = 3294, 2959, 2931, 2878, 1691, 1619, 1560, 1387, 1276, 1130 cm^{-1}. **HR – MS** (ESI, positive ion mode) – *m/z* for $[C_{25}H_{39}F_6N_7O_3+H]^+$ 600.3091, observed 600.3072.

Compounds **1ea** and **1eb**

To a solution of **1d** (236 mg, 0.39 mmol) in dry dichloromethane (5 mL) was added the 1-[2-(trimethylsilyl)ethoxymethyl]imidazole-4(5)-carboxaldehydes (synthesised following a reported procedure[2]) (90 mg, 0.39 mmol) and the mixture was stirred under nitrogen at ambient temperature for 12 hours. Sodium triacetoxyborohydride (93 mg, 0.44 mmol) was added, the mixture was stirred at ambient temperature for 18 hours, after which water was added and the resulting solution was extracted with dichloromethane. The organic phase was dried (Na_2SO_4), the solid was filtered off and the organic phase was concentrated under reduced pressure. The residue was purified by chromatography on silica (eluent dichloromethane : methanol 95 :5) to afford the isomeric products as oils (102 mg, 0.12 mmol, 31 % as mixture of isomers that were separated by chromatography (74 mg **1eb** and 28 mg **1ea** respectively, 0.72 : 0.27 ratio).

Compound **1eb**

1-(2-(3-(3,5-bis(trifluoromethyl)phenyl)ureido)ethyl)-3-n-n-butyl-1-(2-(3-butyl-1-(2-(((1-((2-(trimethylsilyl)ethoxy)methyl)-1H-imidazol-4-yl)methyl)amino)ethyl)ureido)ethyl)urea

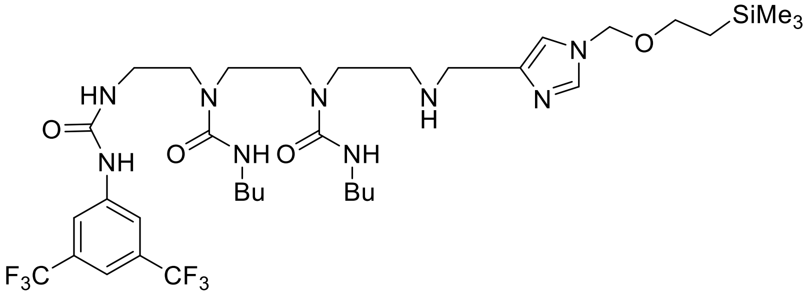

Yield = 74 mg. **^{1}H NMR** (400 MHz, $CDCl_3$) δ 9.25 (s, 1H, N*H*Ph), 7.94 (s, 2H, 2 × CH_{Ph}), 7.55 (s, 1H, CH_{Imid}), 7.38 (s, 1H, CH_{Ph}), 6.99 (s, 1H, CH_{Imid}), 6.79 (bs, 1H, N*H*Bu), 6.68 (bs, 1H, N*H*Bu), 6.60 (bs, 1H, N*H*), 5.31 (s, 2H, Imid-CH_2O), 3.89 (s, 2H, NHCH_2Imid), 3.44 (t, J = 8.24 Hz, 2H, OCH_2CH$_2$Si), 3.40 – 3.22 (m, 8H, 4 × CH_2N), 3.21 – 3.14 (m, 2H, CH_{2Bu}NH), 3.14 – 3.07 (m, 2H, CH_{2Bu}NH), 2.88 – 2.78 (m, 2H, CH_2NH), 1.53 – 1.44 (m, 2H, CH_{2Bu}), 1.44 – 1.35 (m, 2H, CH_{2Bu}), 1.35 – 1.21 (m, 4H, 2 × CH_{2Bu}), 0.90 – 0.78 (m, 8H, 2 × CH_3Bu, CH_2Si), -0.08 (s, 9H, (CH_3)$_3$Si). **^{13}C NMR** (101 MHz, $CDCl_3$) δ 160.0 (*C*=O), 159.0 (*C*=O), 156.4 (*C*=O), 141.8 (C_{Ph}), 138.9 (CH_{Imid}), 131.9 (q, J = 33.0 Hz, 2C, 2 × *C*CF$_3$), 129.9 (CH_{Imid}), 128.2 (C_{Imid}), 123.5 (q, J = 272.6 Hz, 2C, 2 × CF_3), 117.8 (2C, 2 × CH_{Ph}), 114.8 (CH_{Ph}), 74.3 (Imid-CH_2O), 66.4 (OCH_2CH$_2$Si), 48.9 (CH_2N), 48.3 (CH_2N), 48.2 (CH_2N), 47.3 (CH_2N), 46.9 (CH_2N), 42.3 (NHCH_2Imid), 41.0 (CH_{2Bu}NH), 40.7 (CH_{2Bu}NH), 39.2 (CH_2N), 32.2 (CH_{2Bu}), 31.9 (CH_{2Bu}), 20.2 (CH_{2Bu}), 20.2 (CH_{2Bu}), 17.8 (CH_2Si), 13.9 (CH_{3Bu}), 13.8 (CH_{3Bu}), -1.4 (3C, (CH_3)$_3$Si). **HR – MS** (ESI, positive ion mode) – m/z for $[C_{35}H_{57}N_9F_6O_4SiNa]^+$ 832.4099, found 832.4080.

Compound **1ea**

1-(2-(3-(3,5-bis(trifluoromethyl)phenyl)ureido)ethyl)-3-butyl-1-(2-(3-butyl-1-(2-(((1-((2-(trimethylsilyl)ethoxy)methyl)-1H-imidazol-5-yl)methyl)amino)ethyl)ureido)ethyl)urea

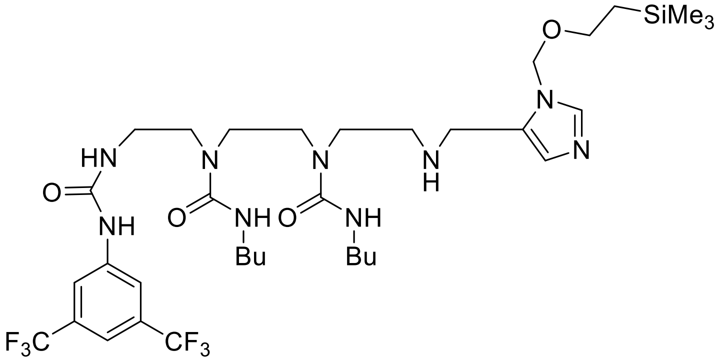

Yield = 28 mg. **^{1}H NMR** (400 MHz, $CDCl_3$) δ 9.12 (s, 1H, N*H*Ph), 7.95 (s, 2H, 2 × C*H*$_{Ph}$), 7.54 (s, 1H, C*H*$_{Imid}$), 7.39 (s, 1H, C*H*$_{Ph}$), 7.14 (s, 1H, N*H*), 6.98 (s, 1H, N*H*), 6.92 (s, 1H, C*H*$_{Imid}$), 6.56 (s, 1H, N*H*), 5.21 (s, 2H, Imid-C*H*$_2$O), 3.76 (s, 2H, NHC*H*$_2$Imid), 3.52 – 3.44 (m, 2H, OC*H*$_2$CH$_2$Si), 3.34 (s, 8H, 4 × C*H*$_2$N), 3.23 – 3.17 (m, 2H, C*H*$_{2Bu}$NH), 3.17 – 3.09 (m, 2H, C*H*$_{2Bu}$NH), 2.88 – 2.79 (m, 2H, C*H*$_2$NH), 1.54 – 1.46 (m, 2H, C*H*$_{2Bu}$), 1.46 – 1.38 (m, 2H, C*H*$_{2Bu}$), 1.35 – 1.23 (m, 4H, 2 × C*H*$_{2Bu}$), 0.92 – 0.81 (m, 8H, 2 × C*H*$_3$Bu, C*H*$_2$Si), -0.02 (s, 9H, (C*H*$_3$)$_3$Si). **^{13}C NMR** (101 MHz, $CDCl_3$) δ 160.4 (*C*=O), 159.1 (*C*=O), 156.3 (*C*=O), 141.9 (*C*$_{Ph}$), 139.4 (*C*$_{Imid}$), 137.4 (*C*H$_{Imid}$), 132.0 (q, *J* = 33.0 Hz, 2C, 2 × *C*CF$_3$), 123.5 (q, *J* = 272.7 Hz, 2C, 2 × *C*F$_3$), 117.9 (2C, 2 × *C*H$_{Ph}$), 116.7 (*C*H$_{Imid}$), 114.7 (*C*H$_{Ph}$), 76.1 (Imid-*C*H$_2$O), 66.7 (O*C*H$_2$CH$_2$Si), 48.5 (2C, *C*H$_2$N, NH*C*H$_2$Imid), 47.6 (*C*H$_2$N), 47.0 (*C*H$_2$N), 46.4 (*C*H$_2$N), 41.1 (2C, *C*H$_{2Bu}$NH, *C*H$_2$N), 40.7 (*C*H$_{2Bu}$NH), 39.2 (*C*H$_2$N), 32.2 (*C*H$_{2Bu}$), 32.0 (*C*H$_{2Bu}$), 20.3 (*C*H$_{2Bu}$), 20.2 (*C*H$_{2Bu}$), 17.8 (*C*H$_2$Si), 13.9 (*C*H$_{3Bu}$), 13.8 (*C*H$_{3Bu}$), -1.3 (3C, (*C*H$_3$)$_3$Si). **HR – MS** (ESI, positive ion mode) – m/z for $[C_{35}H_{57}N_9F_6O_4SiNa]^+$ 832.4099, found 832.4079.

Compound **1fb**

1-(2-(3-(3,5-bis(trifluoromethyl)phenyl)ureido)ethyl)-3-butyl-1-(2-(3-butyl-1-(2-(3-butyl-1-((1-((2-(trimethylsilyl)ethoxy)methyl)-1H-imidazol-4-yl)methyl)ureido)ethyl)ureido)ethyl)urea

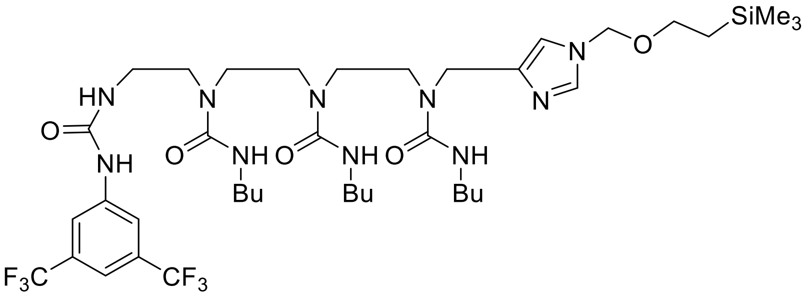

To a solution of **1eb** (90 mg, 0.11 mmol) in dry dichloromethane (3 mL) was added n-butyl isocyanate (0.014 mL, 0.12 mmol) and the mixture was stirred under nitrogen for 18 h. Upon completion monitored by thin layer chromatography, the mixture was concentrated under reduced pressure and the residue was purified by chromatography on silica (eluent dichloromethane:methanol 9:1) to afford the product **1fb** as a foam (76 mg, 76 %). **^{1}H NMR** (400 MHz, $CDCl_3$) δ 9.13 (s, 1H, N*H*Ph), 7.96 (s, 2H, 2 × C*H*$_{Ph}$), 7.67 (s, 1H, C*H*$_{imid}$), 7.44 (s, 1H, C*H*$_{Ph}$), 7.06 (s, 1H, C*H*$_{imid}$), 6.54 (s, 1H, N*H*CH$_{2Bu}$), 6.45 (t, *J* = 4.9 Hz, 1H, N*H*CH$_{2Bu}$), 6.35-5.75 (bs, 1H, NH), 6.34 (s, 1H, N*H*CH$_{2Bu}$), 5.32 (s, 2H, Imid-C*H*$_2$-O), 4.55 (s, 2H, N-C*H*$_2$-Imid), 3.50 – 3.45 (m, 2H, OC*H*$_2$CH$_2$Si), 3.44 – 3.14 (m, 16H, 8 × C*H*$_2$N), 3.02 (s, 2H, C*H*$_2$N), 1.54 – 1.43 (m, 6H, 3 × C*H*$_{2Bu}$), 1.37 –

1.26 (m, 6H, 3 × CH_{2Bu}), 1.01 – 0.84 (m, 11H, 3 × CH_{3Bu}, CH_2Si), -0.04 (s, 9H, (CH_3)$_3$Si). **^{13}C NMR** (101 MHz, CDCl$_3$) δ 158.9 (*C*=O), 158.9 (*C*=O), 158.6 (*C*=O), 156.4 (*C*=O), 141.6 (*C*$_{Ph}$), 139.1 (*C*H$_{Imid}$), 132.1 (q, *J* = 33.0 Hz, 2C, 2 × *C*CF$_3$), 129.2 (*C*H$_{Imid}$), 128.7 (*C*$_{Imid}$), 123.4 (q, *J* = 272.6 Hz, 2C, 2 × *C*F$_3$), 117.9 (2C, 2 × *C*H$_{Ph}$), 115.0 (*C*H$_{Ph}$), 74.5 (1C, Imid-*C*H$_2$-O), 66.6 (1C, O*C*H$_2$CH$_2$Si), 48.2 (1C, *C*H$_2$N), 47.4 (1C, *C*H$_2$N), 47.3 (1C, *C*H$_2$N), 46.8 (1C, *C*H$_2$N), 45.5 (1C, *C*H$_2$N), 44.1 (1C, *C*H$_2$N), 41.0 (1C, *C*H$_2$NH), 41.0 (N-C*H$_2$*-Imid), 40.9 (1C, *C*H$_2$NH), 39.3 (1C, *C*H$_2$NH), 32.2 (*C*H$_{2Bu}$), 32.1 (*C*H$_{2Bu}$), 32.0 (*C*H$_{2Bu}$), 20.2 (2C, 2 × *C*H$_{2Bu}$), 20.2 (*C*H$_{2Bu}$), 17.9 (1C, *C*H$_2$Si), 13.9 (1C, *C*H$_{3Bu}$), 13.9 (1C, *C*H$_{3Bu}$), 13.8 (1C, *C*H$_{3Bu}$), -1.4 (3C, (*C*H$_3$)$_3$Si). **HR – MS** (ESI, positive ion mode) – m/z for $[C_{40}H_{66}N_{10}F_6O_5SiNa]^+$ 931.4783, found 931.4763.

Compound **1fa**

1-(2-(3-(3,5-bis(trifluoromethyl)phenyl)ureido)ethyl)-3-butyl-1-(2-(3-n-butyl-1-(2-(3-n-butyl-1-((1-((2-(trimethylsilyl)ethoxy)methyl)-1H-imidazol-5-yl)methyl)ureido)ethyl)ureido)ethyl)urea

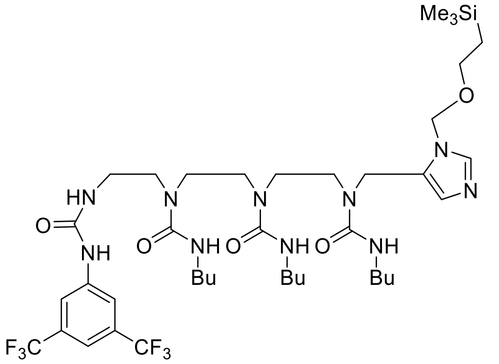

To a solution of **1ea** (28 mg, 0.034 mmol) in dry dichloromethane (0.5 mL) was added n-butyl isocyanate (0.04 mL, 0.034 mmol) and the mixture was stirred under nitrogen for 18 h. Upon completion monitored by thin layer chromatography, the mixture was concentrated under reduced pressure and the residue was purified by chromatography on silica (eluent dichloromethane:methanol 9:1) to afford the product **1fa** as a foam (23 mg, 76 %). **^{1}H NMR** (400 MHz, CDCl$_3$) δ 9.15 (s, 1H, N*H*Ph), 7.96 (s, 2H, 2 × C*H*$_{Ph}$), 7.83 – 7.59 (bs, 1H, C*H*$_{imid}$), 7.39 (s, 1H, C*H*$_{Ph}$), 7.13 (s, 1H, C*H*$_{imid}$), 6.97 – 6.76 (bs, 2H, 2 × N*H*), 6.65 (bs, 1H, N*H*), 6.21 (bs, 1H, N*H*), 5.26 (s, 2H, Imid-C*H$_2$*-O), 4.30 (s, 2H, N-C*H$_2$*-Imid), 3.50 (t, *J* = 8.12 Hz, OC*H$_2$*CH$_2$Si), 3.44 – 3.26 (m, 12H, 6 × C*H$_2$*N), 3.26 – 3.11 (m, 6H, 3 × C*H$_2$*N), 1.57 – 1.44 (m, 6H, 3 × C*H*$_{2Bu}$), 1.39 – 1.27 (m, 6H, 3 × C*H*$_{2Bu}$), 0.94 – 0.82 (m, 11H, 3 × C*H*$_{3Bu}$, C*H$_2$*Si), -0.04 (s, 9H, (C*H$_3$*)$_3$Si). **^{13}C NMR** (101 MHz, CDCl$_3$) δ 159.6 (*C*=O), 159.1 (*C*=O), 159.0 (*C*=O), 156.4 (*C*=O), 141.8 (*C*$_{Ph}$), 138.2 (*C*H$_{Imid}$), 132.0 (q, *J* = 33.0 Hz, 2C, 2 × *C*CF$_3$), 127.7 (*C*H$_{Imid}$), 126.7 (*C*$_{Imid}$), 123.5 (q, *J* = 272.7 Hz, 2C, 2 × *C*F$_3$), 117.9 (2C, 2 × *C*H$_{Ph}$), 114.7 (*C*H$_{Ph}$), 76.8 (1C, Imid-*C*H$_2$-O), 67.2 (1C, O*C*H$_2$CH$_2$Si), 48.6 (1C, *C*H$_2$N), 47.8 (3C, 3 × *C*H$_2$N), 47.3 (2C, 2 × *C*H$_2$N), 41.1 (1C, *C*H$_2$NH), 40.9 (N-C*H$_2$*-Imid), 40.9 (1C, *C*H$_2$NH), 39.6 (1C, *C*H$_2$NH), 32.2 (*C*H$_{2Bu}$), 32.2 (*C*H$_{2Bu}$), 32.0 (*C*H$_{2Bu}$), 20.3 (2C, 2 × *C*H$_{2Bu}$), 20.2 (*C*H$_{2Bu}$), 17.8 (1C, *C*H$_2$Si), 13.9 (2C, 2 × *C*H$_{3Bu}$), 13.8 (*C*H$_{3Bu}$), -1.4 (3C, (*C*H$_3$)$_3$Si). **HR – MS** (ESI, positive ion mode) – m/z for $[C_{40}H_{66}N_{10}F_6O_5SiNa]^+$ 931.4783, found 931.4763, m/z for $[C_{40}H_{67}N_{10}F_6O_5Si]^+$ 909.4964, found 909.4948, m/z for $[C_{40}H_{66}N_{10}F_6O_5SiK]^+$ 947.4523, found 947.4501.

Mixture of compounds **1fa** and **1fb**

The reaction carried out on a mixture of isomers **1ea** and **1eb** led to the expected mixture of **1fa** and **1fb** isomers but these were not separable by chromatography. However each of **1fa** and **1fb** could be characterised by using purified isomers **1ea** and **1eb** as starting materials. Nonetheless if a mixture of **1fa** and **1fb** was taken forward to the next synthetic step then both isomers lead to compound **1**.

Compound **1**

1-(2-(1-((1H-imidazol-4-yl)methyl)-3-n-butylureido)ethyl)-1-(2-(1-(2-(3-(3,5-bis(trifluoromethyl)phenyl)ureido)ethyl)-3-n-butylureido)ethyl)-3-n-butylurea

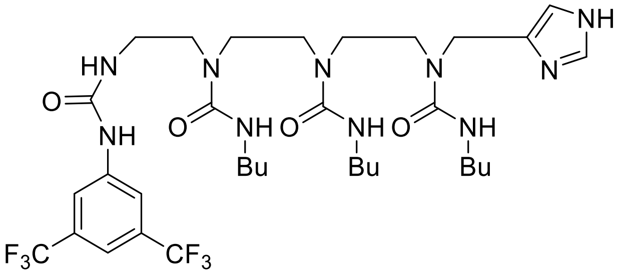

To a solution of **1fa/1fb** (160 mg, 0,176 mmol) in dry tetrahydrofuran (10 mL) was added a solution of tetrabutylammonium fluoride (0.6 mL, 0.6 mmol, 3.4 equiv.) and the mixture was stirred under nitrogen at 55 °C for 3 days. The resulting mixture was concentrated under reduced pressure, the residue was redissolved in dichloromethane, the organic phase was washed with water, dried (Na_2SO_4), concentrated under reduced pressure, the residue was purified by flash chromatography on silica (eluent dichloromethane:methanol 9:1) to yield the product **1** as a white solid (53 mg, 0.068 mmol, 39 %). **^{1}H NMR** (500 MHz, $CDCl_3$) *δ* 9.21 (s, 1H, N*H*Ph), 7.91 (s, 2H, 2 × CH_{Ph}), 7.60 (s, 1H, CH_{Imid}), 7.41 (s, 1H, CH_{Ph}), 6.96 (s, 1H, CH_{Imid}), 6.78 (s, 1H, N*H*), 6.68 - 6.13 (m, 2H, 2 × N*H*), 6.55 (s, 2H), 4.33 (s, 2H, CH_2Imid), 3.43 – 3.34 (m, 2H, CH_2N), 3.34 – 3.22 (m, 8H, 4 × CH_2N), 3.22 - 3.11 (m, 8H, 4 × CH_2N), 1.55 – 1.43 (m, 6H, 3 × C$H_2$$CH_3$), 1.36 – 1.25 (m, 6H, 3 × C$H_2$$CH_3$), 0.9 – 0.79 (m, 9H, 3 × CH_3). **^{13}C NMR** (126 MHz, $CDCl_3$) *δ* 159.6 (*C*=O), 159.1 (*C*=O), 158.9 (*C*=O), 156.6 (*C*=O), 141.5 (C_{Ph}), 136.8 (C_{Imid}), 135.4 (CH_{Imid}), 132.1 (q, *J* = 33.1 Hz, 2C, 2 × CCF_3), 123.43 (q, *J* = 272.6 Hz, 2C, 2 × CF_3), 117.9 (2C, 2 × CH_{Ph}), 115.7 (CH_{Imid}), 115.1 (1C, CH_{Ph}), 48.3 (CH_2N), 47.6 (2 × CH_2N), 47.3 (2 × CH_2N), 45.7 (bs, CH_2Imid), 41.1 (CH_2NH), 41.0 (CH_2NH), 40.9 (CH_2NH), 39.3 (CH_2NH), 32.2 (2 × CH_2), 31.9 (CH_2), 20.2 (CH_2), 20.2 (2 × CH_2), 13.9 (CH_3), 13.8 (CH_3), 13.7 (CH_3).). **FTIR** (neat) $ν_{max}$ = 3293, 3100, 2962, 2931, 2871, 1613, 1539, 1385, 1275, 1174, 1128, 681 cm^{-1}.**HR – MS** (ESI, positive ion mode) – m/z for $[C_{34}H_{52}N_{10}F_6O_4Na]^+$ 801.3969, found 801.3942. **HR – MS** (ESI, negative ion mode) – m/z for $[C_{34}H_{51}N_{10}F_6O_4]^-$ 777.4004, found 777.3991. **MP** 109 -111 °C.

3.2. Synthesis of compound **2**

Compound **2b**

N-Trifluoroacetyl-*N*'-(n-butylcarbamoyl)-*N*'-ethyl-*N*''-ethylethane-1,2-diamine

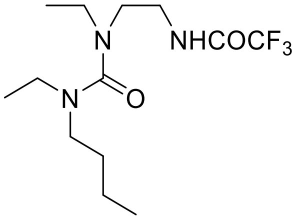

To a solution of *N*-ethylethylenediamine (200 µL, 1.9 mmol) in anhydrous MeOH (4 mL) at -78 °C was added dropwise ethyl trifluoroacetate (225 µL, 1.9 mmol, 1.01 equiv.). The mixture was stirred for 4 hours while allowing the temperature to raise to 20 °C. The solvent was removed under reduced pressure to give the *N*-trifluoroacetyl-*N*'-ethylethane-1,2-diamine **2a** used as is.

Preparation of the carbamoyl chloride: to a solution of triphosgene (532 mg, 1.8 mmol, 0.5 equiv) at 0 °C in dry dichloromethane (20 mL) was added dropwise pyridine (0.29 mL, 3.6 mmol), then *N*-ethylbutan-1-amine (0.5 mL, 3.6 mmol), and the solution was stirred at ambient temperature until no more amine (monitored by thin layer chromatography). The mixture was washed with aqueous HCl 1M, the organic phase was dried (Na_2SO_4), concentrated under reduced pressure, the carbamoyl chloride was used with no further purification.

To a solution of *N*-trifluoroacetyl-*N'*-ethylethane-1,2-diamine **2a** (662 mg, 3.6 mmol) in dry dichloromethane (15 mL) was added the carbamoyl chloride (3.6 mmol) and anhydrous pyridine (0,5 mL, 3.6 mmol). The mixture was stirred at ambient temperature for 48 hours. The organic phase was extracted with water, dried (Na_2SO_4), concentrated under reduced pressure, the residue was purified by flash chromatography (SiO_2) (eluent dichloromethane methanol 100:0 to 95:5) to give the product **2b** as an oil (617 mg, 2 mmol, 56 %). **^{1}H NMR** (400 MHz, $CDCl_3$) δ 9.12 (s, 1H, N*H*$COCF_3$), 3.50 – 3.38 (m, 4H, 2 × CH_2N), 3.25 – 3.08 (m, 6H, 2 × CH_2CH_3, CH_2Bu), 1.56 – 1.44 (m, 2H, CH_{2Bu}), 1.28 (h, *J* = 7.3 Hz, 2H, CH_{2Bu}), 1.17 (t, *J* = 7.2 Hz, 3H, CH_3), 1.12 (t, *J* = 7.1 Hz, 3H, CH_3), 0.91 (t, *J* = 7.3 Hz, 3H, CH_{3Bu}). **^{13}C NMR** (101 MHz, $CDCl_3$) δ 157.8 (q, *J* = 36.9 Hz, *C*=OCF_3), 116.1 (q, *J* = 287.5 Hz, CF_3), 53.6 (CH_2N), 47.9 (CH_2N), 44.0 (CH_2N), 43.3 (CH_2N), 39.0(CH_2N), 30.1 (CH_{2Bu}), 20.3 (CH_{2Bu}), 13.9 (CH_{3Bu}), 13.3 (CH_3), 13.2 (CH_3).). **FTIR** (neat) ν_{max} = 3257, 2963, 2933, 1720, 1620, 1422, 1259, 1154, 1020, 795 cm^{-1}. **HR – MS** (ESI, positive ion mode) – m/z for $[C_{13}H_{24}F_3N_3O_2Na]^+$ 334.1713, found 334.1698.

Compound **2c**.

N'-(n-butylcarbamoyl)-*N*'-ethyl-*N*''-ethylethane-1,2-diamine

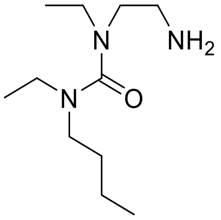

To a solution of *N*-trifluoroacetyl-N'-(n-butylcarbamoyl)-N'-ethyl-N''-ethylethane-1,2-diamine **2b** (617 mg, 2 mmol) in methanol (15 mL) was added aqueous sodium hydroxide 0.2M solution (9 mL, 2.6 mmol of NaOH, 1.3 equiv) and the mixture was stirred at 35 °C for 16 hours. The mixture was concentrated under reduced pressure, the residue was dissolved in dichloromethane, the organic phase was washed with water, dried (Na_2SO_4), filtered and concentrated under reduced pressure to give the product **2c** (400 mg, 1.86 mmol, 93 %). **^{1}H NMR** (400 MHz, $CDCl_3$) *δ* 3.39 – 3.06 (m, 8H, 4 × CH_2N), 2.78 (t, *J* = 6.5 Hz, 2H), 1.52 (bs, 2H, NH_2), 1.50 – 1.41 (m, 2H, $CH_{2\ Bu}$), 1.31 – 1.20 (m, 2H, $CH_{2\ Bu}$), 1.11 – 1.04 (m, 6H, 2 × CH_3), 0.88 (t, *J* = 7.3 Hz, 3H, $CH_{3\ Bu}$). **^{13}C NMR** (101 MHz, $CDCl_3$) δ 165.4 (*C*=O), 50.6 (CH_2N), 47.5 (CH_2N), 44.1 (CH_2N), 42.9 (CH_2N), 40.4 (CH_2NH_2), 30.2 (CH_{2Bu}), 20.3 (CH_{2Bu}), 14.0 ($CH_{3\ Bu}$), 13.3 (CH_3), 13.3 (CH_3).). **FTIR** (neat) ν_{max} = 3375, 2961, 2933, 2878, 1617, 1421, 1378, 1260, 1094, 799 cm^{-1}. **HR – MS** (ESI, positive ion mode) – m/z for $[C_{11}H_{26}N_3O]^+$ 216.2070, found 216.2064.

Compounds **2da** and **2db**

1-n-Butyl-1,3-diethyl-3-(2-(((1-((2-(trimethylsilyl)ethoxy)methyl)-1H-imidazol-4-yl)methyl)amino)ethyl)urea, **2da**

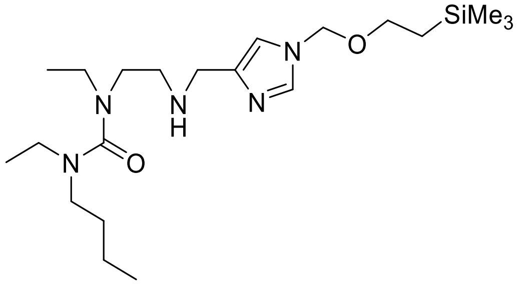

To a solution of *N'*-(n-butylcarbamoyl)-*N*'-ethyl-*N*''-ethylethane-1,2-diamine **2c** (210 mg, 1 mmol) in dry dichloromethane (6 mL) was added the 1-[2-(trimethylsilyl)ethoxymethyl]imidazole-4(5)-carboxaldehydes (220 mg, 1 mmol) then sodium triacetoxyborohydride $NaBH(OAc)_3$ (212 mg, 1 mmol). The mixture was stirred at ambient temperature over 12 hours then water was added. The organic phase was extracted with water, dried (Na_2SO_4), filtered and concentrated. The residue was purified by chromatography on silica (eluent dichloromethane:methanol 9:1) to give the product isomers, the major isomer **2da** was isolated pure (120 mg, 0,28 mmol, 28 %), the spectra of which are provided (the other fractions were the mixed isomers **2da** with **2db**). **^{1}H NMR** (400 MHz, $CDCl_3$) δ 7.53 (d, *J* = 1.1 Hz, 1H, NC*H*N), 6.93 (s, 1H, CH_{imid}), 5.34 (bs, 2H, NCH_2O), 4.59 – 4.40 (bs, N*H*), 3.84 (bs, 2H, $NHCH_2$Imid), 3.48 – 3.39 (m, 2H, OCH_2CH_2Si), 3.22 (t, *J* = 6.4 Hz, 2H, NCH_2), 3.17 – 3.03 (m, 6H, 2 × NCH_2CH_3, NCH_{2Bu}), 2.75 (t, *J* = 6.4 Hz, 2H, NCH_2), 1.51 – 1.37 (m, 2H, CH_{2Bu}), 1.30 – 1.19 (m, 2H, CH_{2Bu}), 1.10 – 1.02 (m, 6H, 2 × CH_3CH_2N), 0.90 – 0.83 (m, 5H, CH_{3Bu}, CH_2Si), -0.05 (s, 9H, $(CH_3)_3Si$). **^{13}C NMR** (101 MHz, $CDCl_3$) δ 165.3 (*C*=O), 138.6 (NCHN), 129.2 (CH_{Imid}), 128.9 (C_{Imid}), 74.4 (NCH_2O), 66.2 (OCH_2CH_2Si), 47.5 (NCH_{2Bu}), 46.8 (NCH_2), 46.7 (NCH_2), 44.2 (NCH_2CH_3), 42.9 (NCH_2CH_3), 42.3 ($NHCH_2$Imid), 30.2 (CH_{2Bu}), 20.3 (CH_{2Bu}), 17.8 (CH_2Si), 14.0 (CH_{3Bu}), 13.3 (CH_3CH_2N), 13.3 (CH_3CH_2N), -1.4 (3C, $(CH_3)_3Si$).). **FTIR** (neat) ν_{max} = 2956, 1631, 1418, 1248, 1089, 834, 661 cm^{-1}.**HR – MS** (ESI, positive ion mode) – m/z for $[C_{21}H_{43}N_5O_2SiNa]^+$ 448.3078, found 448.3063.

Compound **2ea**

1-n-Butyl-3-(2-(3-n-butyl-1-((1-((2-(trimethylsilyl)ethoxy)methyl)-1H-imidazol-4-yl)methyl)ureido)ethyl)-1,3-diethylurea

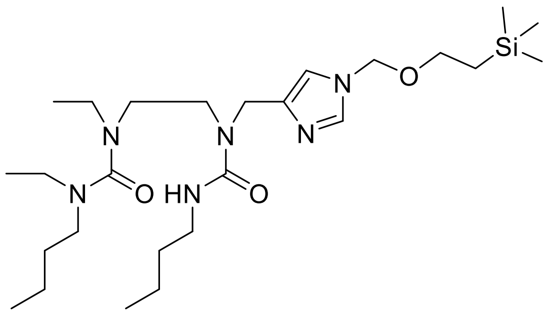

To a solution of **2da and 2db** (mixture of isomers) (150 mg, 0.35 mmol) in dry dichloromethane (5 mL) was added n-butylisocyanate (0.044 mL, 0.39 mmol) and the mixture was stirred at ambient temperature for 16 hours. The mixture was concentrated under reduced pressure, the residue was purified by chromatography on silica (eluent dichloromethane methanol 9:1) to give the expected product as a mixture of isomers **2ea** and **2eb** (130 mg, 0.245 mmol,

71 %) that were difficult to separate by chromatography. The NMR description of the major isomer **2ea** is provided below. **^{1}H NMR** (500 MHz, $CDCl_3$) δ 7.66 (d, *J* = 1.2 Hz, 1H, $NCHN_{imid}$), 7.04 – 6.99 (m, 1H, CH_{imid}), 6.79 (t, *J* = 5.2 Hz, 1H, N*H*), 5.36 (s, 2H, NCH_2O), 4.64 (s, 2H, NCH_2N), 3.56 – 3.47 (m, 2H, OCH_2CH_2Si), 3.32- 3.26 (m, 2H, $CH_{2Bub}NH$), 3.21 – 3.15 (m, 4H, NCH_2CH_3, NCH_2CH_2N), 3.14 – 3.07 (m, 2H, $NCH_{2Bu\,a}$), 3.08-3.02 (m, 2H, NCH_2CH_3), 2.93 – 2.84 (m, 2H, NCH_2CH_2N), 1.64 – 1.54 (m, 2H, $CH_{2Bu\,b}$), 1.54 – 1.46 (m, 2H, $CH_{2Bu\,a}$), 1.44 – 1.38 (m, 2H, $CH_{2Bu\,b}$), 1.33 – 1.27 (m, 2H, $CH_2CH_{3Bu\,a}$), 1.13 – 1.06 (m, 6H, CH_3CH_2N, CH_3CH_2N), 0.97 – 0.91 (m, 8H, $CH_{3Bu\,b}$, $CH_{3Bu\,a}$, CH_2Si), 0.00 (bs, 9H, $(CH_3)_3Si$). **^{13}C NMR** (126 MHz, $CDCl_3$) δ 165.2 (*C*=O), 157.9 (*C*=ONH), 139.0 ($NCHN_{Imid}$), 129.2 (CH_{Imid}), 129.0 (C_{imid}), 74.1 (NCH_2O), 66.0 (OCH_2), 47.3 ($NCH_{2Bu\,a}$), 45.6 (NCH_2CH_3), 45.1 (NCH_2CH_2N), 43.6 (NCH_2CH_2N), 42.7 (NCH_2CH_3), 40.9 ($CH_{2Bub}NH$), 39.5 (NCH_2N), 32.0 ($CH_{2Bu\,b}$), 30.0 ($CH_{2Bu\,a}$), 20.2 ($CH_{2Bu\,b}$), 20.2 ($CH_2CH_{3Bu\,a}$), 17.8 (CH_2Si), 13.9 (2C, $CH_{3Bu\,a}$, $CH_{3Bu\,b}$), 13.5 (CH_3CH_2N), 13.1 (CH_3CH_2N), -1.4 (3C, $(CH_3)_3Si$).). **FTIR** (neat) $ν_{max}$ = 3315, 2956, 2929, 2871, 1622, 1548, 1420, 1377, 1248, 1090, 835, 664 cm^{-1}. **HR – MS** (ESI, positive ion mode) – m/z for $[C_{21}H_{43}N_5O_2SiNa]^+$ 448.3078, found 448.3069.

Compound **2**.

1-(2-(1-((1H-Imidazol-5-yl)methyl)-3-n-butylureido)ethyl)-3-butyl-1,3-diethylurea.

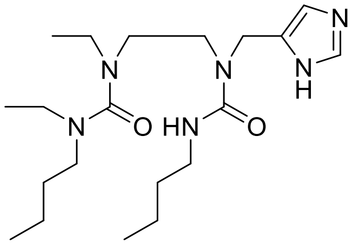

To a solution of **2ea** and **2eb** (mixture of isomers) (130 mg, 0.25 mmol) in anhydrous tetrahydrofuran (10 mL) was added tetrabutylammonium fluoride TBAF 1M solution in THF (0.3 mL, 1.2 equiv) and the mixture was stirred at 55 °C for 2 days. The mixture was concentrated, the residue was dissolved in dichloromethane, the organic phase was washed with water, dried (Na_2SO_4), filtered and concentrated under reduced pressure. The residue was purified by chromatography on silica (eluent dichloromethane methanol 95:5) to give the product (48 mg, 0.12 mmol, 49 %). **^{1}H NMR** (400 MHz, $CDCl_3$) δ 7.56 (d, *J* = 1.0 Hz, 1H, $NCHN_{imid}$), 6.90 (s, 1H, CH_{imid}), 6.70 (t, *J* = 5.2 Hz, 1H, N*H*Bu), 4.34 (s, 2H, NCH_2N), 3.28 – 3.22 (m, 2H, CH_2), 3.22 – 3.17 (m, 2H, CH_2NH), 3.13 (q, *J* = 7.0 Hz, 2H, CH_2), 3.10 – 3.04 (m, 6H, 3 × CH_2), 1.55 – 1.48 (m, 2H, 2 × CH_2), 1.48 – 1.41 (m, 2H, 2 × CH_2), 1.40 – 1.30 (m, 2H, CH_2), 1.24 (h, 2H, CH_2), 1.10 (t, *J* = 7.0 Hz, 3H, CH_3 CH_2), 1.07 (t, *J* = 7.1 Hz, 3H, CH_3CH_2), 0.91 – 0.86 (m, 6H, 2 × $CH_{3\,Bu}$). **^{13}C NMR** (101 MHz, $CDCl_3$) δ 165.3 (*C*=O), 159.4 (*C*=ONH), 135.3 ($NCHN_{Imid}$), 132.2 (C_{Imid}), 121.7 (CH_{Imid}), 47.5 (CH_2N), 46.0 (CH_2N), 45.6 (CH_2N), 45.6 (CH_2N), 43.5 (NCH_2Imid), 42.9 (CH_2N), 40.9 (CH_2NH_{Bu}), 32.1 (CH_{2Bu}), 30.1 (CH_{2Bu}), 20.3 (CH_{2Bu}), 20.2 (CH_{2Bu}), 14.0 ($CH_{3\,Bu}$), 13.9 ($CH_{3\,Bu}$), 13.6 (CH_3CH_2N), 13.2 (CH_3CH_2N). **FTIR** (neat) $ν_{max}$ = 2960, 1617, 1421, 1260, 1027, 800, 625 cm^{-1}. **HR – MS** (ESI, positive ion mode) – m/z for $[C_{20}H_{38}N_6O_2Na]^+$ 417.2948, found 417.2932.

3.3. Synthesis of compound **3**

Molecules **3a**, **3b**, **3c** were made following reported procedures.[1]

Compound **3e**.

N-(tertbutylcarbamoyl)-*N*-benzyl-*N*'-(*tert*butoxycarbonyl)diethylenetriamine.

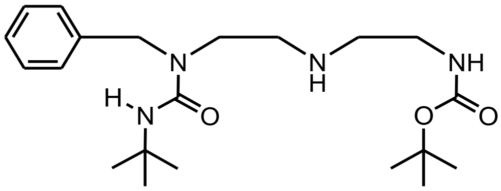

A 0.1 M solution of *N*-(*tert*butylcarbamoyl)-*N*-benzylethylenediamine **3c** (500 mg, 2 mmol) in anhydrous MeOH and *N*-Boc-2-aminoacetaldehyde (477 mg, 3 mmol, 1.5 equiv.) was stirred at 20 °C for 12 hours. Sodium borohydride $NaBH_4$ (3 equiv.) was then added portionwise (1 equiv. every 30 minutes) at 0 °C and the resulting mixture was stirred for 5 hours at 20 °C. Water (20 mL) was added and the mixture was stirred for 30 minutes at 20 °C, then was concentrated under reduced pressure to remove MeOH. Brine was added to the resulting aqueous phase and was extracted with $CHCl_3$: *i*PrOH 2:1 mixture, the organic layer was dried over Na_2SO_4, filtered and concentrated under reduced pressure. Flash chromatography of the residue on silica gel gave the title compound **3e** as colourless oil (480 mg, 1.24 mmol, 62 %). The product tends to degrade on silica, no chromatography purification is preferred for that step. **^{1}H NMR** (400 MHz, $CDCl_3$) δ 7.25 (m, 2H, 2 × CH_{Ar}), 7.22 – 7.15 (m, 3H, 3 × CH_{Ar}), 5.66 (s, 1H, N*H*), 5.02 (m, 1H, N*H*), 4.38 (s, 2H, CH_2Ph), 3.24 (t, *J* = 5.6 Hz, 2H, CH_2), 3.11 (q, *J* = 5.6 Hz, 2H, CH_2), 2.61 (m, 4H, 2 × CH_2), 1.37 (s, 9H, 3 × CH_3C_{Boc}), 1.25 (s, 9H, 3 × CH_3C_{urea}). **^{13}C NMR** (101 MHz, $CDCl_3$) δ 158.6 ($C{=}O_{Boc}$), 156.1 ($C{=}O_{urea}$), 138.7 (C_{Ar}), 128.6 (2C, 2 × CH_{Ar}), 127.3 (2C, 2 × CH_{Ar}), 127.2 (CH_{Ar}), 79.1 ($C(CH_3)_{3Boc}$), 59.3 ($C(CH_3)_{3urea}$), 50.7 (CH_2), 50.5 (CH_2), 50.3 (CH_2), 49.2 (CH_2), 48.4 (CH_2), 48.4 (CH_2), 40.1 (CH_2), 29.4 (3C, 3 × CH_{3Boc}), 28.4 (3C, 3 × CH_{3tBu}). **FTIR (neat)** ν_{max} = 3357, 2975, 2931, 1696, 1524, 1392, 1366, 1252, 1166 cm^{-1}. **HR – MS** (ESI, positive ion mode) – *m/z* for $[C_{21}H_{36}N_4O_3+H]^+$ 393.2860, observed 393.2863.

Compound 3f.

N-(tertbutylcarbamoyl)-N-benzyl-N'-(tertbutoxycarbonyl)-N''-(n-hexylcarbamoyl)diethylenetriamine.

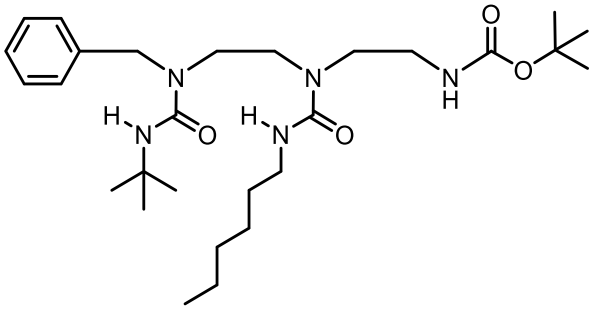

A 0.1M solution of N-(tertbutylcarbamoyl)-N-benzyl-N'-(tertbutoxycarbonyl)diethylenetriamine **3e** (230 mg, 0.59 mmol) in CH_2Cl_2 and n-hexyl isocyanate (0.13 mL, 0.885 mmol, 1.5 equiv) was stirred at 20 °C for 12 hours. Water (10 mL) was then added, the resulting mixture was extracted using CH_2Cl_2. The organic layer was dried over Na_2SO_4, filtered and concentrated under reduced pressure. The crude product was purified by silica gel flash chromatography (CH_2Cl_2:MeOH 95:5) to afford the title compound as a colourless oil (239 mg, 0.466 mmol, 79 %). **^{1}H NMR** (400 MHz, $CDCl_3$) δ 7.31 (t, *J* = 7.4 Hz, 2H, 2 × CH_{Bn}), 7.24 (t, *J* = 7.1 Hz, 1H, CH_{Bn}), 7.21 – 7.14 (m, 2H, 2 × CH_{Bn}), 6.37 (s, 1H, N*H*), 5.41 (t, *J* = 5.6 Hz, 1H, N*H*), 4.83 (s, 1H, N*H*), 4.39 (s, 2H, CH_2Ph), 3.34 (dd, *J* = 9.1, 5.4 Hz, 2H, CH_2N), 3.29 – 3.15 (m, 6H, 3 × CH_2N), 3.11 (dd, *J* = 6.4 Hz, 2H, CH_2N), 1.53 (m, 2H, $CH_{2\ hexyl}$), 1.39 (s, 9H, $(CH_3)_3C$), 1.36 – 1.26 (m, 6H, 3 × $CH_{2\ Hexyl}$), 1.25 (s, 9H, $(CH_3)_3C$), 0.91

– 0.78 (m, 3H, C$H_{3\ hexyl}$). **^{13}C NMR** (101 MHz, $CDCl_3$,) δ 158.7 (C=O), 157.8 (C=O), 156.7 (C=O), 137.9 (C_{Bn}), 128.8 (2C, 2 × CH_{Ar}), 127.6 (CH_{Ar}), 126.8 (2C, 2 × CH_{Ar}), 79.4 ($C(CH_3)_3O$), 51.8 (CH_2Ph), 50.9 ($C(CH_3)_3N$), 47.5 (CH_2), 47.1 (CH_2), 46.7 (CH_2), 41.1 (CH_2), 40.0 (CH_2), 31.6 (CH_2), 29.7 (CH_2), 29.3 (3C, $(CH_3)_3CO$), 28.3 (3C, $(CH_3)_3CN$), 26.7 (CH_2), 22.6 (CH_2), 14.0 (CH_3). **FTIR (neat)** ν_{max} = 3288, 2959, 2927, 2866, 1683, 1628, 1545, 1354, 1282 cm^{-1}. **HR – MS** (ESI, positive ion mode) – *m/z* for $[C_{28}H_{49}N_5O_4+H]^+$ 520.3857, observed 520.3843.

Compound **3g**.

N-(tert-Butylcarbamoyl)-N-benzyl-N''-(n-hexylcarbamoyl)diethylenetriamine

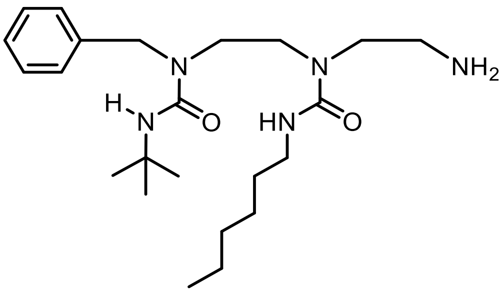

A 0.05 M solution of *N*-(*tert*butylcarbamoyl)-*N*-benzyl-*N*'-(*tert*butoxycarbonyl)-*N*''-(n-hexylcarbamoyl)diethylenetriamine **3f** (410 mg, 0.8 mmol) in CH_2Cl_2 and trifluoroacetic acid (18 equiv.) was stirred for 12 hours at 20 °C. Water then CH_2Cl_2 were added, the organic layer was washed with aqueous $NaHCO_3$, dried over Na_2SO_4, filtered and concentrated under reduced pressure. The residue was purified by flash chromatography on silica gel (eluent: CH_2Cl_2:MeOH 95:5) to give the unprotected amine as a colourless oil (325 mg, 0.776 mmol, 97 %). **^{1}H NMR** (400 MHz, $CDCl_3$) δ 7.28 – 7.19 (m, 3H, 3 × CH_{Ar}), 7.15 – 7.09 (m, 2H, 2 × CH_{Ar}), 6.90 (t, *J* = 5.2 Hz, 1H, NH_{Hexyl}), 4.33 (s, 2H, CH_2Ph), 4.60 (s, 1H, N*Ht*Bu), 3.42 (t, *J* = 6.1 Hz, 2H, CH_2N), 3.31 (m, 2H, CH_2), 3.24 (m, 2H, CH_2), 3.08 (td, *J* = 7.1, 4.9 Hz, 2H, CH_2NH), 2.96 (t, *J* = 6.1 Hz, 2H, CH_2NH_2), 1.46 (q, *J* = 7.2 Hz, 2H, CH_2), 1.31 – 1.09 (m, 6H, 3 × CH_2), 1.16 (s, 9H, 3 × CH_3C_{tBu}), 0.83 – 0.71 (m, 3H, CH_{3hexyl}). **^{13}C NMR** (101 MHz, $CDCl_3$) δ 159.2 (C=O $_{hexyl\ urea}$), 157.8 (C=O $_{tBu\ urea}$), 137.5 (C_{Ar}), 128.9 (2C, CH_{Ar}), 127.6 (CH_{Ar}), 126.6 (2C, CH_{Ar}), 52.0 ($C(CH_3)_3$), 50.9 (CH_2Ph), 47.3 (2C, 2 × CH_2), 46.7 (CH_2), 41.2 (CH_2NH), 39.8 (CH_2NH_2), 31.6 (CH_2), 29.6 (CH_2), 29.2 (3C, 3 $(CH_3)C$), 26.7 (CH_2), 22.5 (CH_2), 14.0 ($CH_{3\ Hexyl}$). **FTIR (neat)** ν_{max} = 3308, 2956, 2926, 1627, 1535, 1452, 1265, 1211 cm^{-1}. **HR – MS** (ESI, positive ion mode) – *m/z* for $[C_{23}H_{41}N_5O_2+H]^+$ 420.3333, observed 420.3323.

Compound **3**.

N-(*tert*Butylcarbamoyl)-*N*-benzyl-*N*''-(n-hexylcarbamoyl)-*N'*-(3,5-*bis*(trifluoromethyl)phenylcarbamoyl)diethylenetriamine

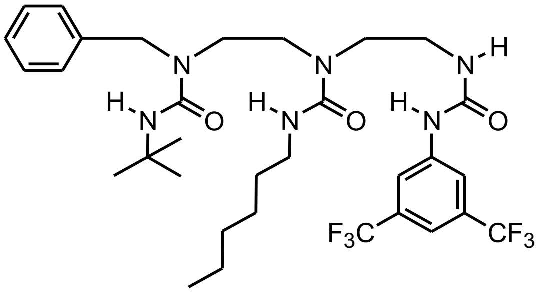

To a 0.05 M solution of *N*-(tertbutylcarbamoyl)-*N*-benzyl-*N*''-(n-hexylcarbamoyl)diethylenetriamine **3g** (25 mg, 0.06 mmol) in CH_2Cl_2 was added dropwise at 20 °C 3,5-*bis*(trifluoromethyl)phenyl isocyanate ((11 µL, 0.06 mmol, 1.2 equiv.),

the mixture was stirred for 4 hours. Addition of water was followed with extraction of the aqueous layer using CH_2Cl_2. The organic layer was dried over Na_2SO_4, filtered and concentrated under reduced pressure. The residue was purified by flash chromatography (eluent: CH_2Cl_2 : MeOH 95:5) on silica to give the title urea **3** as a colourless oil (21 mg, 0.031 mmol, 54 %). **^{1}H NMR** (400 MHz, CD_2Cl_2) δ 8.63 (bs, 1H, N*H*Ar), 7.93 (s, 2H, 2 × CH_{Ar}), 7.33 (s, 1H, CH_{Ar}), 7.29 (m, 2H, 2 × CH_{Ar}), 7.21 (t, 1H, CH_{Ar}), 7.13 (d, *J* = 8.8 Hz, 2H, 2 × CHAr), 6.64 (t, 1H, NH_{hexyl}), 6.37 (t, 1H, N*H*CONHAr), 4.34 (s, 1H, N*Ht*Bu), 4.32 (s, 2H, C*H*$_2$Ph), 3.37 (m, 2H, C*H*$_2$NBn), 3.34 – 3.27 (m, 4H, 2 × C*H*$_2$N), 3.26 (m, 2H, CH_2NH_{ethyl}), 3.13 (dd, J = 6.6 Hz, 2H, CH_2NH_{hexyl}), 1.46 (m, 2H, C*H*$_2$), 1.29 – 1.17 (m, 6H, 3 × C*H*$_2$), 1.16 (s, 9H, $(CH_3)_3C$), 0.82 (t, 3H, C*H*$_3$). **^{13}C NMR** (101 MHz, $CDCl_3$) δ 159.3 ($C(O)NH_{Hexyl}$), 158.2 (*C*(O)NH*t*Bu), 156.1 (NH*C*(O)NH), 141.8 (C_{Ar}), 136.9 (C_{Ar}), 132.0 (q, *J* = 33.1 Hz, 2C, 2 × $C_{Ar}CF_3$), 129.3 (2C, CH_{Ar}), 128.1 (CH_{Ar}), 126.4 (2C, 2 × CH_{Ar}), 123.4 (q, *J* = 272.7 Hz, 2C, 2 × CF_3), 118.0 (CH_{Ar}), 114.9 (CH_{Ar}), 52.9 (CH_2Ph), 51.3 (*Ct*Bu), 48.4 (CH_2NHBn), 48.4 (CH_2), 47.0 (CH_2), 41.7 ($NHCH_{2\ Hexyl}$), 39.2 (CH_2NHCONHAr), 31.7 (CH_2CH_3), 29.6 (CH_2), 29.3 (3C, $(CH_3)_3C$), 26.9 (CH_2), 22.6 (CH_2), 14.1 (CH_3). **FTIR (neat)** ν_{max} = 2960, 2935, 2874, 1631, 1472, 1277 cm^{-1}. **HR – MS** (ESI, positive ion mode) – *m/z* for $[C_{32}H_{44}F_6N_6O_3+H]^+$ 675.3452, observed 675.3458.

3.4. Synthesis of control compounds S15-S17

Compound SI5

N,N'-diethyl-*N,N'*-bis(n-butylcarbamoyl)ethylenediamine

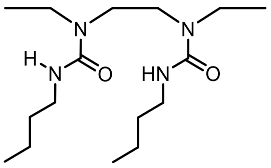

To a 0.04 M solution of *N,N'*-di(ethyl)ethylenediamine (100 µL, 0.69 mmol) in 1,2-dichloroethane was added n-butyl isocyanate (195 µL, 1.72 mmol, 2.5 equiv.), the mixture was stirred at 20 °C for 16 hours. Water was added, the resulting mixture was extracted with CH_2Cl_2, the organic layer was dried over $MgSO_4$, filtered and concentrated under reduced pressure. The residue was purified using silica gel flash chromatography (eluent CH_2Cl_2:MeOH 97:3) to provide the title compound as a white solid (134 mg, 0.44 mmol, 64 %). **^{1}H NMR** (400 MHz, $CDCl_3$) δ 5.19 (s, 2H, 2 × NH), 3.28 (s, 4H), 3.27 – 3.18 (m, 8H), 1.56 – 1.44 (m, 4H), 1.43 – 1.29 (m, 4H), 1.13 (t, *J* = 7.1 Hz, 6H), 0.92 (t, *J* = 7.3 Hz, 6H). **^{1}H NMR** (400 MHz, CD_2Cl_2) δ 5.43 (s, 2H, 2 × NH), 3.15 (m, 8H, 2 × CH_2N, 2 × $CH_{2\ Et}$), 3.13 – 3.06 (m, 4H, 2 × CH_{2Bu}), 1.48 – 1.33 (m, 4H), 1.27 (dt, *J* = 7.2 Hz, 4H, 2 × C*H*$_2$), 1.01 (t, *J* = 7.1 Hz, 6H, 2 × CH_{3cEt}), 0.84 (t, *J* = 7.3 Hz, 6H, 2 × CH_{3Bu}). **^{13}C NMR** (101 MHz, CD_2Cl_2) δ 158.5 (C=O), 47.0 (2C, 2 × CH_2), 43.3 (2C, 2 × CH_2), 41.1 (2C, 2 × CH_2), 33.0 (2C, 2 × CH_2), 20.7 (2C, 2 × CH_2), 14.5 (CH_3), 14.2 (CH_3). **FTIR (neat)** ν_{max} = 3344, 2954, 2927, 2872, 1623, 1533, 1406, 1273 cm^{-1}. **HR – MS** (ESI, positive ion mode) – *m/z* for $[C_{18}H_{36}N_2O_2+H]^+$ 315.2754, observed 315.2765. **MP** 156-158 °C.

Compound SI6

N,N'-*Bis*(benzyl)-*N,N',N''*-tri(n-butylcarbamoyl)diethylenetriamine,

Compound **S16** was made following reported procedures.[3]

Compound **SI7**

1-n-Butyl-3-(4-(trifluoromethyl)phenyl)urea

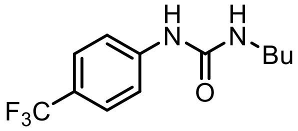

To a solution of n-butylamine (70 µL, 0.7 mmol) in anhydrous dichloromethane was added 4-trifluoromethylphenyl isocyanate (100 µL, 0.7 mmol) and the mixture was stirred for 12 hours at 20 °C. Concentration of the mixture under reduced pressure afforded the product as a white solid (182 mg, 0.7 mmol, quantitative). **^{1}H NMR** (400 MHz, CD_2Cl_2) δ 7.54 – 7.36 (m, 4H, 4 × CH_{Ar}), 7.03 – 6.86 (s, 1H, NH_{Ar}), 5.11 – 4.92 (s, 1H, N*H*), 3.26 – 3.1 (td, *J* = 7.2, 5.7 Hz, 2H, CH_2NH), 1.52 – 1.38 (m, 2H, C$H_{2\ butyl}$), 1.38 – 1.23 (m, 2H, C$H_{2\ butyl}$), 0.96 – 0.79 (t, *J* = 7.3 Hz, 3H, C$H_{3\ butyl}$). **^{1}H NMR** (400 MHz, $CDCl_3$) δ 7.50 (d, *J* = 8.6 Hz, 2H, 2 × CH_{Ar}), 7.42 (d, *J* = 8.5 Hz, 2H, 2 × CH_{Ar}), 6.90 (s, 1H, N*H*), 5.01 (t, *J* = 5.6 Hz, 1H, N*H*), 3.25 (td, *J* = 7.1, 5.6 Hz, 2H, CH_2NH), 1.49 (tt, *J* = 7.9, 6.4 Hz, 2H, CH_2), 1.41 – 1.28 (m, 2H, CH_2), 0.91 (t, *J* = 7.3 Hz, 3H, CH_3). **^{13}C NMR** (101 MHz, CD_2Cl_2) δ 155.9 (*C*=O), 143.3 (C_{Ar}), 131.3 (C_{Ar}), 127.0 – 126.7 (q, *J* = 3.8 Hz, 2C, 2 × CH_{Ar}), 125.6 – 123.8 (q, *J* = 32.5 Hz, 2C, CH_{Ar}), 120.9-129.1 (q, *J* = 269.1 Hz, CF_3), 40.6 (CH_2NH), 32.7 ((CH_2), 20.6 (CH_2), 14.1 (CH_3). **FTIR (neat)** ν_{max} = 3339, 2961, 1637, 1553, 1327, 1119 cm^{-1}. **HR – MS** (ESI, positive ion mode) – *m/z* for $[C_{12}H_{15}F_3N_2O+Na]^+$ 283.1029, observed 283.1015 **MP** 135-136 °C.

III. Supplementary Figures

4. NMR spectra of synthesised compounds

Supplementary Figure 4. ^{1}H NMR (400 MHz) and ^{13}C NMR (100 MHz) spectra of *N*,*N*'-Bis(trifluoroacetyl)-*N*'',*N*'''-bis(n-butylcarbamoyl)triethylenetetramine, **1b** (in DMSO d_6)

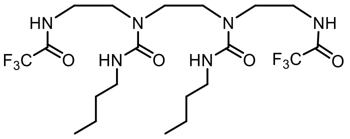

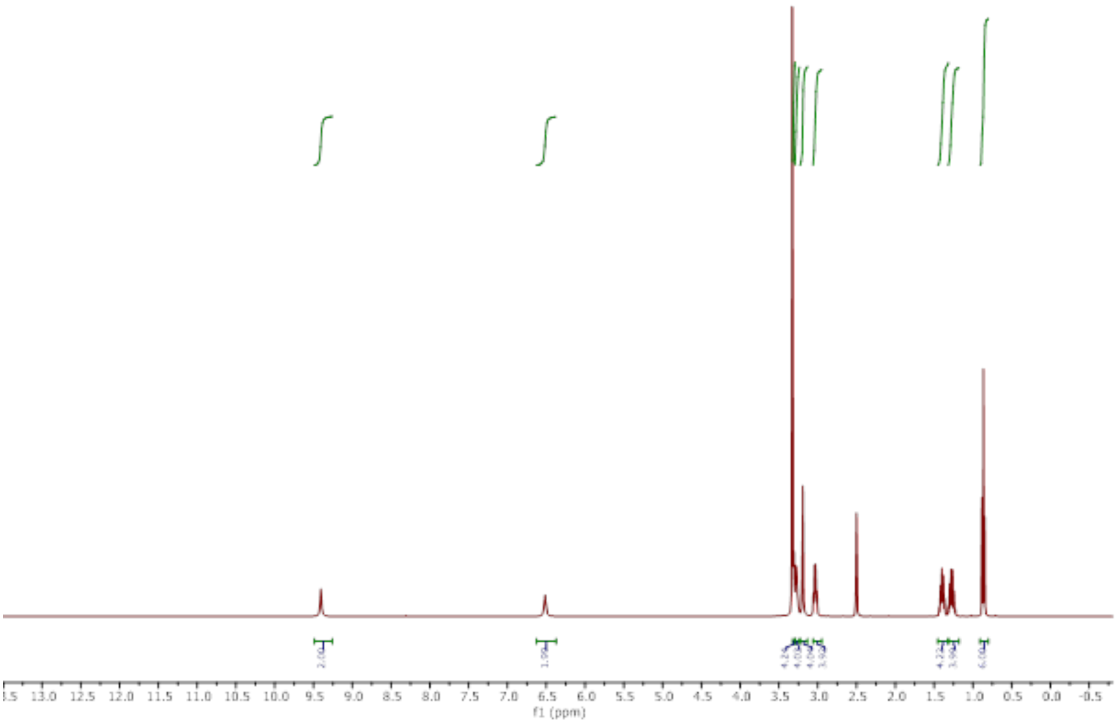

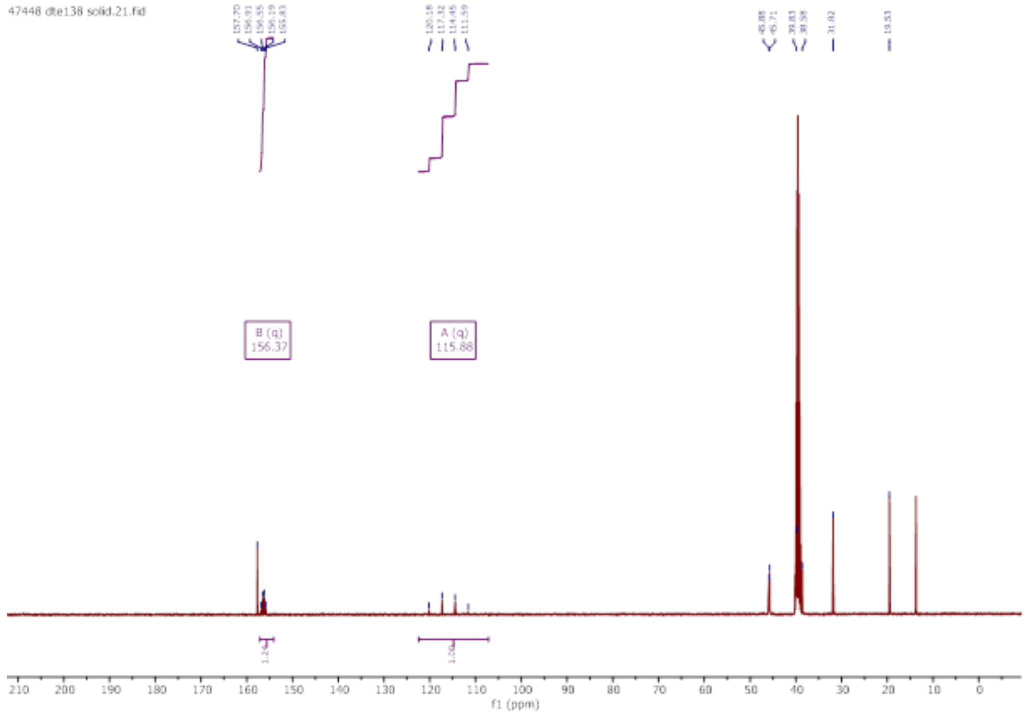

Supplementary Figure 5. ^{1}H NMR (400 MHz) and ^{13}C NMR (100 MHz) spectra of *N''*,*N'''*-Di(n-butylcarbamoyl)triethylenetetramine, **1c** (in $CDCl_3$)

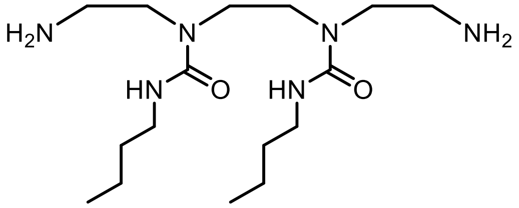

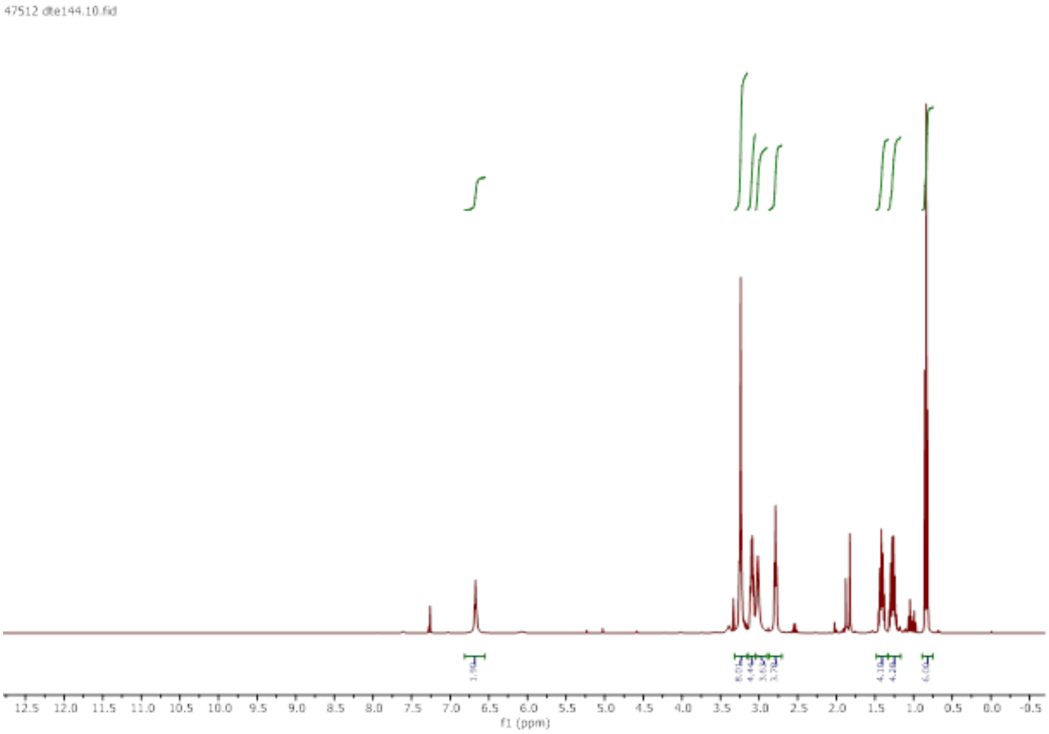

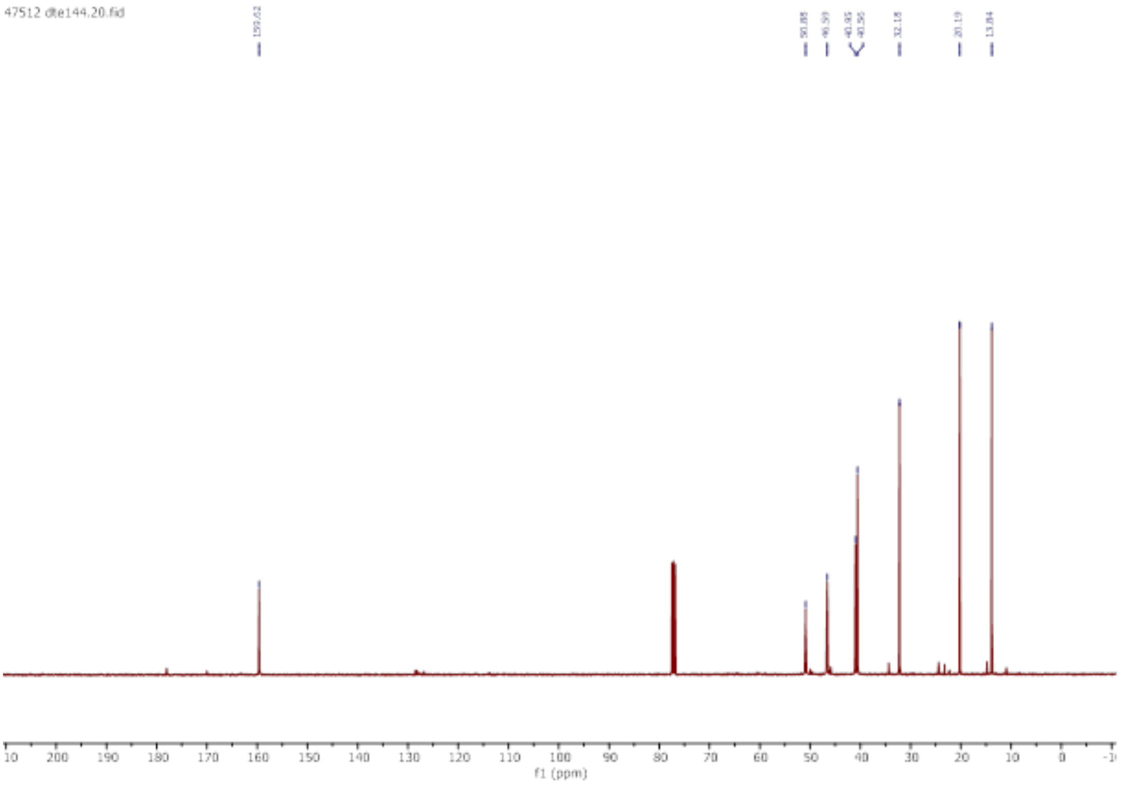

Supplementary Figure 6. ^{1}H NMR (400 MHz) and ^{13}C NMR (100 MHz) spectra of *N,N'*-Bis(3,5-*bis*(trifluoromethyl)phenylcarbamoyl)-*N'',N'''*-*bis*(n-butylcarbamoyl)triethylenetetramine, 1da (in DMSO d_6)

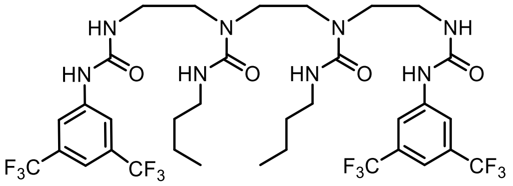

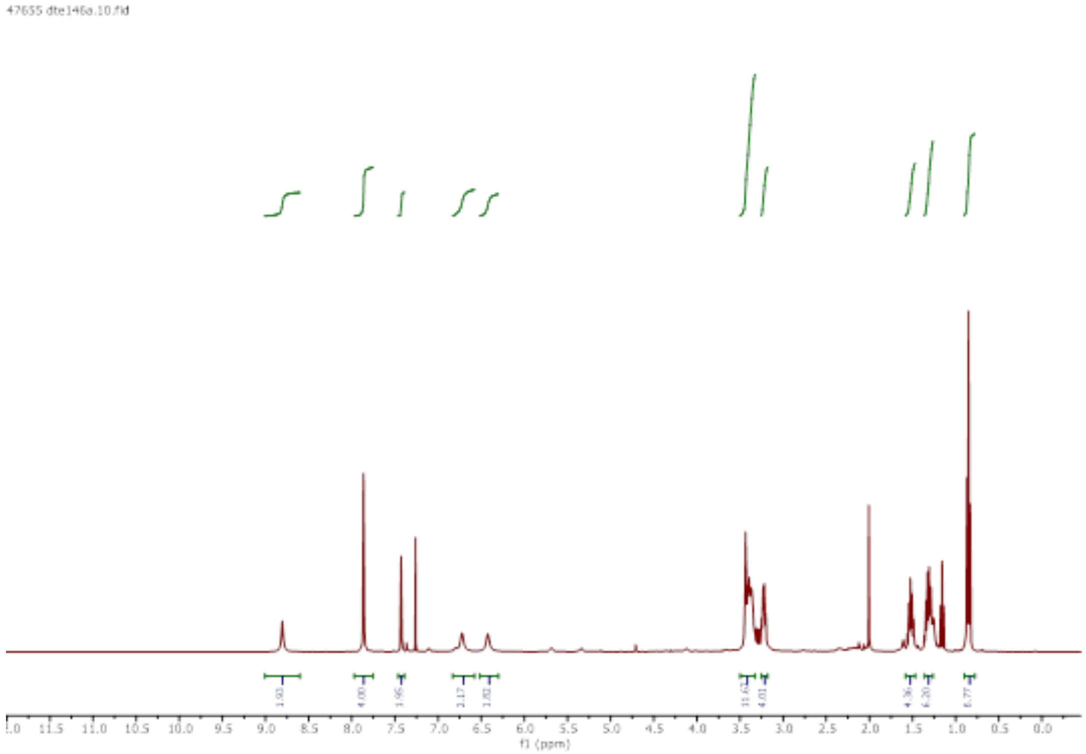

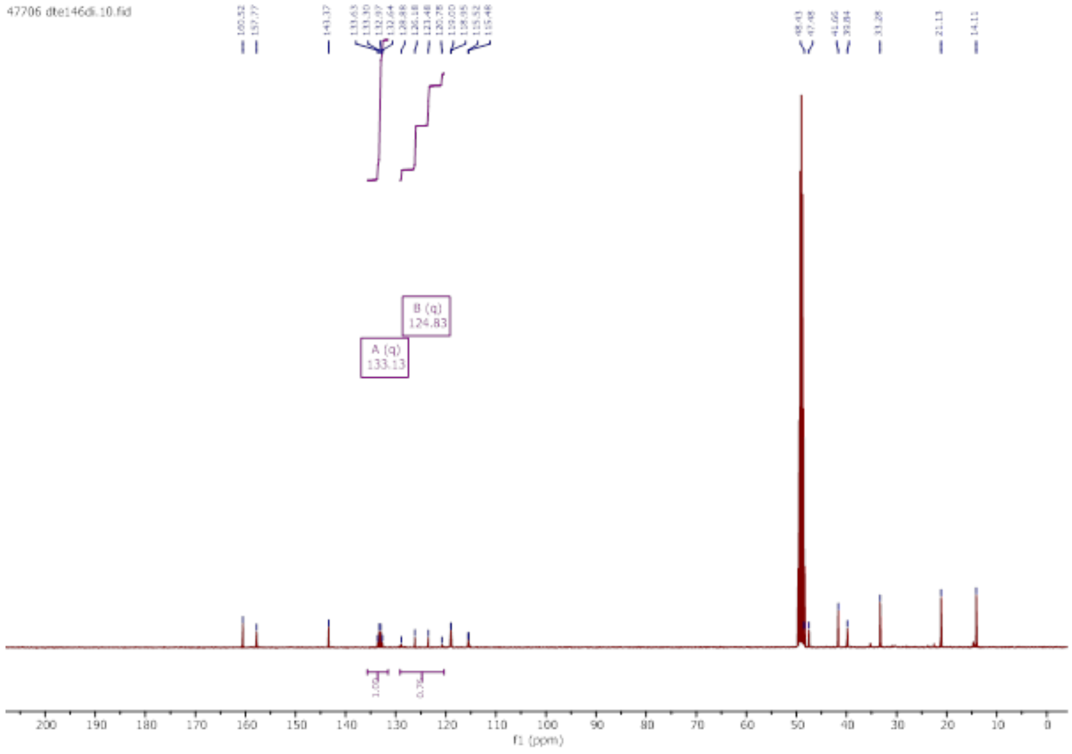

Supplementary Figure 7. ^{1}H NMR (400 MHz) and ^{13}C NMR (100 MHz) spectra of *N*-(3,5-Bis(trifluoromethyl)phenylcarbamoyl)- *N''*,*N'''*-di(n-butylcarbamoyl)triethylenetetramine, **1d** (in $CDCl_3$)

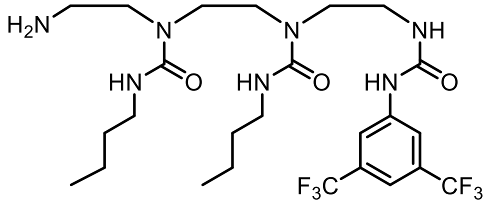

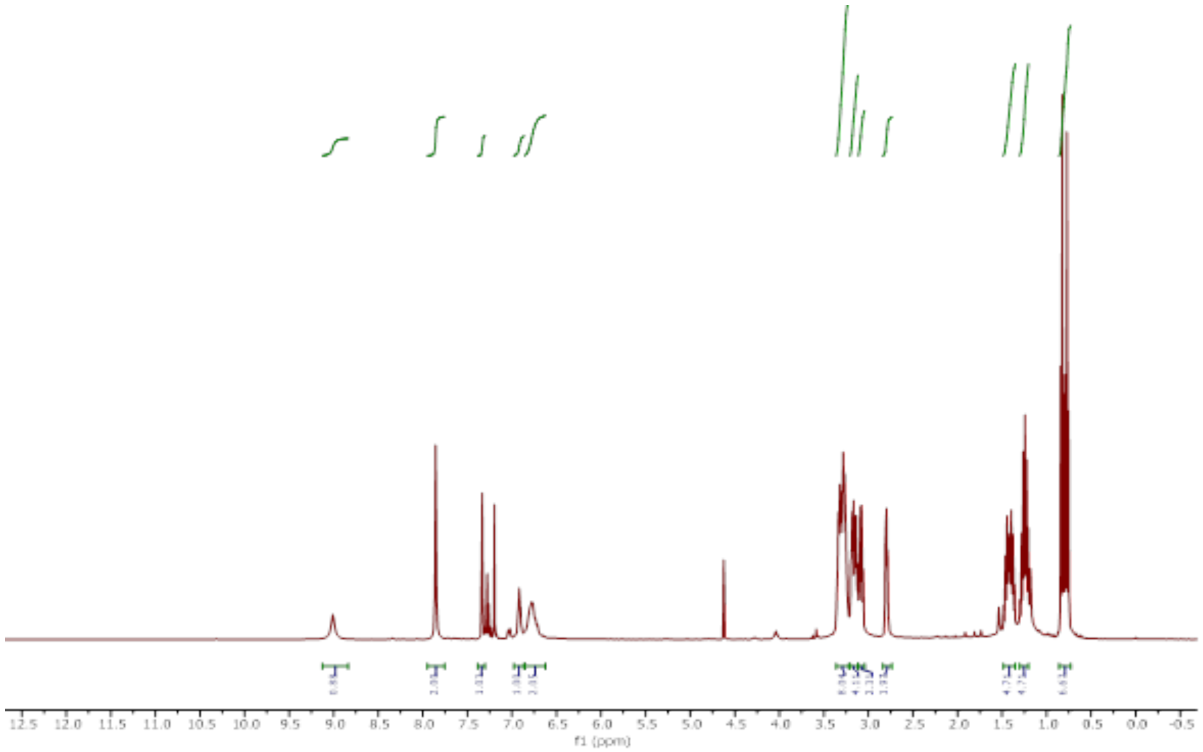

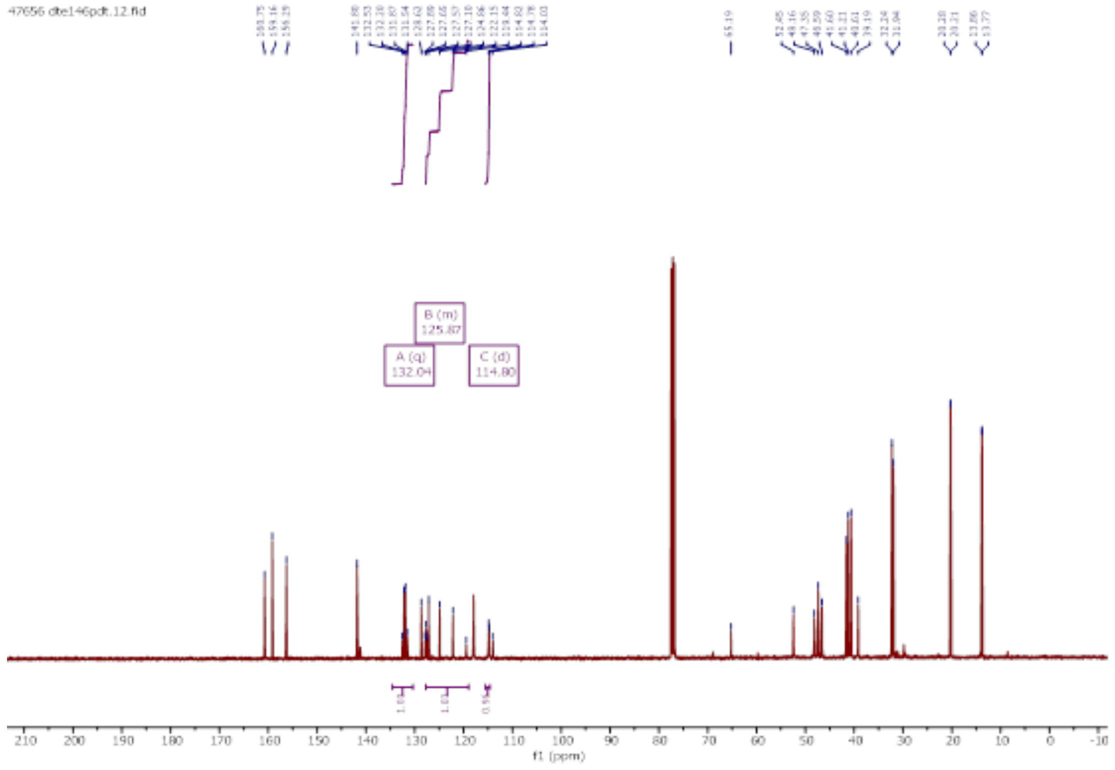

Supplementary Figure 8. ^{1}H NMR (400 MHz), COSY, ^{13}C NMR (100 MHz), HSQC, HMBC spectra of 1-(2-(3-(3,5-Bis(trifluoromethyl)phenyl)ureido)ethyl)-3-n-butyl-1-(2-(3-butyl-1-(2-(((1-((2-(trimethylsilyl)ethoxy)methyl)-1H-imidazol-4-yl)methyl)amino)ethyl)ureido)ethyl)urea, **1eb** (in $CDCl_3$)

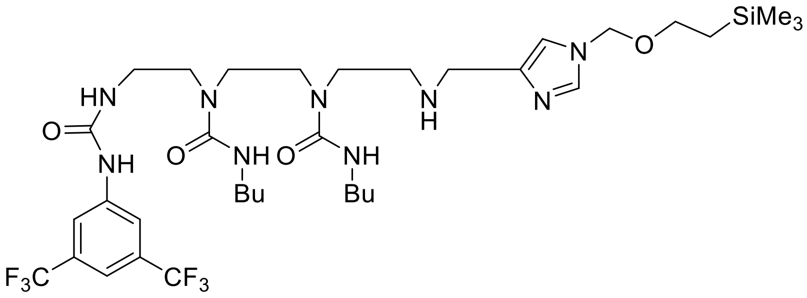

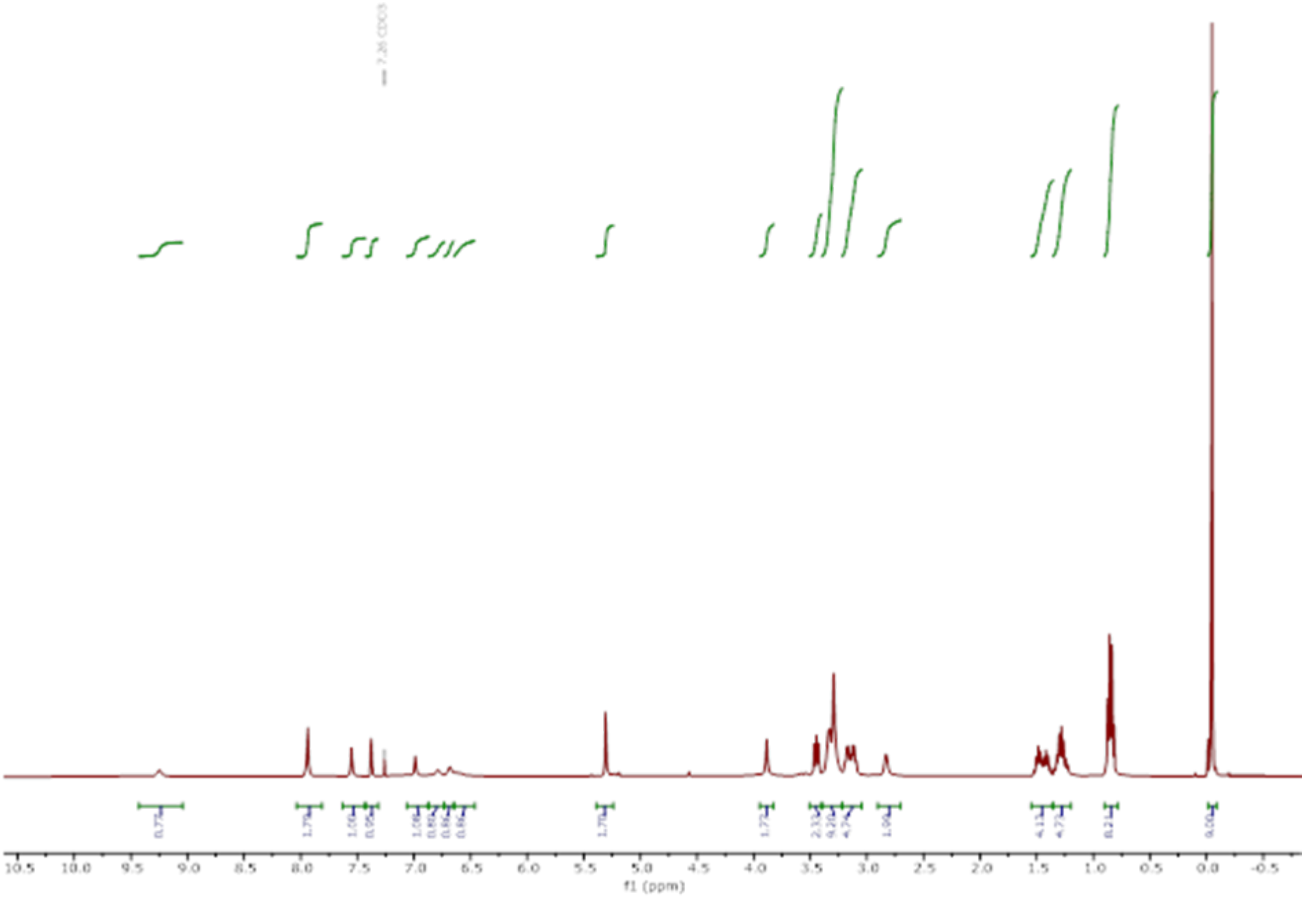

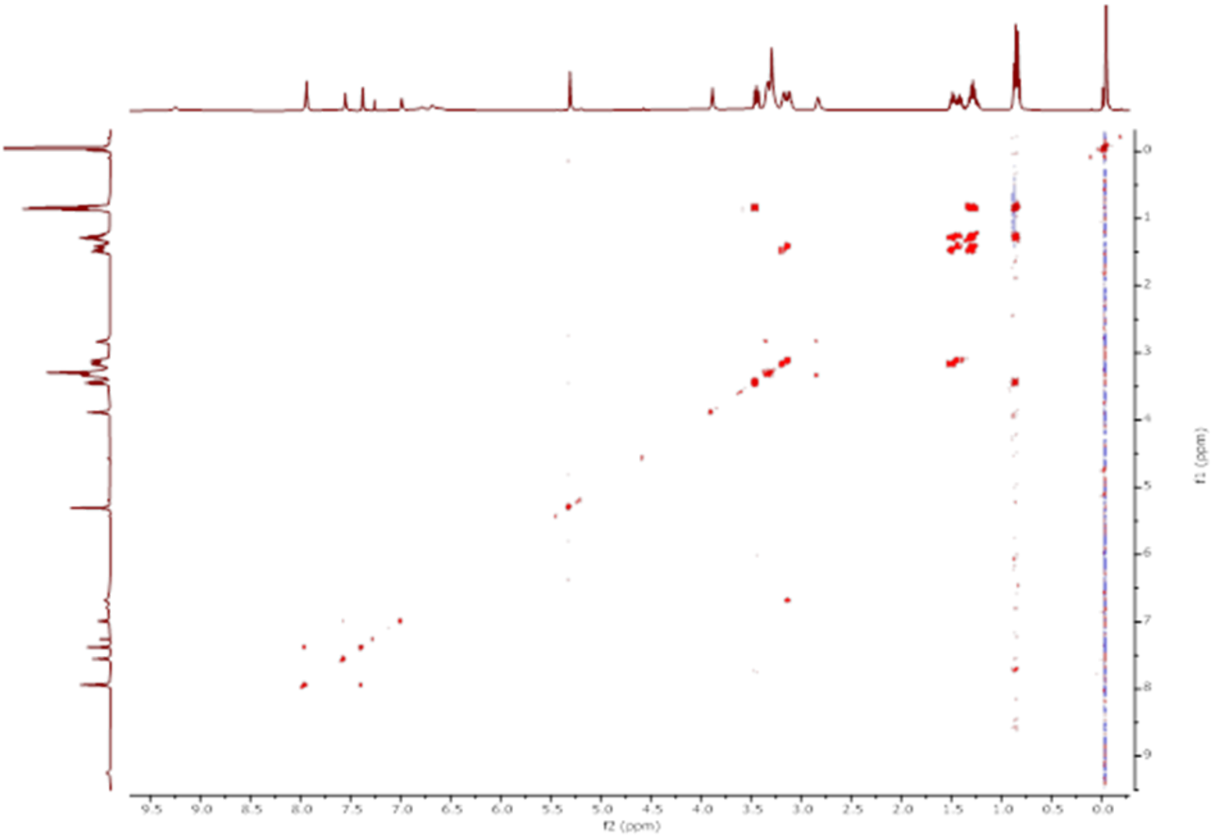
f2 (ppm)
f1 (ppm)

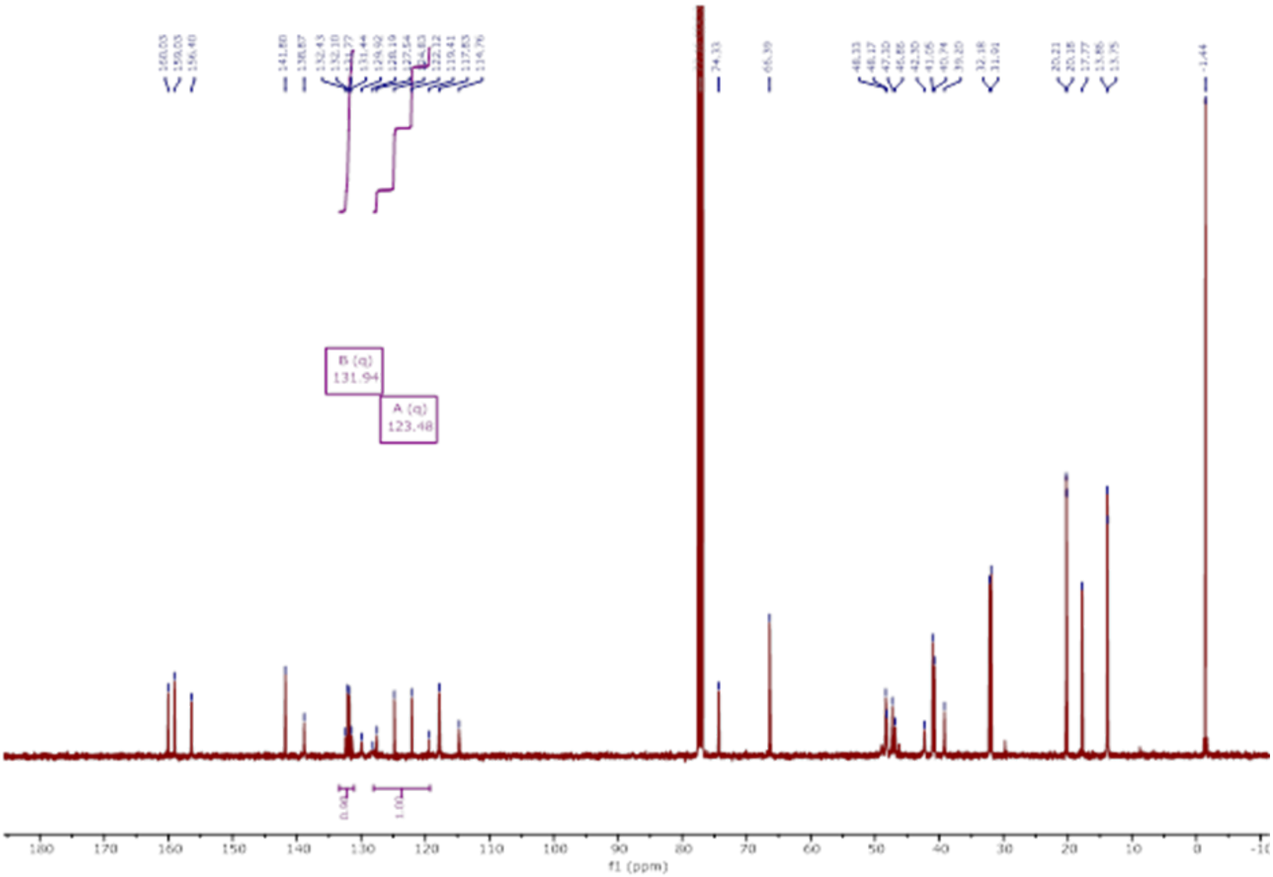
B (q)
131.94
A (q)
123.48
f1 (ppm)

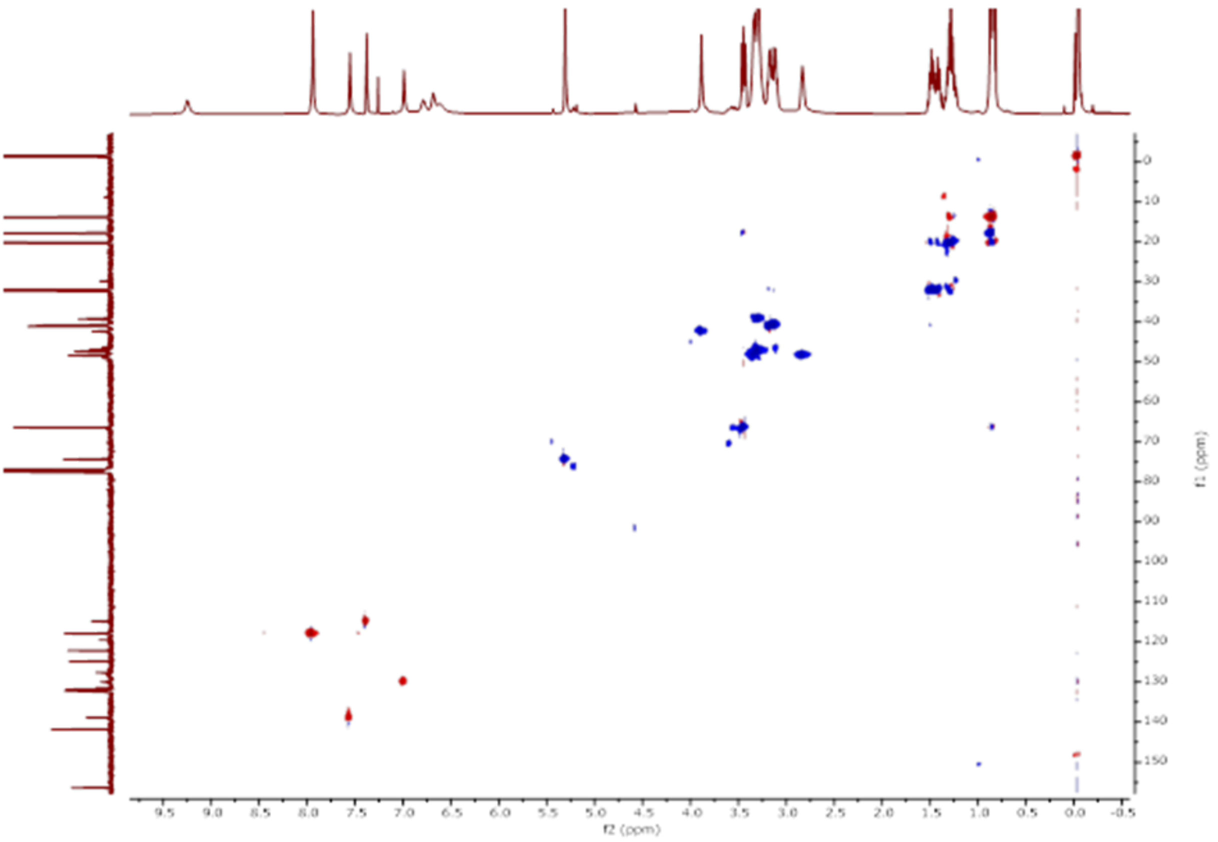
f1 (ppm)
f2 (ppm)

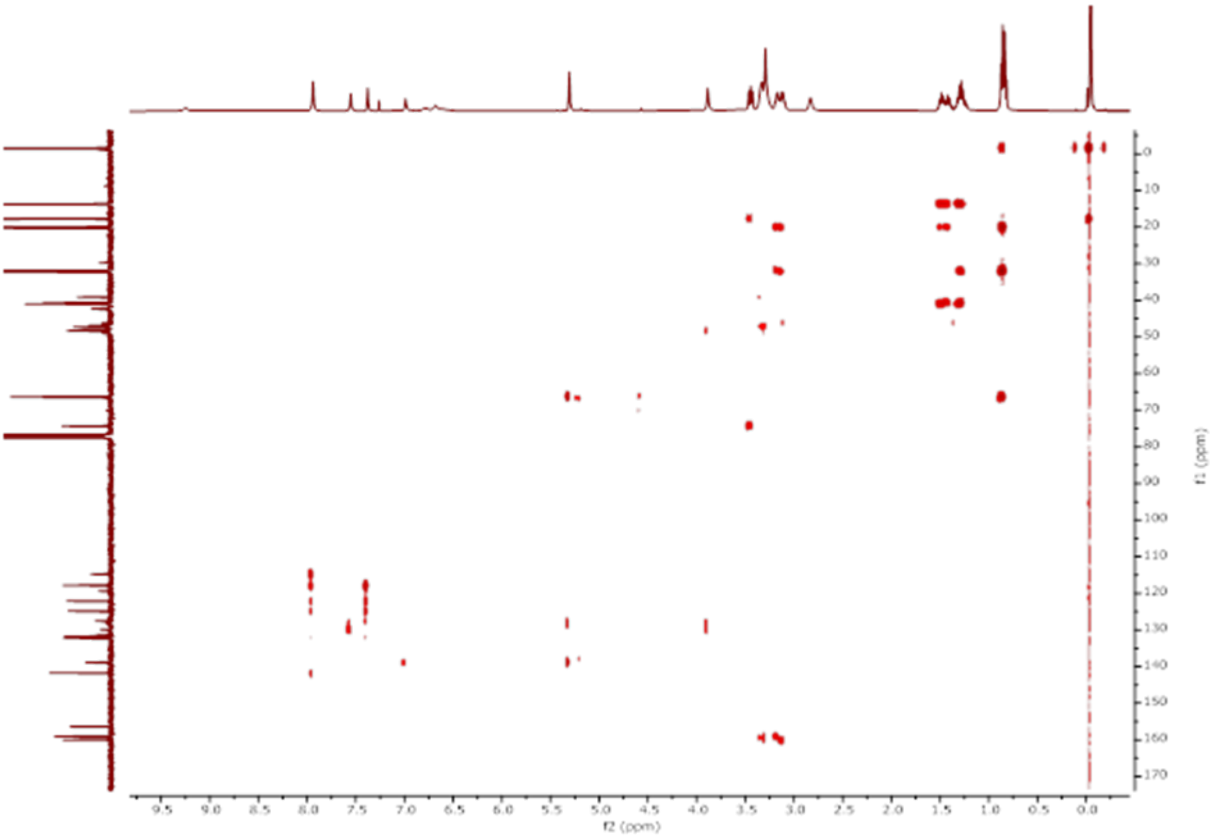
f1 (ppm)
f2 (ppm)

Supplementary Figure 9. ^{1}H NMR (400 MHz), COSY, ^{13}C NMR (100 MHz), HSQC spectra of 1-(2-(3-(3,5-Bis(trifluoromethyl)phenyl)ureido)ethyl)-3-n-butyl-1-(2-(3-butyl-1-(2-(((1-((2-(trimethylsilyl)ethoxy)methyl)-1H-imidazol-5-yl)methyl)amino)ethyl)ureido)ethyl)urea, 1ea (in $CDCl_3$)

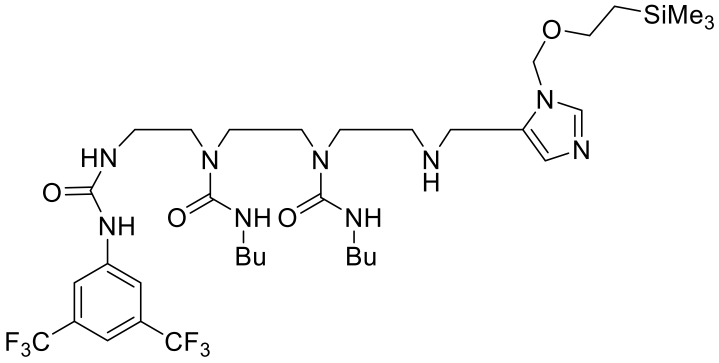

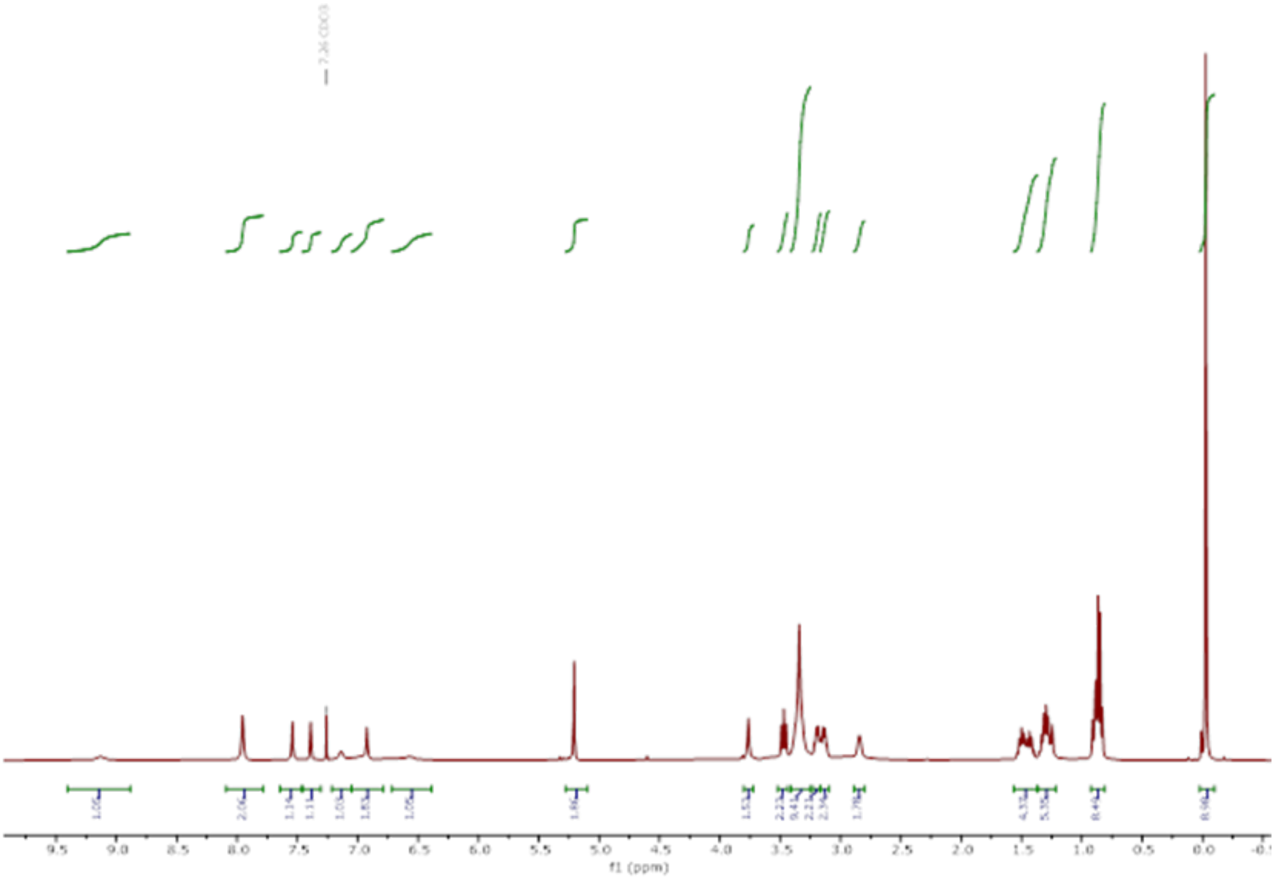

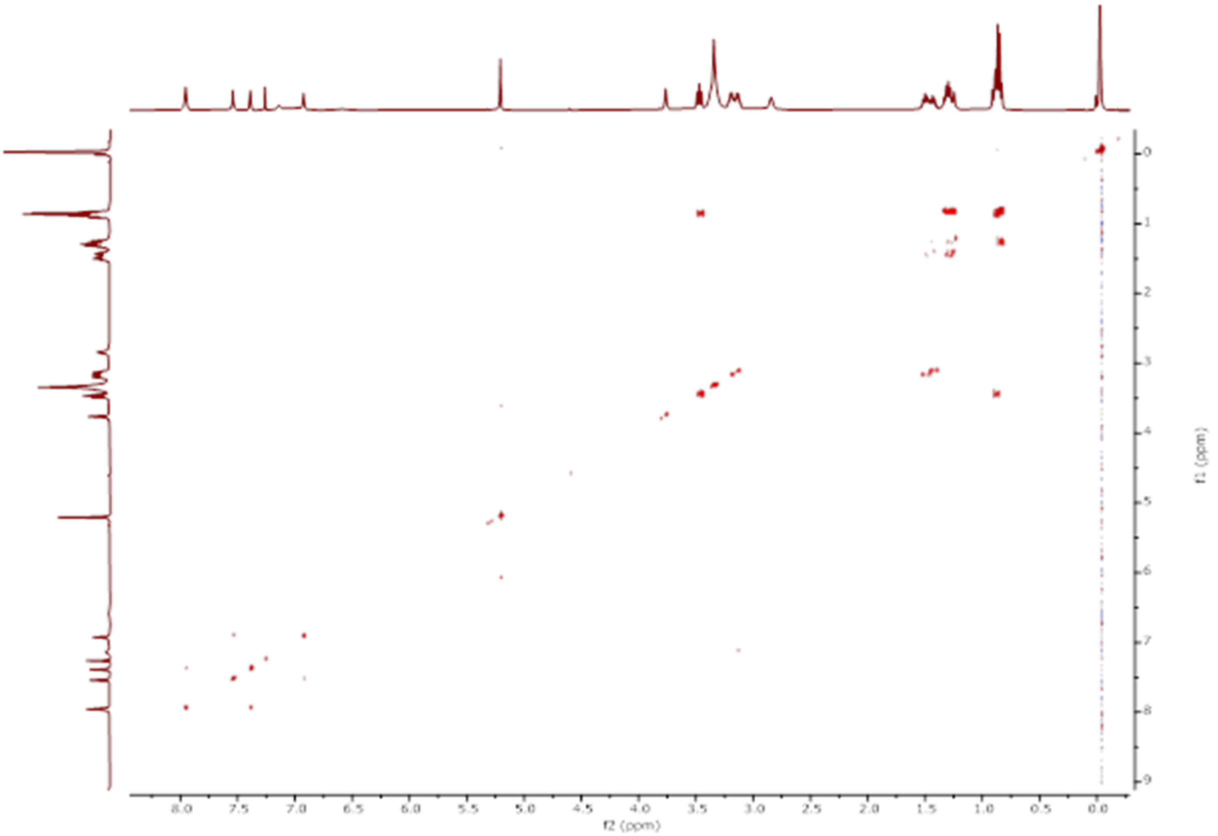
f2 (ppm)
f1 (ppm)

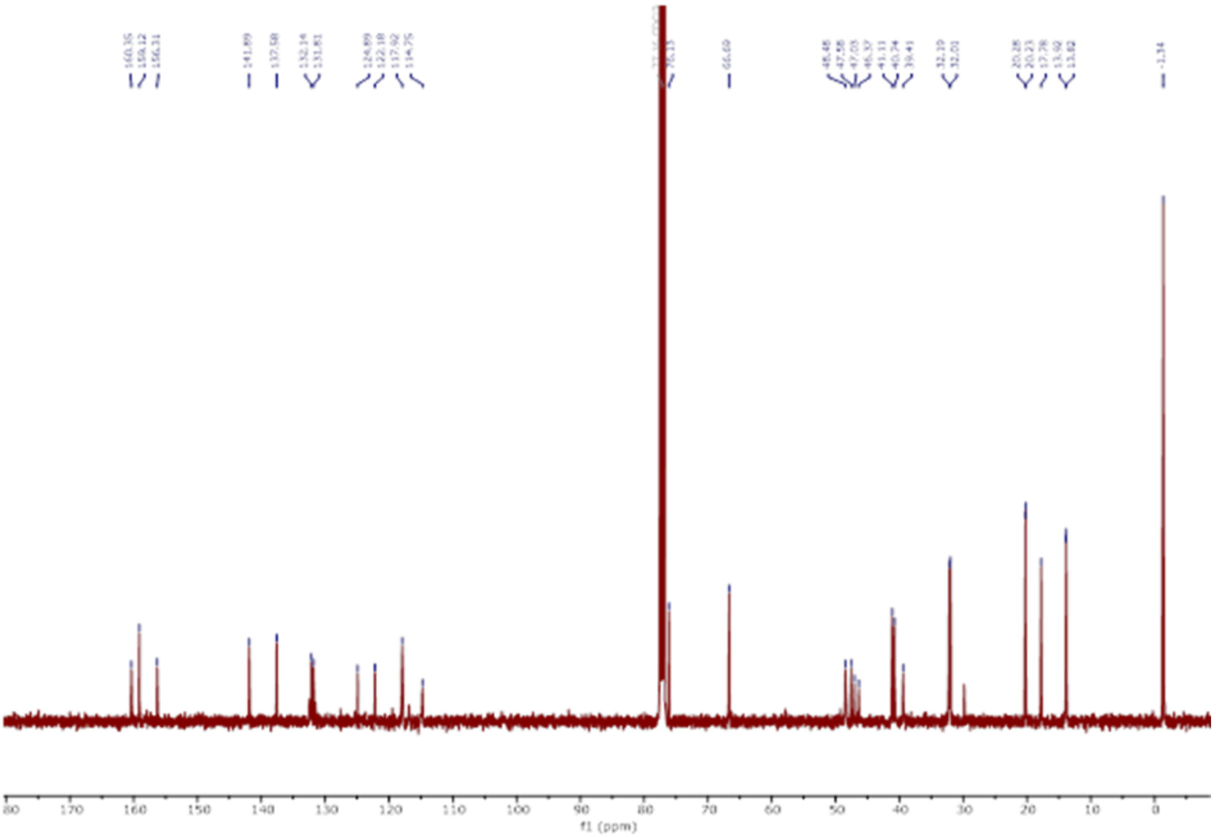
f1 (ppm)

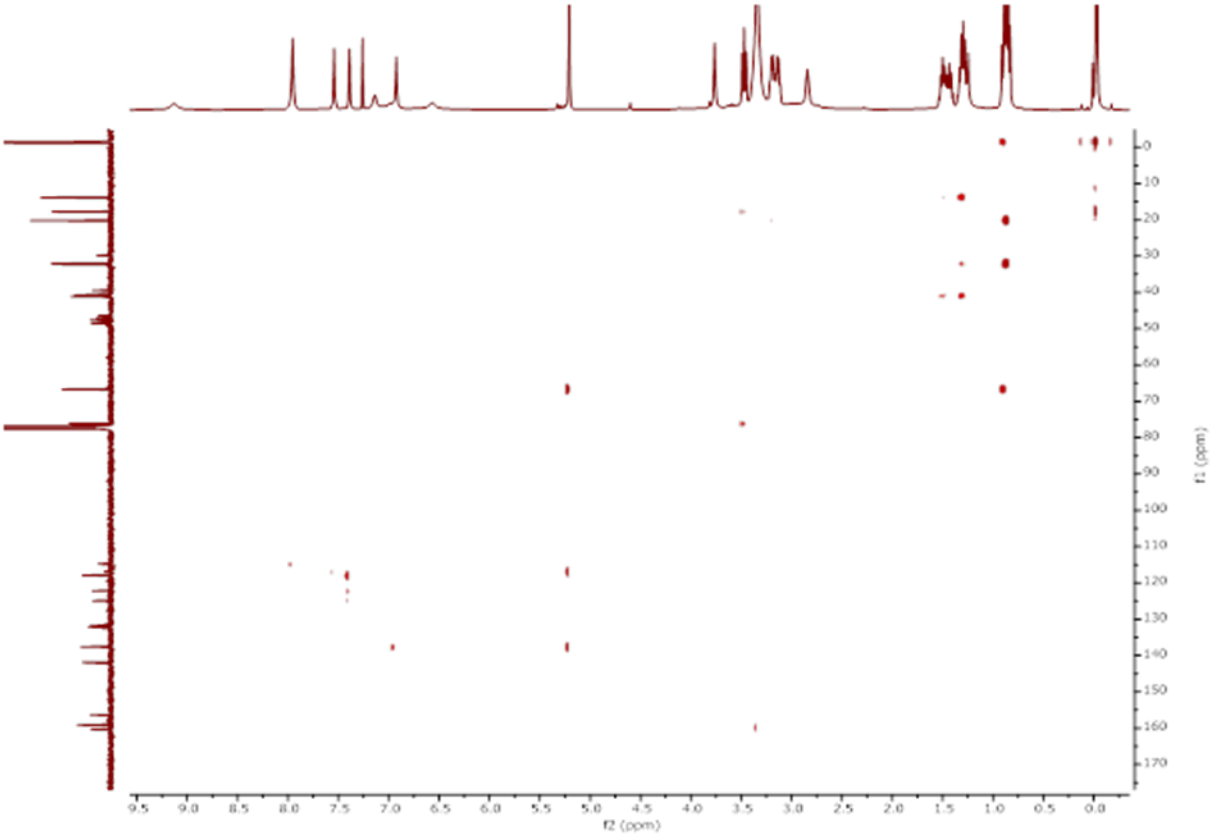
f2 (ppm)
f1 (ppm)

Supplementary Figure 10. ^{1}H NMR (400 MHz), COSY, ^{13}C NMR (100 MHz), HSQC, HMBC spectra of 1-(2-(3-(3,5-Bis(trifluoromethyl)phenyl)ureido)ethyl)-3-n-butyl-1-(2-(3-butyl-1-(2-(3-butyl-1-((1-((2-(trimethylsilyl)ethoxy)methyl)-1H-imidazol-4-yl)methyl)ureido)ethyl)ureido)ethyl)urea, 1fb (in $CDCl_3$)

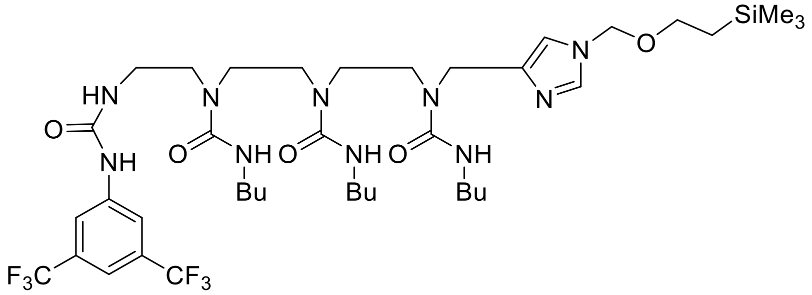

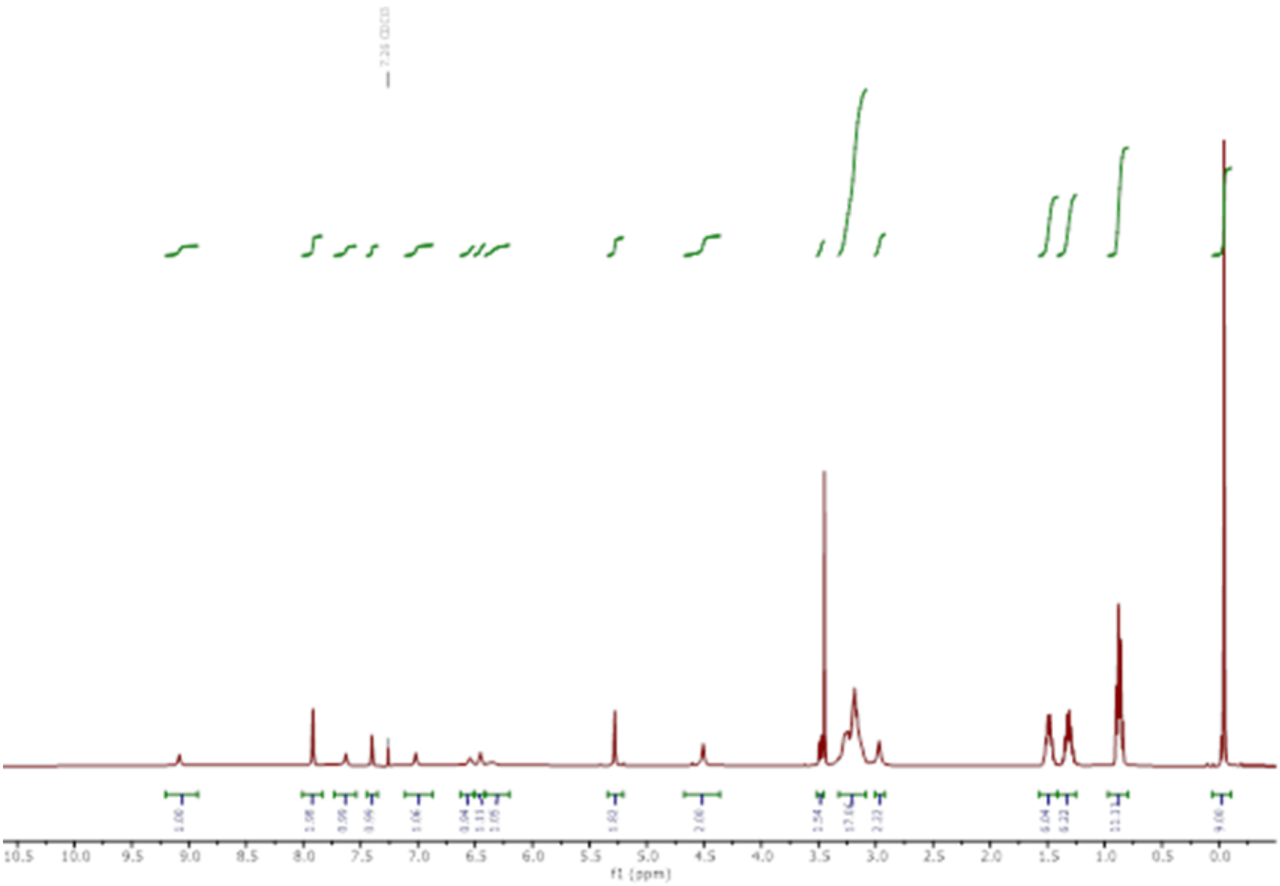

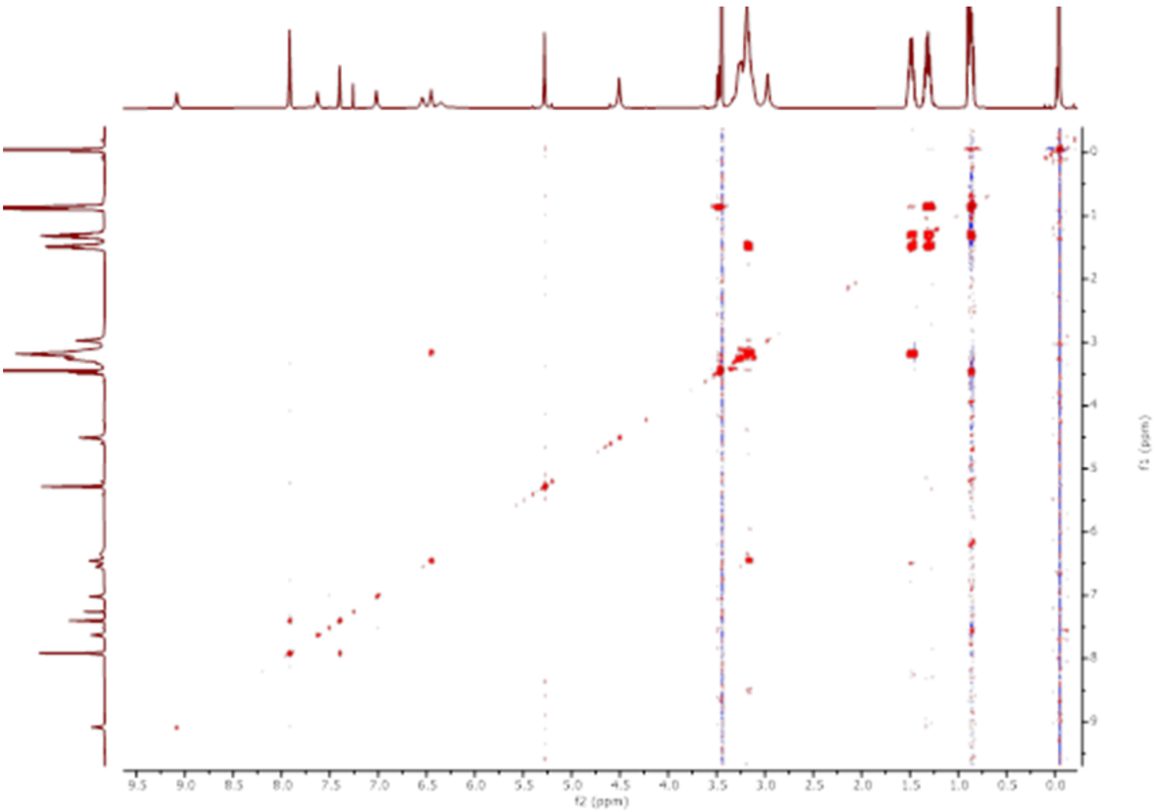
f2 (ppm)
f1 (ppm)

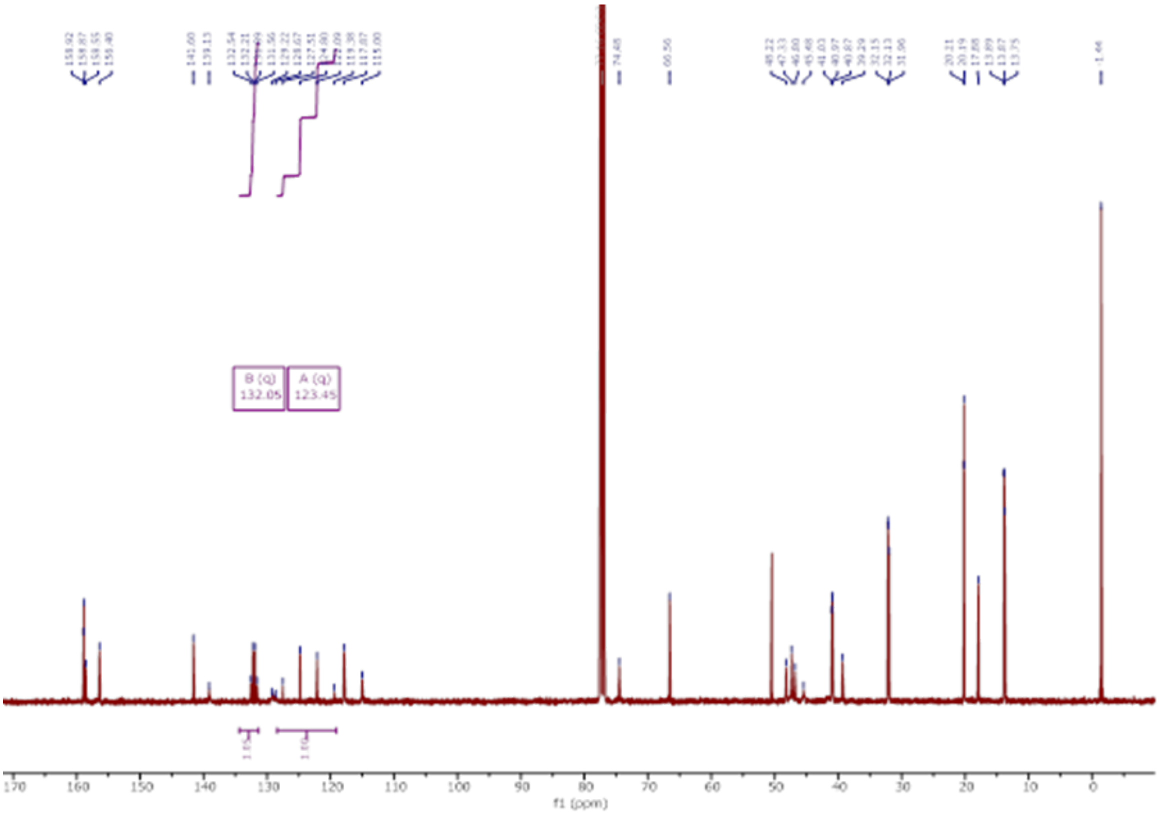
B (q)
132.05
A (q)
123.45
f1 (ppm)

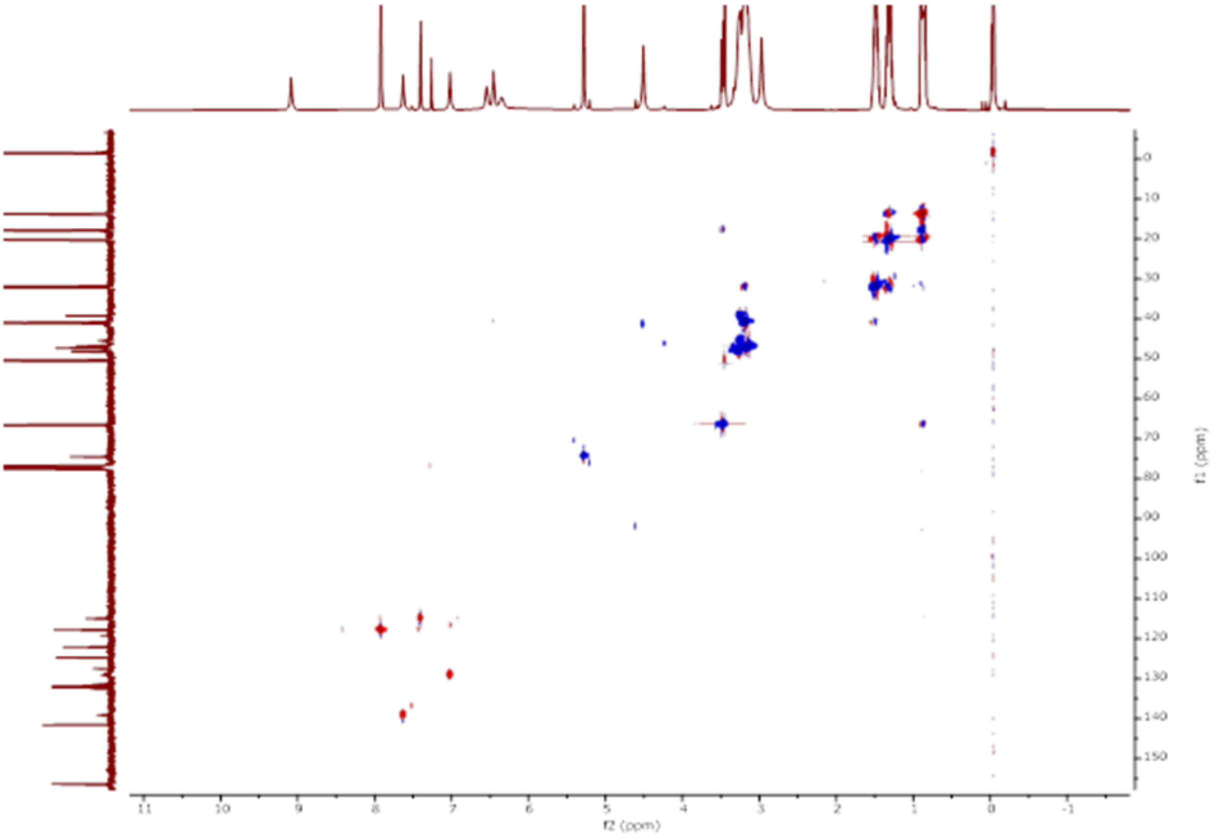
f2 (ppm)
f1 (ppm)

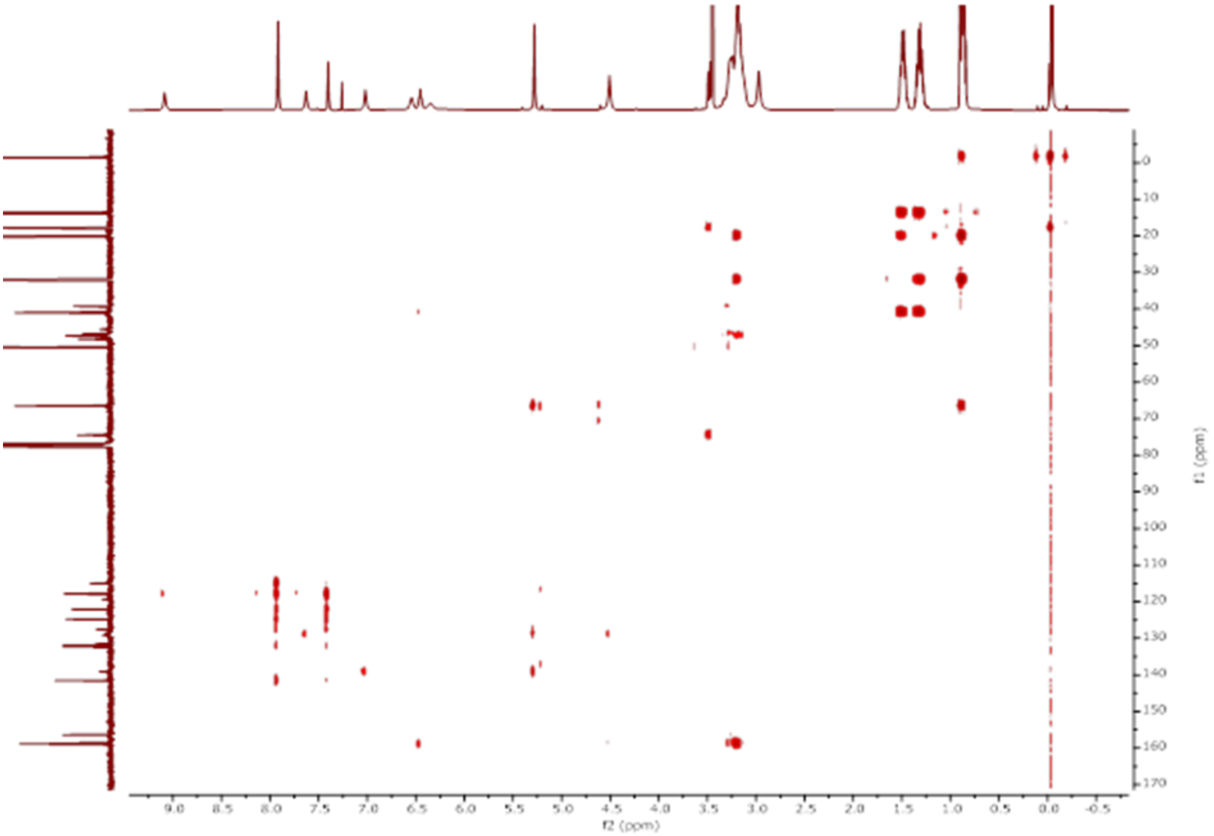
f2 (ppm)
f1 (ppm)

Supplementary Figure 11. ^{1}H NMR (400 MHz) and ^{13}C NMR (100 MHz) spectra of 1-(2-(3-(3,5-Bis(trifluoromethyl)phenyl)ureido)ethyl)-3-n-butyl-1-(2-(3-butyl-1-(2-(3-n-butyl-1-((1-((2-(trimethylsilyl)ethoxy)methyl)-1H-imidazol-5-yl)methyl)ureido)ethyl)ureido)ethyl)urea, 1fa (in $CDCl_3$)

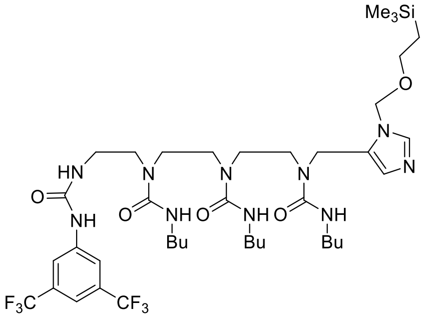

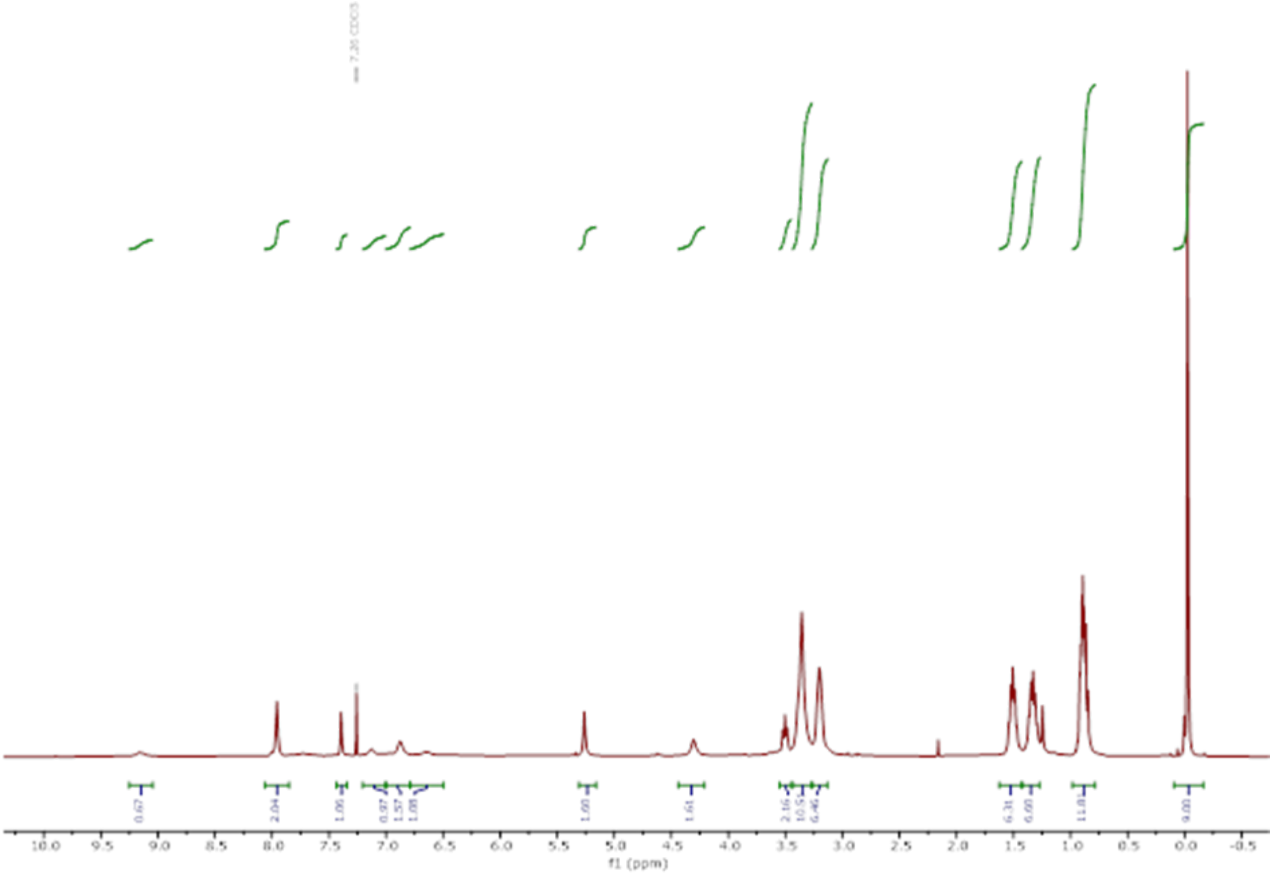

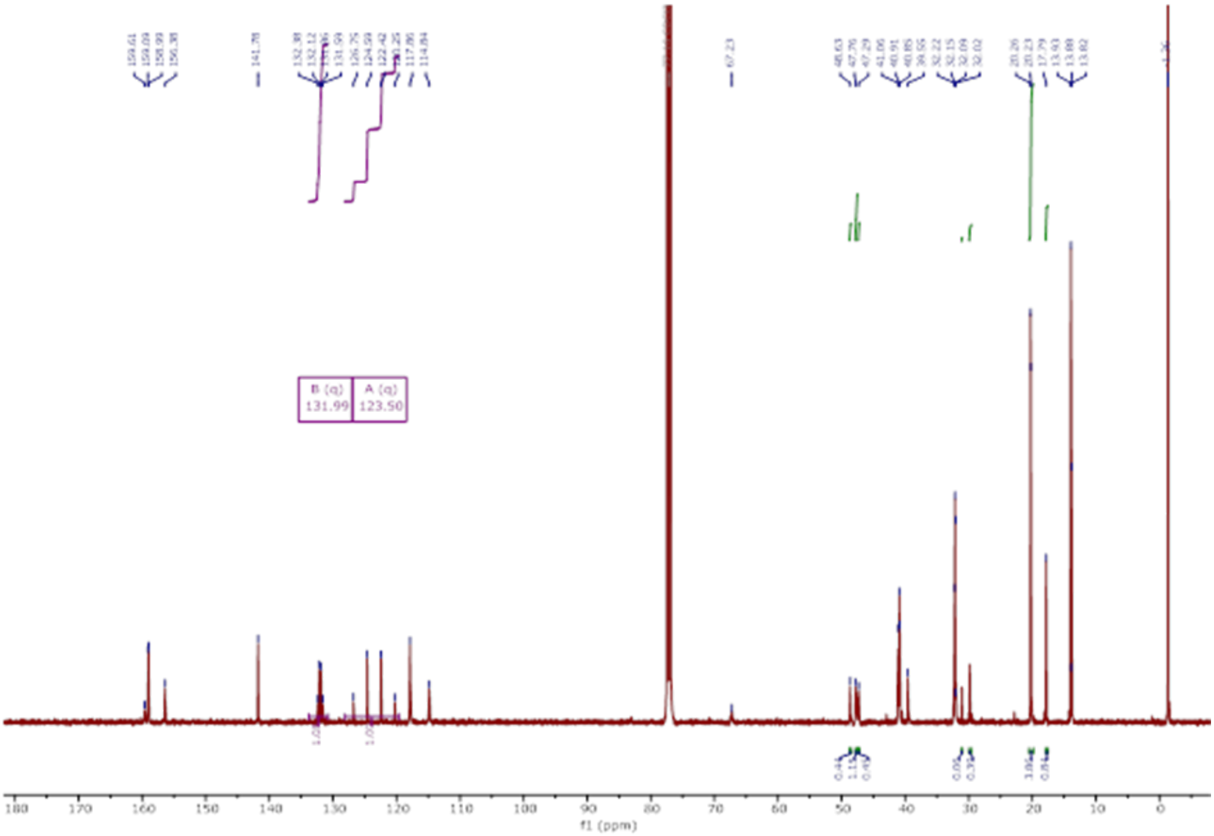
B (q)
131.99
A (q)
123.50
f1 (ppm)

Supplementary Figure 12. ^{1}H NMR (400 MHz) and ^{13}C NMR (100 MHz) spectra of the mixture of compounds **1fa** and **1fb** (in $CDCl_3$)

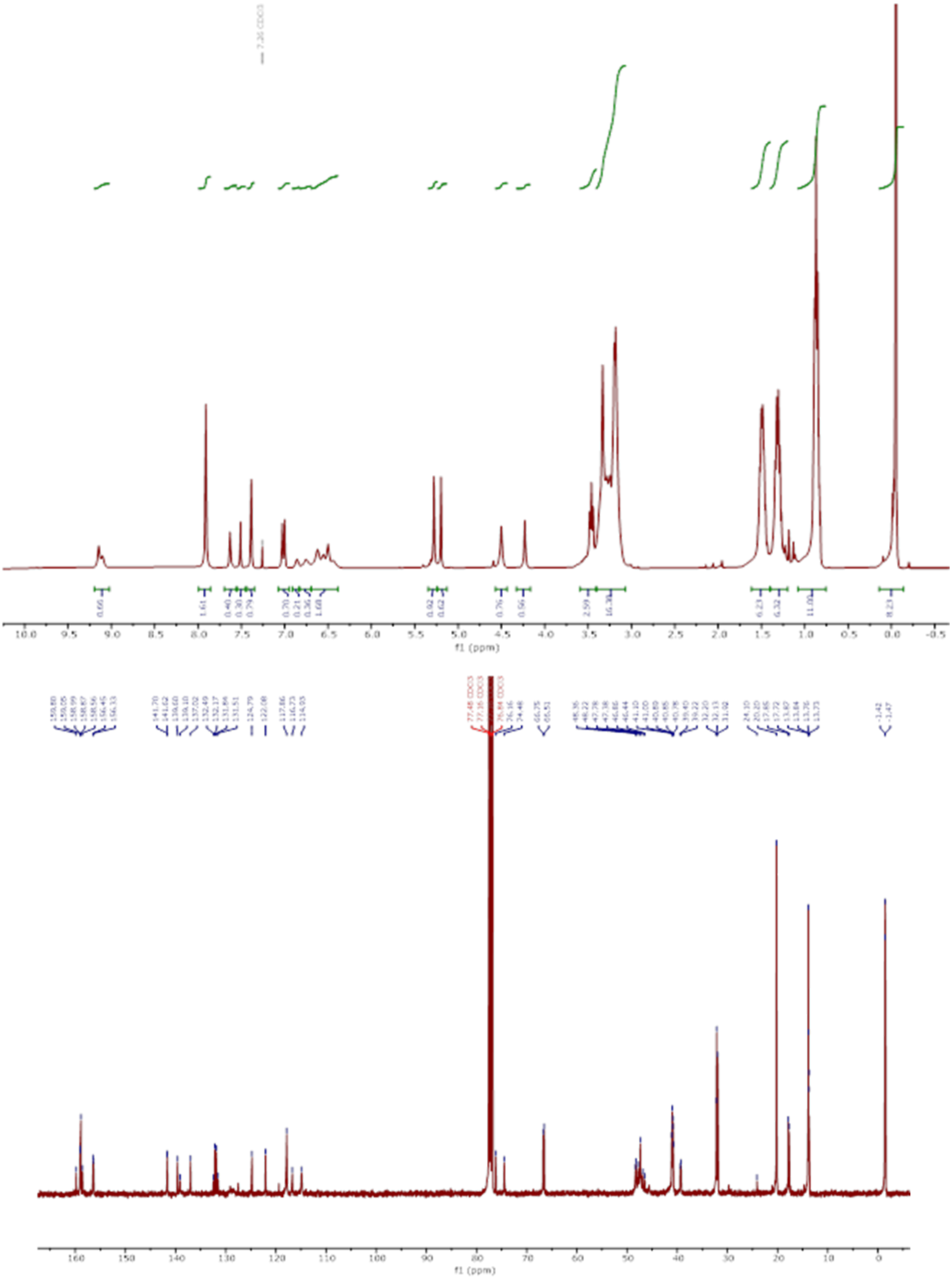

Supplementary Figure 13. ^{1}H NMR (400 MHz), COSY, ^{13}C NMR (100 MHz), HSQC, HMBC spectra of 1-(2-(1-((1H-Imidazol-4-yl)methyl)-3-n-butylureido)ethyl)-1-(2-(1-(2-(3-(3,5-bis(trifluoromethyl)phenyl)ureido)ethyl)-3-n-butylureido)ethyl)-3-n-butylurea, 1 (in $CDCl_3$)

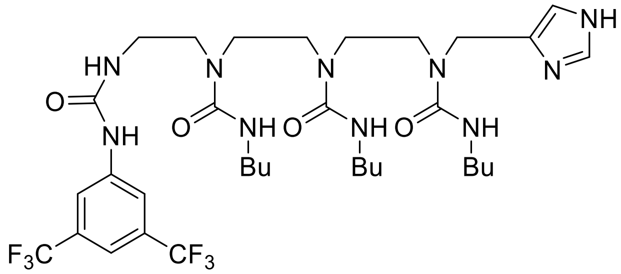

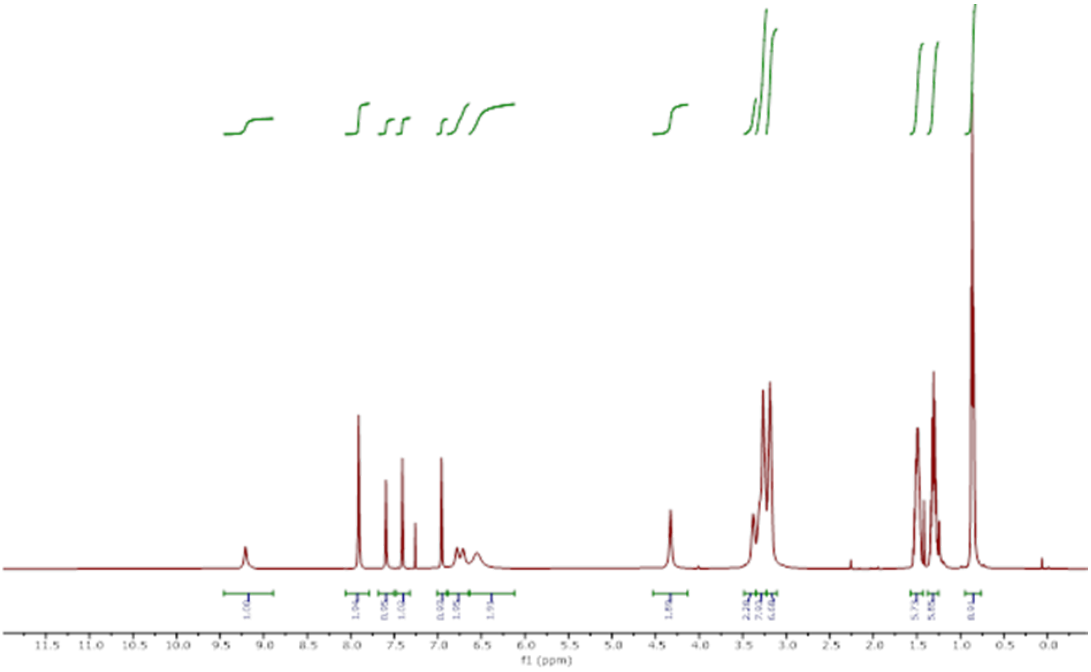

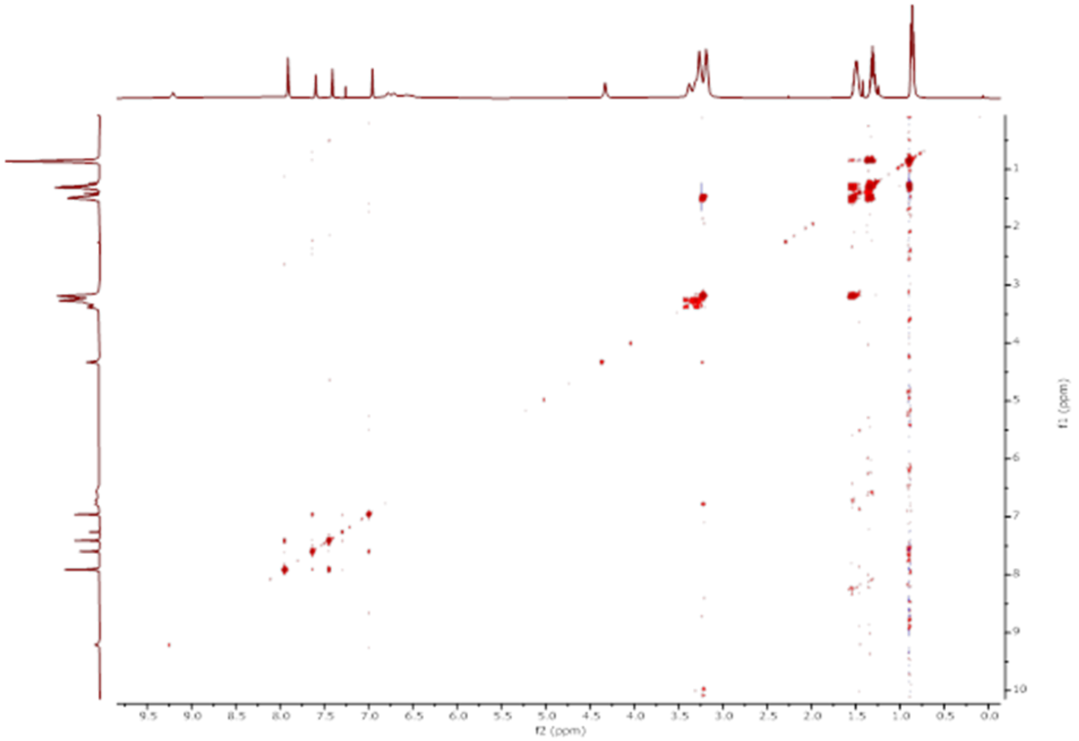

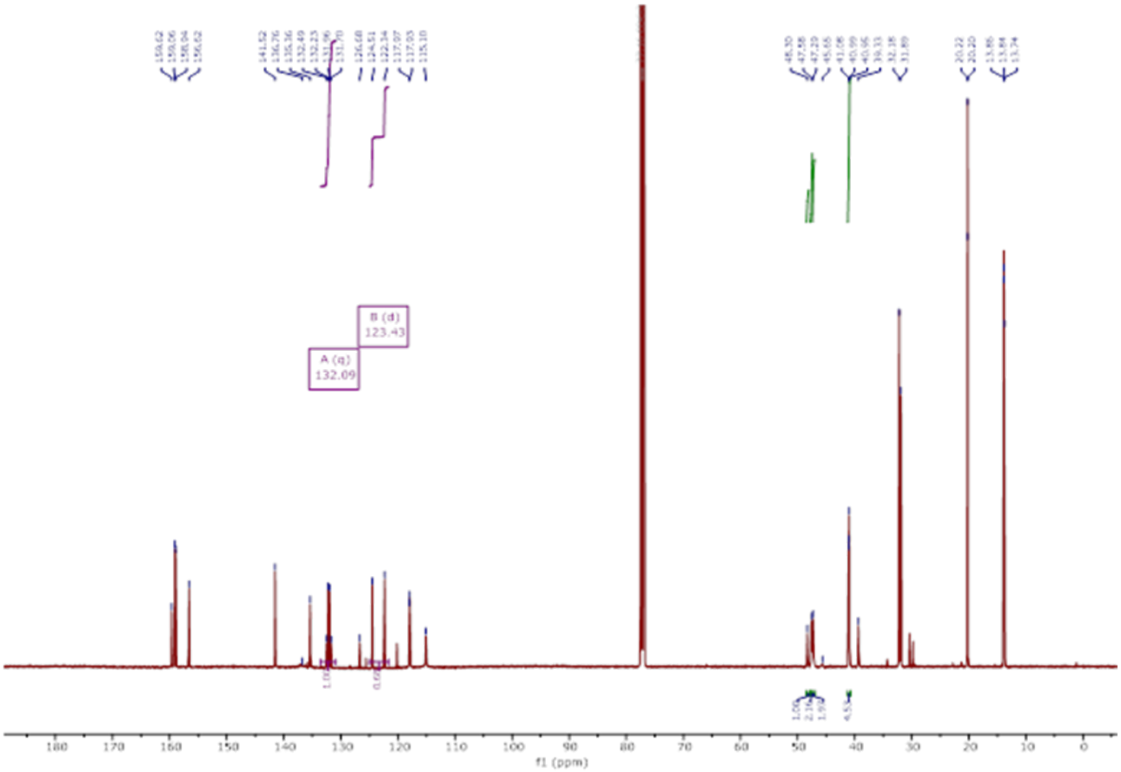
159.62
159.06
158.94
156.62
141.52
136.76
135.36
132.49
132.23
131.96
131.70
126.68
124.51
122.34
117.97
117.93
115.10
48.30
47.58
47.29
45.65
41.08
40.99
40.66
39.33
32.38
31.89
20.22
20.20
13.86
13.84
13.74
B (d)
123.43
A (q)
132.09
f1 (ppm)

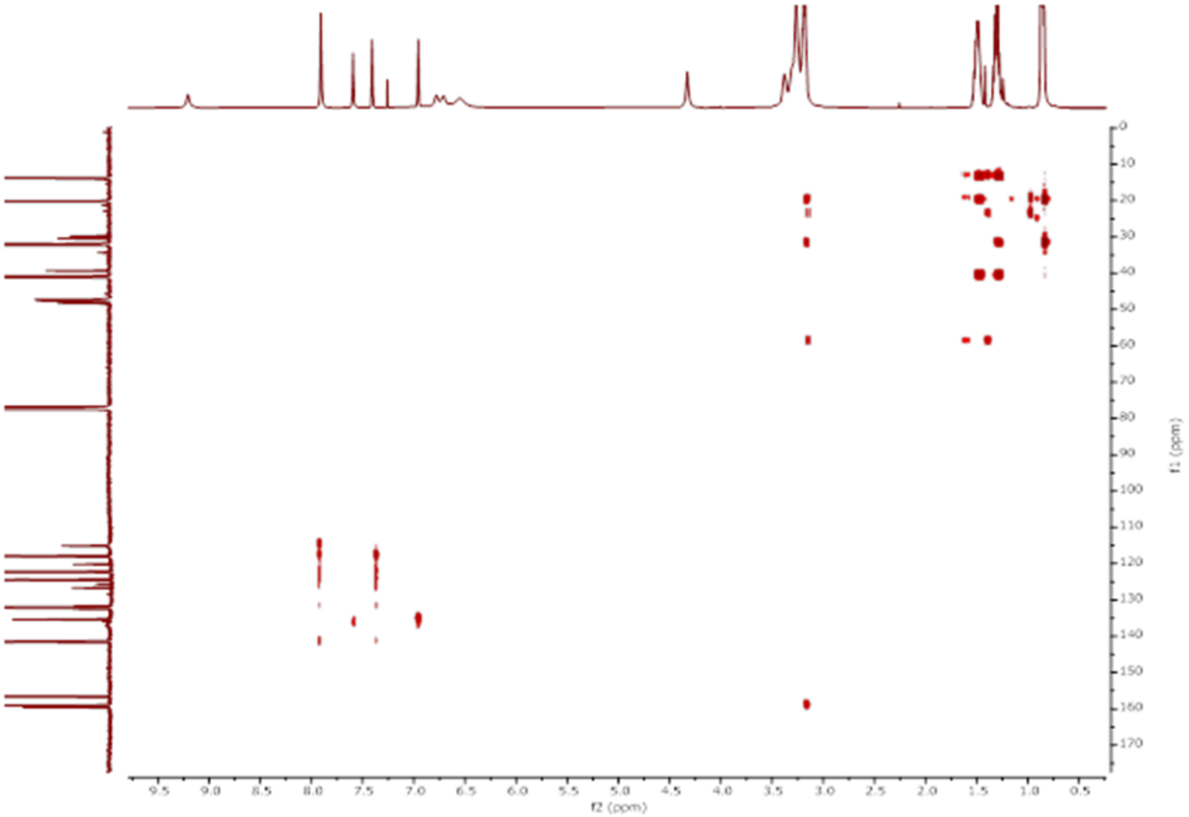
f2 (ppm)
f1 (ppm)

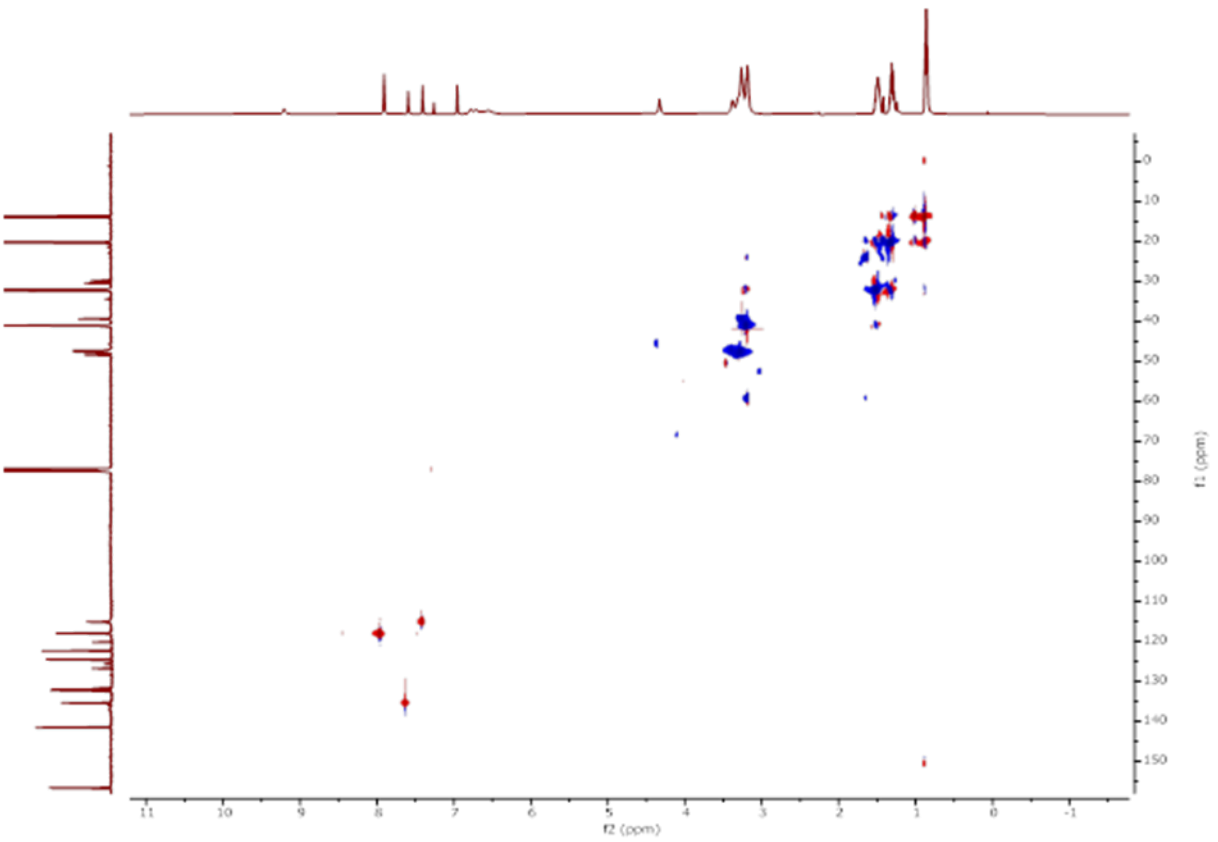
f2 (ppm)
f1 (ppm)

Supplementary Figure 14. ^{1}H NMR (400 MHz), ^{13}C NMR (100 MHz), HSQC, HMBC spectra of *N*-Trifluoroacetyl-*N*'-(n-butylcarbamoyl)-*N*'-ethyl-*N*''-ethylethane-1,2-diamine, 2b (in $CDCl_3$)

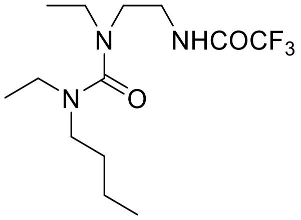

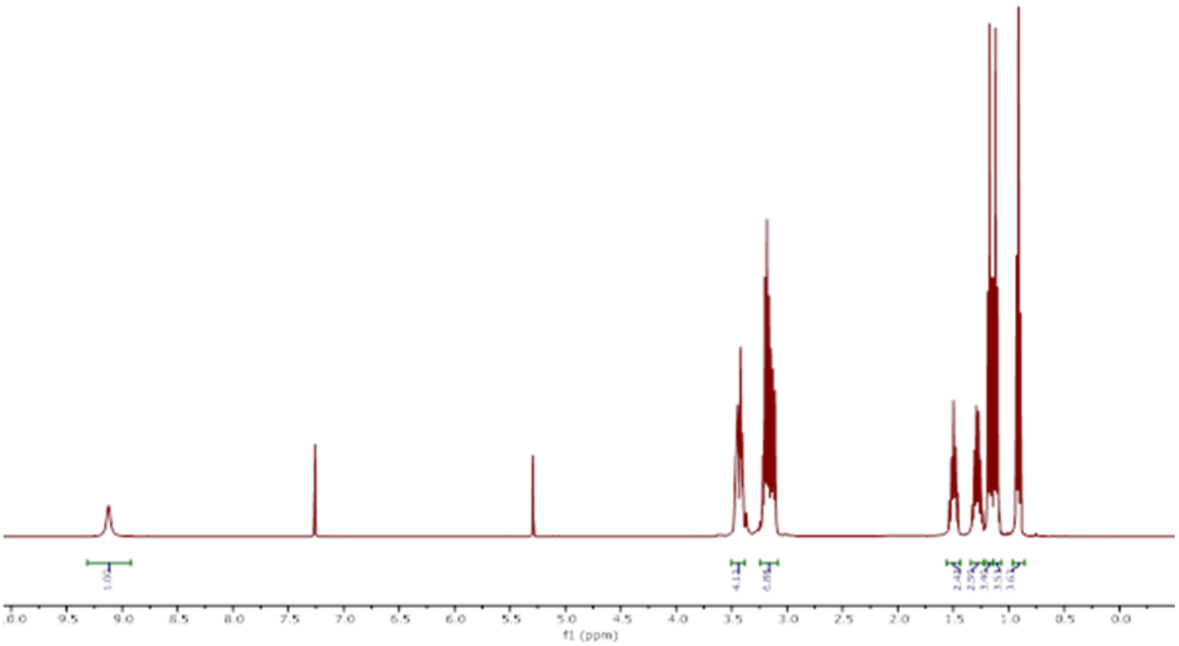

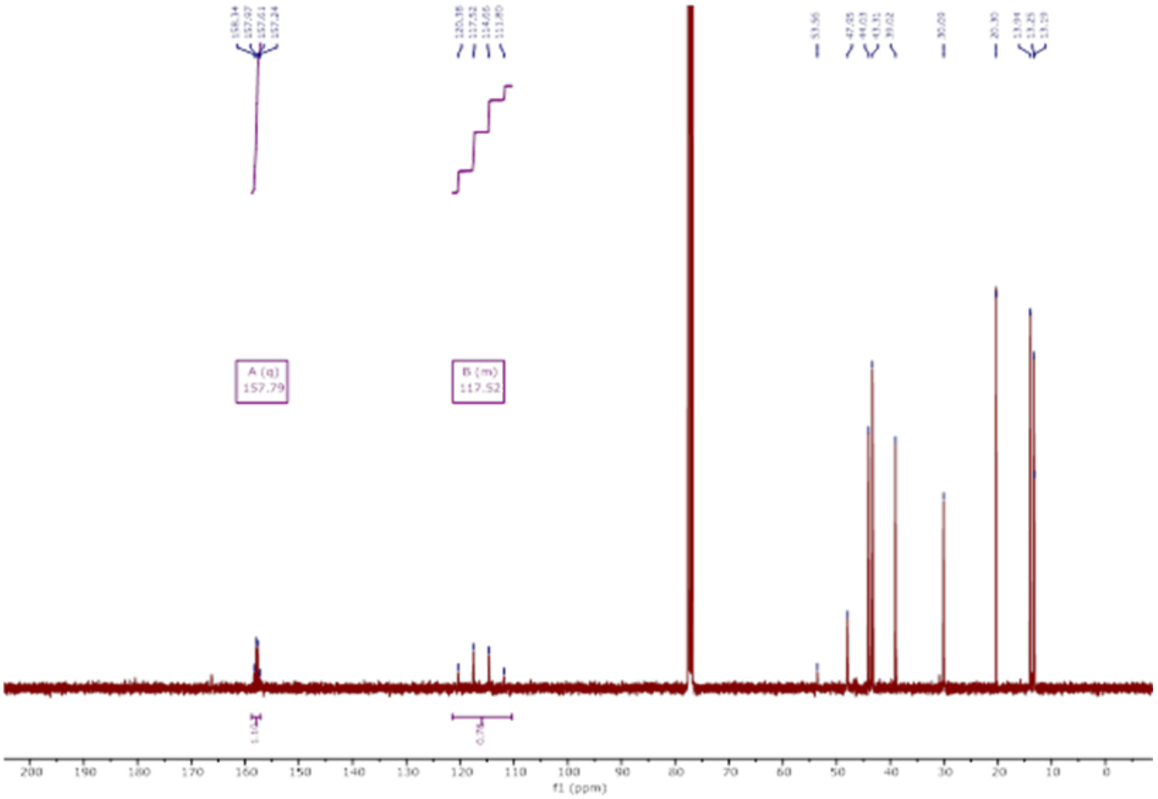

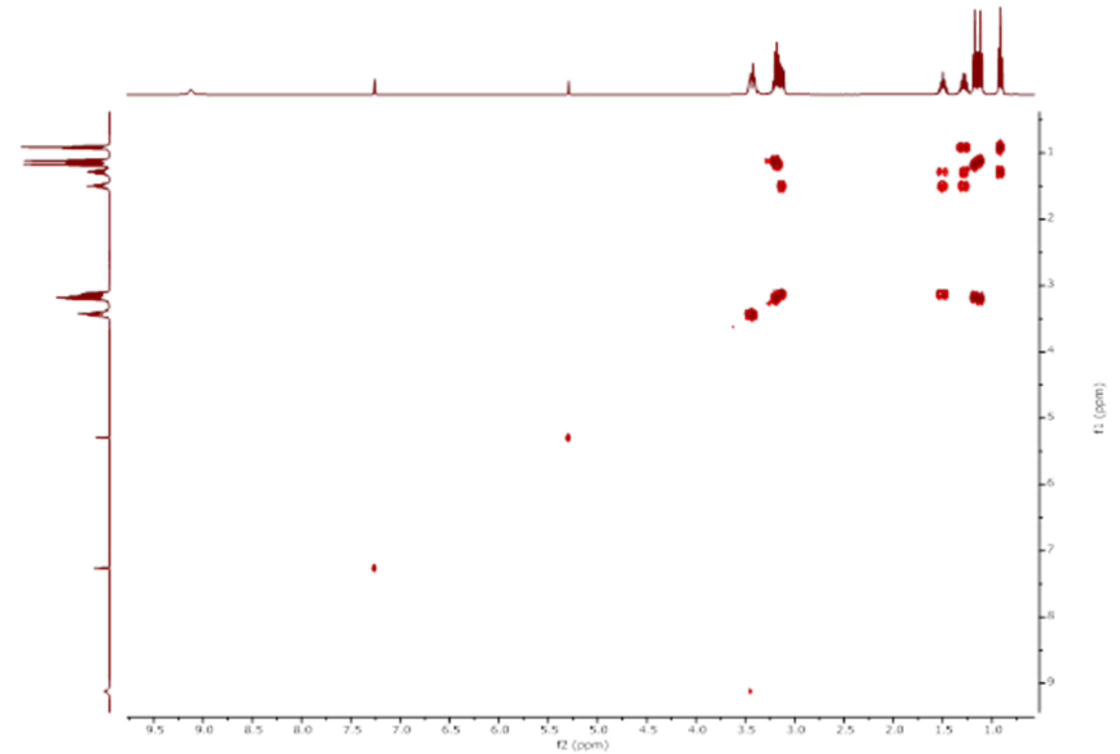
f2 (ppm)
f1 (ppm)

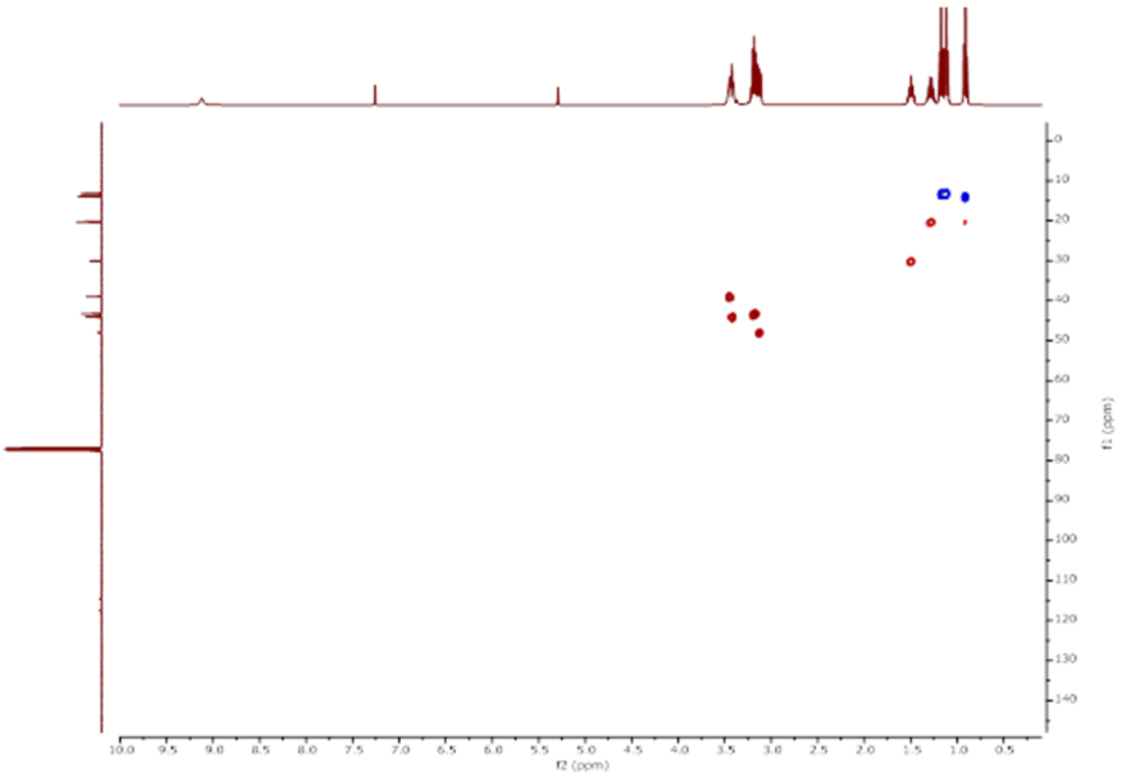
f2 (ppm)
f1 (ppm)

Supplementary Figure 15. ^{1}H NMR (400 MHz), COSY, ^{13}C NMR (100 MHz), HSQC spectra of *N*'-(n-Butylcarbamoyl)-N'-ethyl-N''-ethylethane-1,2-diamine, **2c** (in $CDCl_3$)

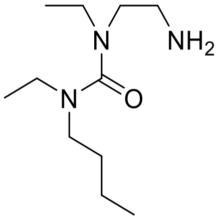

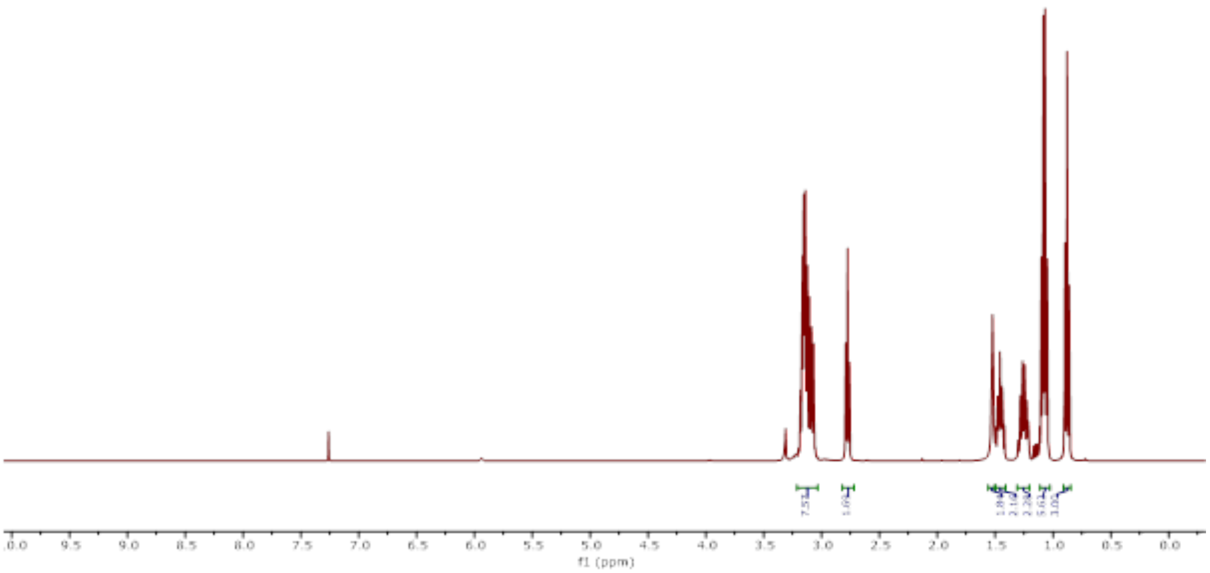

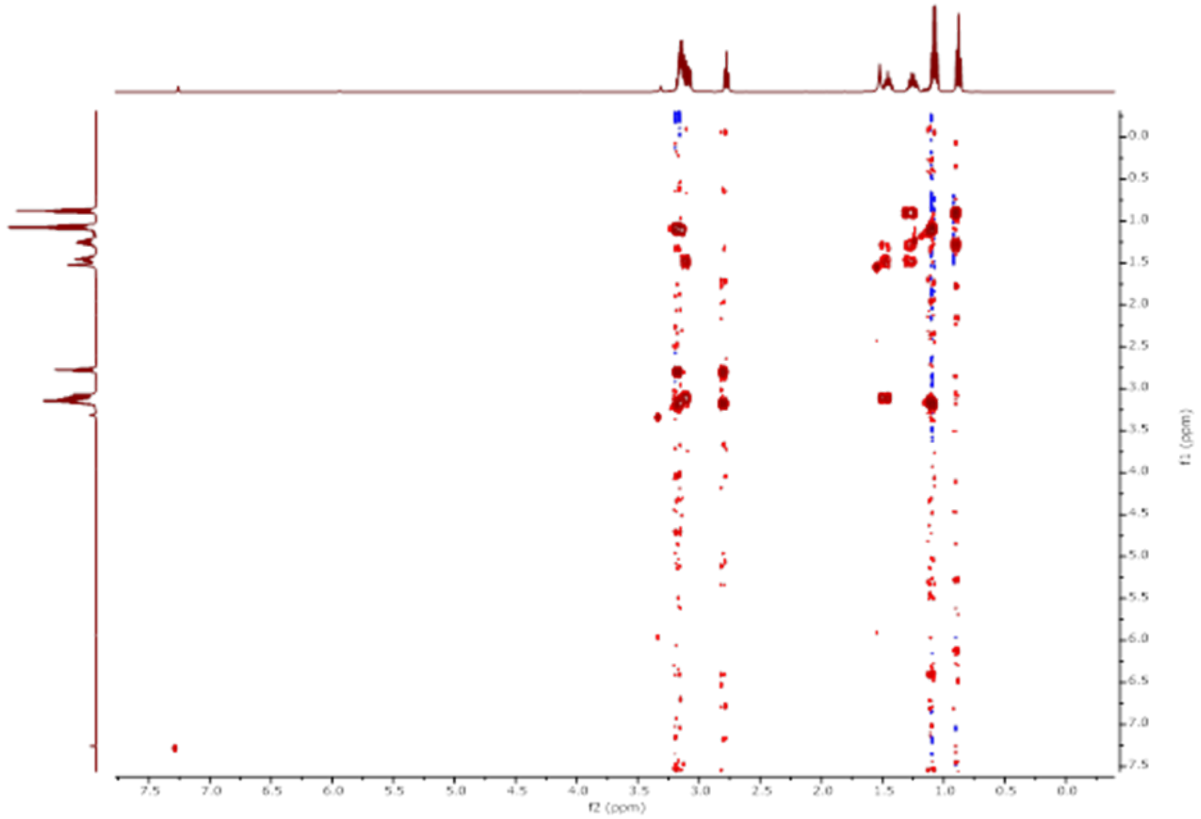

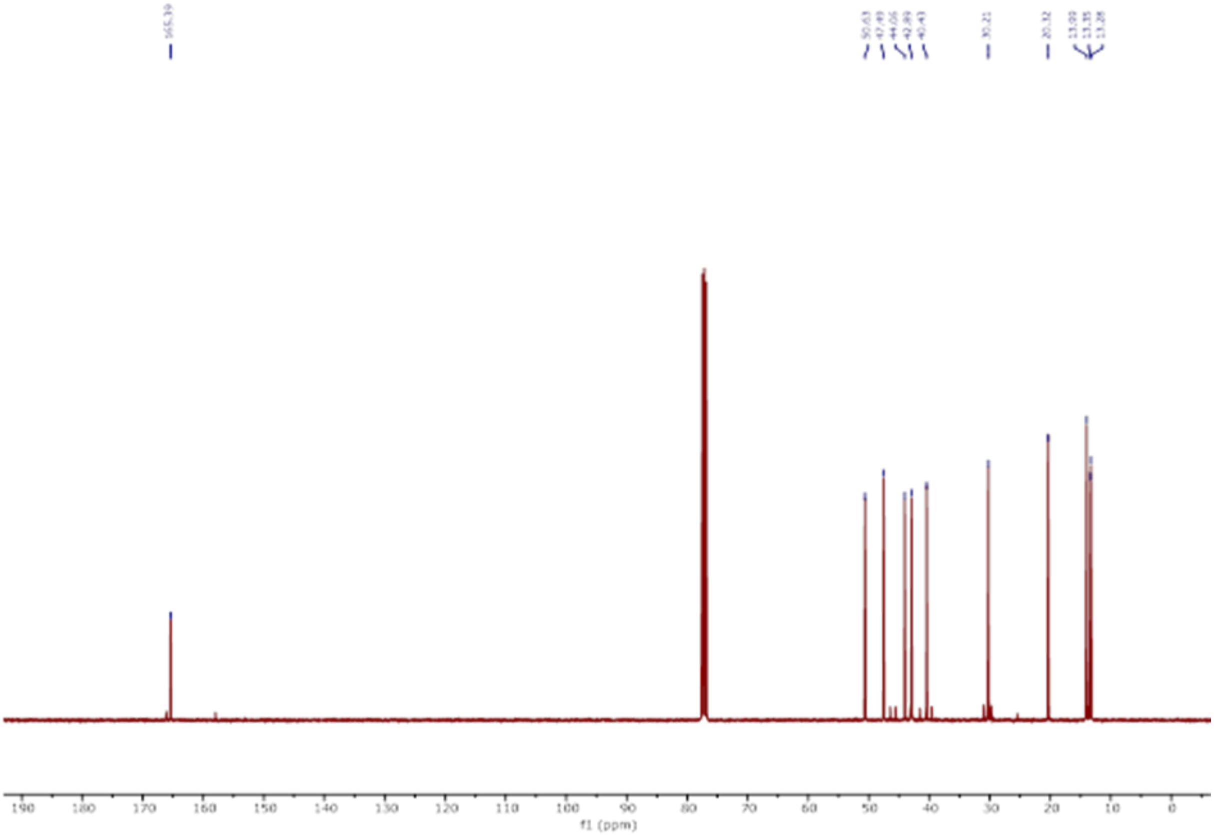
165.39
50.63
47.49
44.06
42.89
40.43
30.21
20.32
13.99
13.35
13.28
f1 (ppm)

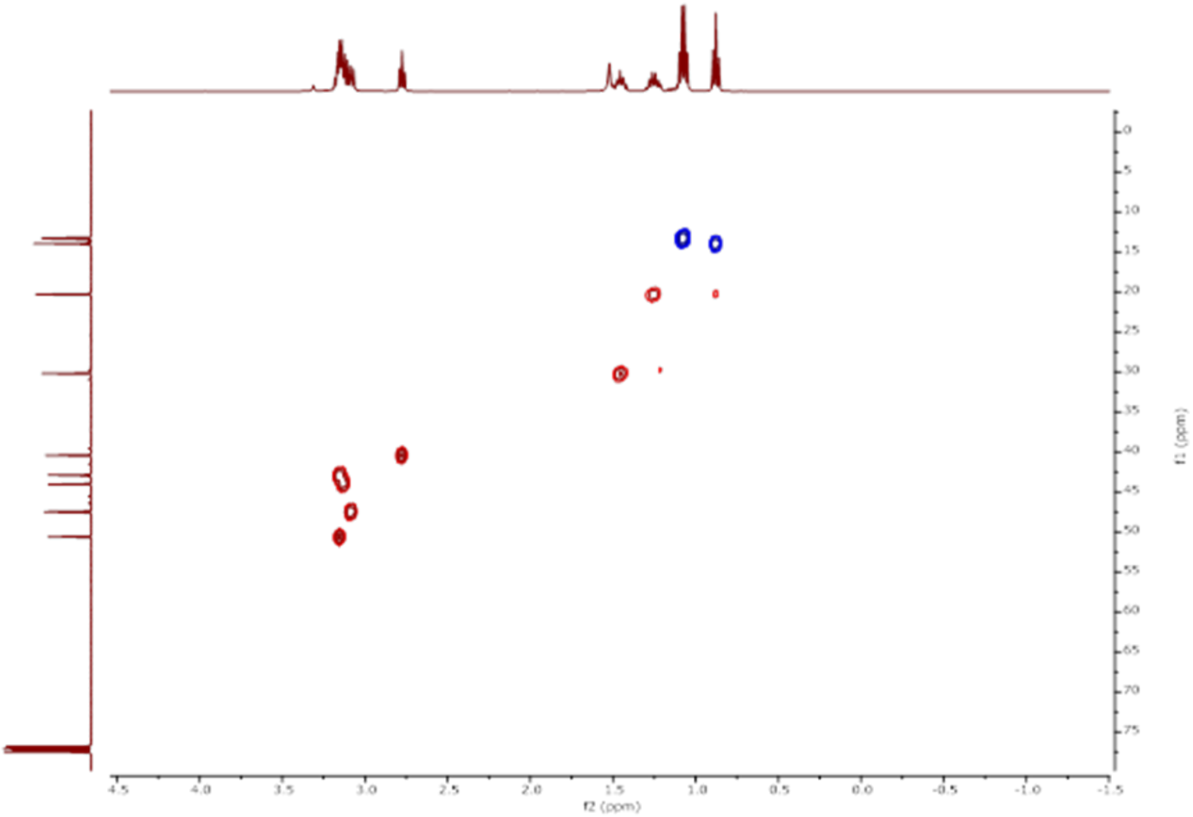
f2 (ppm)
f1 (ppm)

Supplementary Figure 16. ^{1}H NMR (400 MHz), COSY, ^{13}C NMR (100 MHz), HSQC, HMBC spectra of 1-n-Butyl-1,3-diethyl-3-(2-(((1-((2-(trimethylsilyl)ethoxy)methyl)-1H-imidazol-4-yl)methyl)amino)ethyl)urea, **2da** (in $CDCl_3$)

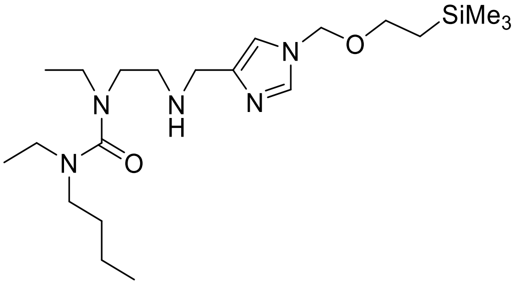

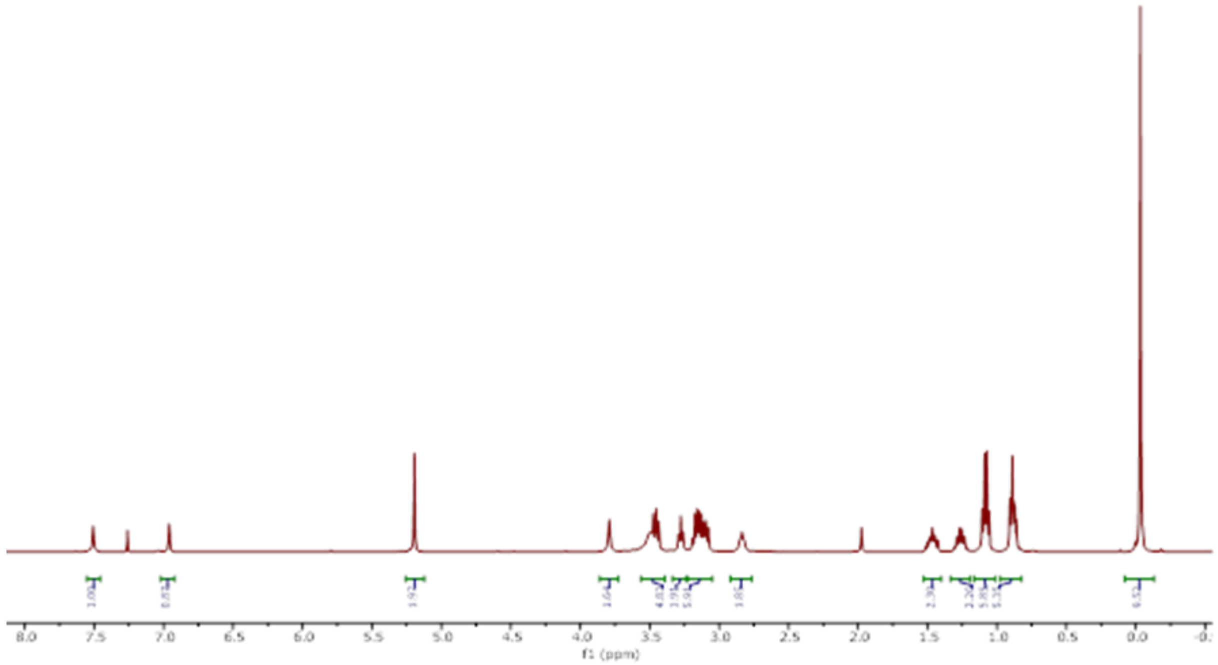

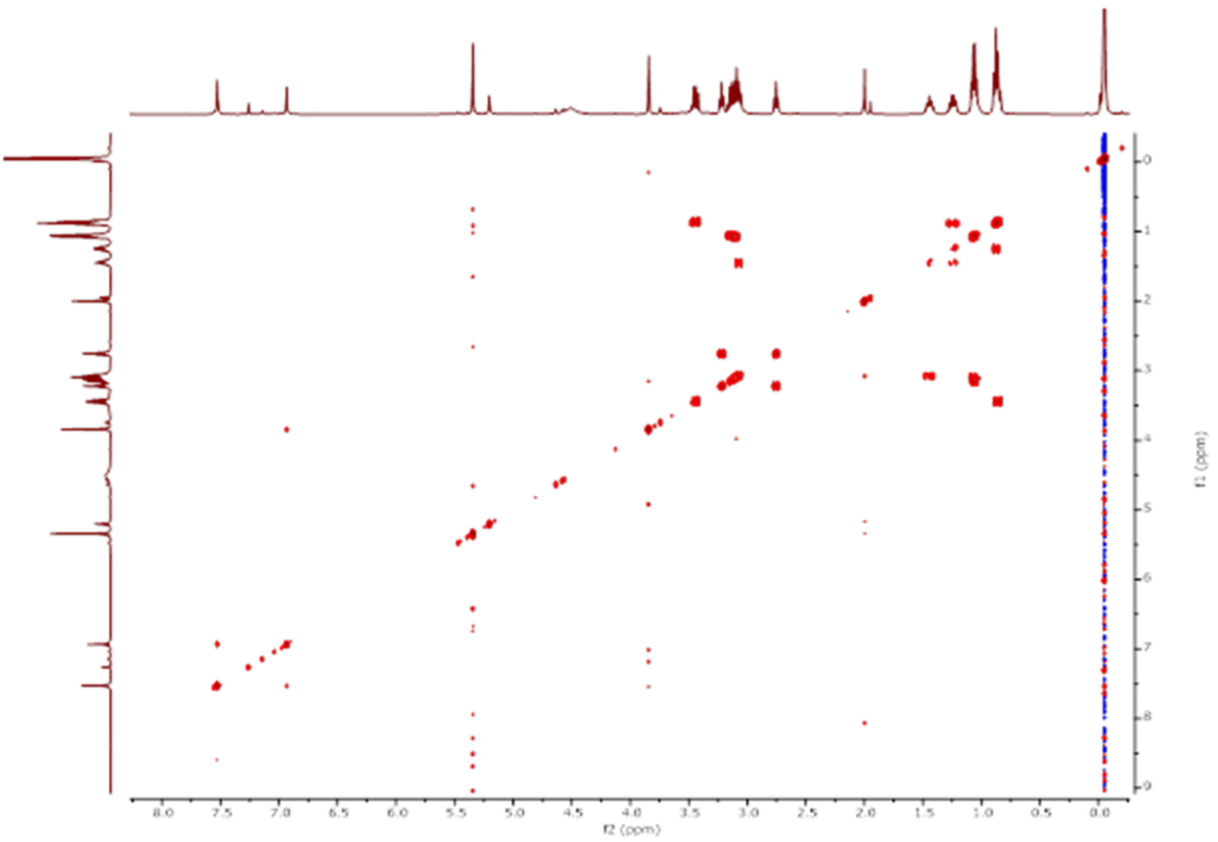

f2 (ppm)
f1 (ppm)

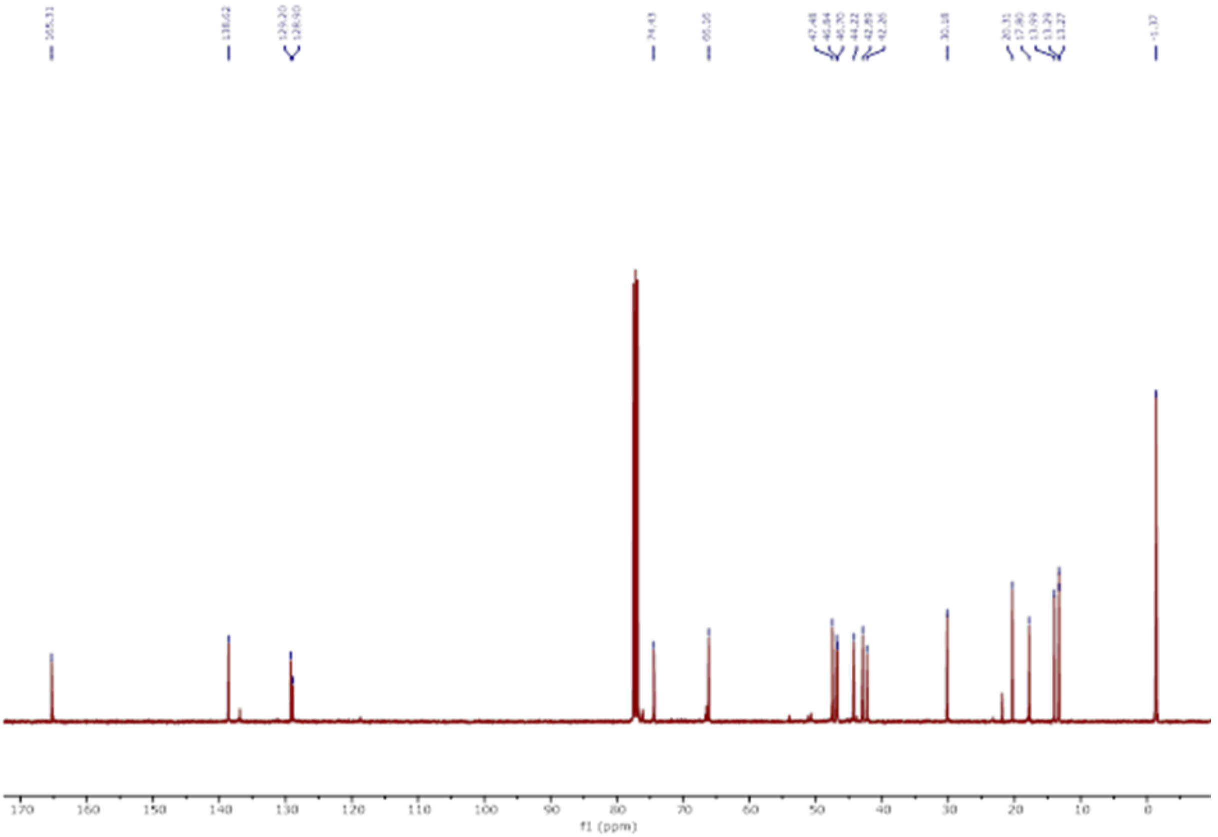

f1 (ppm)

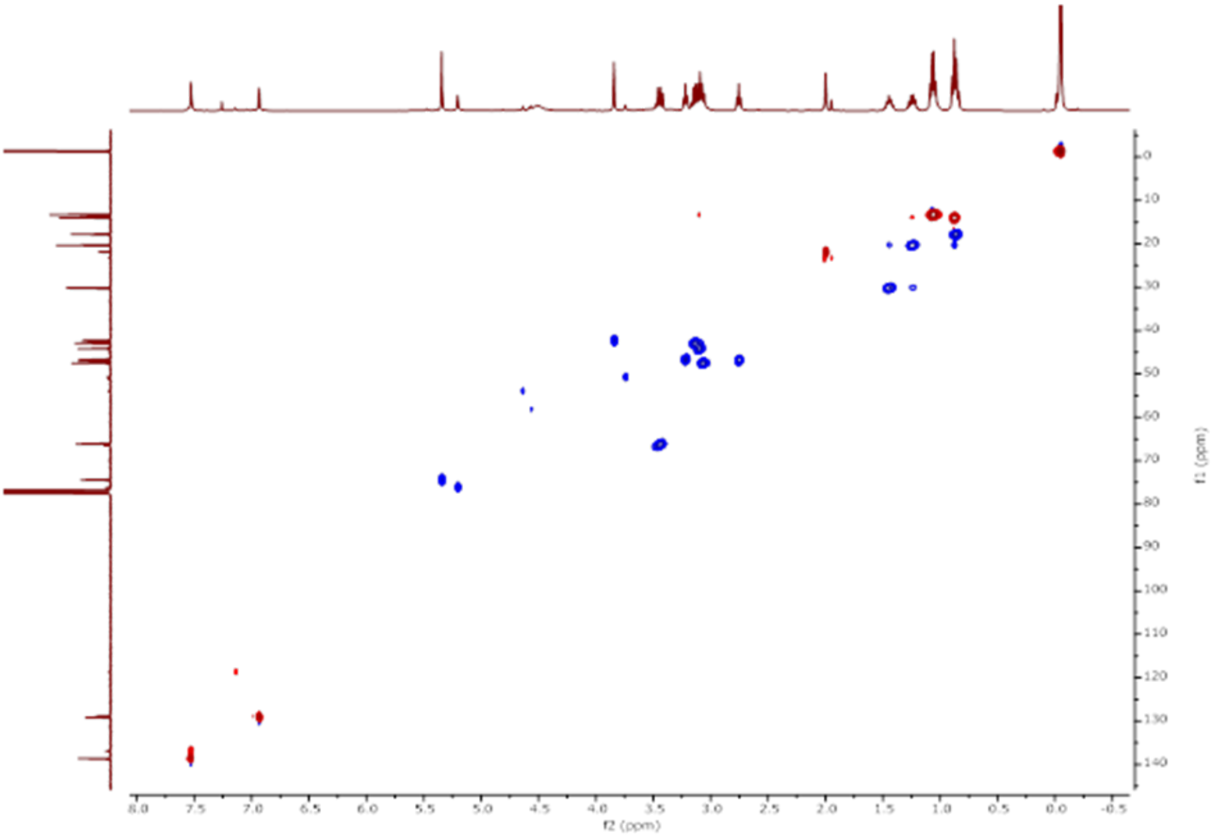
f2 (ppm)
f1 (ppm)

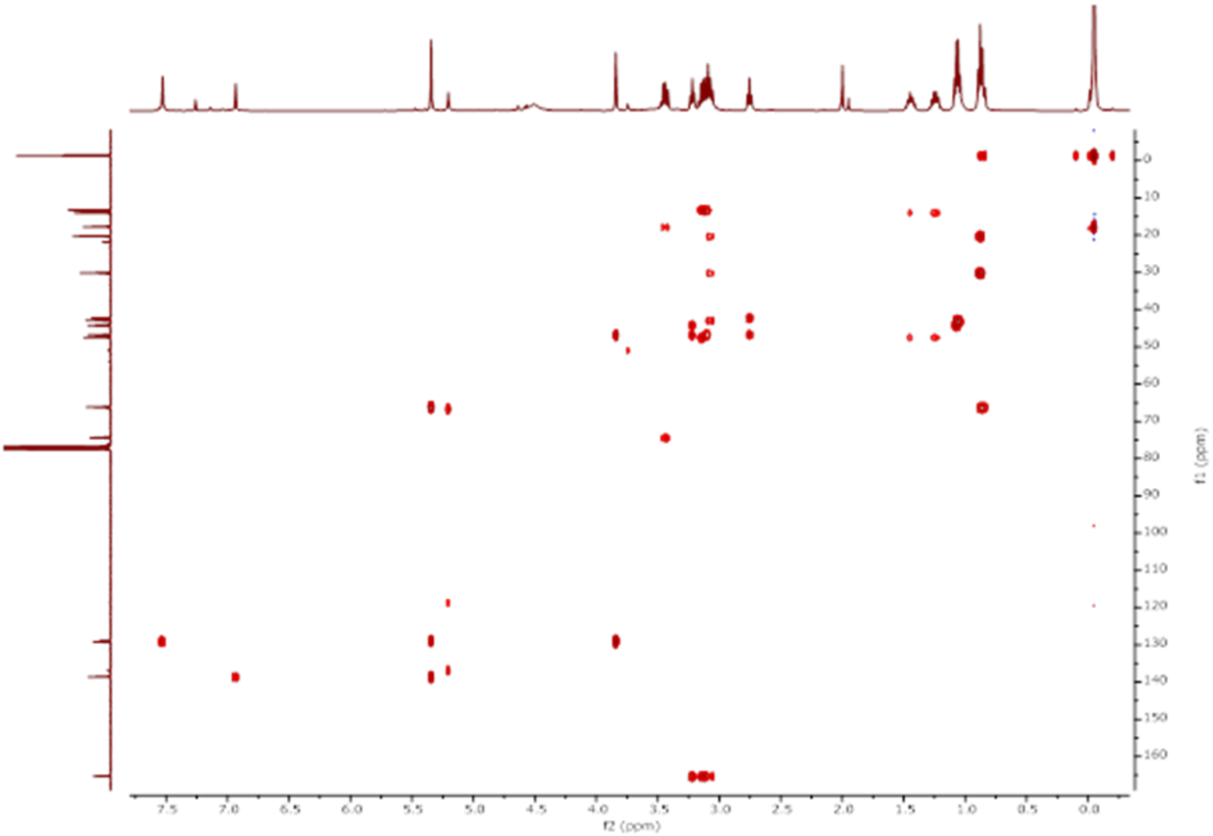
f2 (ppm)
f1 (ppm)

Supplementary Figure 17. ^{1}H NMR (400 MHz) and ^{13}C NMR (100 MHz) spectra of 1-n-Butyl-3-(2-(3-butyl-1-((1-((2-(trimethylsilyl)ethoxy)methyl)-1H-imidazol-4-yl)methyl)ureido)ethyl)-1,3-diethylurea, **2ea** (in $CDCl_3$)

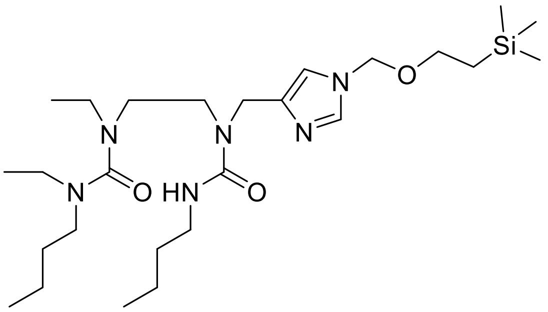

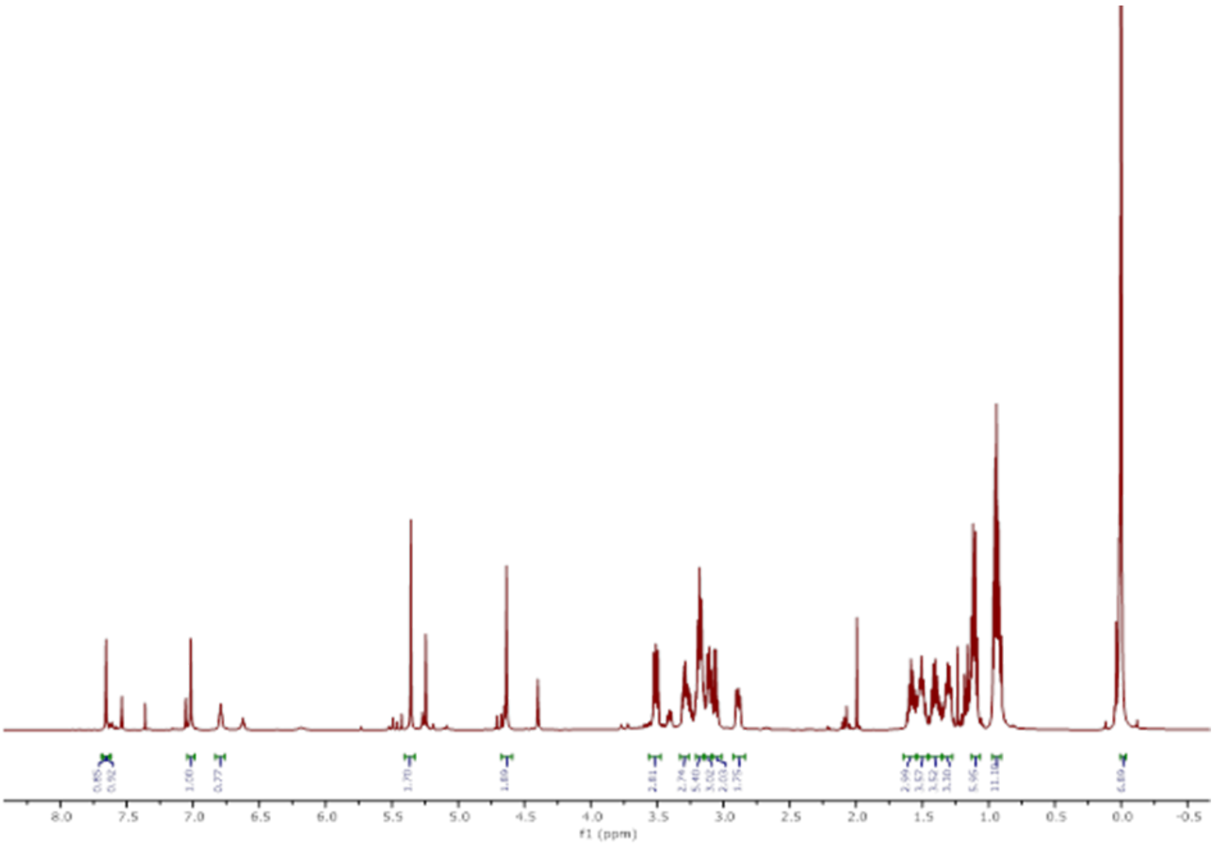

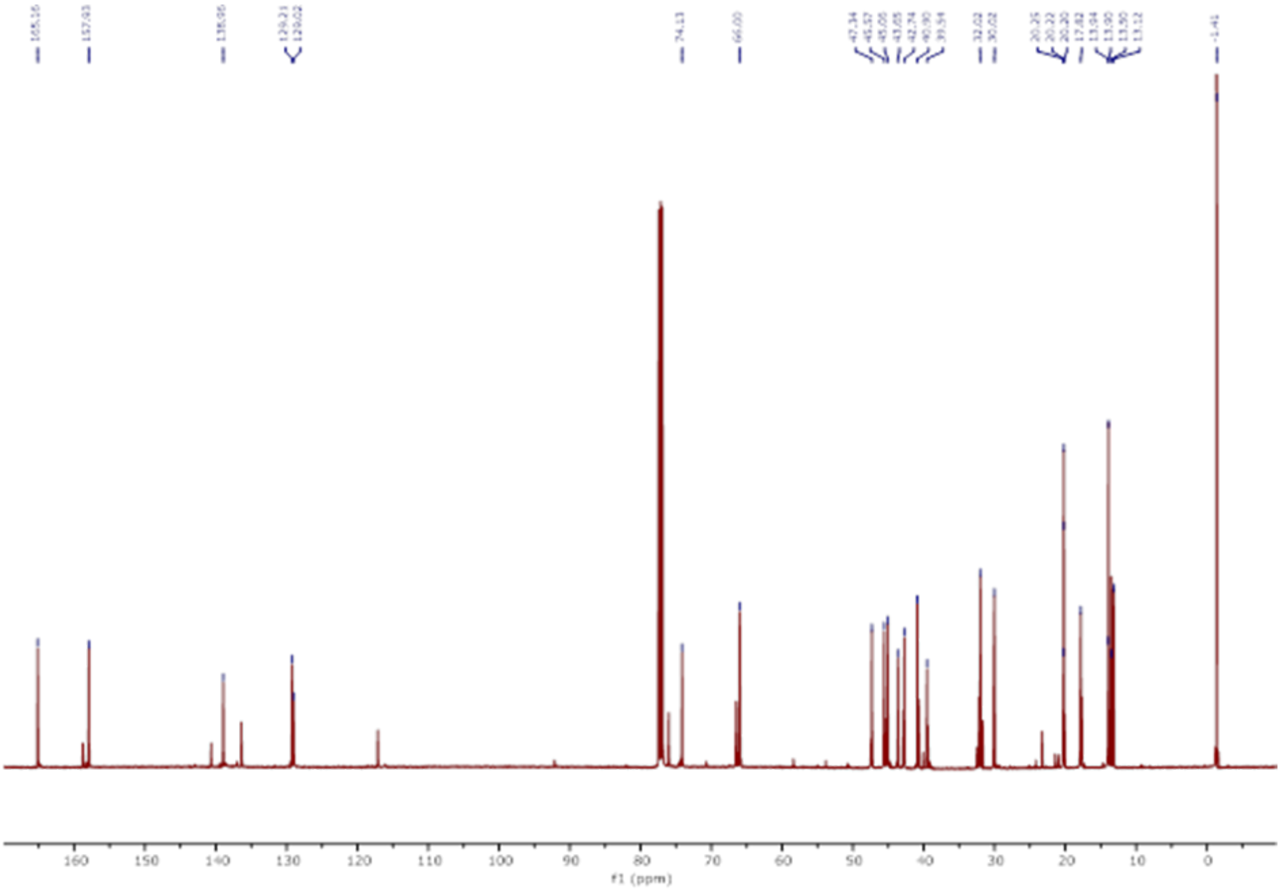
165.16
157.93
138.95
129.21
129.02
74.13
66.00
47.34
45.57
45.06
43.65
42.74
40.90
39.54
32.02
30.02
20.26
20.22
20.20
17.82
13.94
13.90
13.50
13.12
-1.41
160 150 140 130 120 110 100 90 80 70 60 50 40 30 20 10 0
f1 (ppm)

Supplementary Figure 18. ^{1}H NMR (500 MHz), COSY, ^{13}C NMR (125 MHz), HSQC, HMBC, NOESY and NOE spectra of 1-(2-(1-((1H-Imidazol-5-yl)methyl)-3-n-butylureido)ethyl)-3-n-butyl-1,3-diethylurea, **2** (in $CDCl_3$)

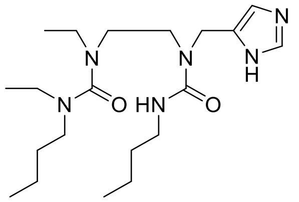

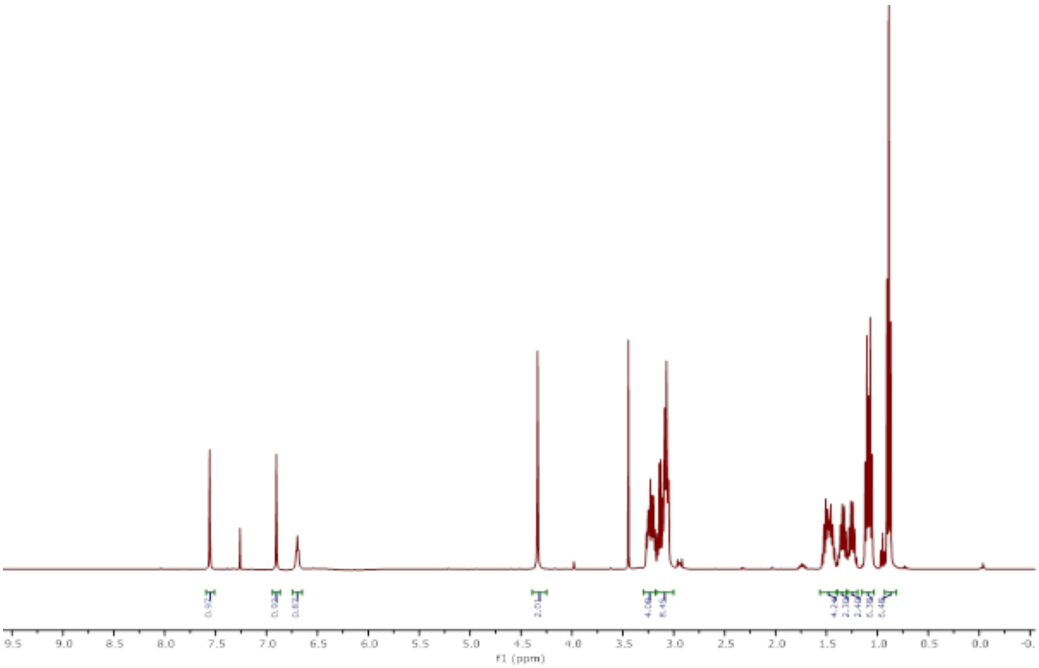

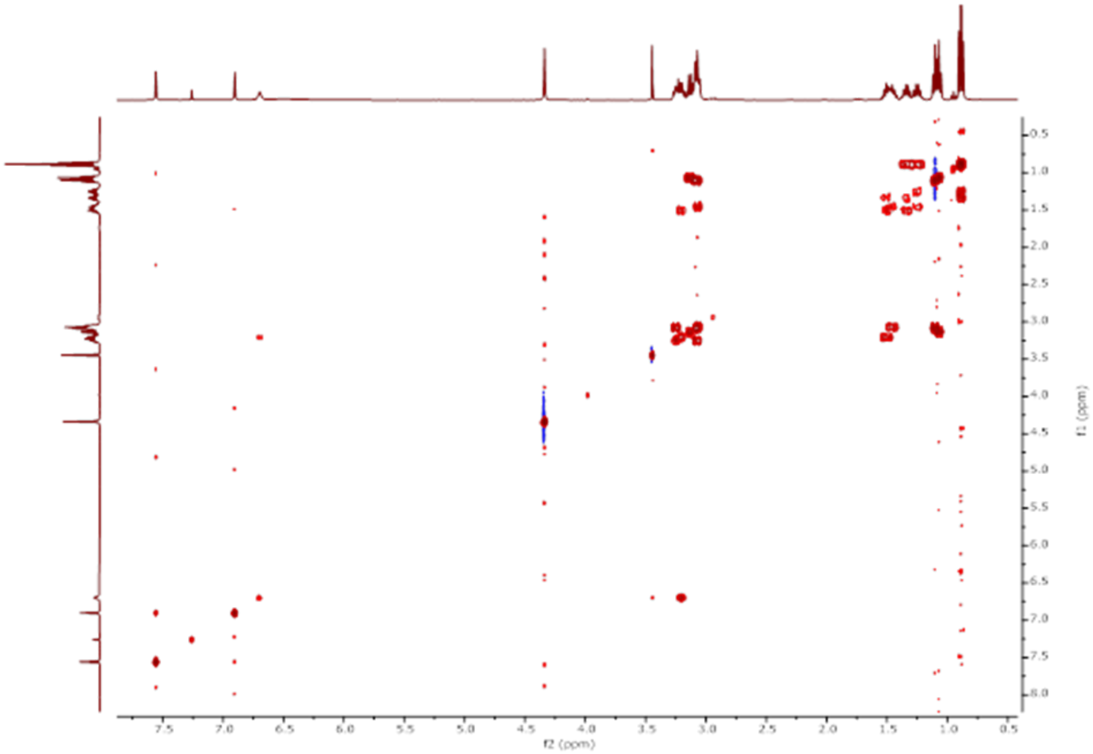

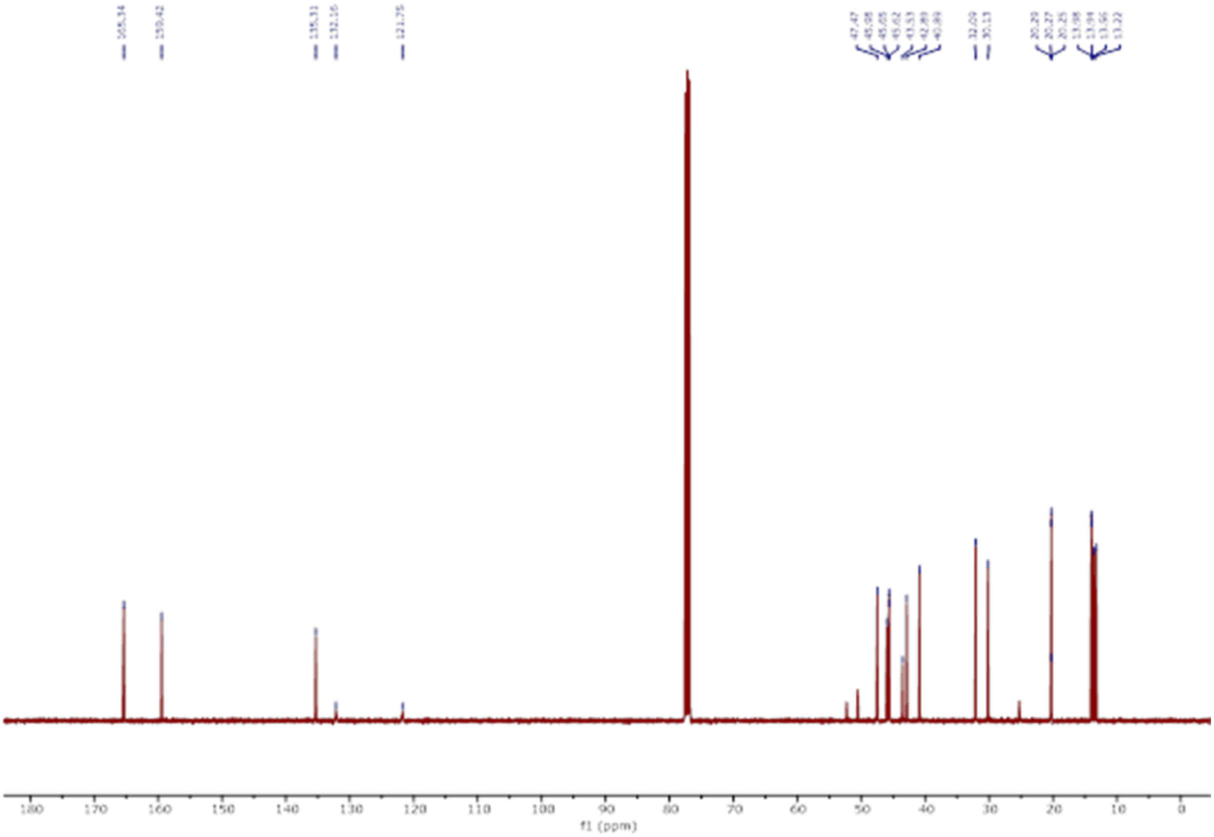
165.34
159.42
135.31
132.16
121.75
47.47
45.65
45.62
43.53
32.09
30.13
20.29
20.27
20.25
13.98
13.94
13.56
13.22
f1 (ppm)

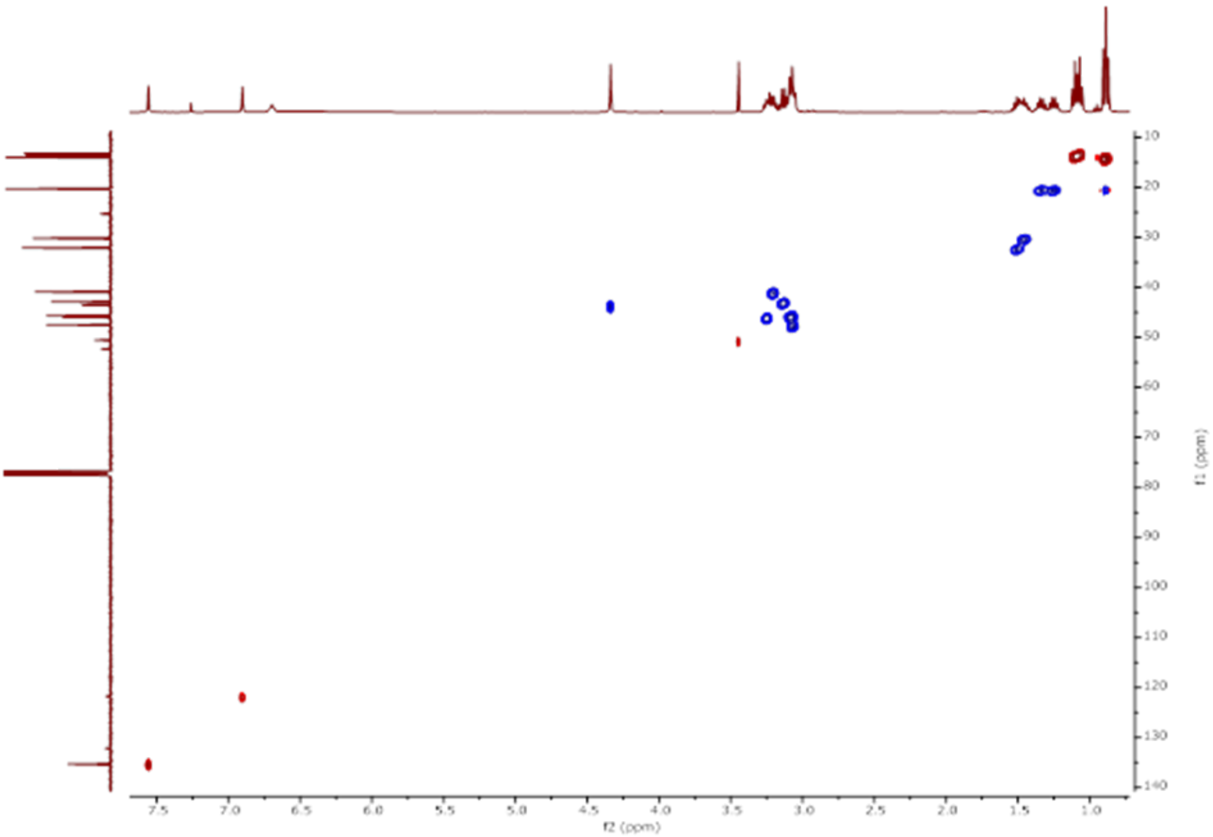
f2 (ppm)
f1 (ppm)

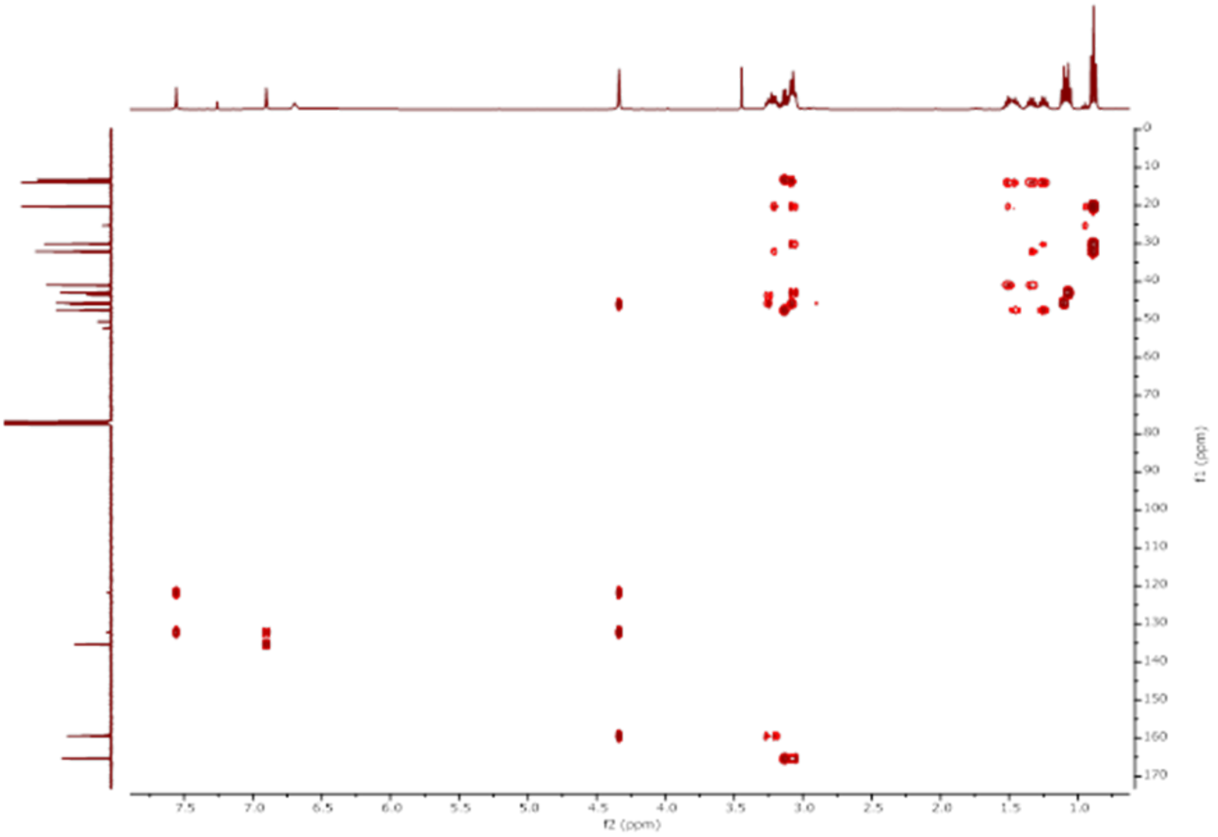

2D ^{1}H-^{1}H NOESY

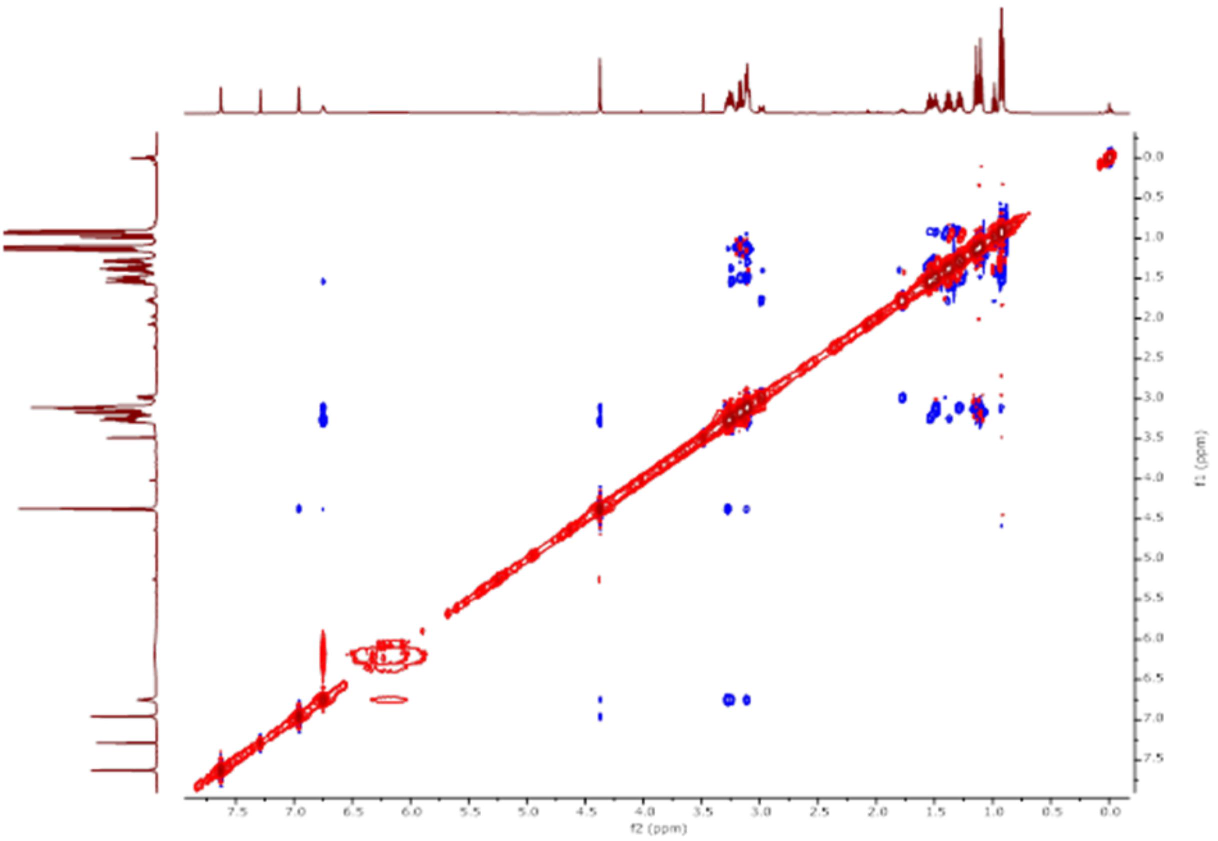

NOE spectrum irradiating the N*H*Bu frequency.

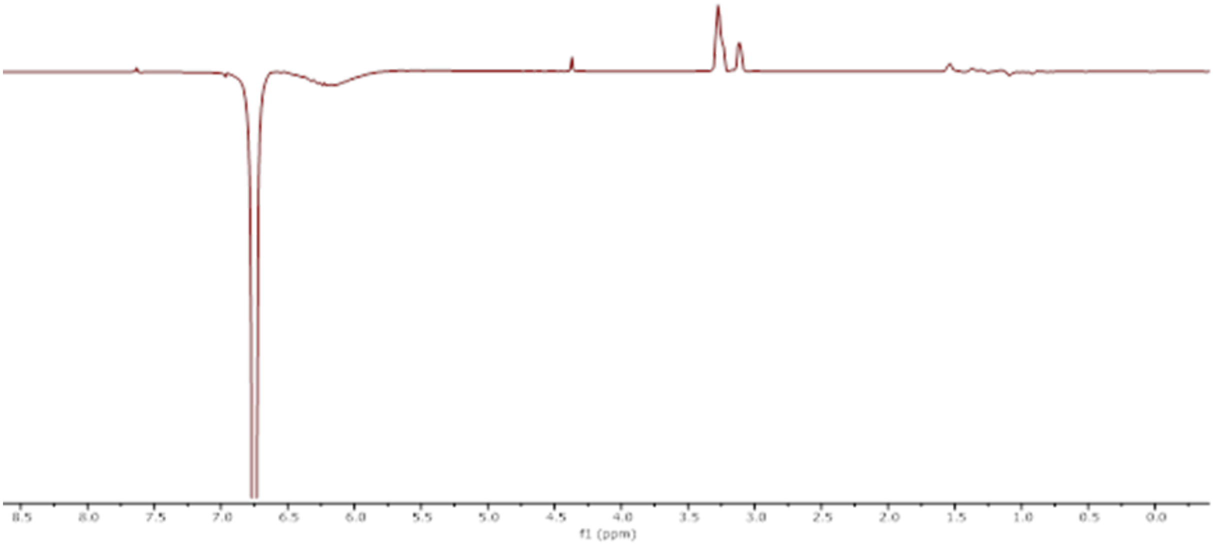

Supplementary Figure 19. ^{1}H NMR (400 MHz) and ^{13}C NMR (100 MHz) spectra of *N*-(*tert*Butylcarbamoyl)-*N*-benzyl-*N*'-(*tert*butoxycarbonyl)diethylenetriamine, **3e** (in $CDCl_3$)

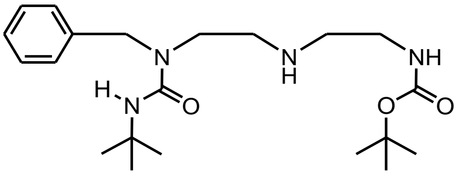

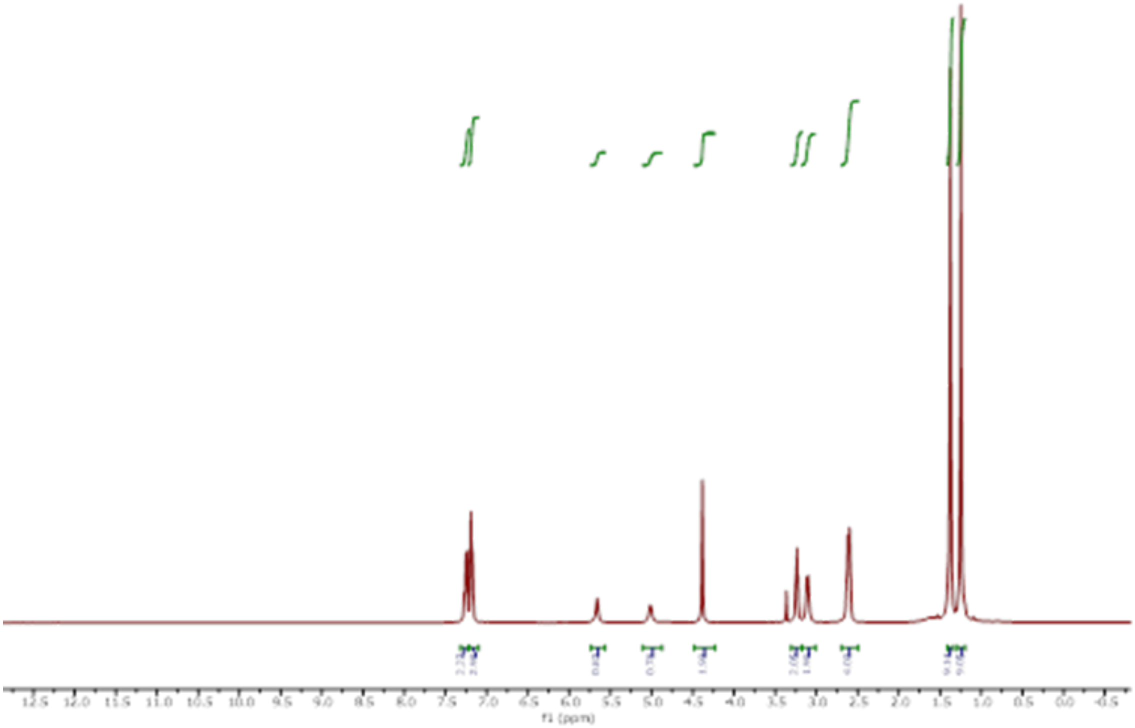

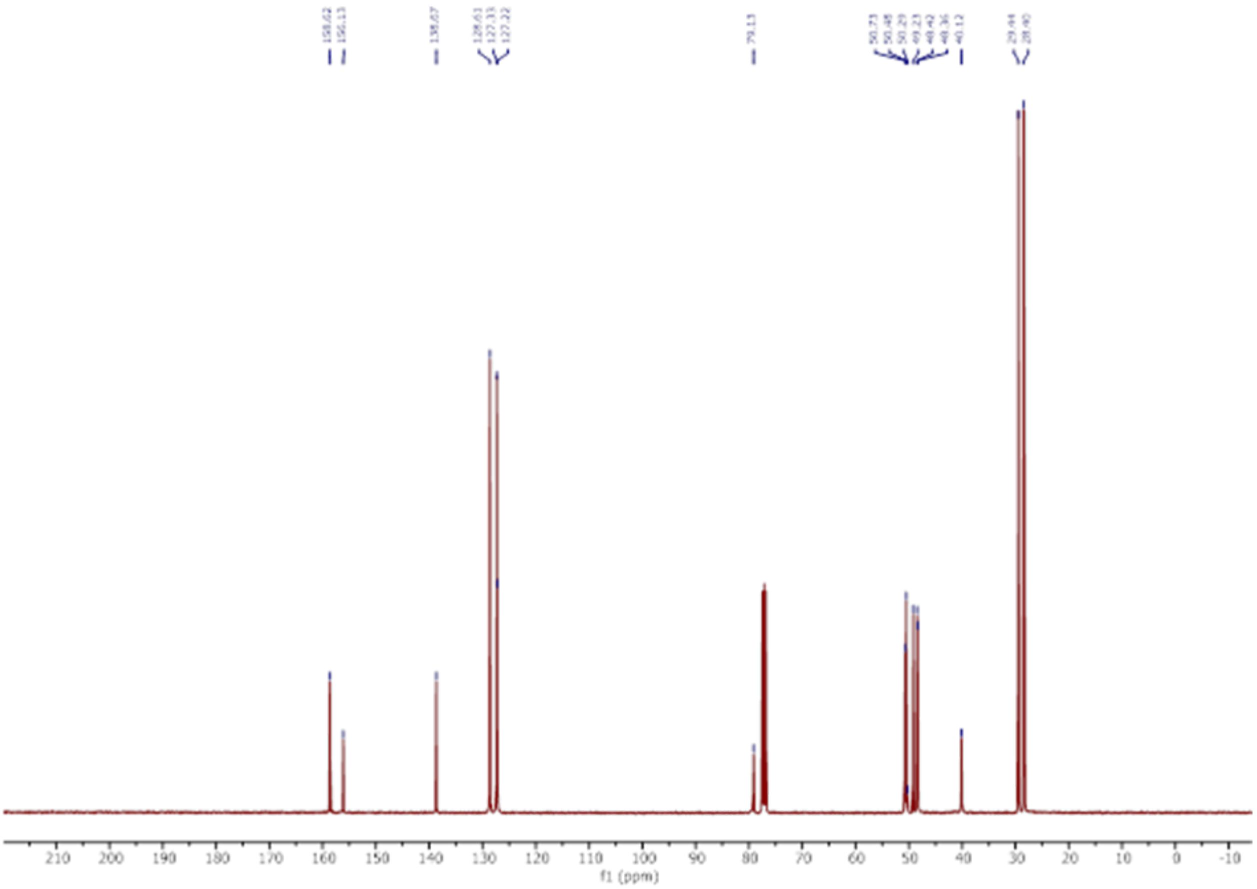
158.62
156.13
138.67
128.61
127.33
127.22
79.13
50.73
50.48
50.29
40.12
29.44
28.40
210 200 190 180 170 160 150 140 130 120 110 100 90 80 70 60 50 40 30 20 10 0 -10
f1 (ppm)

Supplementary Figure 20. ^{1}H NMR (400 MHz) and ^{13}C NMR (100 MHz) spectra of *N*-(*tert*Butylcarbamoyl)-*N*-benzyl-*N*'-(*tert*butoxycarbonyl)-*N*''-(n-hexylcarbamoyl)diethylenetriamine, **3f** (in $CDCl_3$)

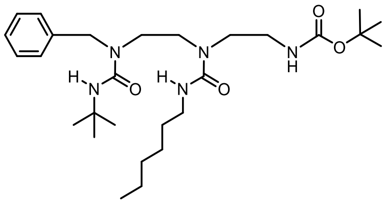

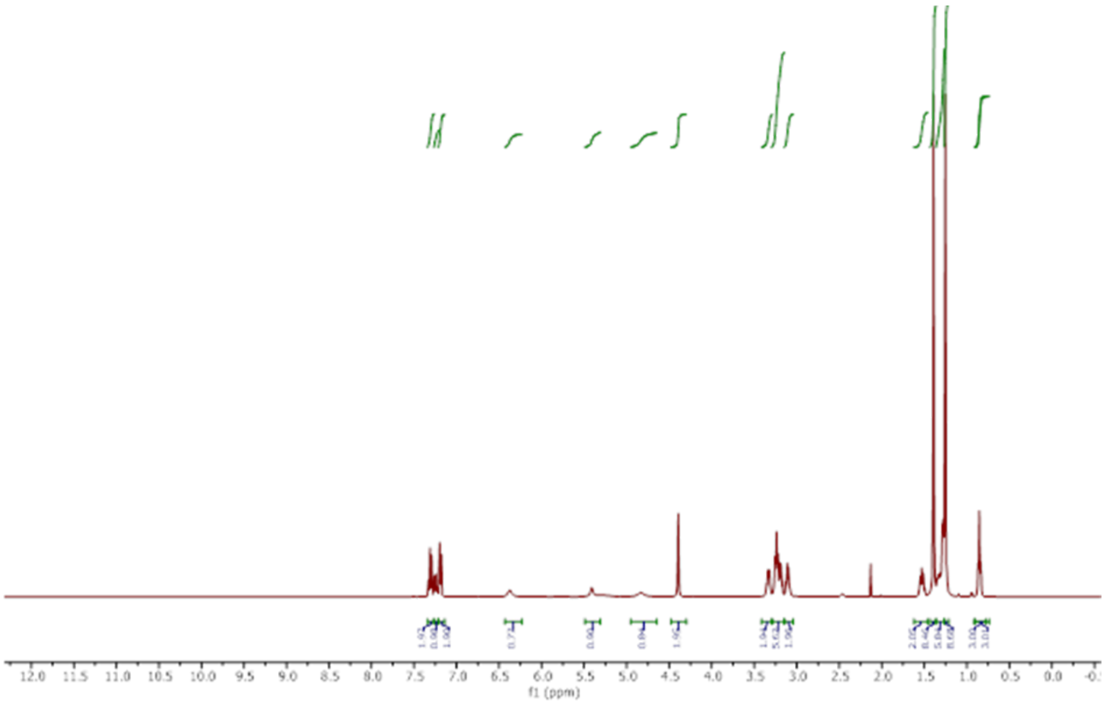

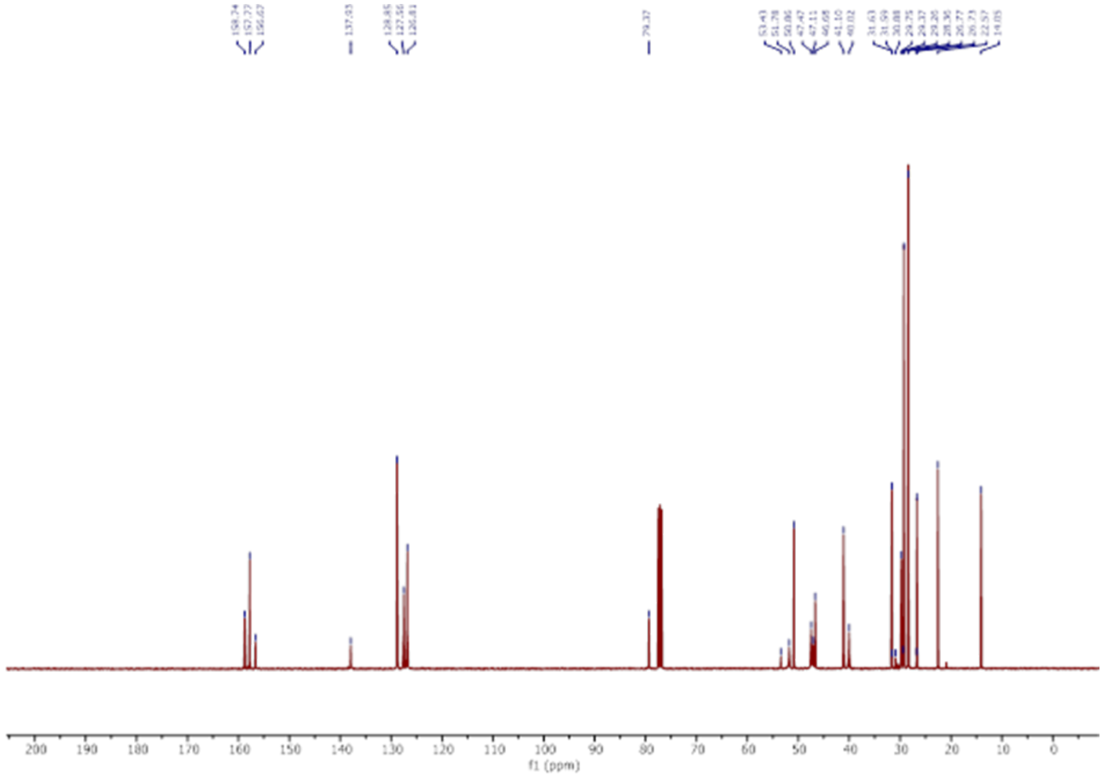

Supplementary Figure 21. ^{1}H NMR (400 MHz) and ^{13}C NMR (100 MHz) spectra of *N*-(*tert*Butylcarbamoyl)-*N*-benzyl-*N*''-(n-hexylcarbamoyl)diethylenetriamine, **3g** (in $CDCl_3$)

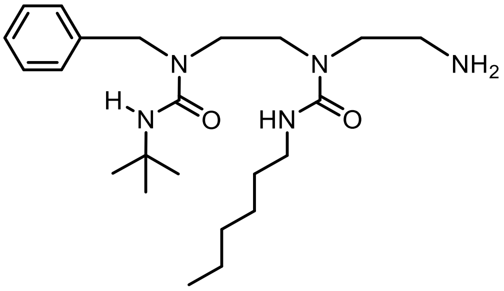

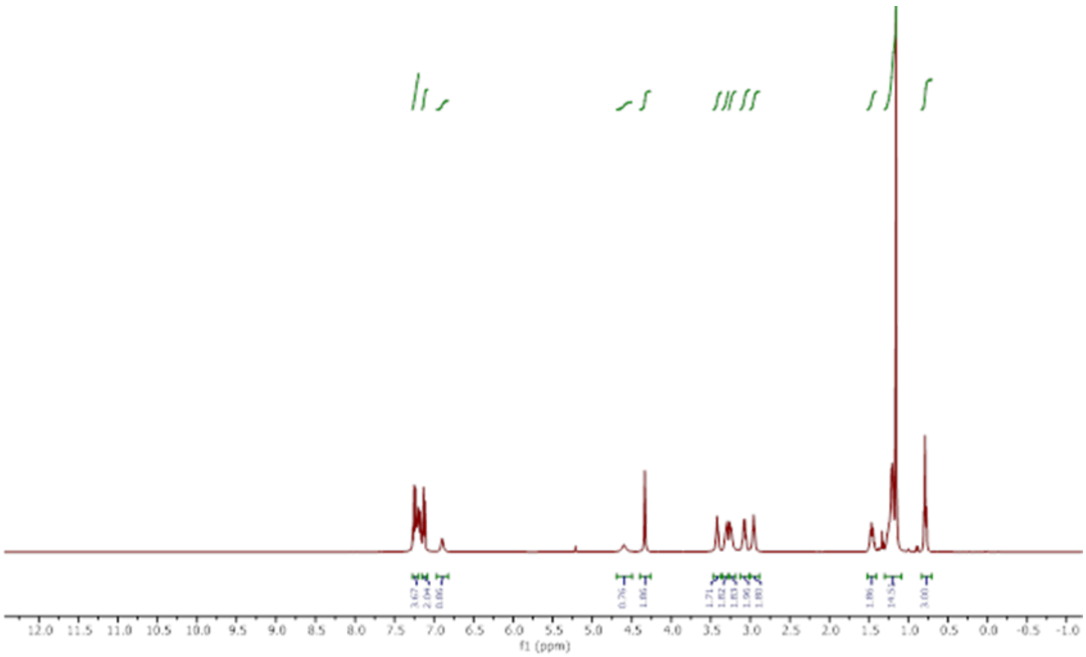

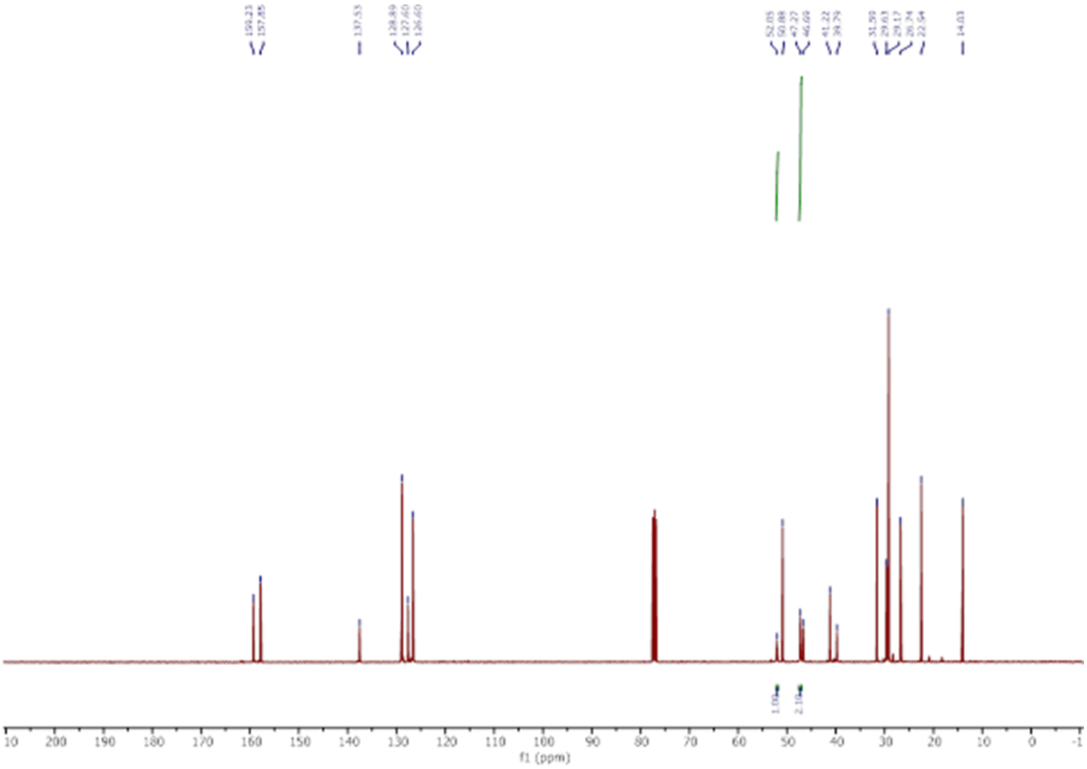

Supplementary Figure 22. ^{1}H NMR (400 MHz) and ^{13}C NMR (100 MHz) spectra of *N*-(*tert*Butylcarbamoyl)-*N*-benzyl-*N*''-(n-hexylcarbamoyl)-*N'*-(3,5-*bis*(trifluoromethyl)phenylcarbamoyl)diethylenetriamine, **3** (in $CDCl_3$)

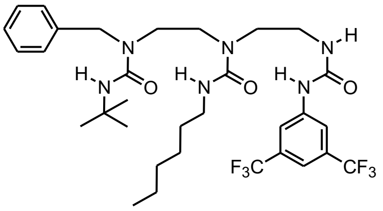

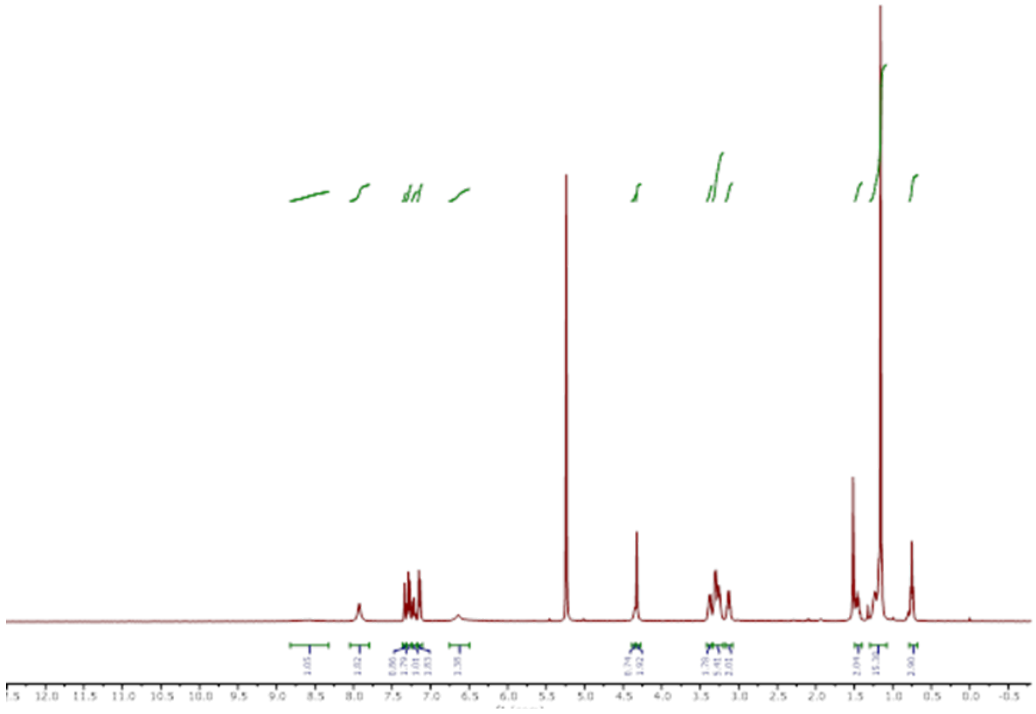

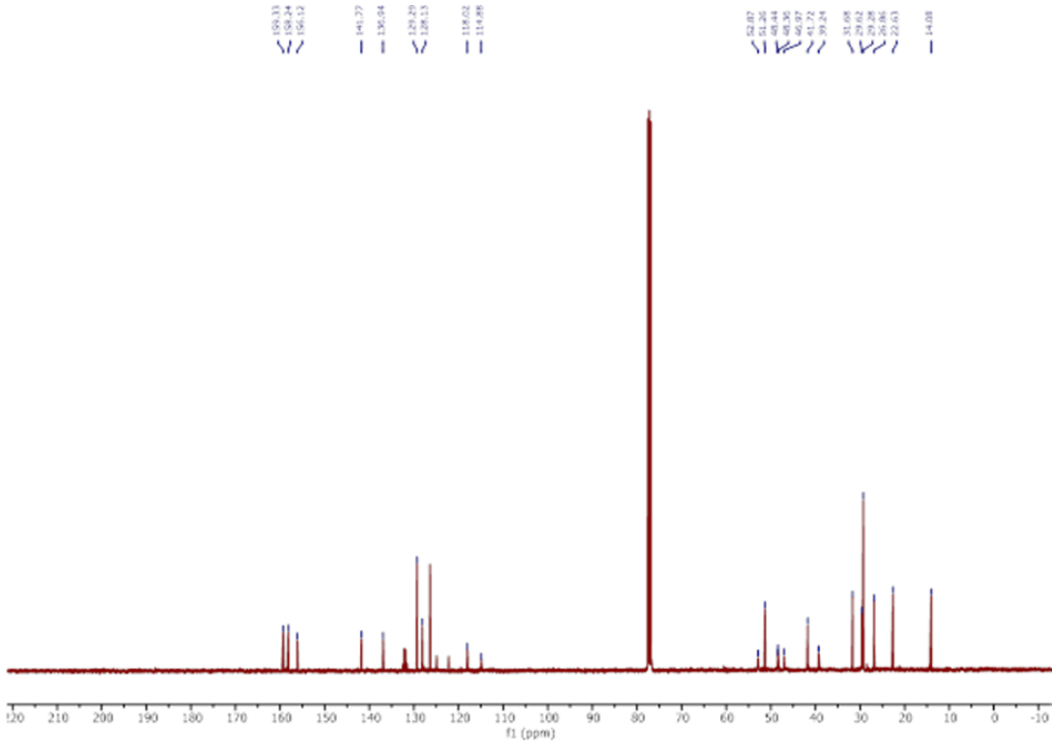

Supplementary Figure 23. ^{1}H NMR (400 MHz in $CDCl_3$), ^{1}H NMR (400 MHz in CD_2Cl_2), and ^{13}C NMR (100 MHz in CD_2Cl_2) spectra of *N,N′*-Diethyl- *N,N′*-bis(n-butylcarbamoyl)ethylenediamine, **SI5**

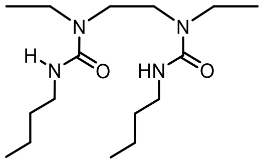

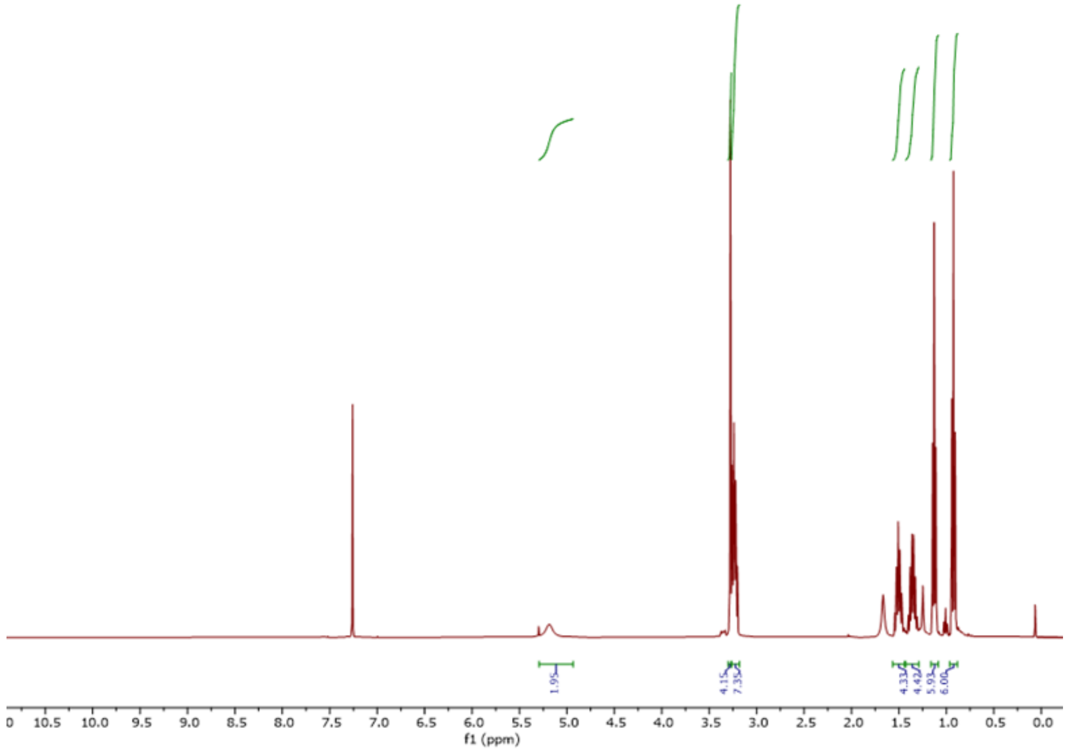

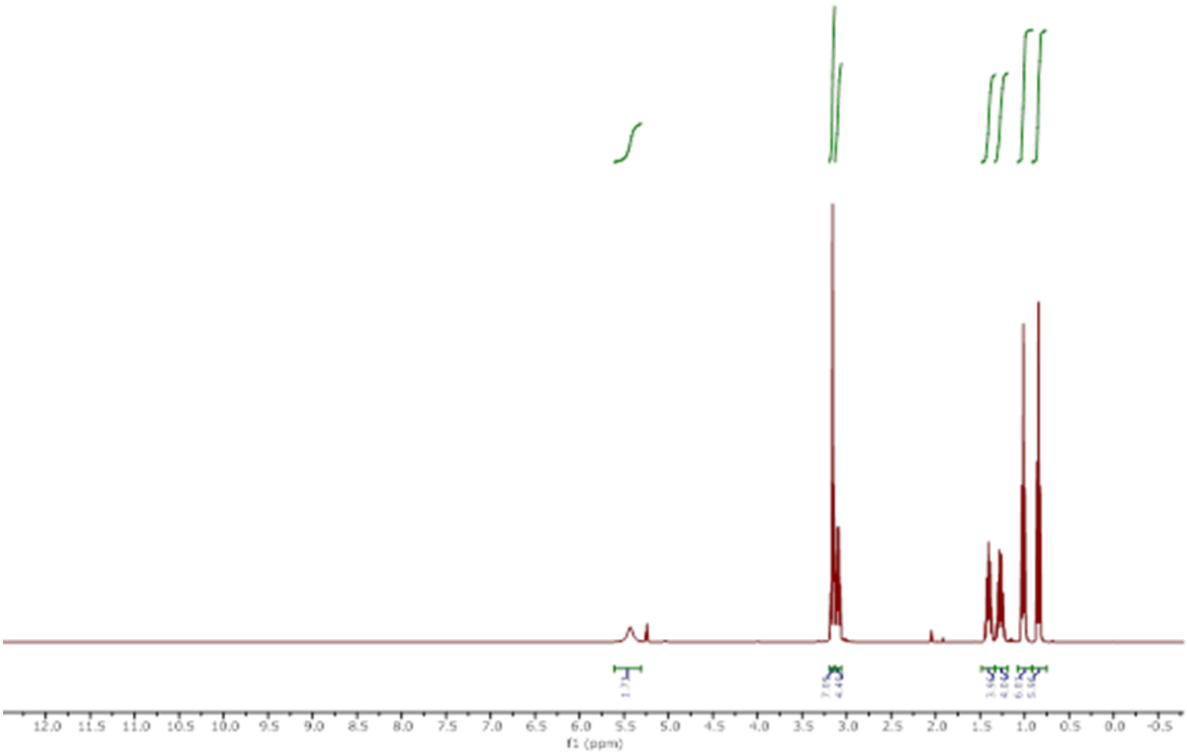
12.0 11.5 11.0 10.5 10.0 9.5 9.0 8.5 8.0 7.5 7.0 6.5 6.0 5.5 5.0 4.5 4.0 3.5 3.0 2.5 2.0 1.5 1.0 0.5 0.0 -0.5
f1 (ppm)

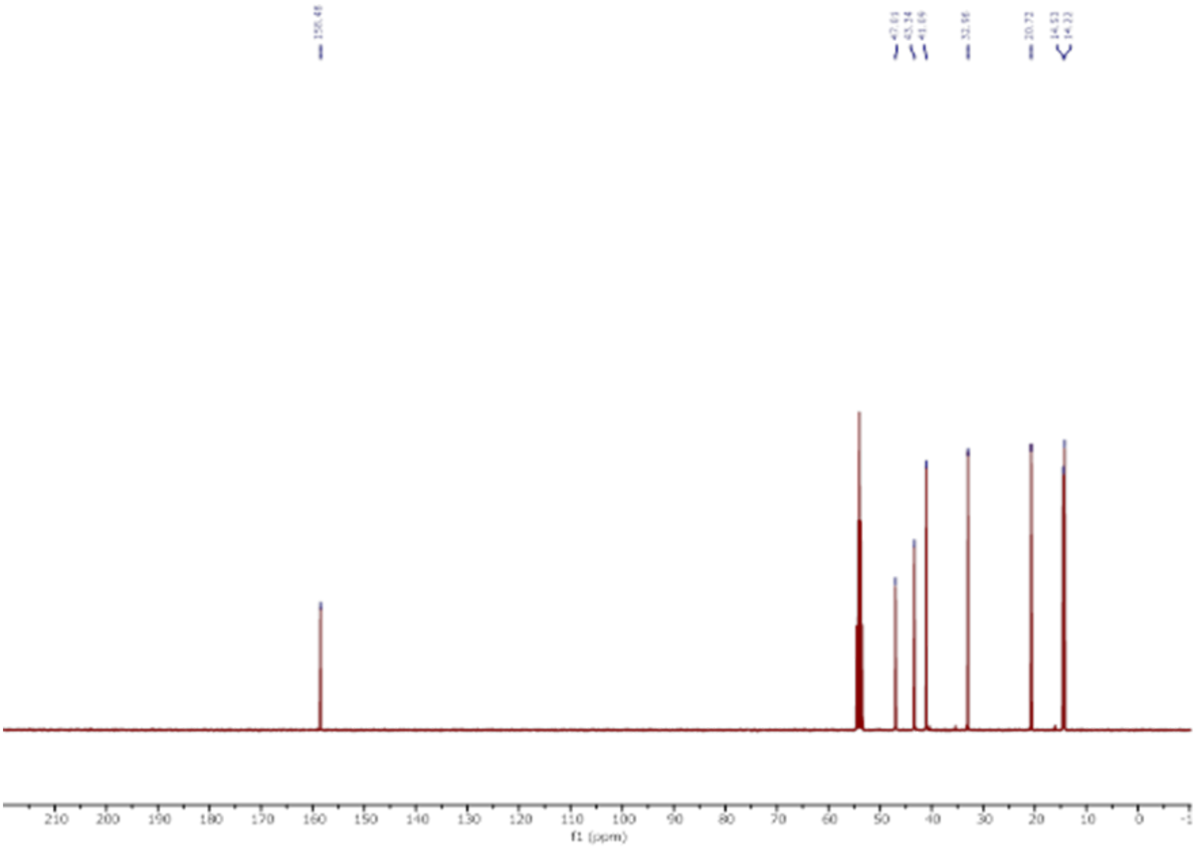
158.48
32.96
20.72
14.53
14.22
210 200 190 180 170 160 150 140 130 120 110 100 90 80 70 60 50 40 30 20 10 0
f1 (ppm)

Supplementary Figure 24. ^{1}H NMR (400 MHz) and ^{13}C NMR (100 MHz) spectra of 1-n-Butyl-3-(4-(trifluoromethyl)phenyl)urea **SI7**

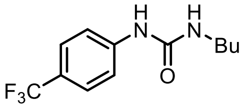

In CD_2Cl_2

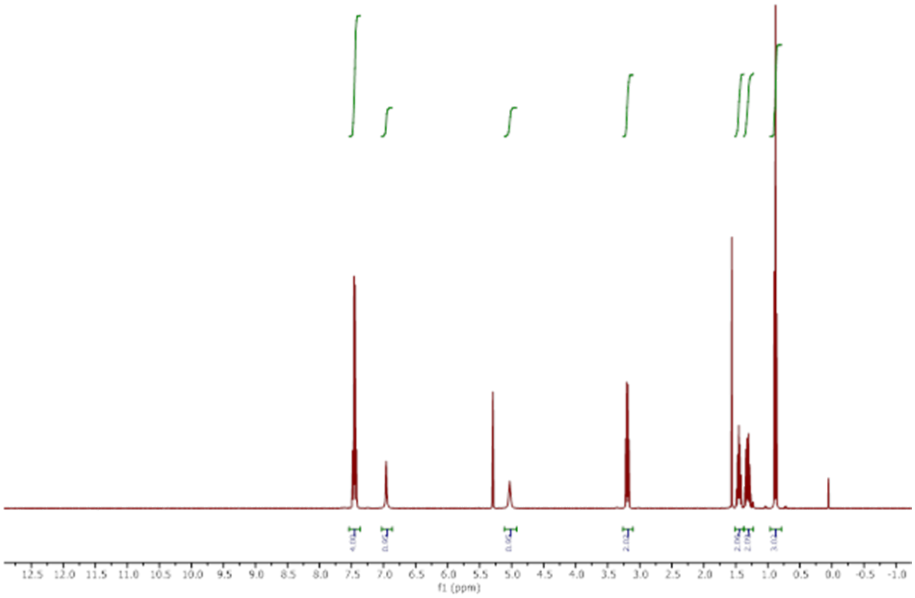

In $CDCl_3$

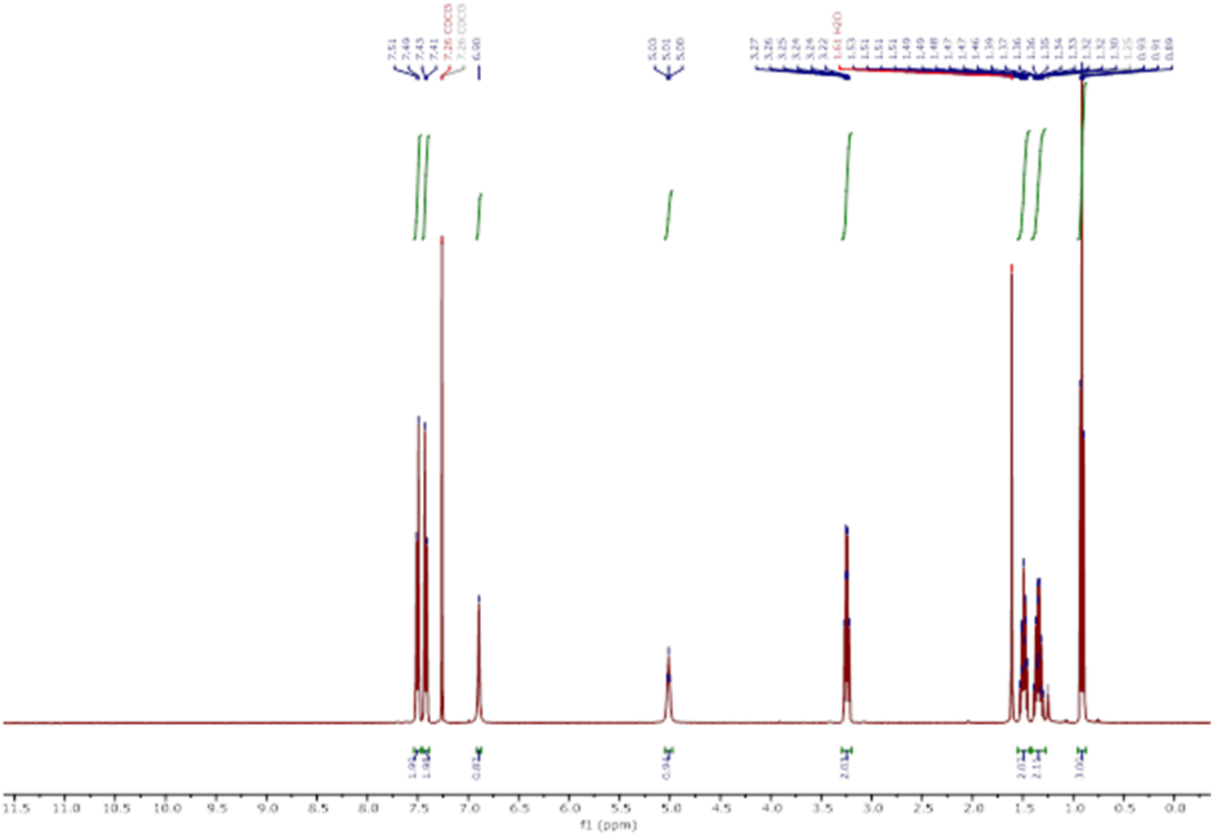

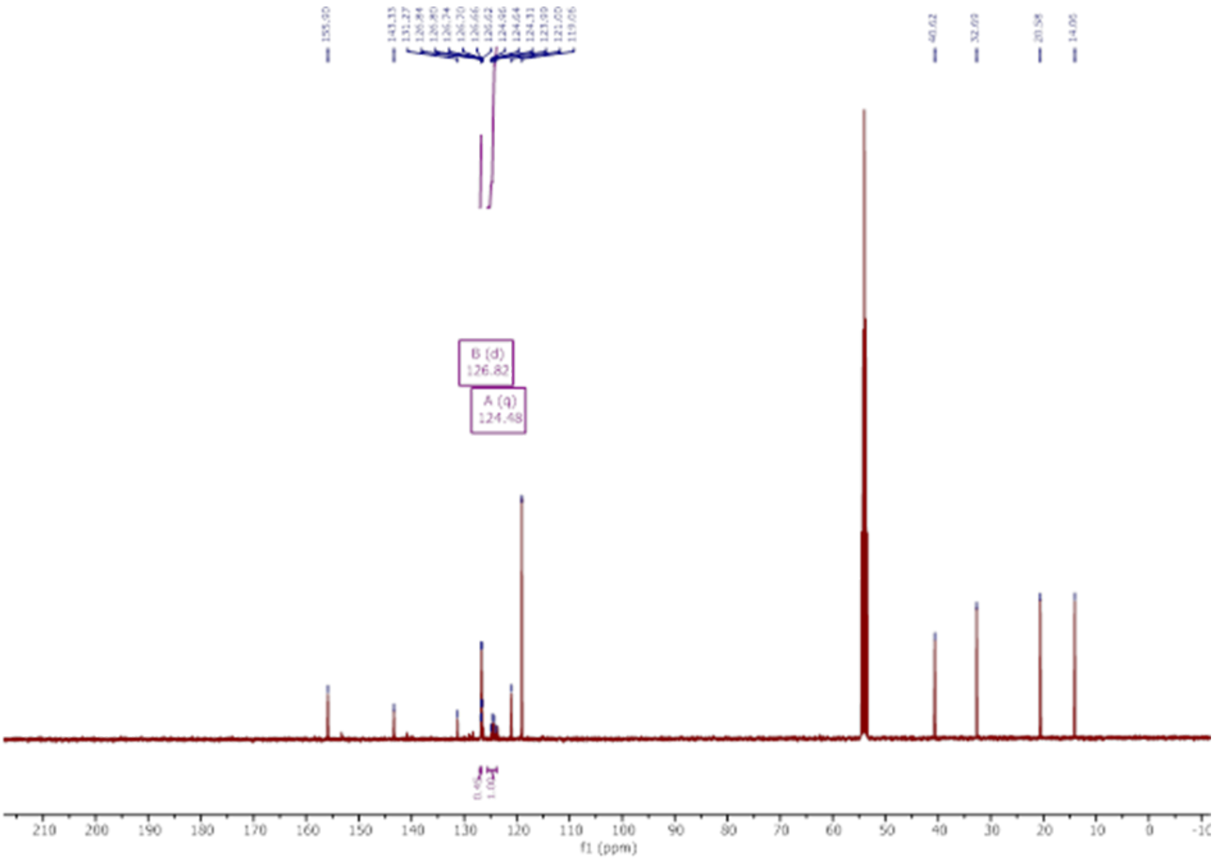
B (d)
126.82
A (q)
124.48
f1 (ppm)

IV. Supplementary Discussion

5. NMR spectroscopy studies

5.1. Compound 1

5.1.1. Assignment of ^{1}H and ^{13}C signals

^{1}H and ^{13}C NMR signals of compound **1** were assigned based on COSY, HSQC, HMBC and 2D NOESY experiments in CD_2Cl_2. ^{1}H ^{1}H 2D NOESY NMR experiment (supplementary figure 29) shows correlation peaks between CH_2^{10} and both NH^5 and H^8, a weak correlation peak between CH_2^{10} and NH^4 is also present while no correlation peak with NH^3 is visible; correlation peaks between NH^1 and NH^2 and between H^7 and NH^1 allow for the attribution of those signals. ^{1}H ^{1}H COSY spectrum confirms the coupling between NH^5, NH^4, NH^3 with CH_{2Bu} signals at 3.47 – 2.95 ppm.

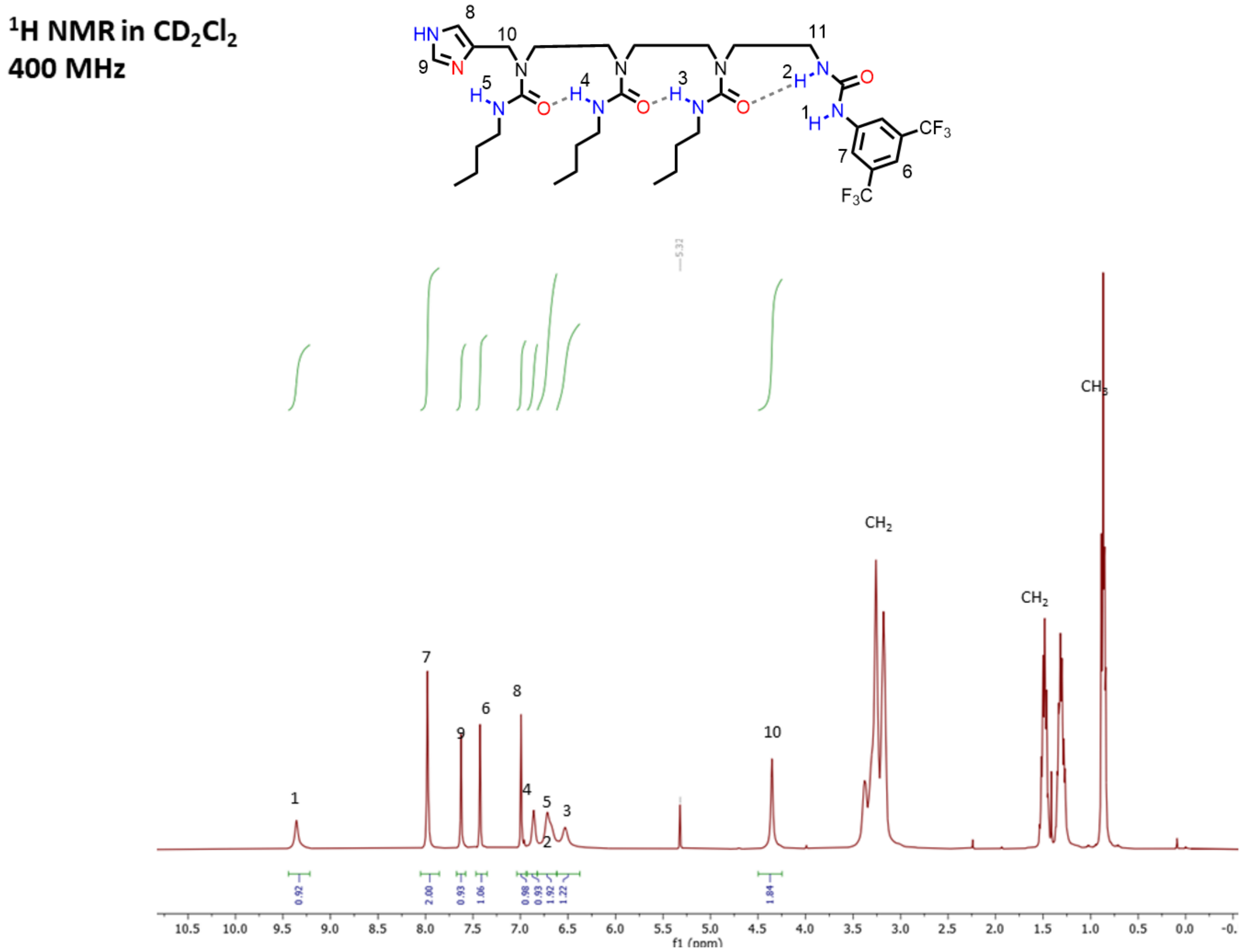

Supplementary Figure 25. ^{1}H NMR spectrum of **1** in CD_2Cl_2 at 25 °C (400 MHz).

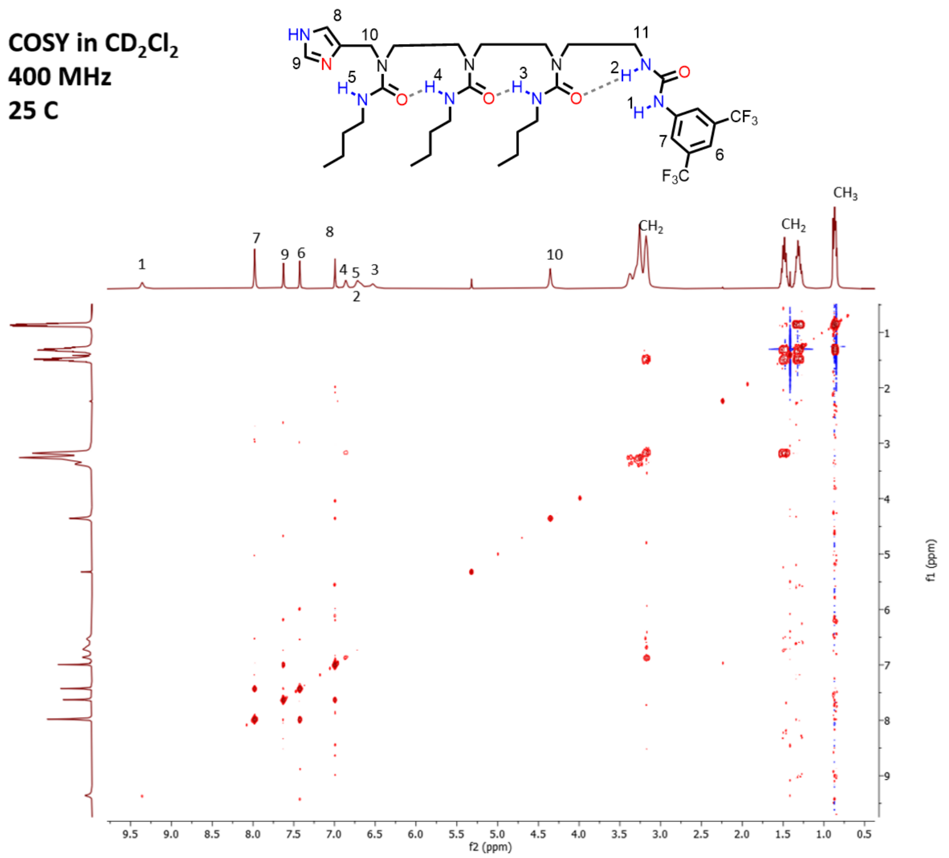

Supplementary Figure 26. ^{1}H COSY NMR spectrum of **1** in CD_2Cl_2 at 25 °C (400 MHz).

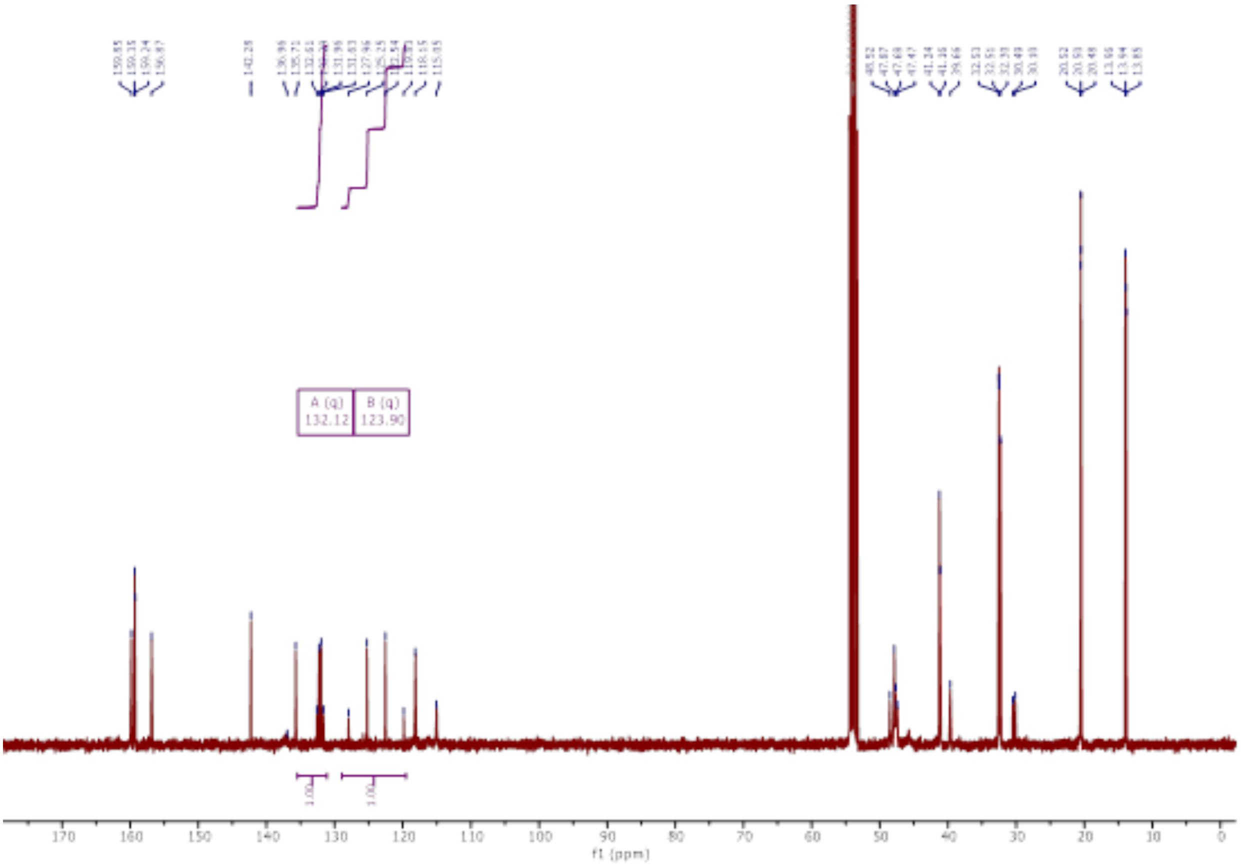

Supplementary Figure 27. ^{13}C NMR spectrum of **1** in CD_2Cl_2 at 25 °C (400 MHz).

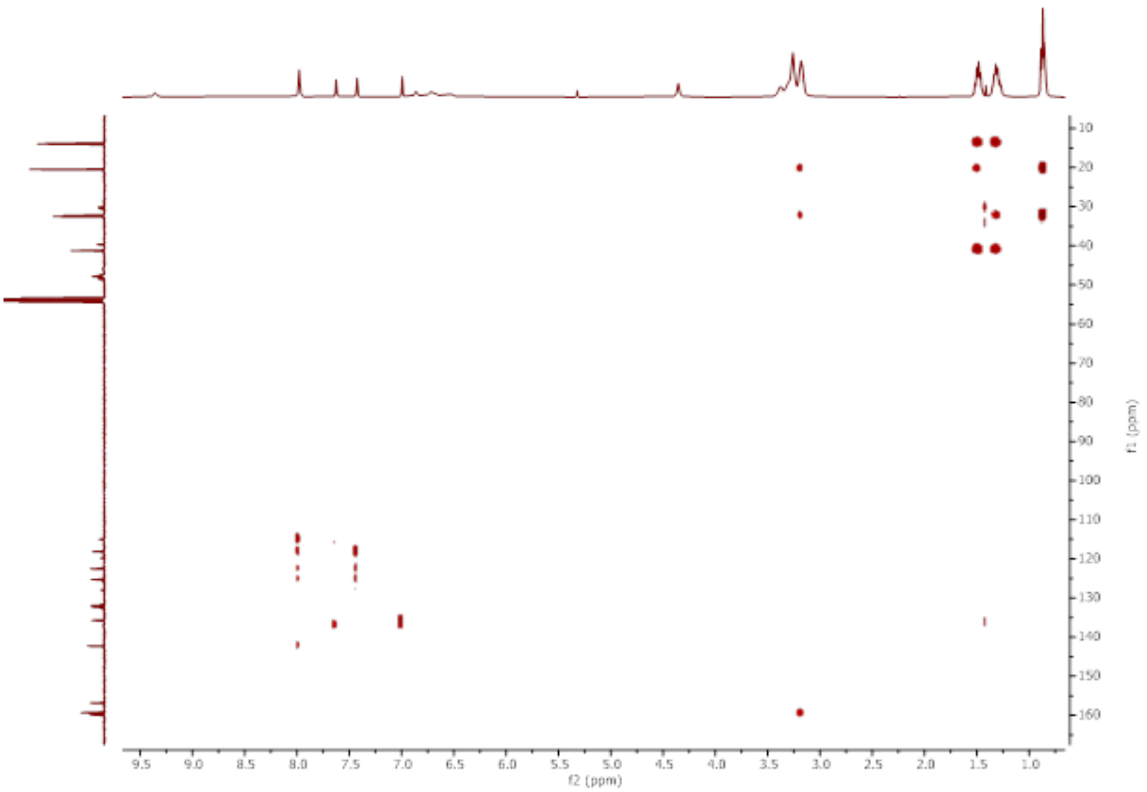

Supplementary Figure 28. ^{1}H ^{13}C HMBC spectrum of **1** in CD_2Cl_2 at 25 °C (400 MHz).

A)

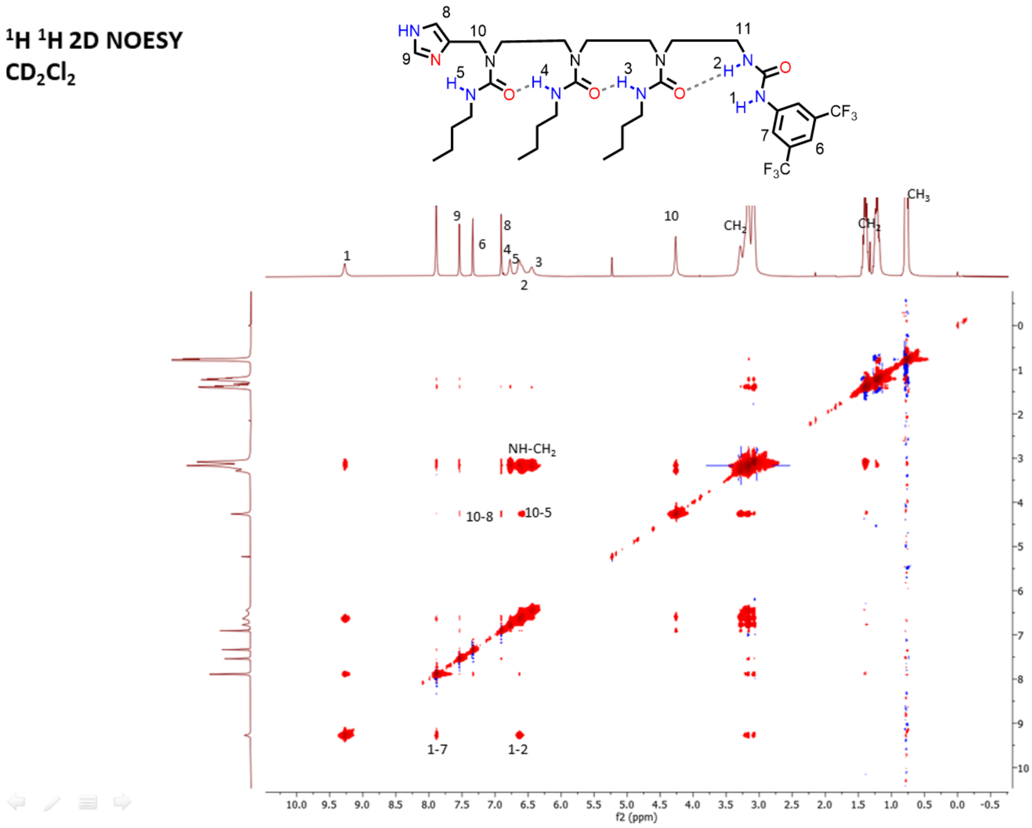

B)

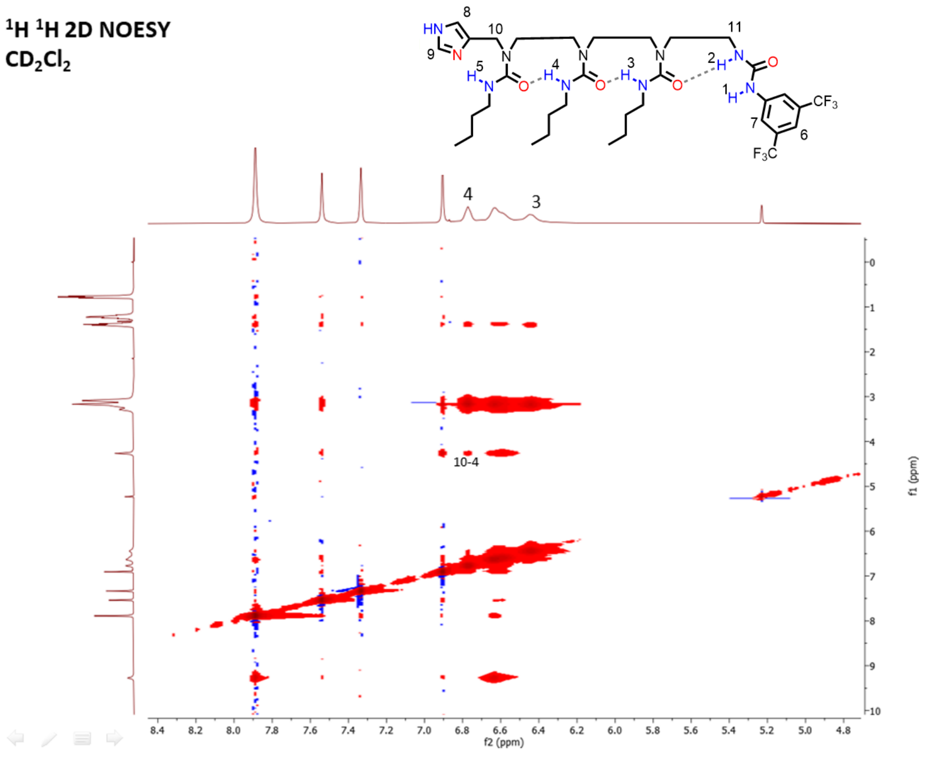

Supplementary Figure 29. 2D NOESY ^{1}H spectra of **1** in CD_2Cl_2 at 25 °C (400 MHz) (A) full spectrum and B) expansion at 4.8-.4 ppm).

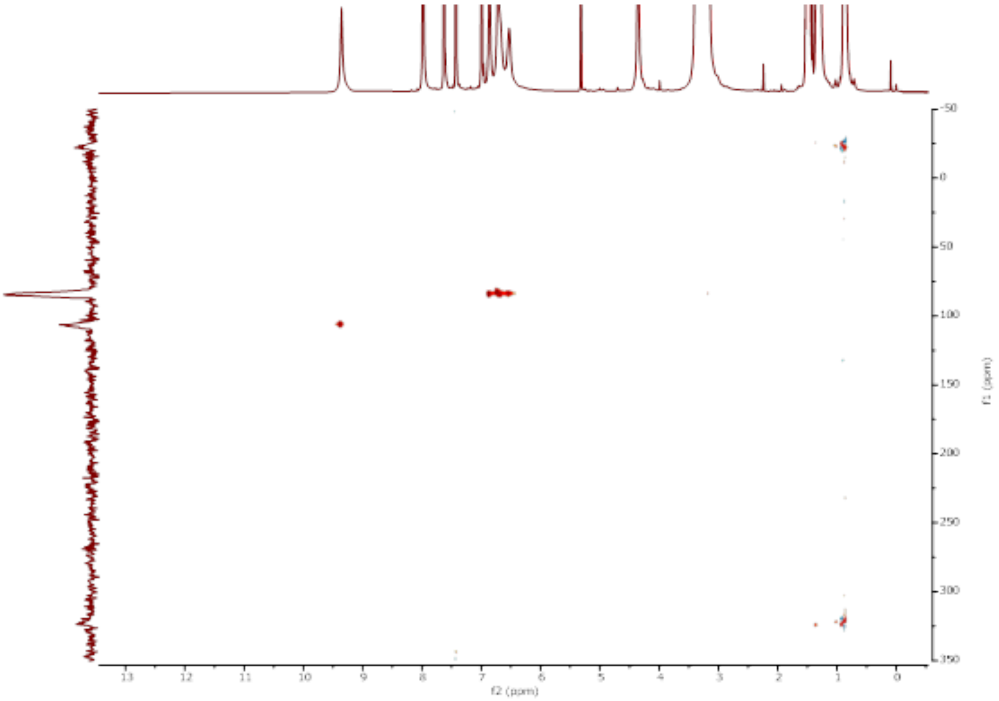

Supplementary Figure 30. ^{1}H ^{15}N HSQCETGP spectrum of **1** in CD_2Cl_2 at 25 °C (400 MHz).

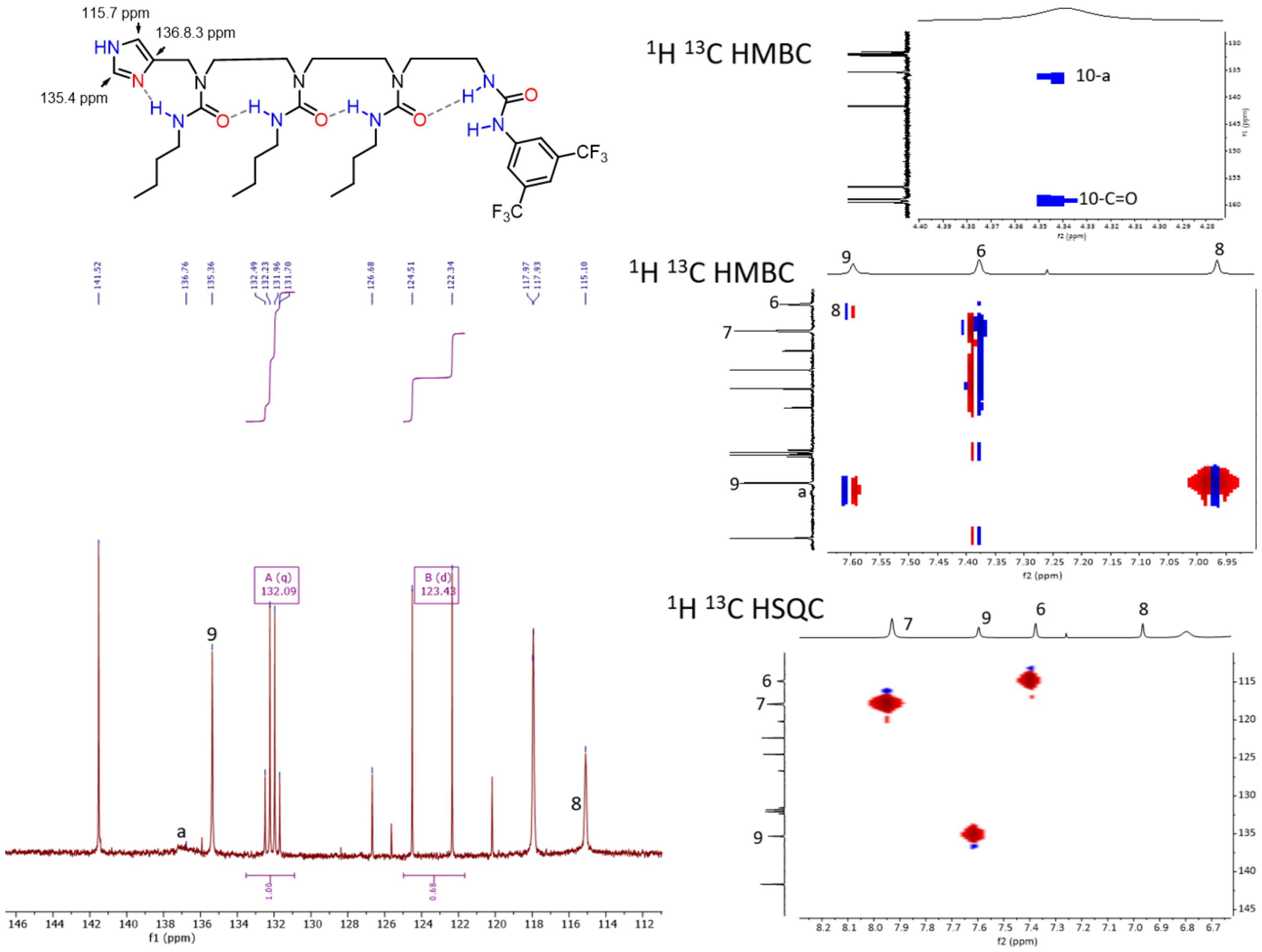

Supplementary Figure 31. Assignment of ^{13}C NMR signals of the imidazole moiety of **1** in $CDCl_3$

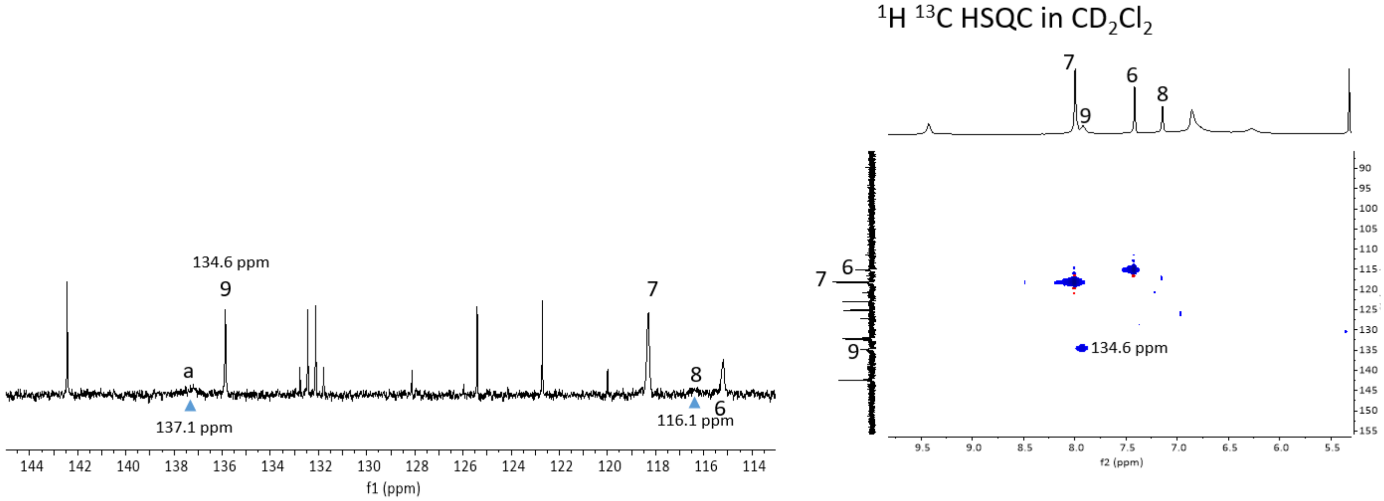

Supplementary Figure 32. Assignment of ^{13}C NMR signals of the imidazole moiety of **1** in CD_2Cl_2

5.1.2. Dilution study of compound **1** in CD_2Cl_2 at 25 °C

A dilution study of compound **1** in CD_2Cl_2 at 25 °C was done by recording ^1H NMR spectra (400 MHz) at concentrations between 7.4 mM and 118.2 mM (Supplementary Figure 33), the variations of chemical shifts for the NH signals are presented in Supplementary Table 1. Their variations is attributed to a low degree of self association in CD_2Cl_2 (K_{assoc} = 31 ± 2 M^{-1}) (Supplementary Figure 34).

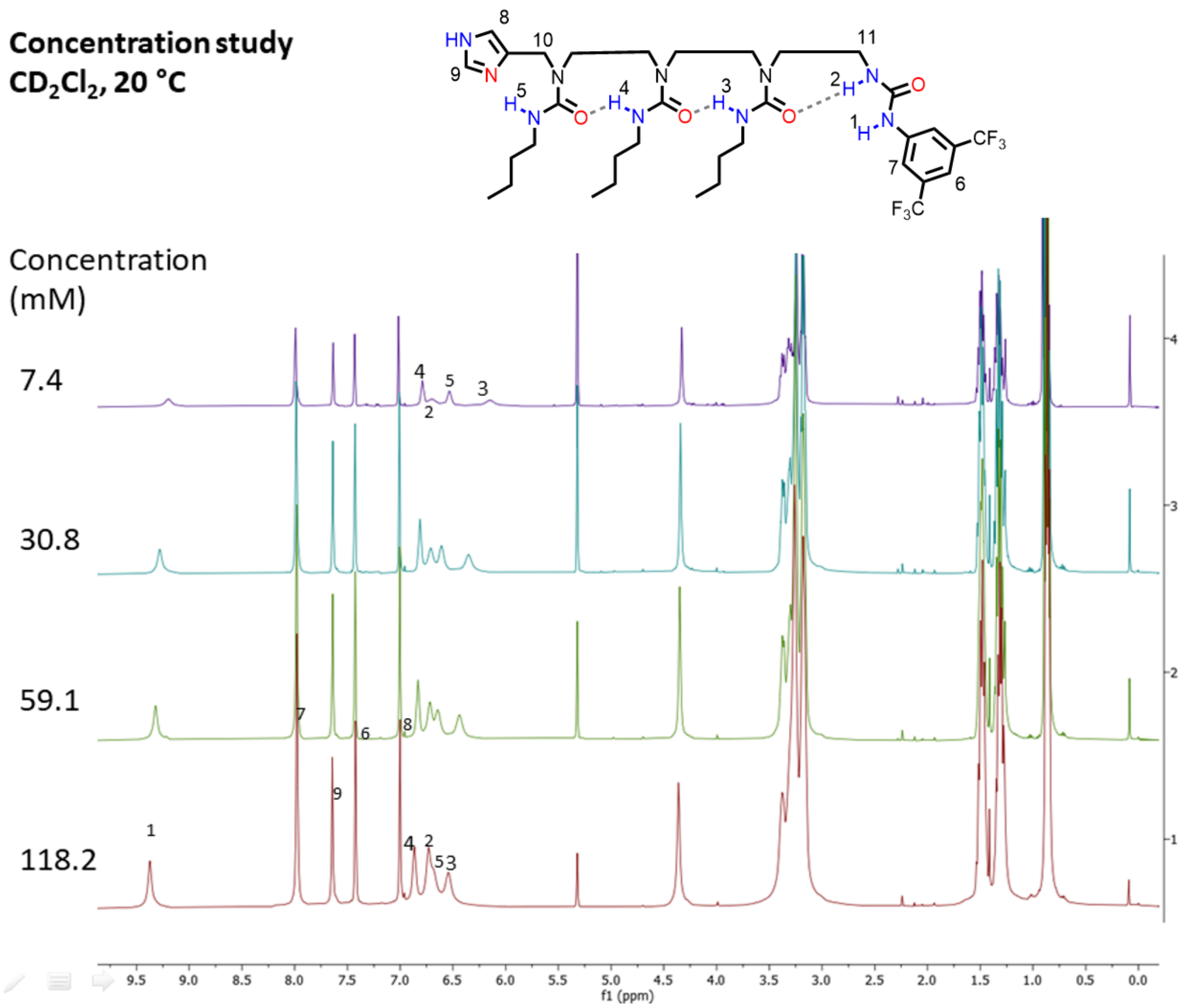

Supplementary Figure 33. Overlay of ^{1}H NMR spectra of compound **1** in CD_2Cl_2 at 25 °C (400 MHz spectrometer) at concentrations between 7.4 mM to 118.2 mM

Supplementary Table 1. ^{1}H NMR chemical shifts (ppm) of NH groups of compound **1** in CD_2Cl_2 at 25 °C (400 MHz spectrometer) at concentrations between 7.4 mM to 118.2 mM

Concentration (mM)	NH^1 (ppm)	NH^2 (ppm)	NH^5 (ppm)	NH^4 (ppm)	NH^3 (ppm)
**7.4**	9.19	6.69	6.53	6.79	6.15
**30.8**	9.28	6.70	6.61	6.81	6.35
**59.1**	9.31	6.71	6.64	6.83	6.43
**118.2**	9.37	6.73	6.68	6.86	6.54
**CIS**	0.18	0.04	0.15	0.07	0.39

**Time to fit**	0.1841 s
**SSR**	6.3896e-4
**Fitted datapoints**	20
**Fitted params**	11

Parameters

Parameter (bounds)	Optimised	Error	Initial
K_e (0 → ∞) (Kd)	31.07 M^{-1} 15.54 M^{-1}	± 6.3152 % ± 3.1576 %	100.00 M^{-1}

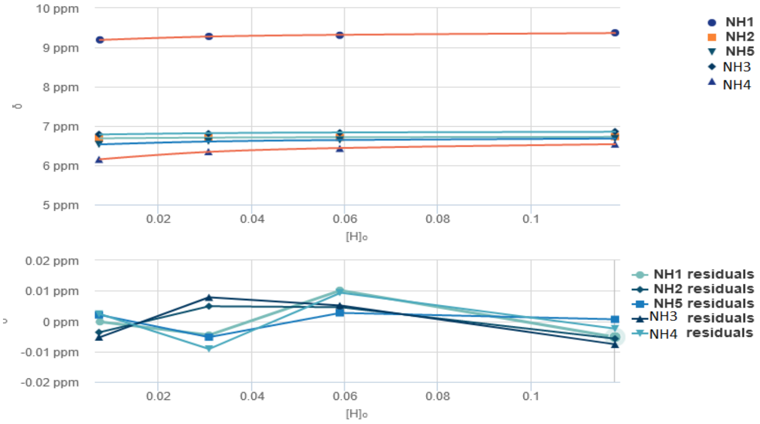

Supplementary Figure 34. Non-linear curve fitting analysis of the experimental concentration study of compound **1** (between 7.4 mM and 118.2 mM in CD_2Cl_2) using a theoretical binding isotherm for dimer aggregation Source data are provided as a Source Data file. http://app.supramolecular.org/bindfit/view/91aa69ef-2ea6-4247-90ed-f6c183fa5894

5.1.3. Variable temperature ^{1}H NMR study (25 to -40 °C)

^{1}H NMR spectra of compound **1** (18 mM in CD_2Cl_2) were recorded between 25 °C and -40 °C (Supplementary Figure 35). The compound precipitates out of solution from -20 °C (observed by eye). At -20 °C, several signals split (NH^1, H^{10}) while all NH signals shift downfield (CIS 0.3 ppm for all NH signals). Self-aggregation possibly causes the observations.

a)

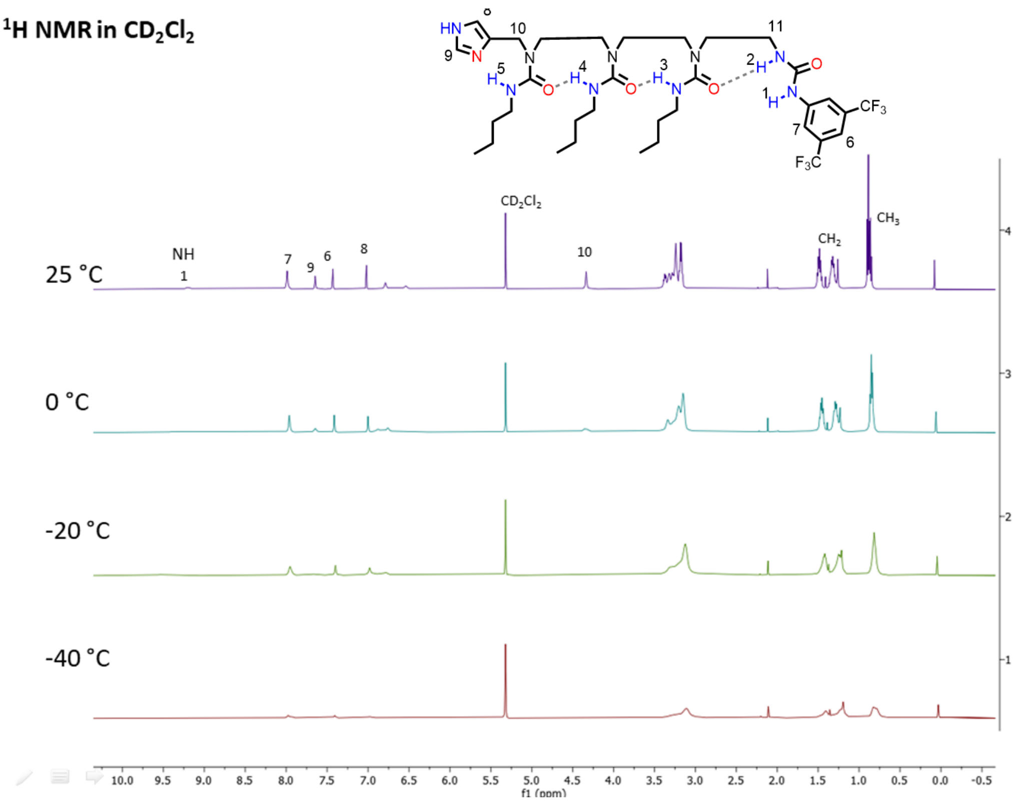

b)

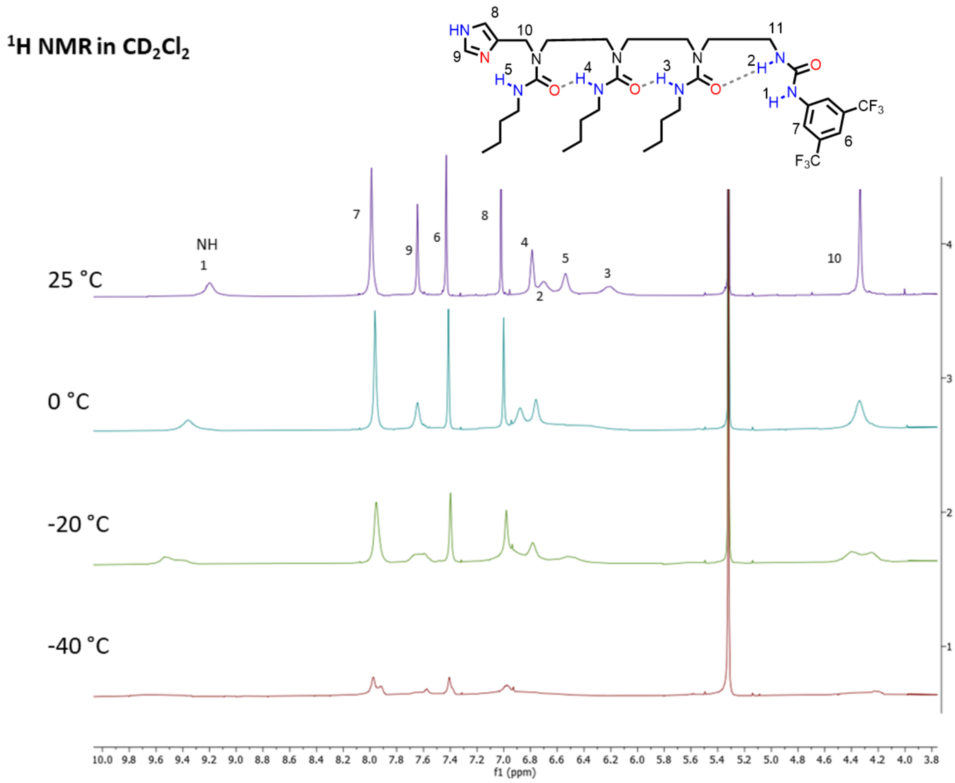

Supplementary Figure 35. Overlay of ^1H NMR spectra (500 MHz) of compound **1** (18 mM in CD_2Cl_2) recorded between 25 °C and -40 °C (a) full spectra and b) expansion of the 4ppm-10ppm region).

5.1.4. Titration of compound **1** with tetrabutylammonium chloride in CD_2Cl_2 at 25 °C

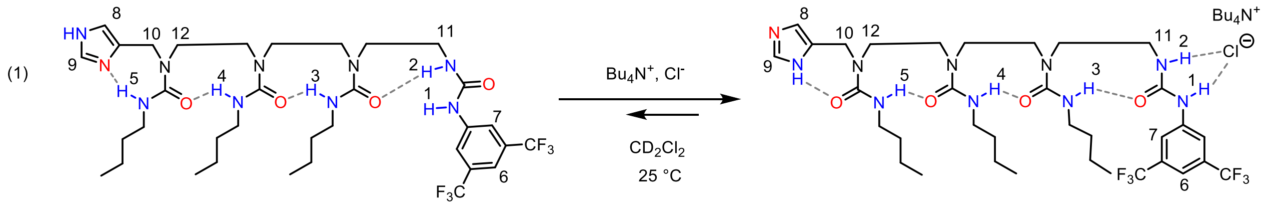

Titration of compound **1** (18 mM) in CD_2Cl_2 at 25 °C with increasing amounts of tetrabutylammonium chloride (from 0 to 2 equivalents) was followed by ^{1}H NMR (Supplementary Figure 36). The values of chemical shifts of the NH of compound **1** upon addition of ligand were collected in Supplementary Table **2** and plotted in a graph chemical shift of NH = f(ligand added) (Supplementary Figure 37). Progressive downfield shifts of ^{1}H NMR signals for both NH^1 (CIS 0.99 ppm) and NH^2 (CIS 0.92 ppm) are observed upon addition of ligand until 1 equivalent of ligand is added, the chemical shifts do not vary much upon further addition of ligand. This is coherent with the regioselective formation of intermolecular hydrogen bonds between the ligand and the host at NH^1 and NH^2.

2D NOESY NMR analysis on compound **1** in the presence of 2 equivalents of chloride ligand (Supplementary Figure 40) shows NOE correlation between NH^5 and CH_2^{10} allowing the unambiguous assignment of ^{1}H NMR signal for NH^5. NOE correlation between NH^5 and adjacent ethylene CH_2^{12} is stronger, indicating a preferred directionality in the presence of ligand, opposite to the one observed in the absence of ligand (compare with supplementary figure 29). The value of chemical shift of NH^5 (6.51 ppm) before addition of ligand is diagnostic of its hydrogen bonded state, which also indicates the tautomer form of imidazole in the native conformation. During the titration, the ^{1}H NMR signal for NH^5 initially shifts downfield (0.1 ppm) until 0.5 equivalents of ligand then upfield (-0.1 ppm) until 1 equivalent of ligand is added, the signal does not shift upon further addition of ligand to 2 equivalents; NH^5 stays in hydrogen bonding upon addition of ligand, binds the carbonyl of the adjacent urea instead of the imidazole upon directionality switch of the hydrogen bond chain. The ^{1}H NMR signals for NH^4 (CIS -0.13 ppm) and NH^3 (CIS 0.2 ppm) indicate they stay in hydrogen bonding during the titration.

An association constant for the binding event of chloride ligand was calculated by non-linear curve fitting analysis of the titration curves with theoretical binding isotherms for 1:1, 1:2, 2:1 binding modes (Supplementary Figures 38, 39). The best fit were obtained for a 1:1 binding mode and a 2:1 binding mode, in the former case with $K = 1600 \pm 600\ M^{-1}$, and in the latter case, $K_{1:1} = 1200 \pm 200\ M^{-1}$, $K_{1:2} = 62 \pm 7\ M^{-1}$.

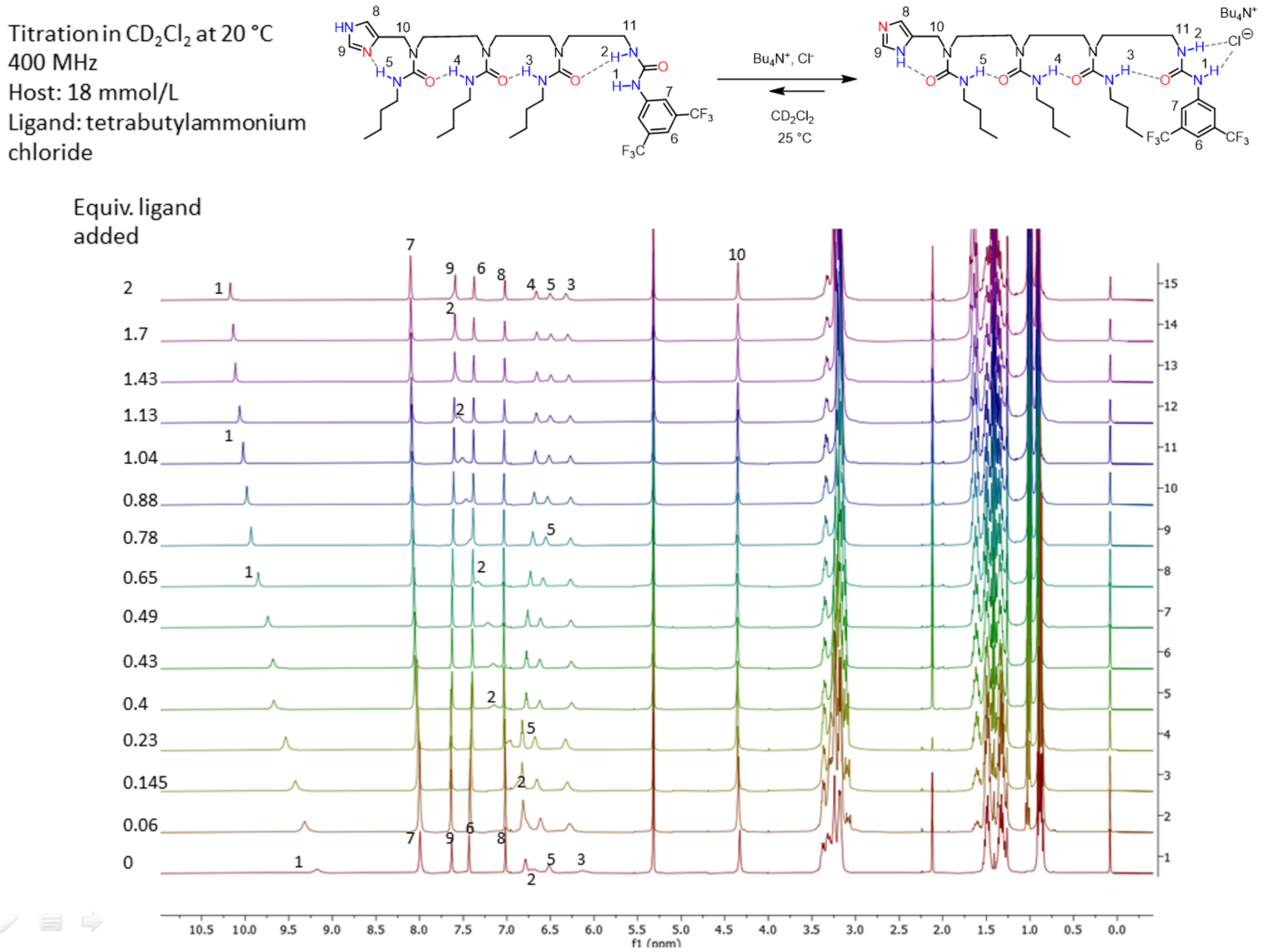

Supplementary Figure 36. Overlay of ^{1}H NMR spectra of compound **1** (18 mM in CD_2Cl_2) in the presence of increasing amounts of tetrabutylammonium chloride from 0 to 2 equivalents, recorded at 25 °C (400 MHz).

Supplementary Table 2. Chemical shifts of ^1H NMR signals of compound **1** recorded during the NMR titration of **1** (18 mM in CD_2Cl_2) with increments of tetrabutylammonium chloride at 25 °C (400 MHz). $NH_{imidazole}$ not visible.

Concentration of host (mol/L)	Concentration of chloride ligand (mol/L)	Equiv. ligand	Chemical shift NH^1 (ppm)	Chemical shift NH^2 (ppm)	Chemical shift NH^5 (ppm)
0.018	0	0	9.18	6.68	6.51
0.018	0.001125	0.063	9.27	6.78	6.55
0.018	0.00261	0.145	9.39	6.86	6.56
0.018	0.004194	0.23	9.52	6.97	6.60
0.018	0.0072	0.4	9.67	7.14	6.62
0.018	0.00774	0.43	9.68	7.16	6.62
0.018	0.00882	0.49	9.74	7.215	6.61
0.018	0.011628	0.65	9.85	7.32	6.583
0.018	0.01404	0.78	9.93	7.41	6.55
0.018	0.015894	0.88	9.98	7.464	6.53
0.018	0.01872	1.04	10.02	7.51	6.516
0.018	0.02034	1.13	10.06	7.55	6.5
0.018	0.02574	1.43	10.11	7.59	6.495
0.018	0.0306	1.7	10.14	7.59	6.495
0.018	0.03654	2	10.17	7.6	6.5
	**CIS (ppm)**		**0.99**	**0.92**	**0.11**

Concentration of host (mol/L)	Concentration of chloride ligand (mol/L)	Equiv. ligand	Chemical shift NH^4 (ppm)	Chemical shift NH^3 (ppm)	Chemical shift H^7
0.018	0	0	6.784	6.12	7.99
0.018	0.001125	0.063	6.80	6.17	
0.018	0.00261	0.145	6.81	6.22	
0.018	0.004194	0.23	6.81	6.23	
0.018	0.0072	0.4	6.775	6.257	
0.018	0.00774	0.43	6.775	6.26	
0.018	0.00882	0.49	6.76	6.265	
0.018	0.011628	0.65	6.73	6.27	
0.018	0.01404	0.78	6.7	6.27	
0.018	0.015894	0.88	6.68	6.27	
0.018	0.01872	1.04	6.67	6.27	
0.018	0.02034	1.13	6.66	6.27	
0.018	0.02574	1.43	6.656	6.29	
0.018	0.0306	1.7	6.65	6.3	
0.018	0.03654	2	6.66	6.32	8.10
	**CIS (ppm)**		**-0.13**	**0.2**	**0.11**

a)

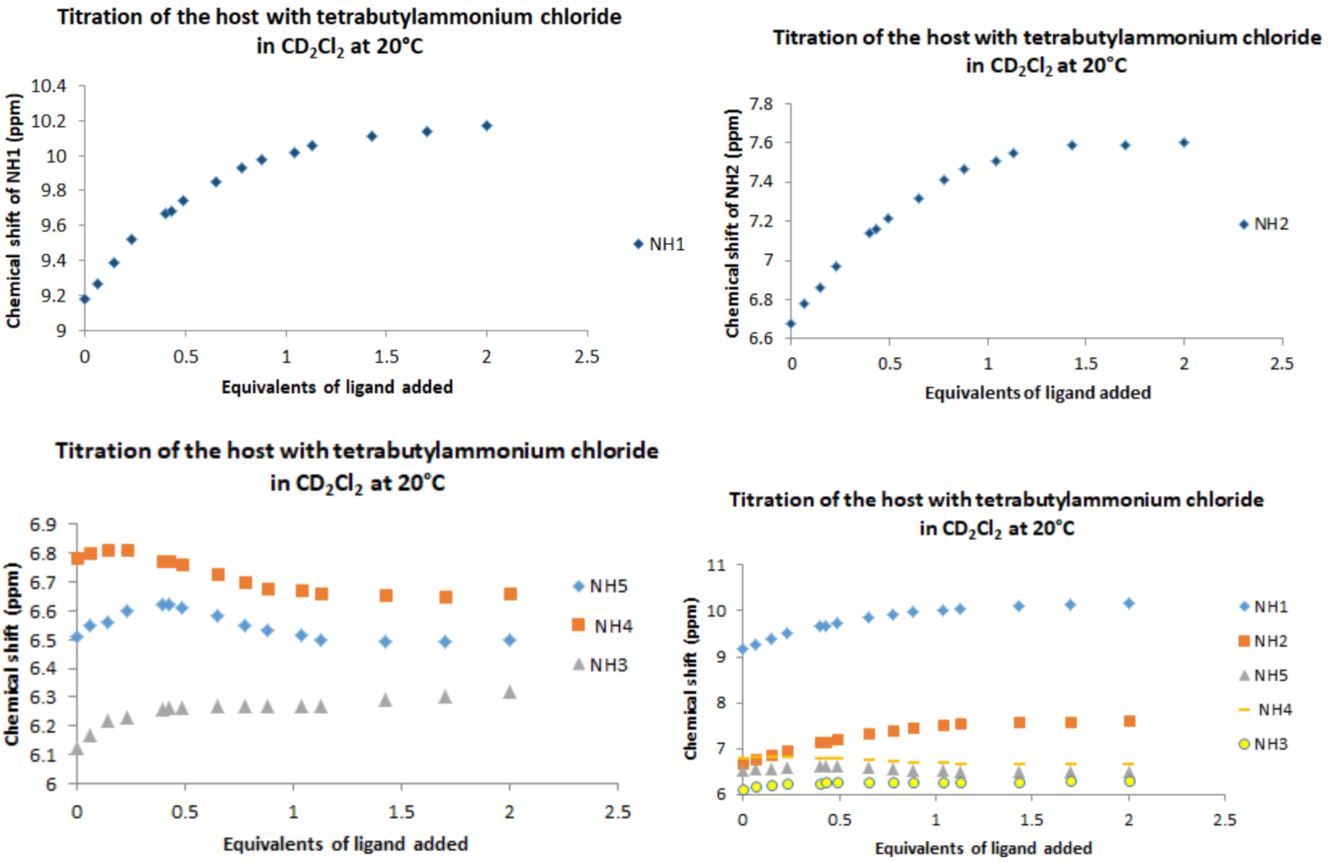

b)

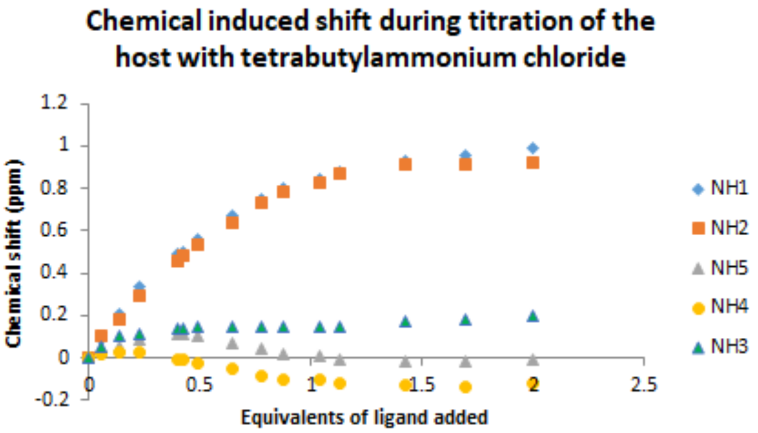

Supplementary Figure 37. a) Plots of variation of chemical shifts of NH signals, and b) plot of chemical induced shifts of NH signals of compound **1** (18 mM in CD_2Cl_2) upon addition of increments of tetrabutylammonium chloride, recorded by ^{1}H NMR at 25 °C (400 MHz).

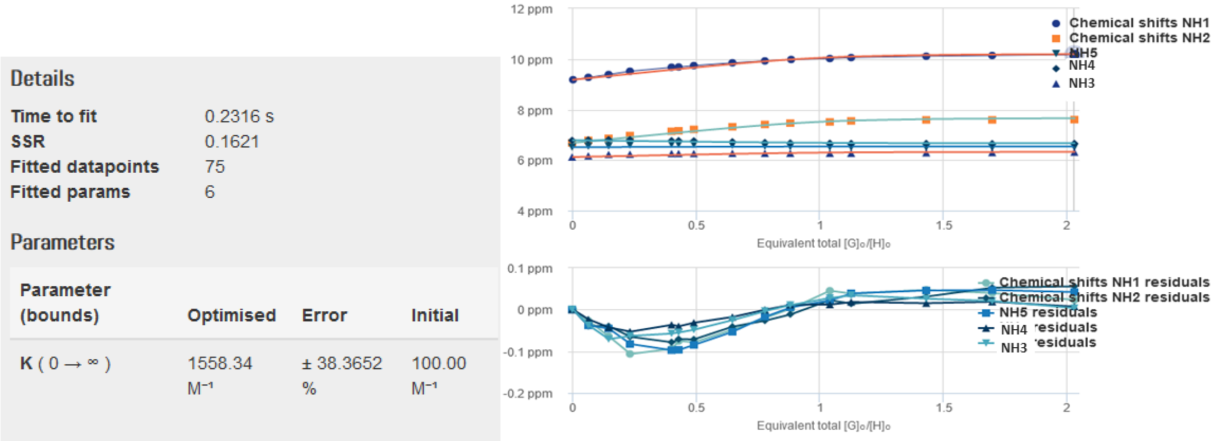

Details

Time to fit	0.2316 s
SSR	0.1621
Fitted datapoints	75
Fitted params	6

Parameters

Parameter (bounds)	Optimised	Error	Initial
K (0 → ∞)	1558.34 M⁻¹	± 38.3652 %	100.00 M⁻¹

Supplementary Figure 38. Non-linear curve fitting analysis of the experimental titration data of compound **1** (18 mM in CD_2Cl_2) with increments of tetrabutylammonium chloride recorded at 25 °C (400 MHz) using a theoretical binding isotherm for 1:1 binding, Source data are provided as a Source Data file.http://app.supramolecular.org/bindfit/view/146b5828-2bf6-4cbb-ae2f-fe31d0959d1a

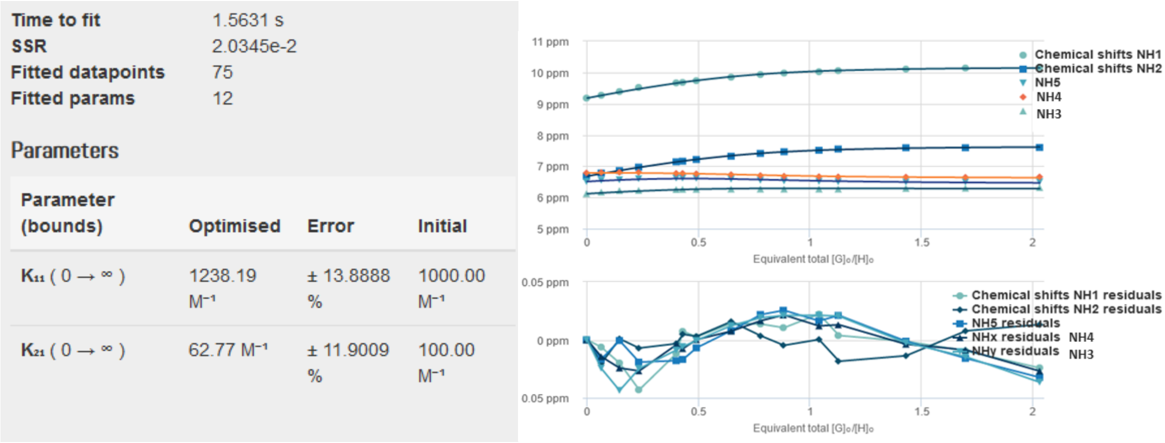

Time to fit	1.5631 s
SSR	2.0345e-2
Fitted datapoints	75
Fitted params	12

Parameters

Parameter (bounds)	Optimised	Error	Initial
K_{11} (0 → ∞)	1238.19 M⁻¹	± 13.8888 %	1000.00 M⁻¹
K_{21} (0 → ∞)	62.77 M⁻¹	± 11.9009 %	100.00 M⁻¹

Supplementary Figure 39. Non-linear curve fitting analysis of the experimental titration data of compound **1** (18 mM in CD_2Cl_2) with increments of tetrabutylammonium chloride recorded at 25 °C (400 MHz) using a theoretical binding isotherm for 2:1 binding, **http://app.supramolecular.org/bindfit/view/99a15107-e94b-4ba0-bf93-4a9455a392bc**

5.1.5. NMR signal assignment of compound **1** mixed with 2 equivalents of tetrabutylammonium chloride in CD_2Cl_2 at 25 °C

^{1}H and ^{13}C NMR signals were assigned based on COSY, HSQC, HMBC and 2D NOESY experiments in CD_2Cl_2. ^{1}H ^{1}H 2D NOESY NMR experiment (Supplementary Figure 40) shows correlation peaks between CH_2^{10} and both NH^5 and H^8; the correlation peak between NH^5 and CH_2^{10} is weaker in intensity than the correlation between NH^5 and CH_2^{12}, indicating a directionality preference opposite to the one observed in the absence of ligand (compare with supplementary figure 29).

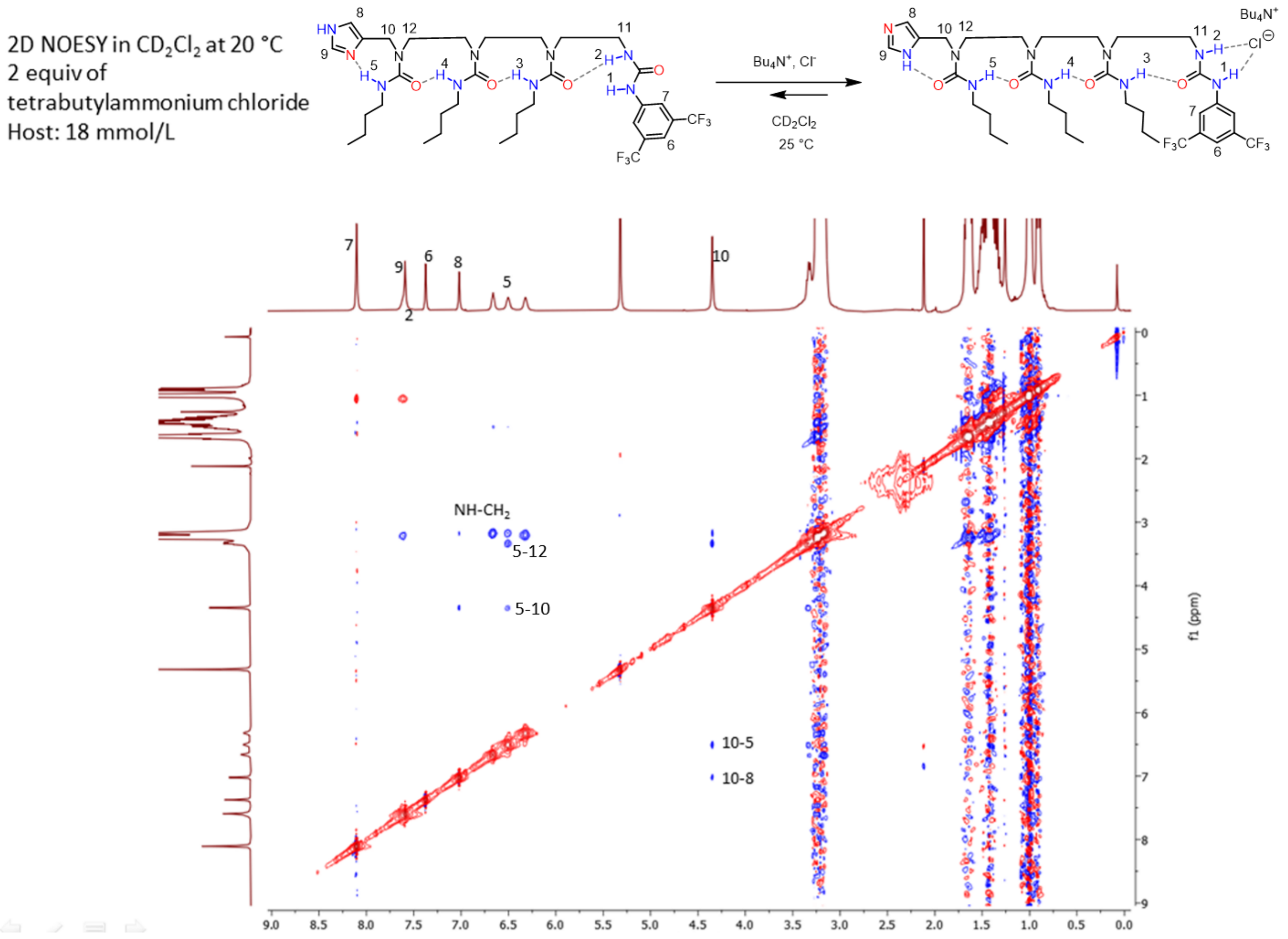

Supplementary Figure 40. ^1H-^1H 2D NOESY NMR spectrum of compound **1** (18 mM in CD_2Cl_2) in the presence of 2 equivalents of tetrabutylammonium chloride at 25 °C (400 MHz).

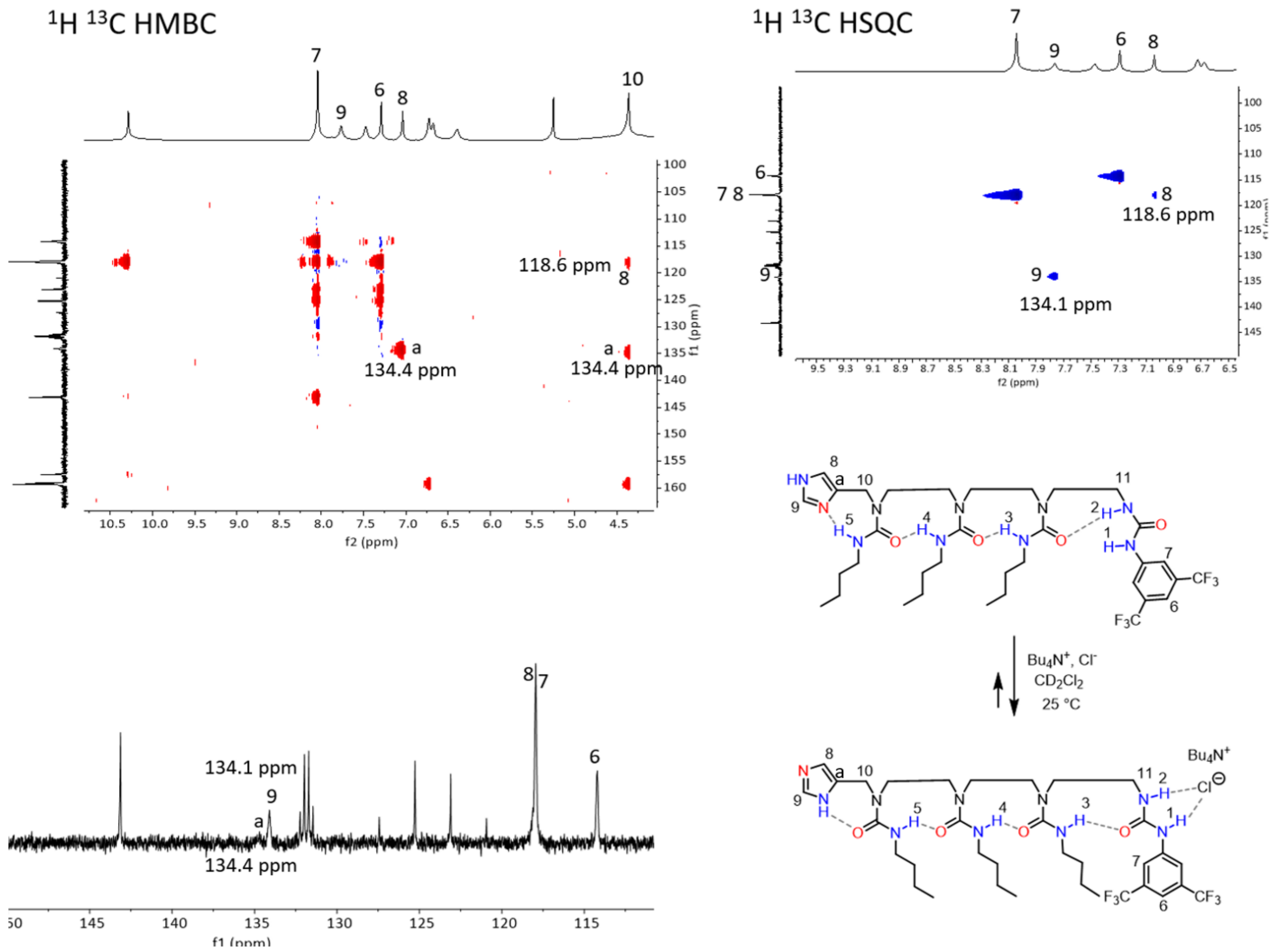

Supplementary Figure 41. NMR spectra of compound **1** (45 mM in CD_2Cl_2 at 25 °C) in the presence of 2 equivalents of tetrabutylammonium chloride at 25 °C (400 MHz).

5.1.6. Variable temperature NMR of compound 1 in the presence of 2 equivalents of tetrabutylammonium chloride

^{1}H NMR spectra of compound **1** (18 mM in CD_2Cl_2) in the presence of 2 equivalents of tetrabutylammonium chloride were recorded between 25 °C and -40 °C (Supplementary Figure 42). The compound precipitates out of solution at -40 °C (seen by eye), likely due to self-aggregation at lower temperatures.

a)

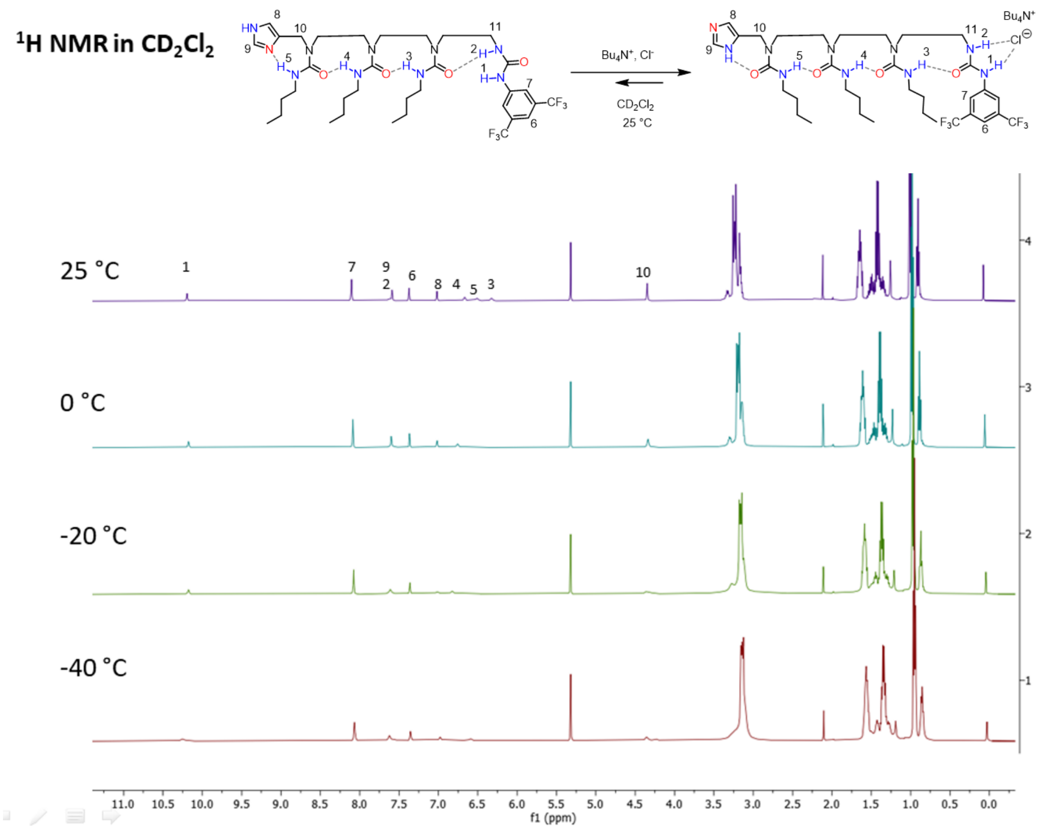

b)

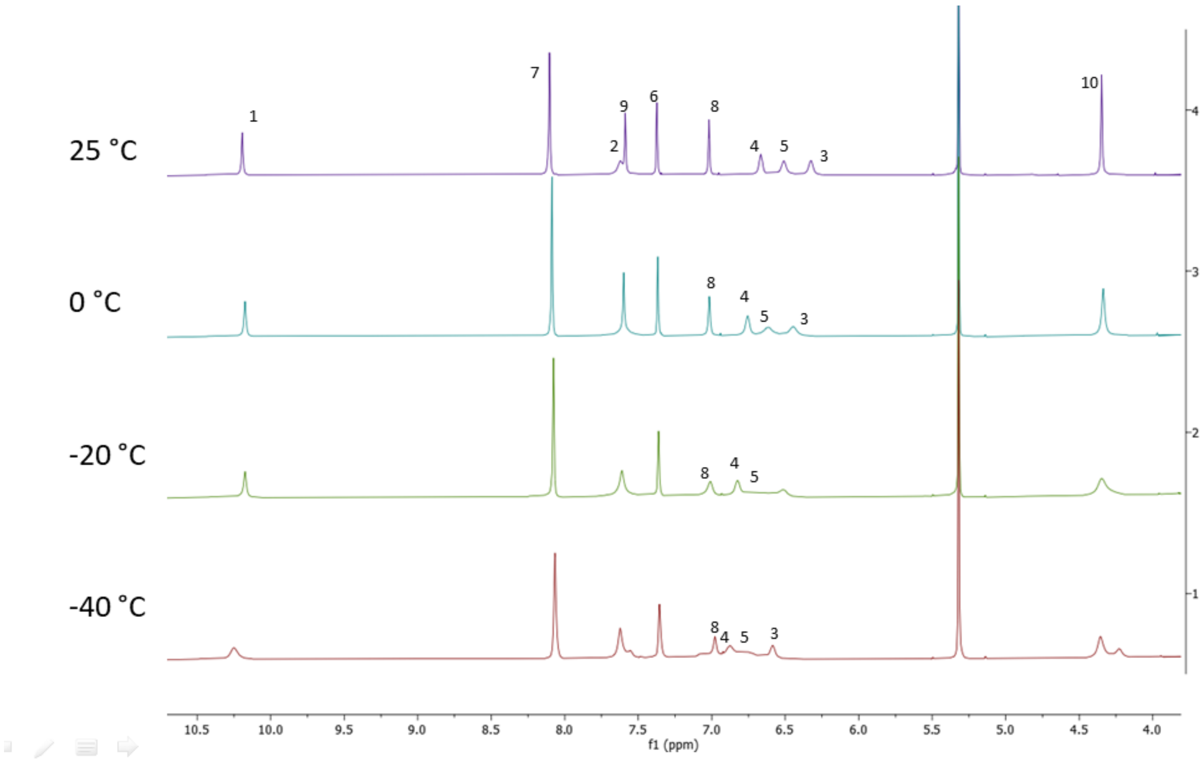

Supplementary Figure 42. Overlay of ^{1}H NMR spectra (500 MHz) of compound **1** (18 mM in CD_2Cl_2) in the presence of tetrabutylammonium chloride (e equivalents) recorded between 25 °C and -40 °C (a) full spectra and b) expansion of the 4ppm-10ppm region).

5.2. Compound **2**

5.2.1. Assignment of ^{1}H and ^{13}C NMR signals

^{1}H and ^{13}C NMR signals were assigned based on COSY, HSQC, HMBC and 2D NOESY experiments in $CDCl_3$.

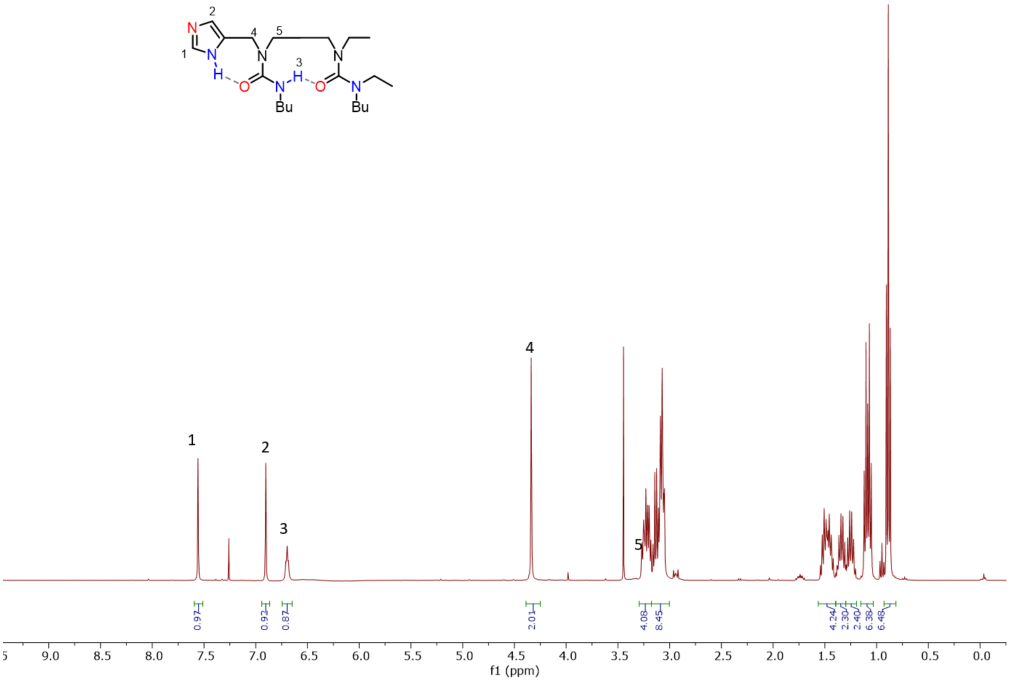

Supplementary Figure 43. ^{1}H NMR spectrum of compound **2** in $CDCl_3$ at 25 °C (400 MHz).

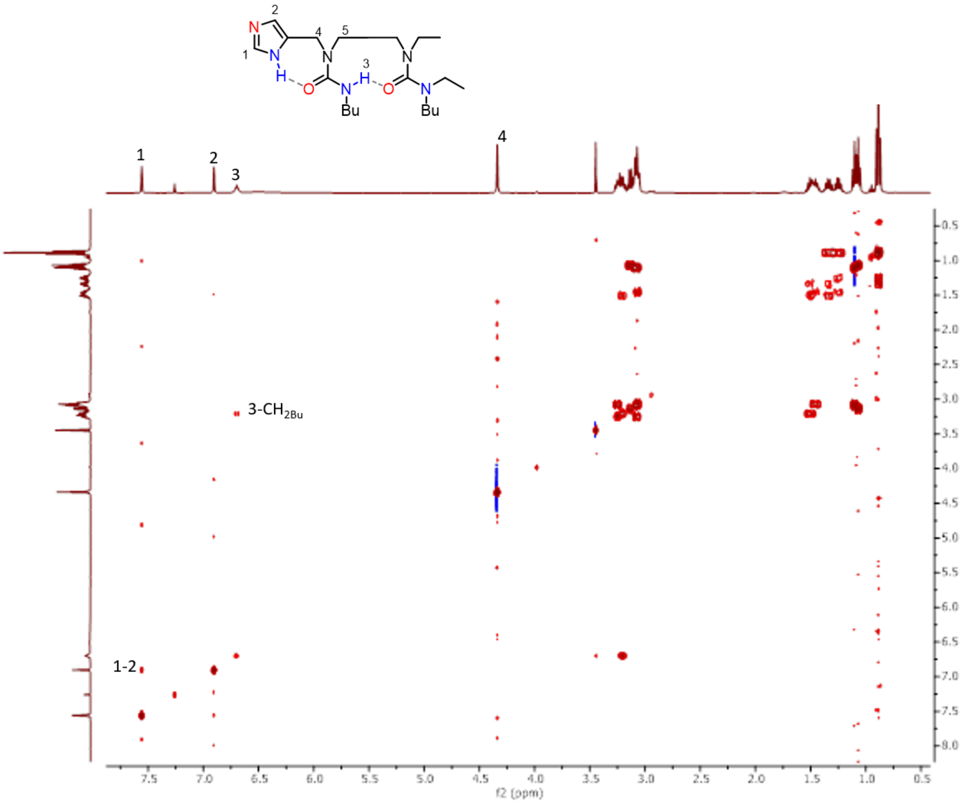

Supplementary Figure 44. ^{1}H-^{1}H COSY NMR spectrum of compound **2** in $CDCl_3$ at 25 °C (400 MHz).

A)

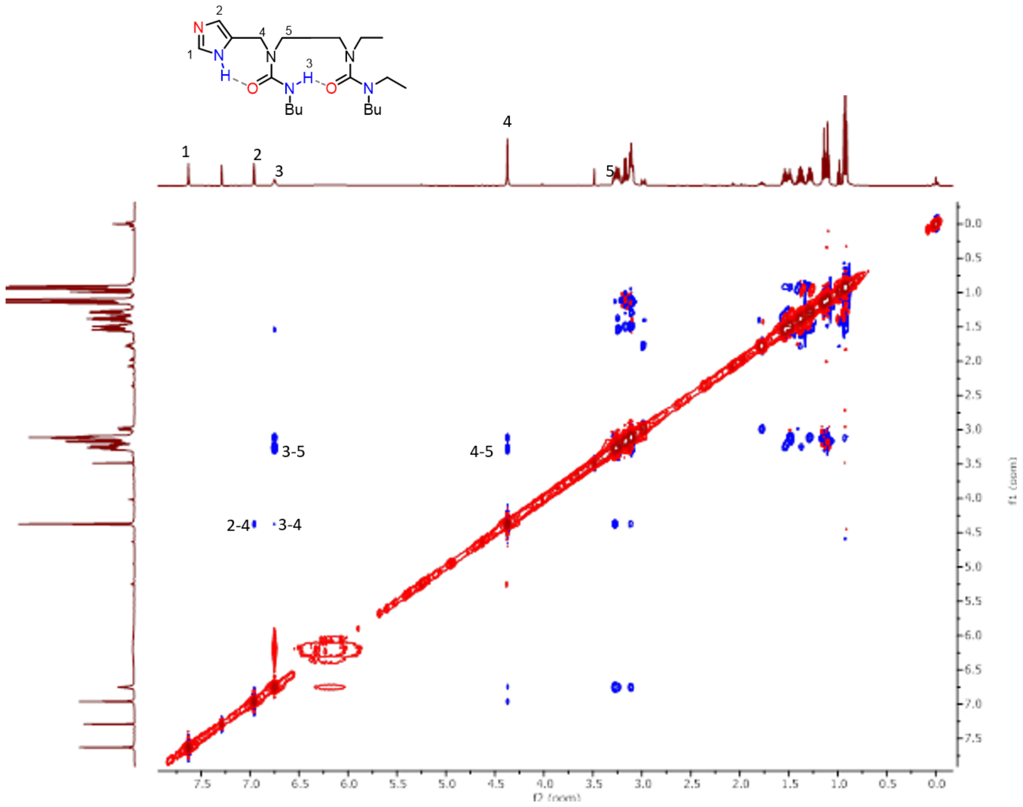

B)

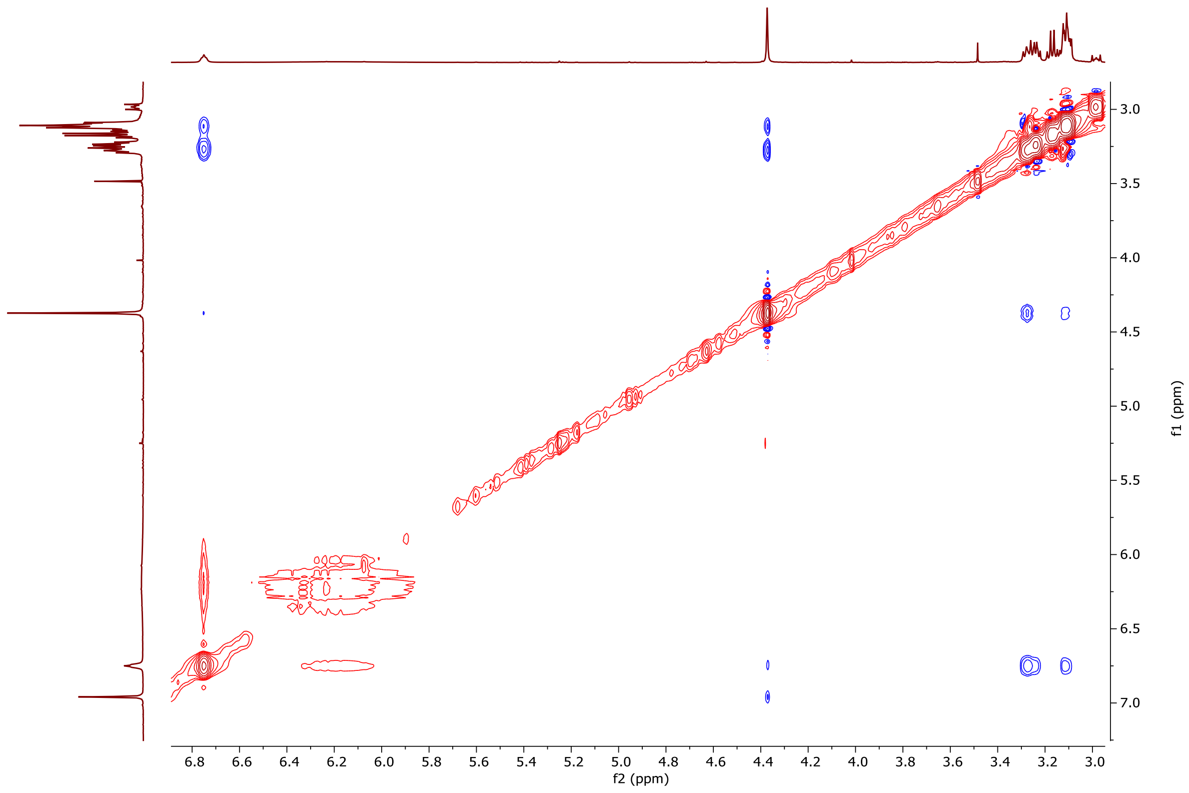

Supplementary Figure 45. 2D ^{1}H-^{1}H NOESY spectrum of compound **2** in $CDCl_3$ at 25 °C (400 MHz) (A) full spectrum and B) expansion between 3.00-7.00 ppm).

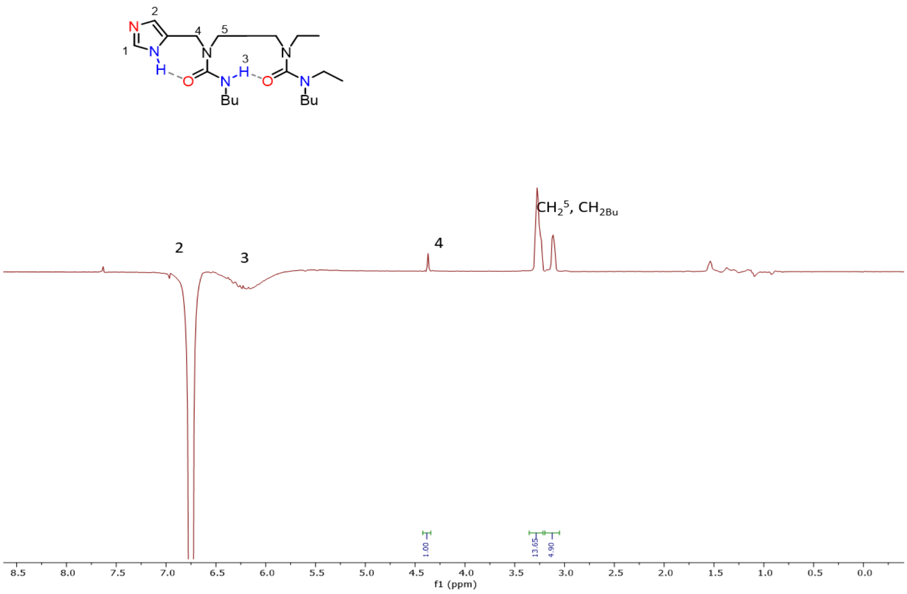

Supplementary Figure 46. 1D NOE spectrum in $CDCl_3$ of compound **2** irradiating at the frequency of N*H*^{n}Bu. The spectrum shows a correlation between the N*H*^{3n}Bu with *CH*$_2$^{5}, and a comparatively very weak correlation with *CH*$_2$^{4}. This indicates a favoured hydrogen bond directionality with the N*H*Bu urea carbonyl pointing towards the imidazole. The observation is coherent with previous observations collected on analogous ethylenediamine bridged ureas bearing a covalent hydrogen bond directionality controller placed at one terminus of the chain.[3]

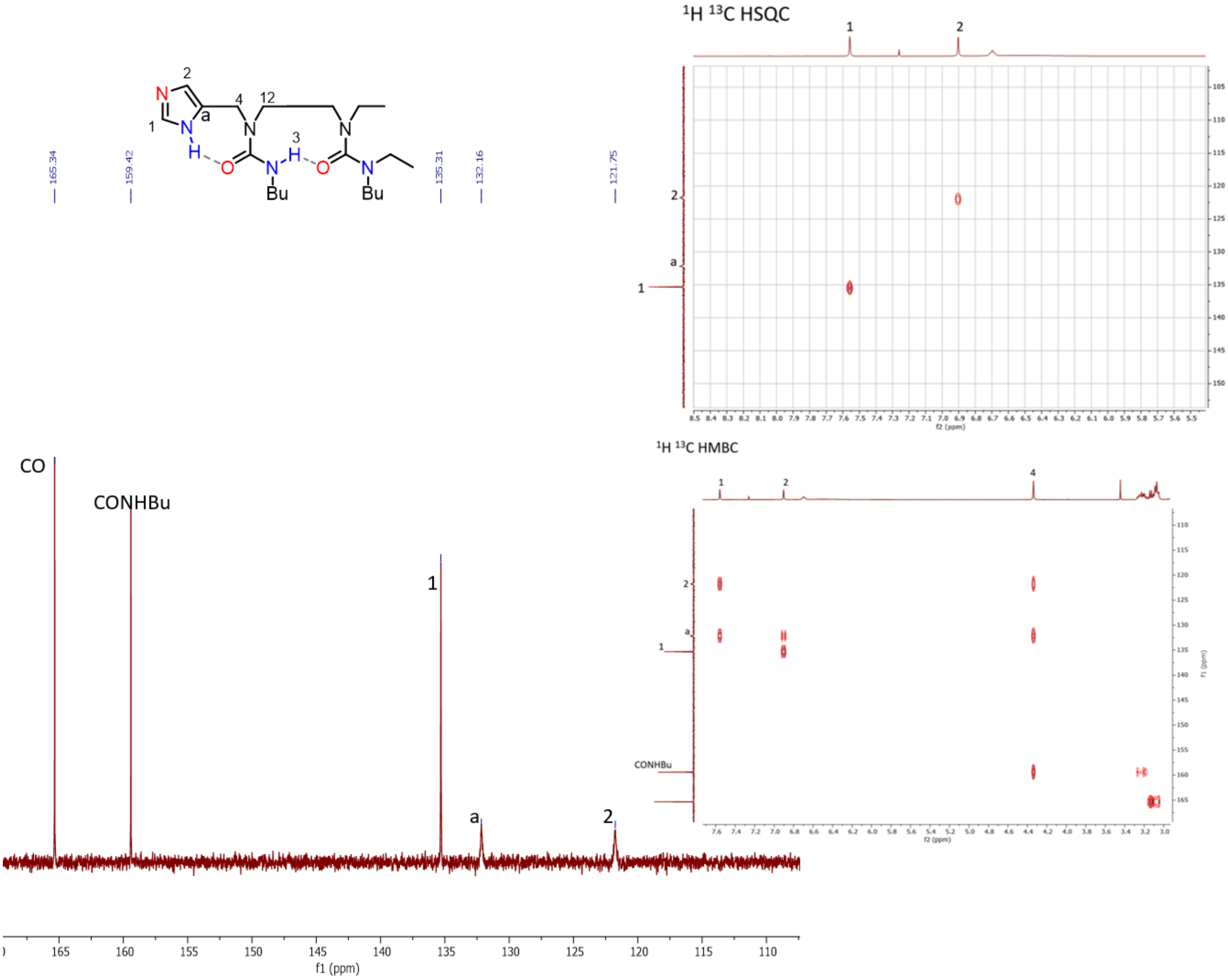

Supplementary Figure 47. Chemical shifts of imidazole of compound **2** in $CDCl_3$.

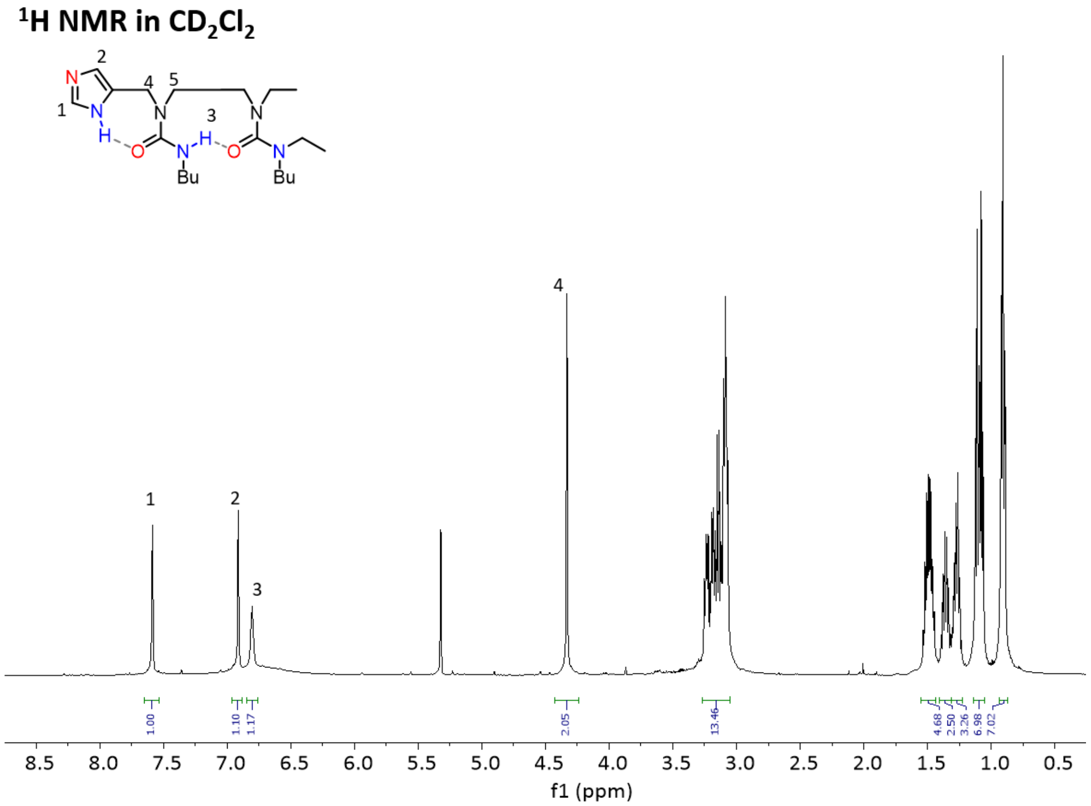

Supplementary Figure 48. ^{1}H NMR spectrum of compound **2** in CD_2Cl_2 at 25 °C (400 MHz).

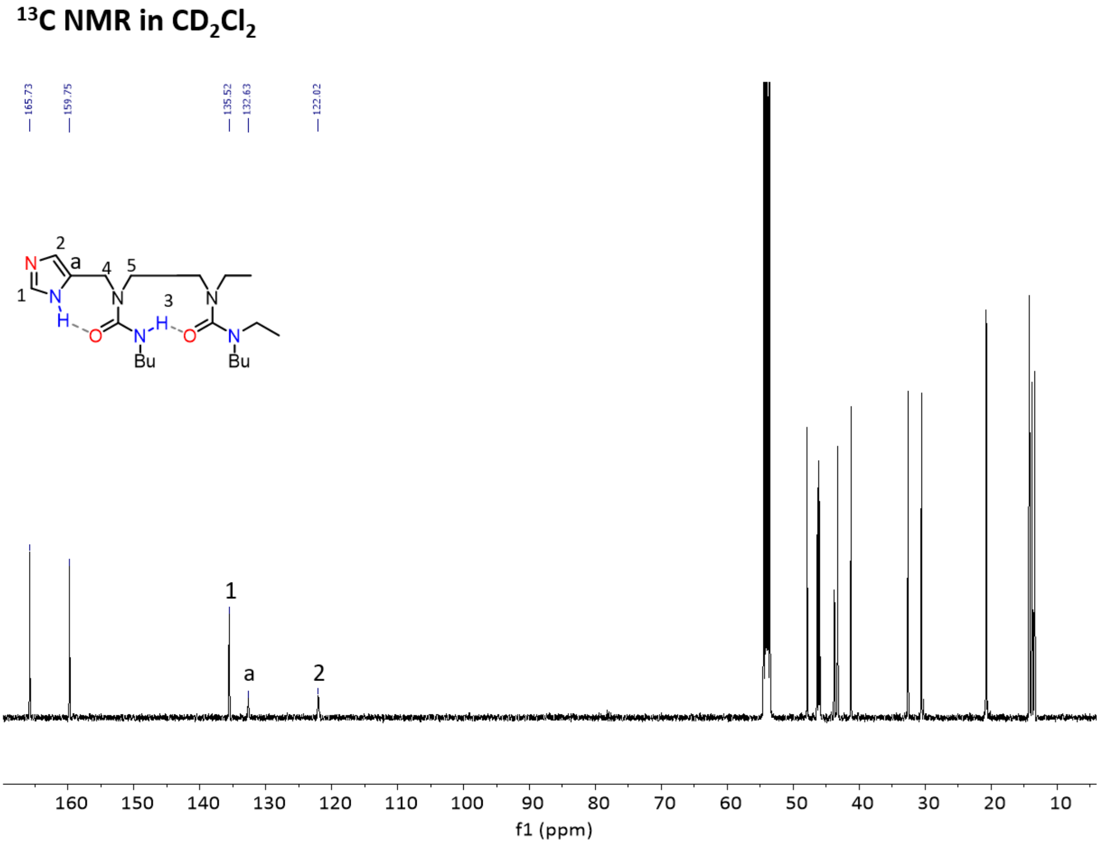

Supplementary Figure 49. ^{13}C NMR spectrum of compound **2** in CD_2Cl_2 at 25 °C (400 MHz).

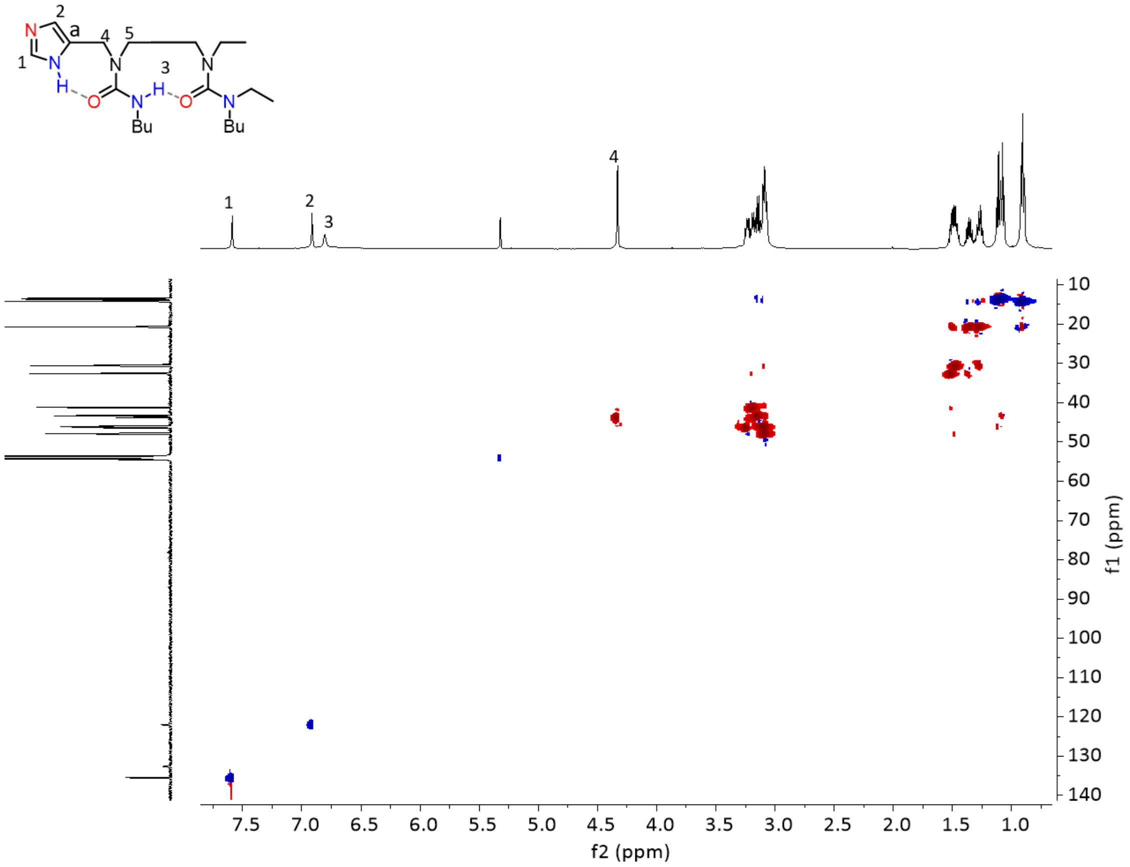

Supplementary Figure 50. ^{1}H ^{13}C HSQC NMR spectrum of compound **2** in CD_2Cl_2 at 25 °C (400 MHz).

^{1}H-^{13}C HMBC in CD_2Cl_2

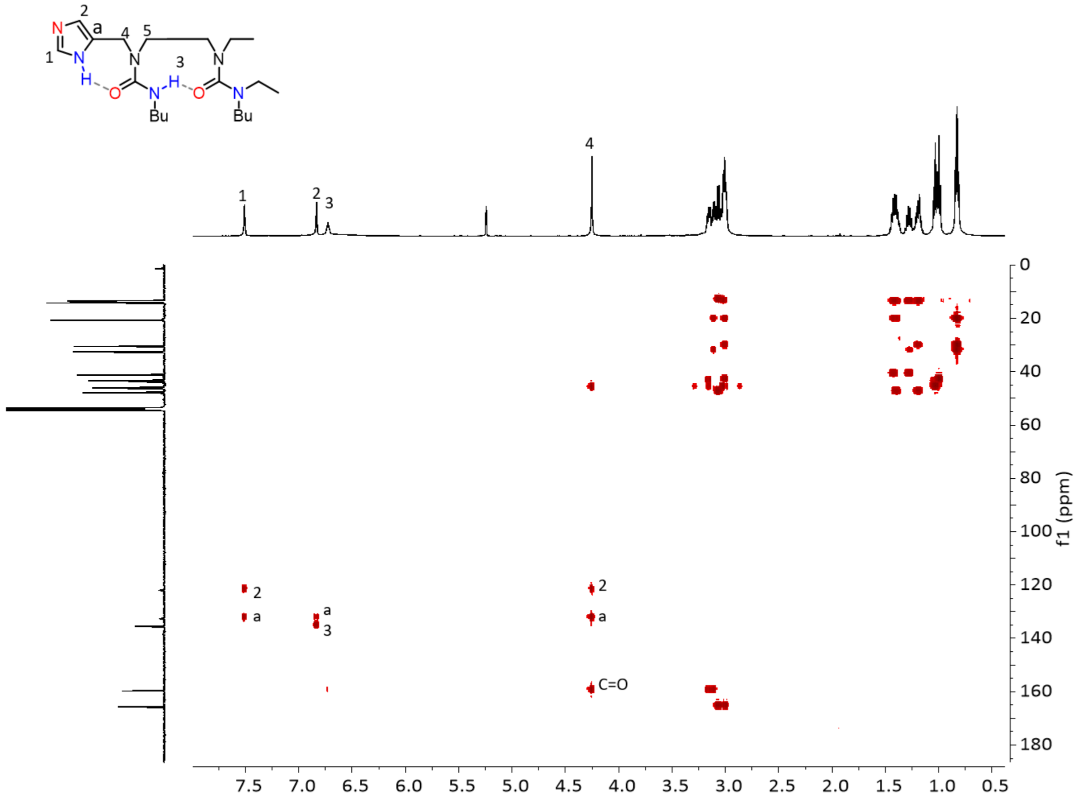

Supplementary Figure 51. ^{1}H ^{13}C HMBC NMR spectrum of compound **2** in CD_2Cl_2 at 25 °C (400 MHz).

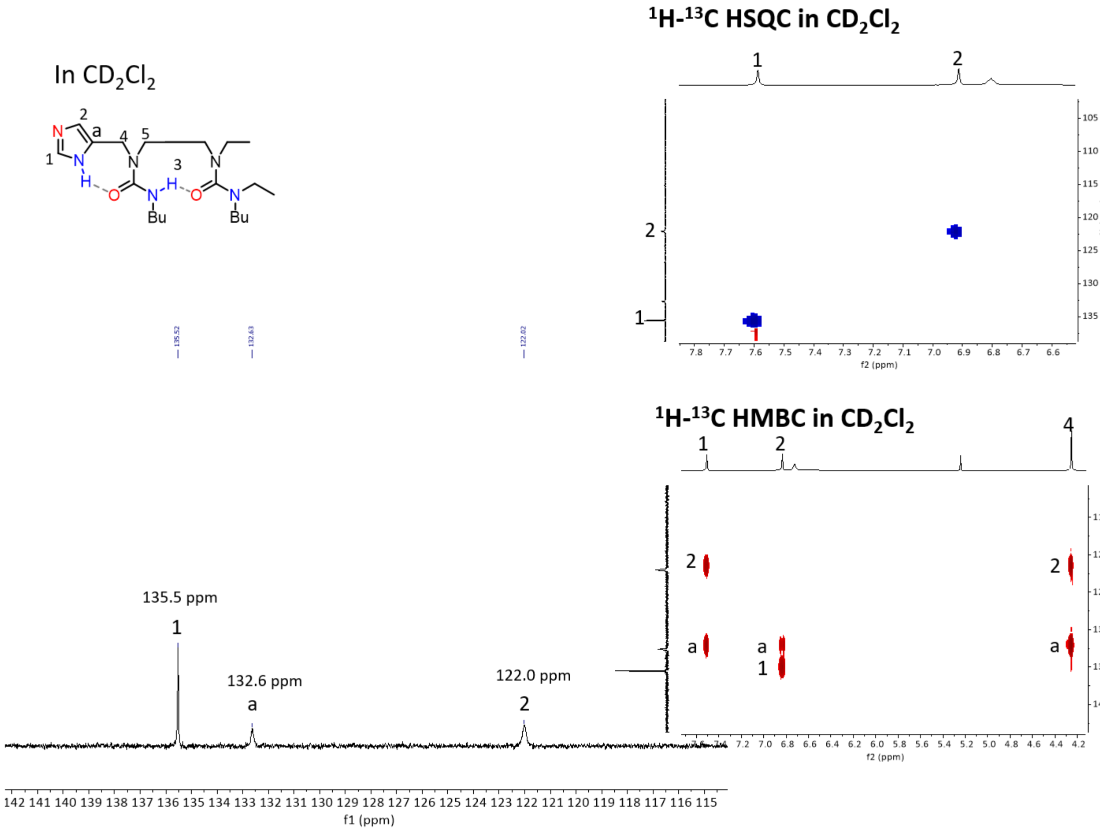

Supplementary Figure 52. ^{13}C, HSQC, HMBC NMR spectra of compound **2** in CD_2Cl_2 at 25 °C- expansions.

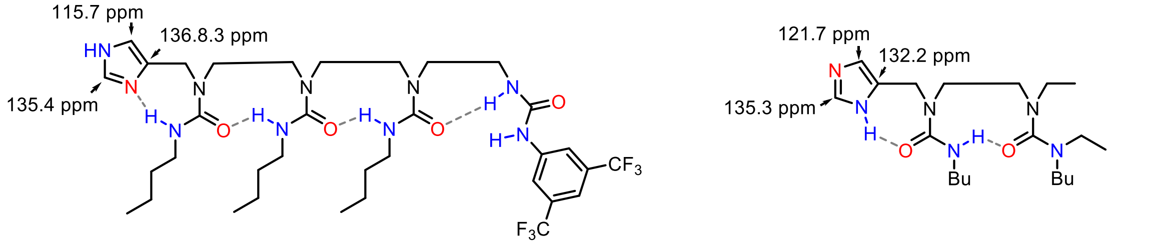

1 **2**

Supplementary Figure 53. ^{13}C NMR chemical shifts of imidazole carbons in compounds **1** and **2** in $CDCl_3$ at 25 °C.

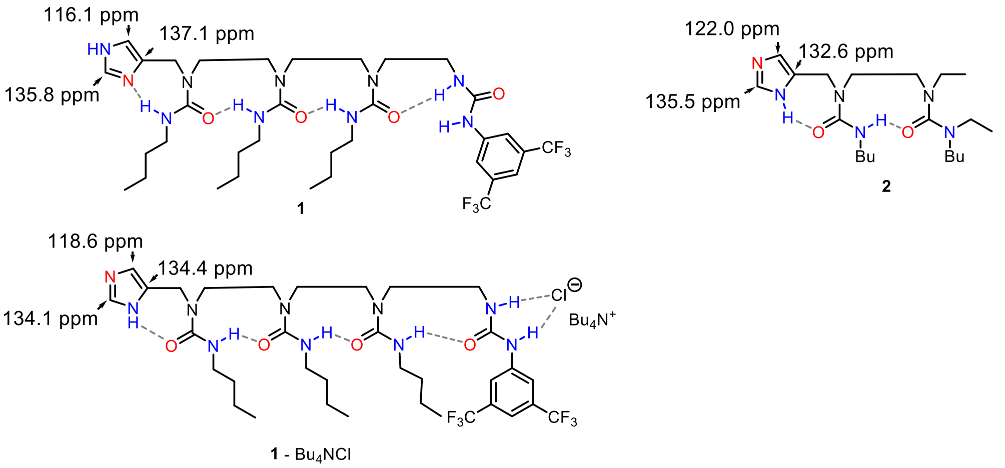

Supplementary Figure 54. ^{13}C NMR chemical shifts of imidazole carbons in compounds **1** and **2** in CD_2Cl_2 at 25 °C.

5.2.2. DFT calculations on compound **2**

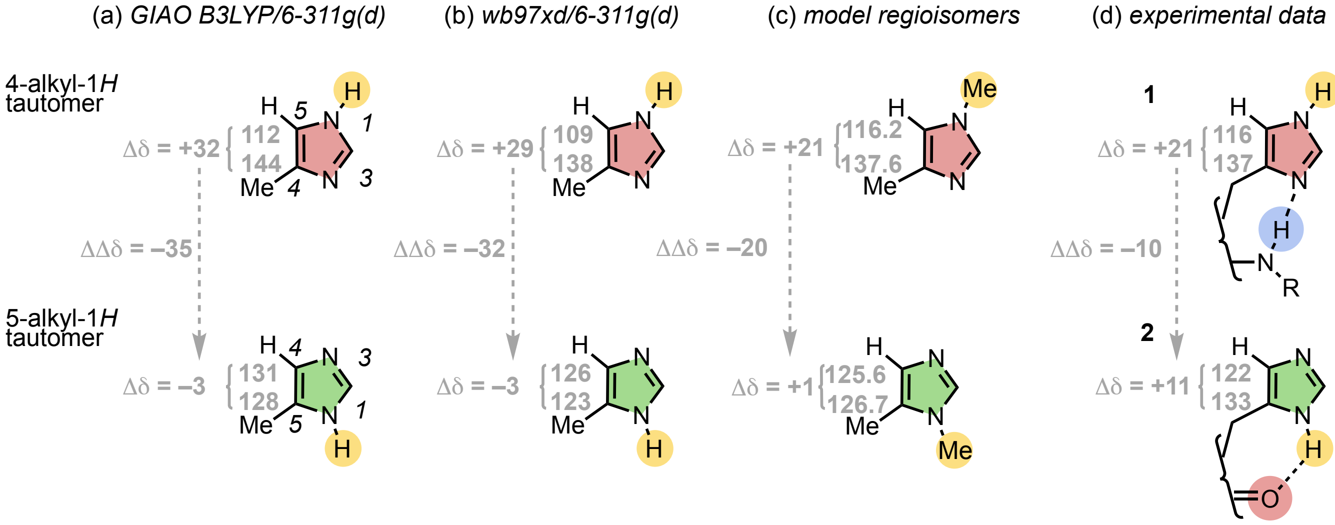

Supplementary Figure 55. Identifying the tautomer state of a substituted imidazole by ^{13}C NMR spectroscopy. (a,b) Computed ^{13}C NMR chemical shifts (using two different functionals) for the two tautomers of methylimidazole (Δδ values shown in ppm). (c) Model regioisomers 1,4-dimethylimidazole and 1,5-dimethylimidazole. (d) Experimental ^{13}C NMR chemical shifts (CD_2Cl_2, 25 °C) for device **1** and benchmark structure **2**. Geometries and frequencies for 4-methylimidazole and 5-methylimidazole were optimised and calculated by DFT using Gaussian16 (Revision A.03)[4] with the B3LYP density functional,[5,6] a 6-31G(d) split-valence basis set[7] and an ultrafine integration grid. Chemical shifts and spin-spin coupling constants[8,9] were calculated from the optimised geometries using Gauge-Independent Atomic Orbital (GIAO) method.[10,11] The B3LYP density functional and ultrafine integration grid were maintained from the geometry and frequency calculations, however a larger 6-311G(d) split-valence basis set was used.[12] The NMR parameters were also calculated from the same input geometries as before using the ωB97-XD functional[13] and 6-311G(d) basis set. The difference in chemical shift (ΔΔδ) between C4 and C5 is consistently reduced significantly in the tautomer or regioisomer with the protonated or methylated nitrogen adjacent to the C-alkyl substituent.

5.2.3. Titration of compound **2** with tetrabutylammonium chloride in CD_2Cl_2 at 25 °C

Titration of compound **2** (10.64 mM) in CD_2Cl_2 at 25 °C with increasing amounts of tetrabutylammonium chloride (from 0 to 2 equivalents) was monitored by ^{1}H NMR (Supplementary Figure 56). The ligand was added as a solid so no variation of concentration occurs during the titration. The values of chemical shifts of the NH of compound **3** upon addition of tetrabutylammonium chloride are collected in Supplementary Table 3. Minimal shft of ^{1}H NMR signals (0.01-0.03 ppm) occur upon addition of tetrabutylammonium chloride, indicative of negligible hydrogen binding between the chloride anion and compound **2** under the conditions tested.

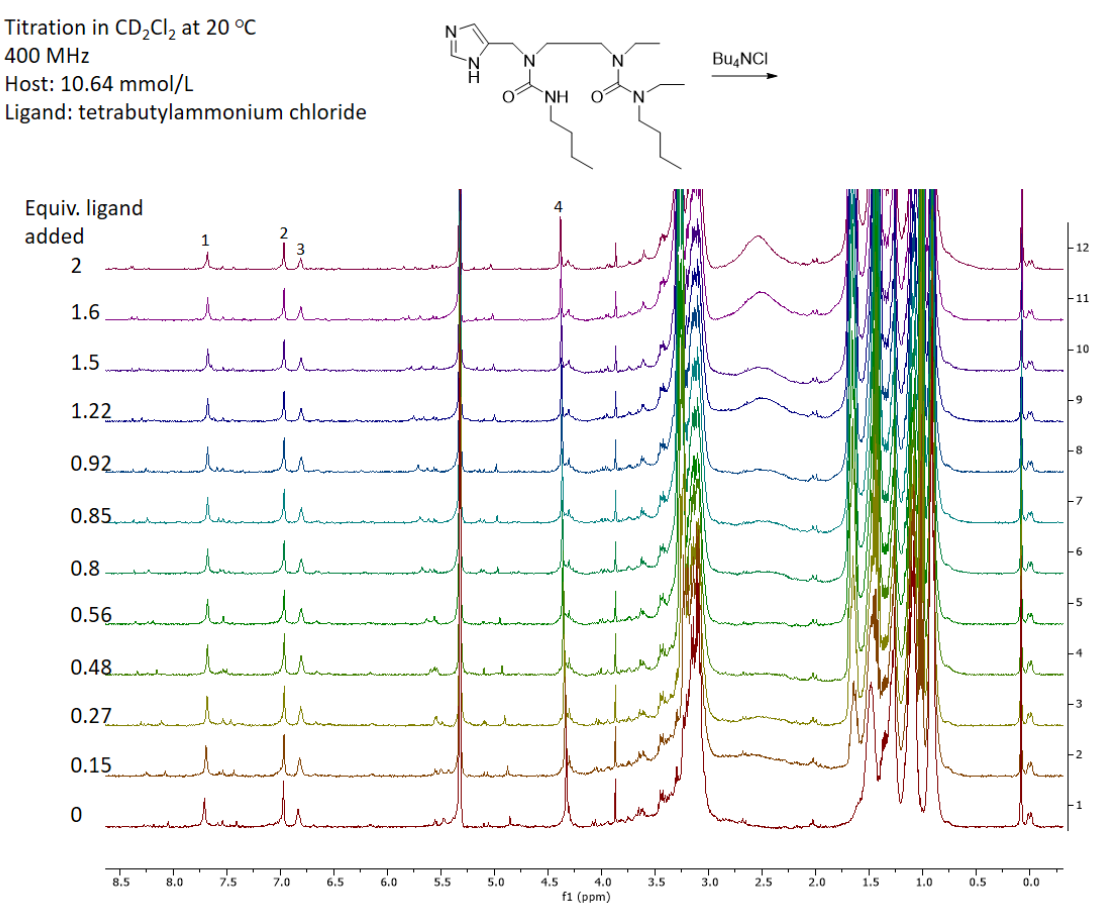

Supplementary Figure 56. Overlay of ^{1}H NMR spectra of compound **2** (10.6 mM in CD_2Cl_2) in the presence of increasing amounts of tetrabutylammonium chloride from 0 to 2 equivalents, recorded at 25 °C (400 MHz).

Supplementary Table 3. Chemical shifts of ^{1}H NMR signals of compound **2** recorded during the NMR titration of **2** (10.6 mM) with increments of tetrabutylammonium chloride at 25 °C (400 MHz). N$H_{imidazole}$ not visible.

Concentration of host (mol/L)	Concentration of chloride ligand (mol/L)	Equiv. ligand	Chemical shift NH^3 (ppm)	Chemical shift CH^1 (ppm)	Chemical shift CH^2 (ppm)
0.0106	0	0	6.83	7.71	6.97
0.0106	0.00159	0.15	6.82	7.69	6.96
0.0106	0.002862	0.27	6.81	7.68	6.96
0.0106	0.005088	0.48	6.8	7.68	6.96
0.0106	0.005936	0.56	6.8	7.68	6.96
0.0106	0.00848	0.8	6.8	7.68	6.96
0.0106	0.00901	0.85	6.8	7.68	6.96
0.0106	0.009752	0.92	6.8	7.68	6.96
0.0106	0.01293	1.22	6.8	7.68	6.96
0.0106	0.0159	1.5	6.8	7.68	6.96
0.0106	0.01696	1.6	6.8	7.68	6.96
0.0106	0.0212	2	6.8	7.68	6.96
**CIS (ppm)**			**0.03**	**0.03**	**0.01**

5.3. Compound **3**

5.3.1. Assignment of ^{1}H and ^{13}C NMR signals

^{1}H COSY NMR experiment of compound **3** in $CDCl_3$ (Supplementary Figure 58) shows couplings between NH^{14} and H^{8}, between NH^{15} and H^{7}, allowing for the assignment of both NH^{14} and NH^{15} signals. Overlapping of NH^{13} signal with the methylene signal of the benzyl group was observed. To overcome the problem a range of temperatures was screened in CD_2Cl_2 using a 700 MHz NMR apparatus (Supplementary Figure 60). Variable temperature ^{1}H NMR between 5 °C and 35 °C clearly show NH^{13} signal. 2D NOESY experiment (Supplementary Figures 61, 63) show correlation signals between NH^{13} and H^{12}, between ^{t}butyl H^{2} and H^{12}, allowing for assignment of NH^{13} signal and giving indication on the directionality of the hydrogen bond chain. ^{15}N HSQC experiment also confirmed the position of the NH signals.

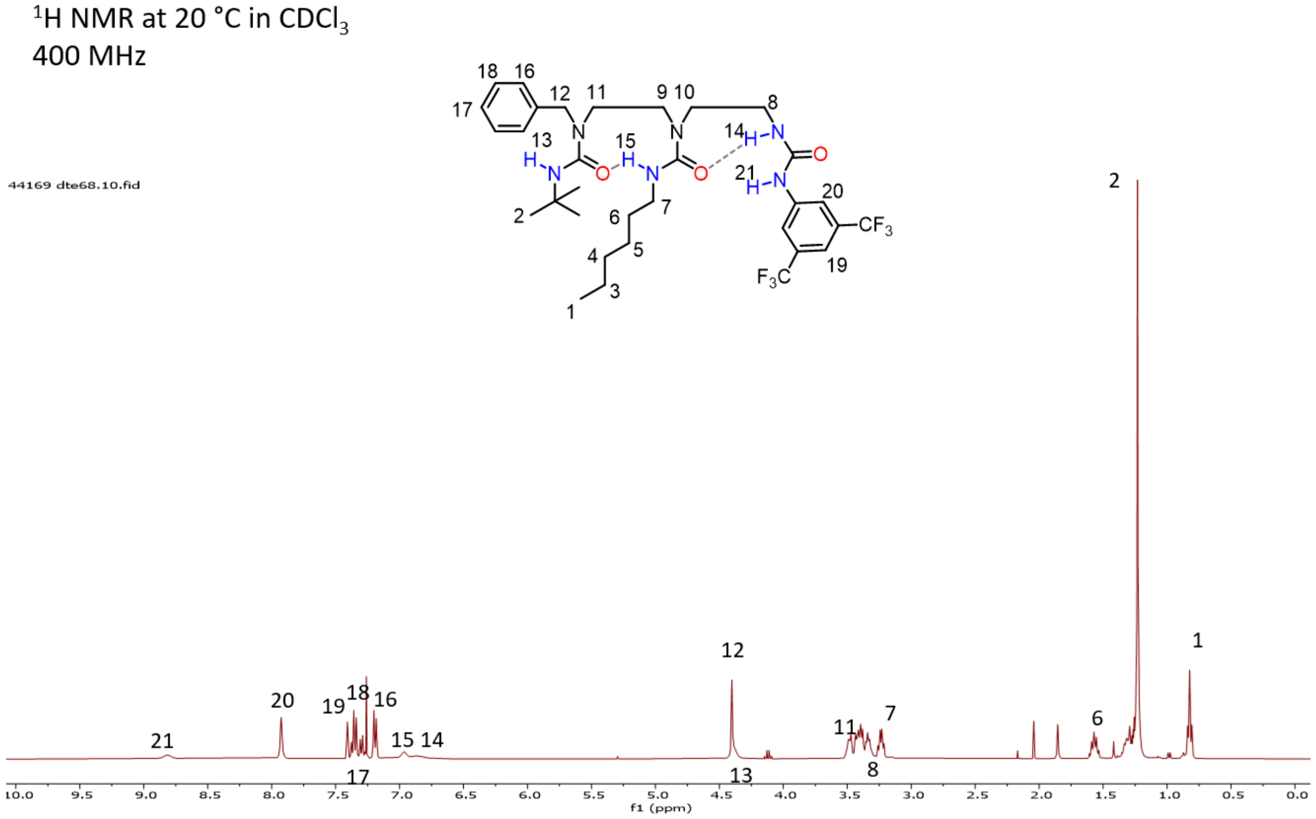

Supplementary Figure 57. ^{1}H NMR spectrum of compound **3** in $CDCl_3$ at 20 °C (400 MHz).

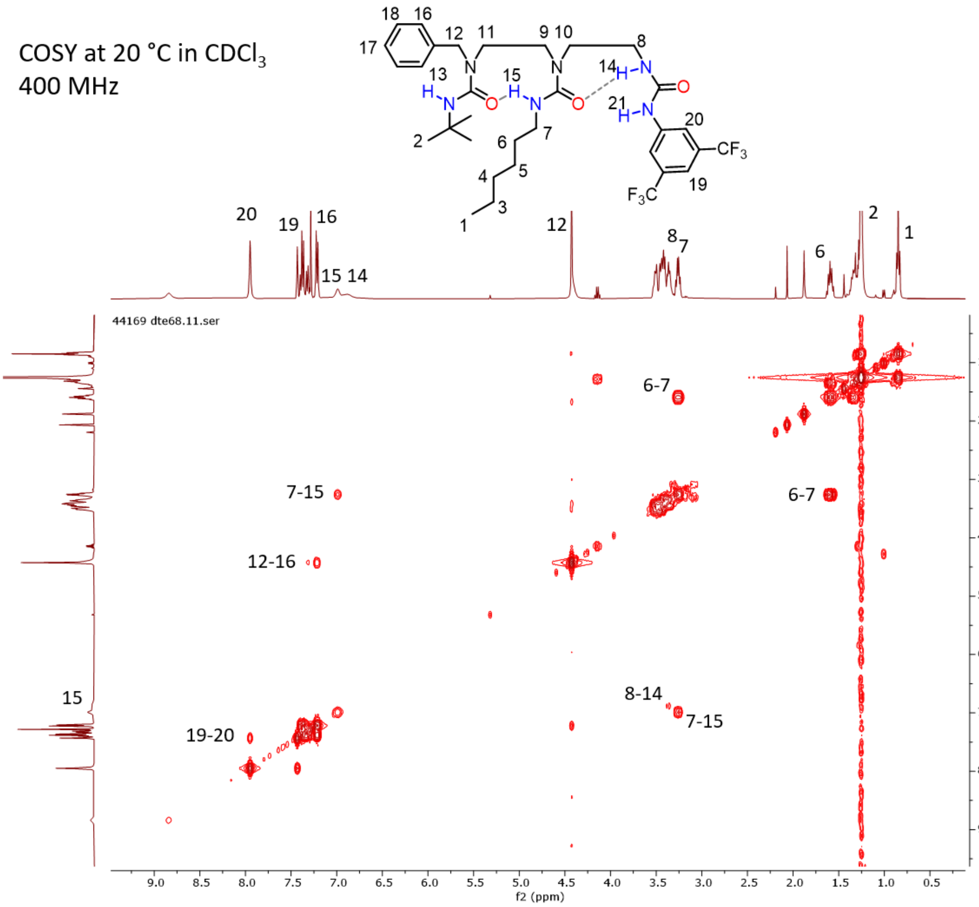

Supplementary Figure 58. ^{1}H COSY NMR spectra of compound **3** in $CDCl_3$ at 20 °C (400 MHz).

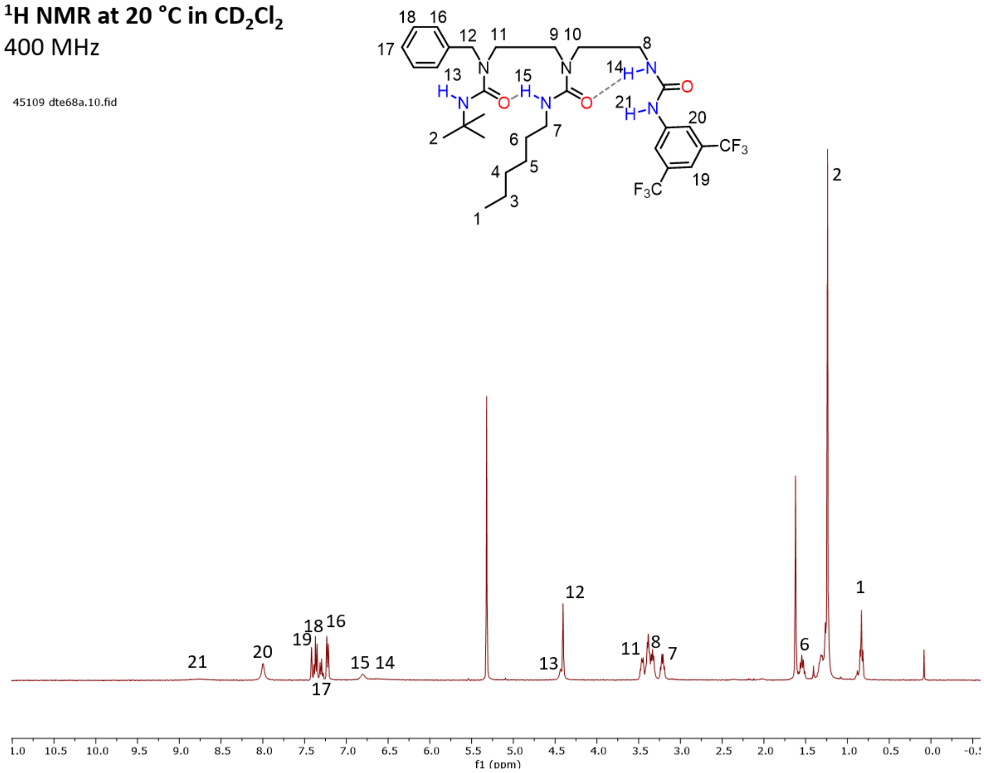

Supplementary Figure 59. ^{1}H NMR spectrum of compound **3** in CD_2Cl_2 at 20 °C (400 MHz).

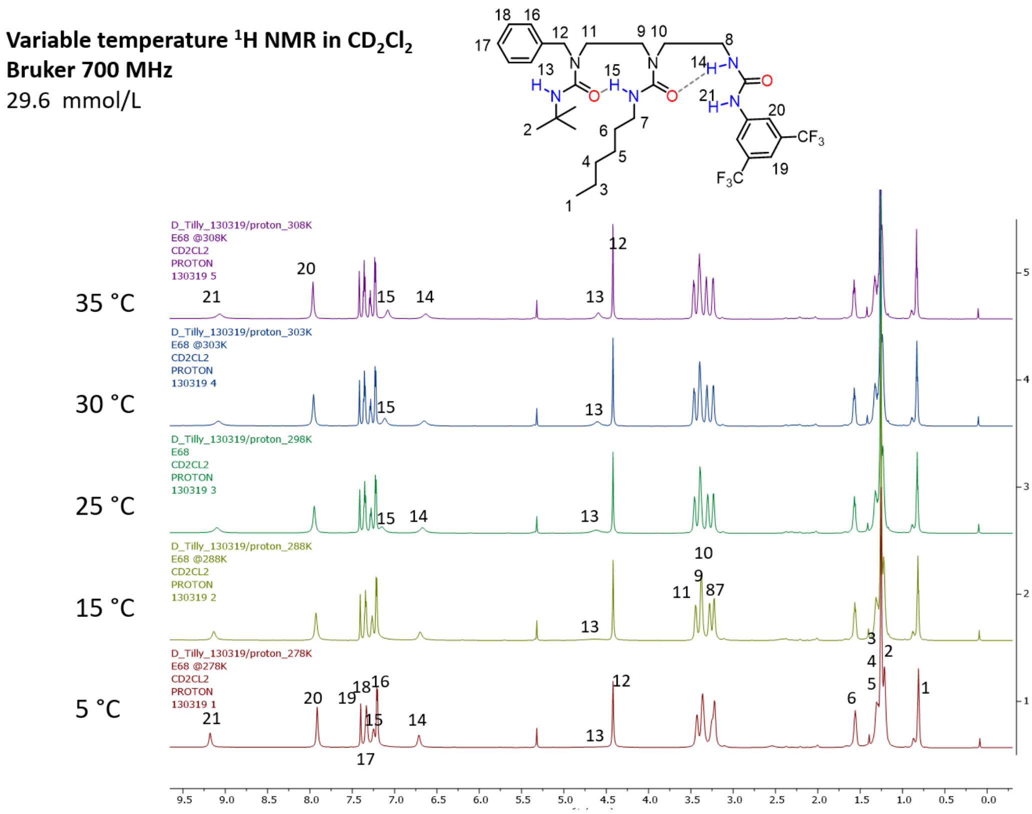

Supplementary Figure 60. Overlay of ^{1}H NMR spectra of compound **3** (29.6 mM in CD_2Cl_2) recorded (700 MHz) between 5 °C and 35 °C.

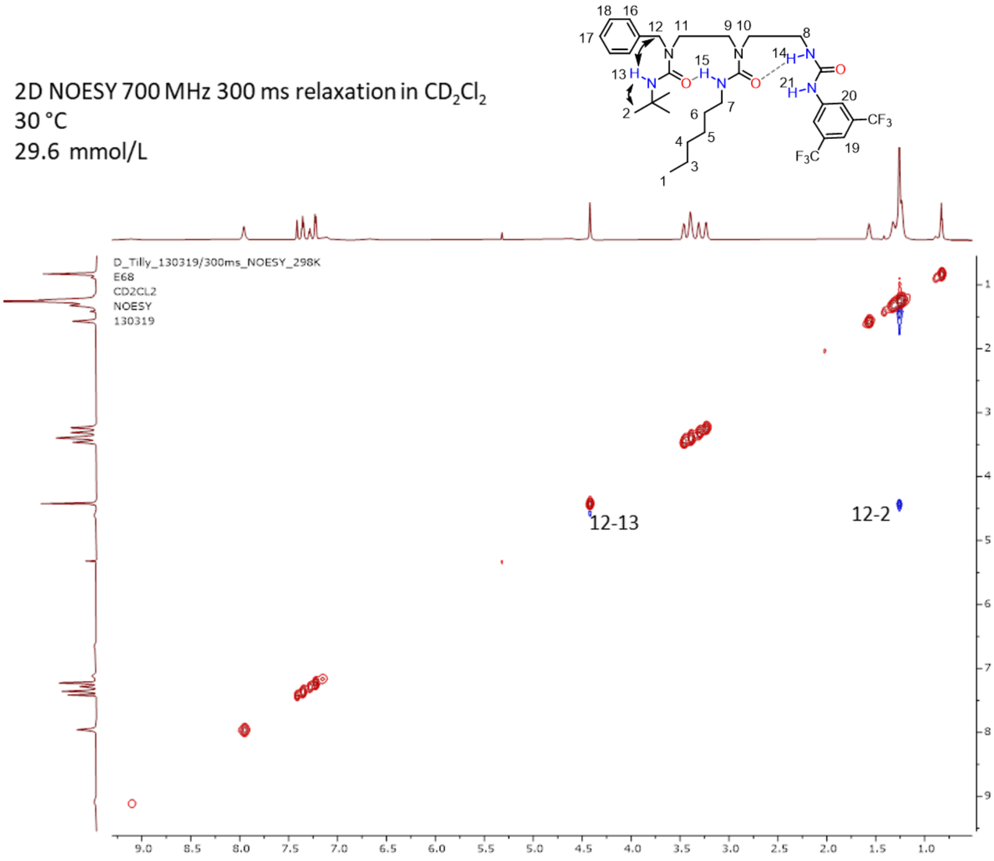

Supplementary Figure 61. ^{1}H 2D NOESY NMR spectrum of compound **3** in CD_2Cl_2 at 30 °C (700 MHz).

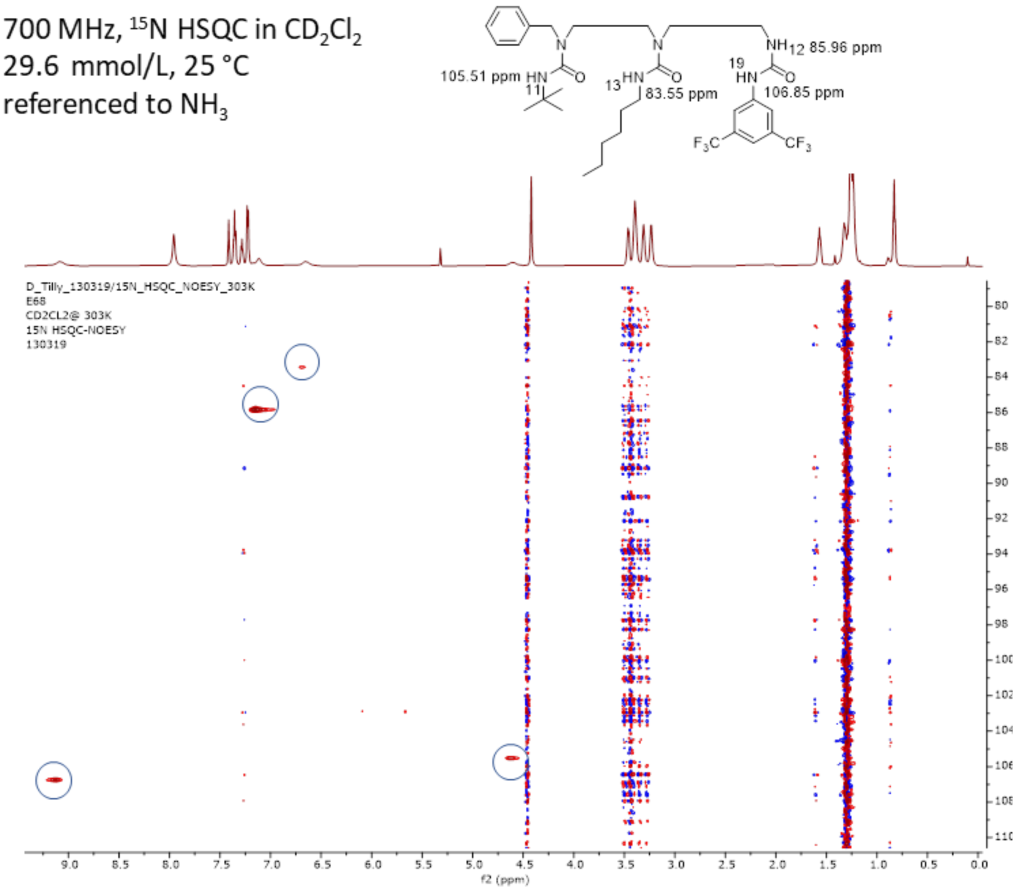

Supplementary Figure 62. ^{1}H-^{15}N HSQC NMR spectrum of compound **3** (29.6 mM in CD_2Cl_2) at 25 °C (700 MHz) (referenced to NH_3).

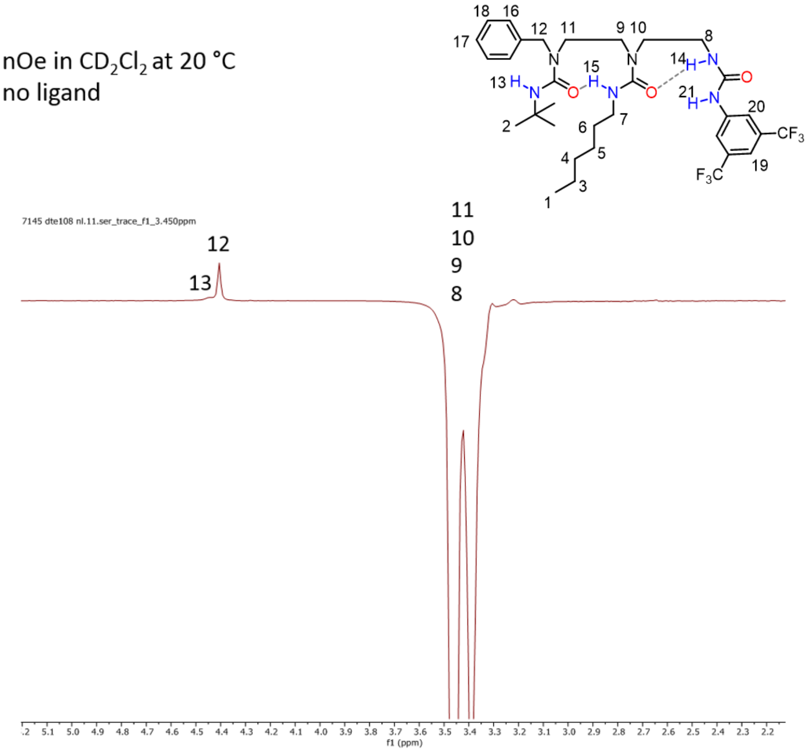

Supplementary Figure 63. ^{1}H NOE NMR spectrum of compound **3** in CD_2Cl_2 at 25 °C (500 MHz), irradiation of H8/9/10/11.

5.3.2. Identification of hydrogen bond directionality preference

In CD_2Cl_2: the ^1H NMR chemical shift values of ureido NH^{21} (8.77 ppm), NH^{14} (6.80 ppm), NH^{15} (6.63 ppm), NH^{13} (4.44 ppm) at 25 °C in CD_2Cl_2 were compared to the ^1H NMR chemical shift values of published compounds having ureido NH in similar topological environments (Supplementary Figure 64). NH^{13} is not involved in a direct hydrogen bond with a carbonyl, NH^{15} and NH^{14} (chemical shifts values are downfield) are in hydrogen bonding with a carbonyl. NMR data are consistent with a well-defined conformation containing intramolecular hydrogen bonds as drawn. NOE experiment (Supplementary Figure 63) irradiating $H^{8/9/10/11}$ shows a correlation signal with H^{12} and a correlation signal of weak intensity with H^{13}, the weak intensity of it is indicative of a hydrogen bond directionality as drawn.

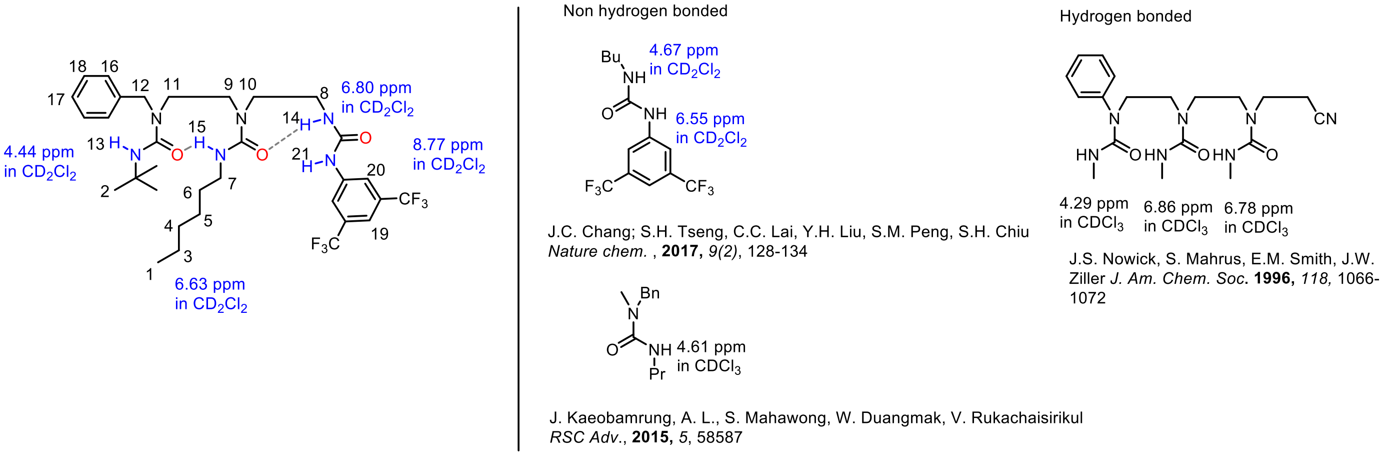

Supplementary Figure 64. ^1H NMR chemical shift values of ureido NH for compound **3** in CD_2Cl_2 at 25 °C and published values of ureido NH in similar topological environments.

5.3.3. Titration of compound **3** with tetrabutylammonium chloride in CD_2Cl_2 at 25 °C

(2) 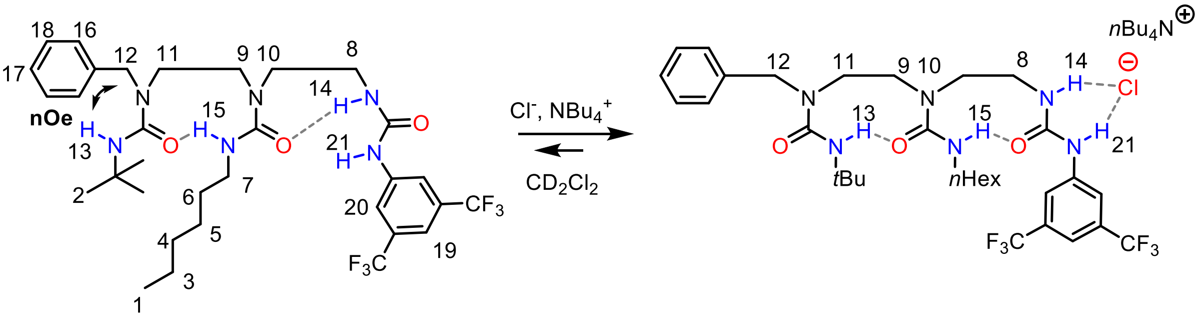

Titration of compound **3** (11.9 mM) in CD_2Cl_2 at 25 °C with increasing amounts of tetrabutylammonium chloride (from 0 to 2.69 equivalents) was monitored by ^1H NMR (Supplementary Figure 65). The ligand was added as a solid so no variation of concentration occurs during the titration. The values of chemical shifts of the NH of compound **3** upon addition of tetrabutylammonium chloride are collected in Supplementary Table 4 and plotted in a graph as {chemical shift of NH = f(ligand added)} (Supplementary Figure 67).

Progressive downfield shifts of both NH^{14} (CIS 0.94 ppm) and NH^{21} (CIS 1.67 ppm) signals are observed from 0 to 1 equivalent of ligand added, further addition of ligand has little effect on the chemical shifts.The observation is coherent with the formation of intermolecular hydrogen bonds between the ligand and the host at those positions. The upfield chemical shift variations of NH^{15} (CIS 0.39 ppm) upon addition of ligand is comparatively smaller, NH^{15} is involved in intramolecular hydrogen bonds with adjacent ureas no matter the directionality of the hydrogen bond chain so no important variation of chemical shift value is expected upon change of global hydrogen bond directionality. The signal for NH^{13} moves downfield (CIS 1.26 ppm), the chemical shifts vary from 0 to 1 equivalent of ligand added then stabilise even more ligand is added. It is coherent with a change of global directionality of the hydrogen bond chain controlled by the binding of chloride at the terminus of the chain.

Without ligand, 2D NOESY experiment on compound **3** in CD_2Cl_2 at 25 °C shows a correlation peak between NH^{13} and H^{12}, NOE experiment irradiating $H^{8/9/10/11}$ displays a correlation peak of weak intensity between H^{11} and NH^{13}, and a correlation peak between H^{11} and H^{12} (Supplementary Figures 61 - 63). In contrast, after addition of 2.7 equivalents of tetrabutylammonium chloride to the same NMR sample, the 2D NOESY data show no correlation peak is observed between NH^{13} and H^{12}, and a correlation peak of good intensity between NH^{13} and H^{11} (Supplementary Figure 69). Those results are consistent with a reversal in the global directionality of the hydrogen bond chain.

An association constant for the binding event of chloride ligand to NH^{14} and NH^{21} was estimated by non-linear curve fitting analysis of the experimental titration curves and comparing the results with theoretical binding isotherms for 1:1, 1:2, 2:1 binding modes using supramolecular.org (Supplementary Figures 67-68). The best fit is obtained for a 1:2 binding mode with $K_{1:1} = 1300 \pm 500\ M^{-1}$ and $K_{1:2} = 120 \pm 60\ M^{-1}$. Titration at lower concentrations to determine more accurately a binding constant value was not possible as NH signals become difficult to see at lower concentrations.

Mass spectrometry analysis of compound **3** + tetrabutylammonium chloride ligand (1.5 equiv.) using Synapt G2S Waters nanospray TOF MS ES+ experiment shows the formation of a 1:1 complex between **3** and the ligand. **HR – MS** (ES, positive ion mode) – *m/z* for $[C_{64}H_{116}F_6ClN_8O_3]^+$ 1193.8763, observed 1193.8768 (Supplementary Figure 70).

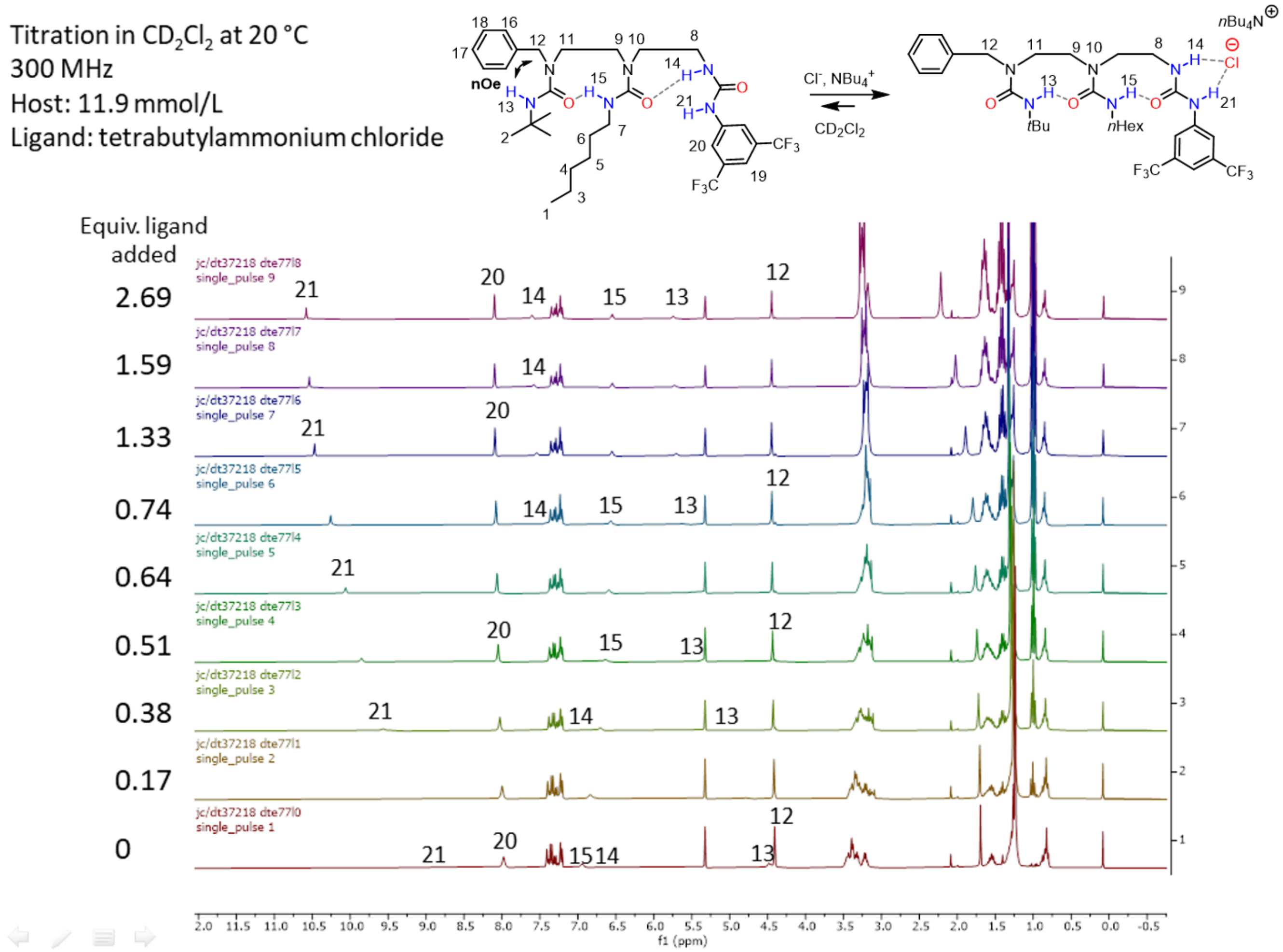

Supplementary Figure 65. Overlay of ^1H NMR spectra of compound **3** (11.9 mM in CD_2Cl_2) in the presence of increasing amounts of tetrabutylammonium chloride from 0 to 2.69 equivalents at 25 °C (300 MHz).

Supplementary Table 4. Chemical shifts of ^{1}H NH signals recorded during the NMR titration of **3** (11.9 mM in CD_2Cl_2) with increments of tetrabutylammonium chloride from 0 to 2.69 equivalents at 25 °C (300 MHz).

Concentration of host (mol/L)	Concentration of chloride ligand (mol/L)	Chemical shift NH^{21} (ppm)	Chemical shift NH^{14} (ppm)	Chemical shift NH^{15} (ppm)	Chemical shift NH^{13} (ppm)
0.01187	0	8.91	6.66	6.94	4.48
0.01187	0.002018	9.16	6.79	6.83	4.76
0.01187	0.00451	9.56	7.02	6.7	5.14
0.01187	0.00605	9.85	7.2	6.63	5.35
0.01187	0.0076	10.06	7.3	6.59	5.51
0.01187	0.0088	10.26	7.42	6.57	5.61
0.01187	0.0158	10.47	7.54	6.55	5.7
0.01187	0.0189	10.54	7.58	6.55	5.72
0.01187	0.032	10.58	7.6	6.55	5.74
**CIS (ppm)**		**1.66**	**0.94**	**-0.39**	**1.26**

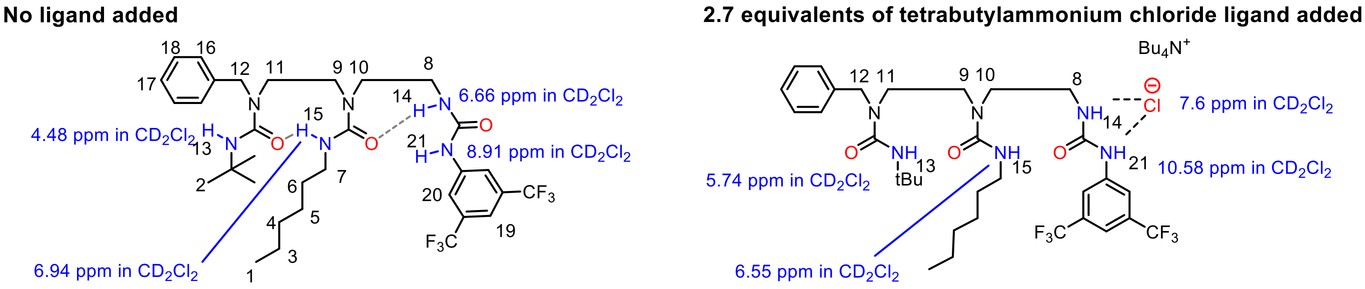

Supplementary Figure 66. ^{1}H NMR chemical shifts of compound **3** NH signals (17.8 mM in CD_2Cl_2) before and after addition of 2.7 equivalents of tetrabutylammonium chloride at 25 °C.

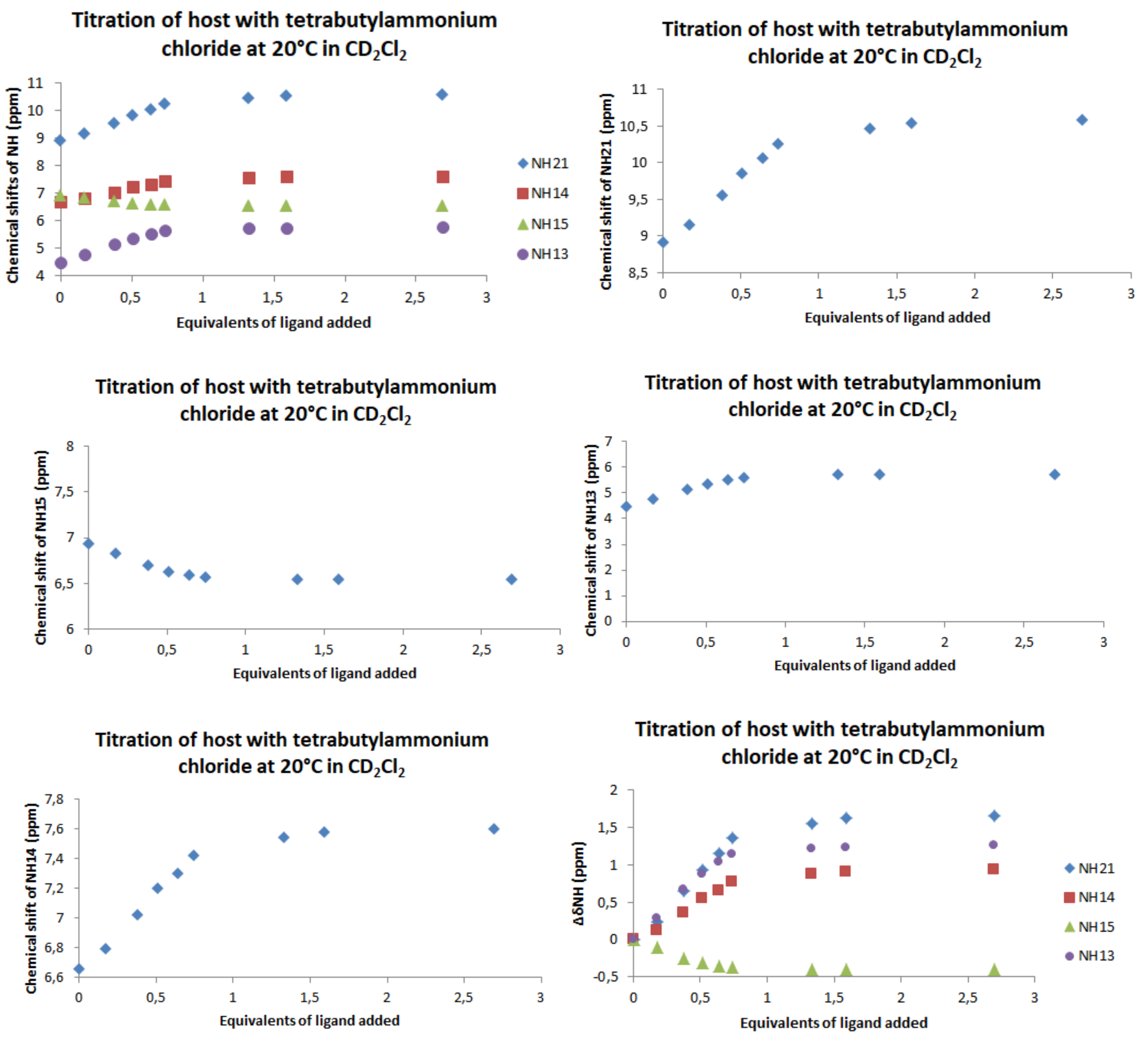

Supplementary Figure 67. Plots of variation of chemical shifts of NH signals of compound **3** (11.9 mM in CD_2Cl_2), and plot of variation of chemical induced shifts of NH signals upon addition of increments of tetrabutylammonium chloride from 0 to 2.69 equivalents, recorded by ^{1}H NMR at 25 °C (300 MHz).

a)

Details

**Time to fit**	0.2214 s
**SSR**	1.6434
**Fitted datapoints**	45
**Fitted params**	6

Parameters

**Parameter (bounds)**	**Optimised**	**Error**	**Initial**
**K** (0 → ∞)	305.43 M⁻¹	± 33.8625 %	100.00 M⁻¹

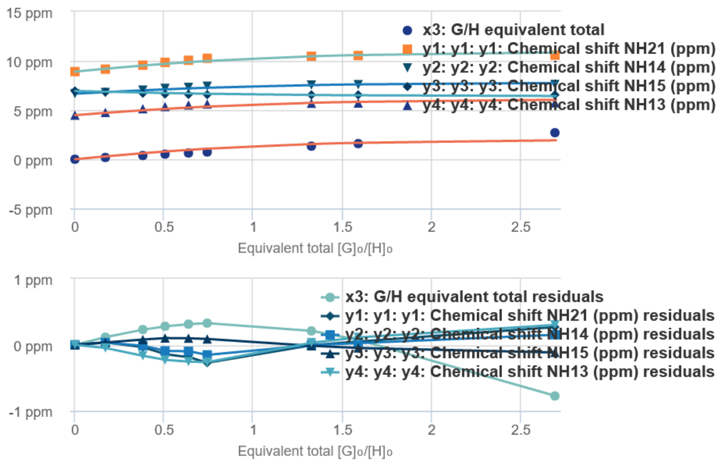

b)

Details

**Time to fit**	1.2746 s
**SSR**	0.1179
**Fitted datapoints**	36
**Fitted params**	10

Parameters

**Parameter (bounds)**	**Optimised**	**Error**	**Initial**
K_{11} (0 → ∞)	1255.99 M⁻¹	± 40.7558 %	1000.00 M⁻¹
K_{12} (0 → ∞)	121.90 M⁻¹	± 50.3673 %	100.00 M⁻¹

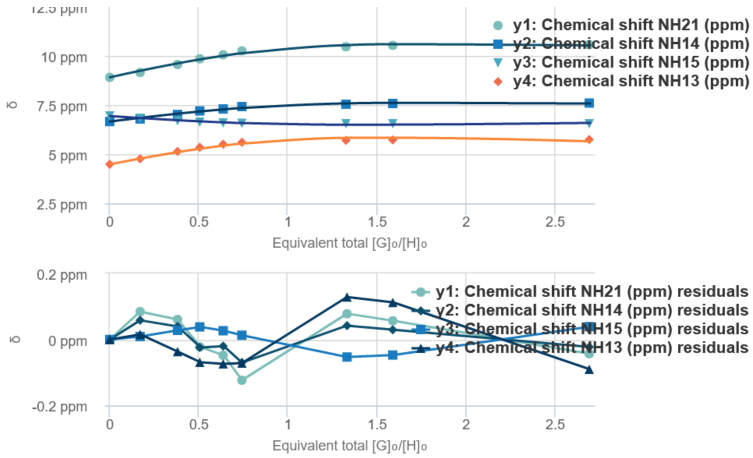

Supplementary Figure 68. Non-linear curve fitting analysis of the experimental titration data of compound **3** (11.9 mM in CD_2Cl_2) with increments of tetrabutylammonium chloride recorded at 25 °C (400 MHz) using a theoretical binding isotherm for a) 1:1 Source data are provided as a Source Data file: http://app.supramolecular.org/bindfit/view/9eab6e9c-9cbd-43d7-941f-71828f7fe6f6 and b) 1:2 binding http://app.supramolecular.org/bindfit/view/92a35920-8c98-4d4b-878b-e79ce7193360

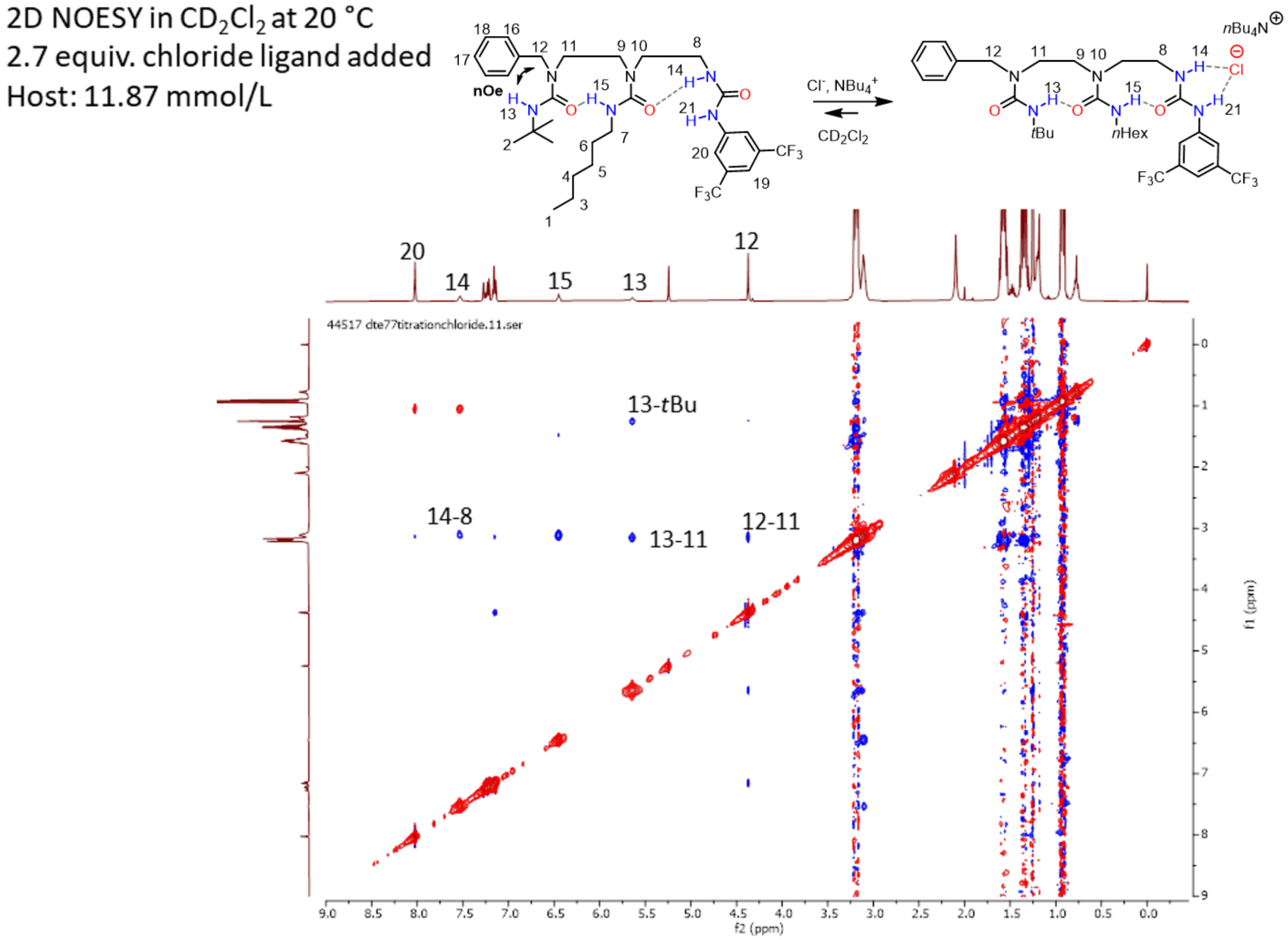

Supplementary Figure 69. ^{1}H-^{1}H 2D NOESY NMR spectrum of compound **3** (11.9 mM in CD_2Cl_2) in the presence of 2.7 equivalents of tetrabutylammonium chloride at 25 °C (500 MHz).

a

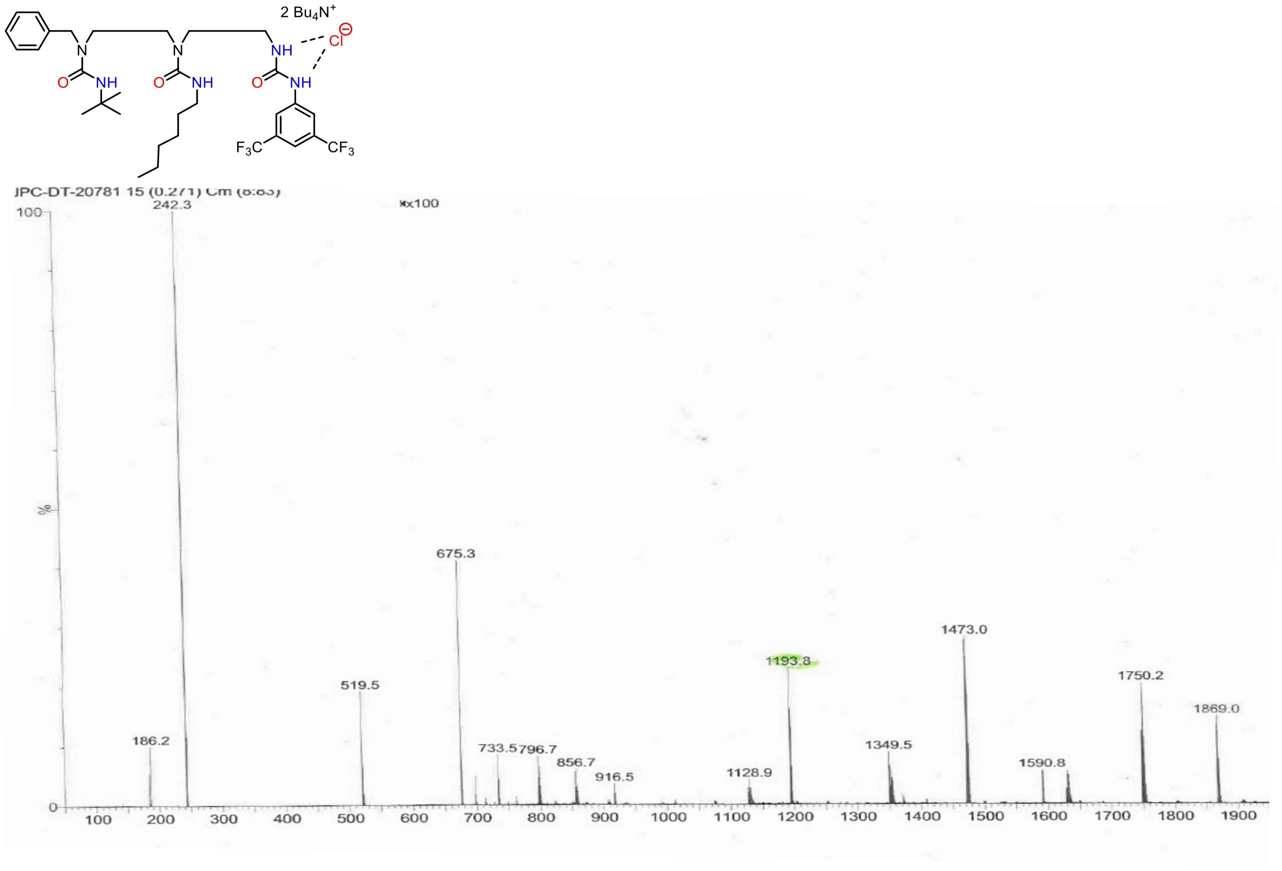

b

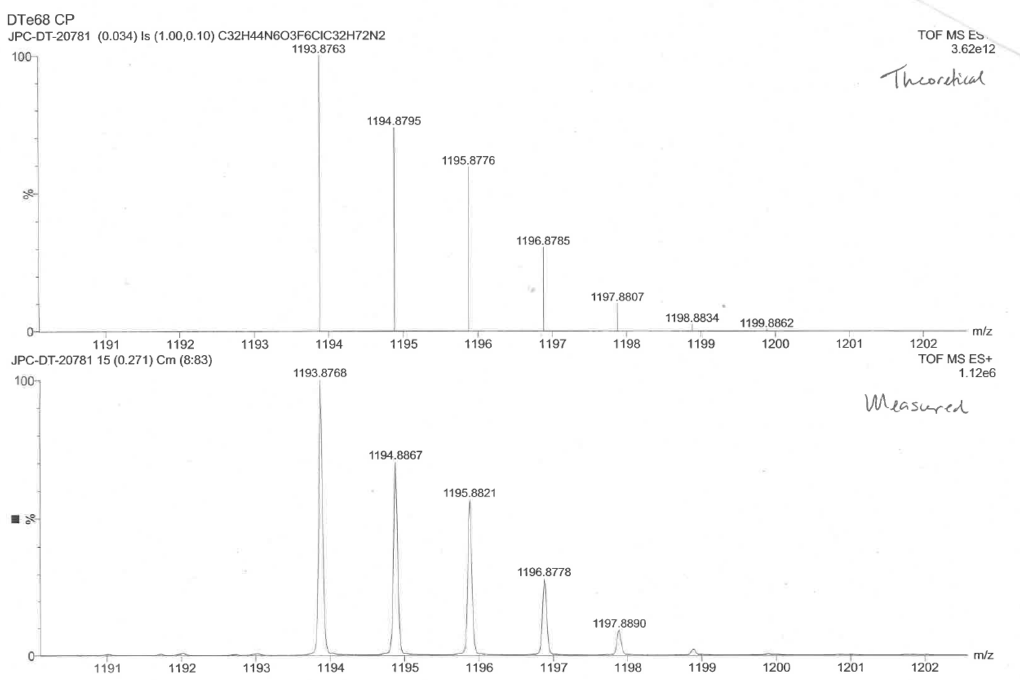

Supplementary Figure 70. a) ESI-MS (+ve) spectrum of compound **3** + Cl^-, 2 Bu_4N^+, b) experimental and simulated isotopic distribution of mass spectrum of **3** + Cl^-, 2 Bu_4N^+.

5.3.4. Titration of compound **3** with tetrabutylammonium iodide in CD_2Cl_2 at 25 °C

(3) 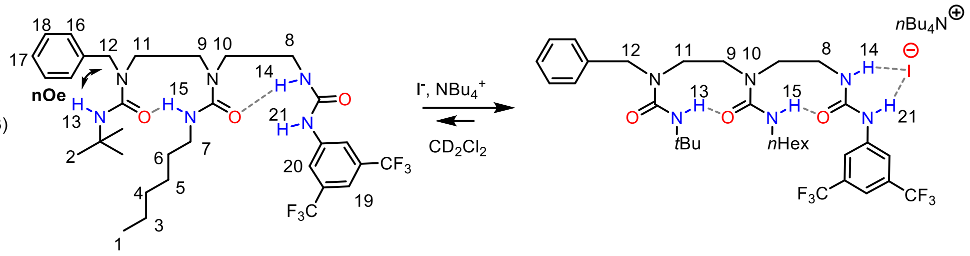

Titration of compound **3** (14.8 mM) in CD_2Cl_2 at 25 °C with increasing amounts of tetrabutylammonium iodide (from 0 to 5 equivalents) was monitored by ^{1}H NMR (Supplementary Figure 71). The ligand was added as a solid so no variation of concentration occurs during the titration. The values of chemical shifts of the NH of compound **3** upon addition of tetrabutylammonium iodide are collected in Supplementary Table 5 and plotted in a graph as {chemical shift of NH = f(ligand added)} (Supplementary Figure 72).

Progressive downfield shifts of both NH^{14} (CIS 0.32 ppm) and NH^{21} (CIS 0.5 ppm) signals are observed from 0 to 5 equivalent of ligand added. The observation is coherent with the formation of weak intermolecular hydrogen bonds between the ligand and the host at those positions. The signal for NH^{13} moves downfield (CIS 1.09 ppm), coherent with a change of global directionality of the hydrogen bond chain controlled by the binding of iodide at the terminus of the chain.

An association constant for the binding event of chloride ligand to NH^{14} and NH^{21} was estimated by non-linear curve fitting analysis of the experimental titration curves and comparing the results with theoretical binding isotherms for 1:1, 1:2, 2:1 binding modes using supramolecular.org (Supplementary Figure 73). The best fit is obtained for a 1:1 binding mode with K = 150 ± 20 M^{-1}. Titration at lower concentrations to determine more accurately a binding constant value was not possible as NH signals become difficult to see at lower concentrations.

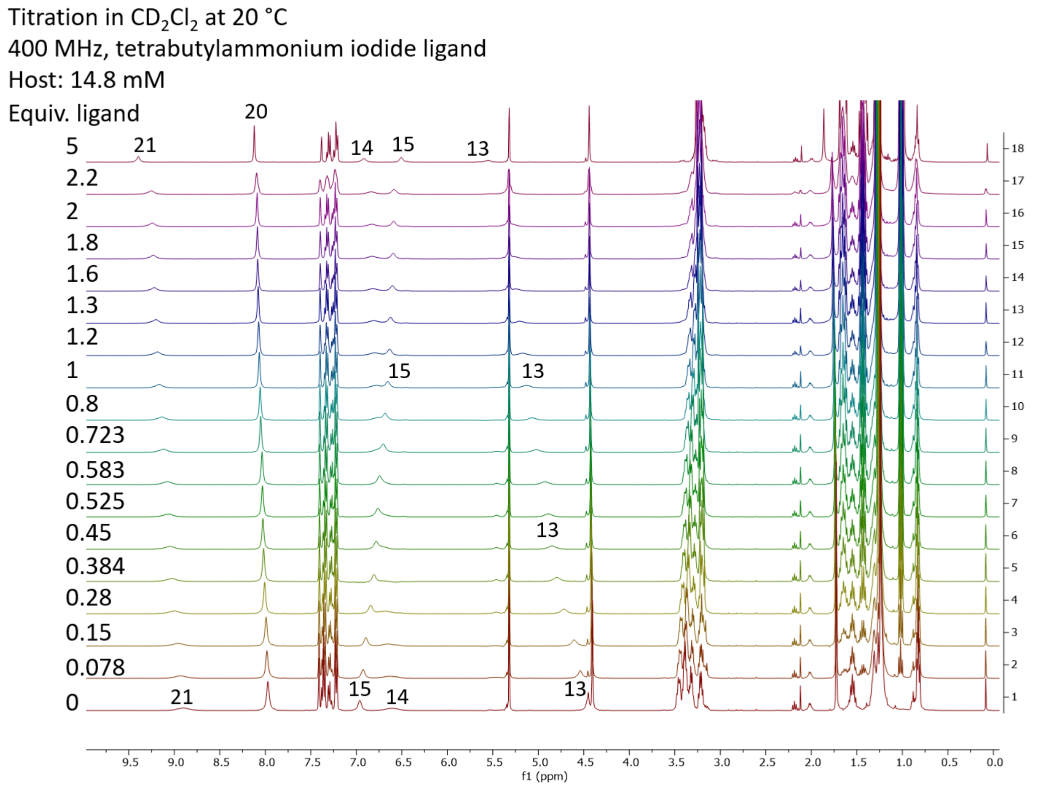

Supplementary Figure 71. Overlay of ^{1}H NMR spectra of compound **3** (14.8 mM in CD_2Cl_2) in the presence of increasing amounts of tetrabutylammonium iodide from 0 to 5 equivalents at 25 °C (400 MHz)

Supplementary Table 5. Chemical shifts of ^{1}H NH signals recorded during the NMR titration of **3** (14.8 mM in CD_2Cl_2) with increments of tetrabutylammonium iodide from 0 to 5 equivalents at 25 °C (400 MHz).

Concentration of host (mol/L)	Equivalents of iodide ligand	Concentration of iodide ligand (mol/L)	Chemical shift NH^{21} (ppm)	Chemical shift NH^{14} (ppm)	Chemical shift NH^{15} (ppm)	Chemical shift NH^{13} (ppm)
0.0148	0	0	8.9	6.6	6.96	4.46
0.0148	0.078	0.00115	8.93	6.63	6.93	4.54
0.0148	0.15	0.00222	8.96	6.65	6.89	4.6
0.0148	0.28	0.00414	8.99	6.68	6.84	4.72
0.0148	0.384	0.00568	9.02	6.7	6.8	4.8
0.0148	0.45	0.00667	9.05		6.78	4.85
0.0148	0.525	0.00778	9.06		6.76	4.89
0.0148	0.583	0.0086	9.08		6.74	4.92
0.0148	0.723	0.0107	9.12		6.71	5.02
0.0148	0.8	0.01184	9.13		6.68	5.07
0.0148	1	0.0148	9.17	6.78	6.65	5.12
0.0148	1.2	0.01776	9.18	6.79	6.64	5.17
0.0148	1.4	0.02072	9.2	6.81	6.62	5.2
0.0148	1.6	0.02368	9.22	6.82	6.6	5.24
0.0148	1.8	0.02664	9.23	6.83	6.6	
0.0148	2	0.0296	9.24	6.83	6.59	
0.0148	2.2	0.03256	9.25	6.83	6.59	
0.0148	5	0.074	9.4	6.92	6.51	5.55
**CIS (ppm)**			**0.5**	**0.32**	**-0.45**	**1.09**

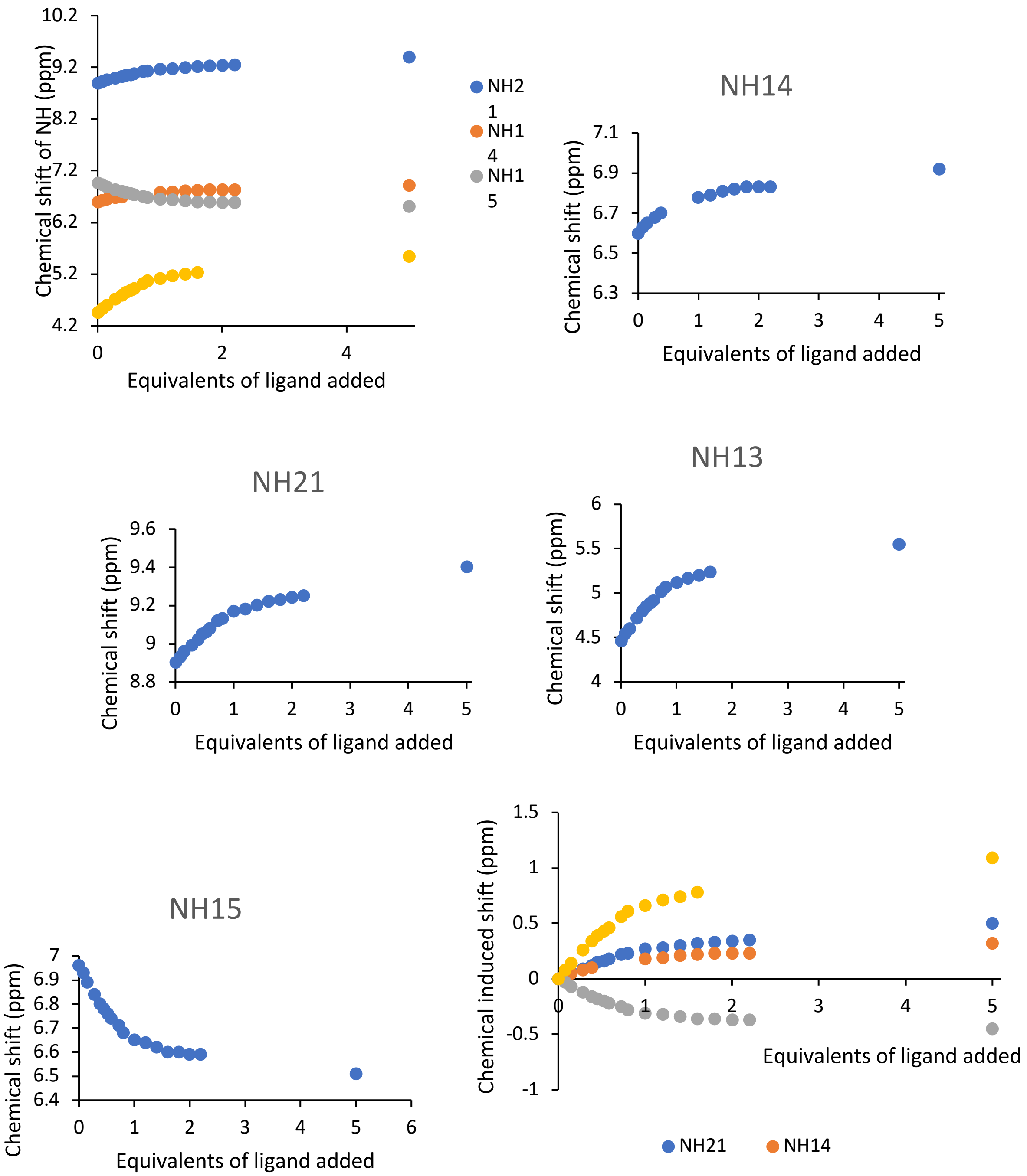

Supplementary Figure 72. Plots of variation of chemical shifts of NH signals of compound **3** (14.8 mM in CD_2Cl_2), and plot of variation of chemical induced shifts of NH signals upon addition of increments of tetrabutylammonium iodide from 0 to 5 equivalents, recorded by ^1H NMR at 25 °C (400 MHz).

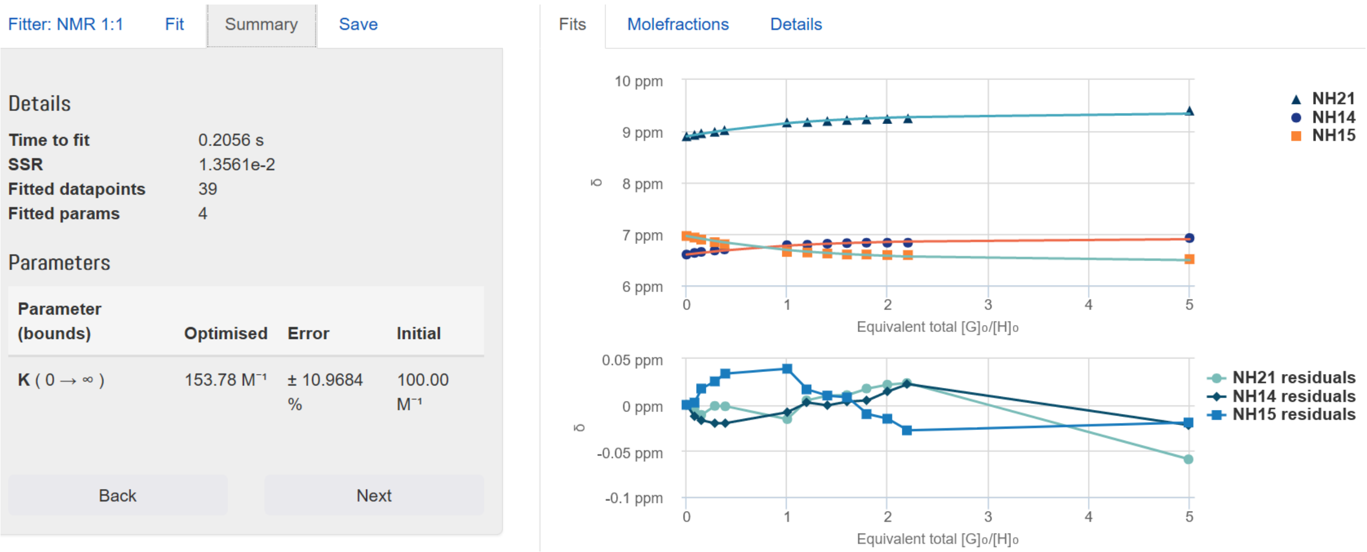

Supplementary Figure 73. Non-linear curve fitting analysis of the experimental titration data of compound **3** (14.8 mM in CD_2Cl_2) with increments of tetrabutylammonium iodide recorded at 25 °C (400 MHz) using a theoretical binding isotherm for 1:1. Source data are provided as a Source Data file. http://app.supramolecular.org/bindfit/view/7881d9dd-6dd6-422e-8a6d-a5262e95b2a6

5.3.5. Titration of compound **3** with tetrabutylammonium bromide in CD_2Cl_2 at 25 °C

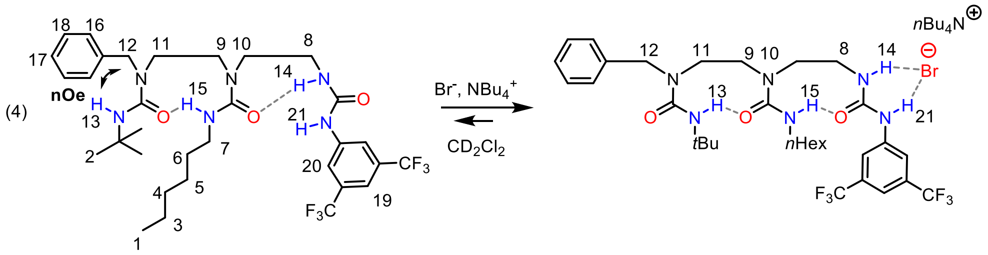

Titration of compound **3** (14.8 mM) in CD_2Cl_2 at 25 °C with increasing amounts of tetrabutylammonium bromide (from 0 to 5 equivalents) was monitored by ^{1}H NMR (Supplementary Figure 74). The ligand was added as a solid so no variation of concentration occurs during the titration. The values of chemical shifts of the NH of compound **3** upon addition of tetrabutylammonium bromide are collected in Supplementary Table 6 and plotted in a graph as {chemical shift of NH = f(ligand added)} (Supplementary Figure 75).

Progressive downfield shifts of both NH^{14} (CIS 0.55 ppm) and NH^{21} (CIS 1.19 ppm) signals are observed from 0 to 5 equivalent of ligand added. The observation is coherent with the formation of intermolecular hydrogen bonds between the ligand and the host at those positions. The signal for NH^{13} moves downfield (CIS 1.26 ppm), coherent with a change of global directionality of the hydrogen bond chain controlled by the binding of iodide at the terminus of the chain.

An association constant for the binding event of chloride ligand to NH^{14}, NH^{21} and NH^{15} was estimated by non-linear curve fitting analysis of the experimental titration curves and comparing the results with theoretical binding isotherms for 1:1, 1:2, 2:1 binding modes using supramolecular.org (Supplementary Figure 76). The best fit is obtained for a 1:1 binding mode with K = 170 ± 20 M^{-1}. Titration at lower concentrations to determine more accurately a binding constant value was not possible as NH signals become difficult to see at lower concentrations.

Titration in CD_2Cl_2 at 20 °C
400 MHz, tetrabutylammonium bromide ligand
Host: 14.8 mM

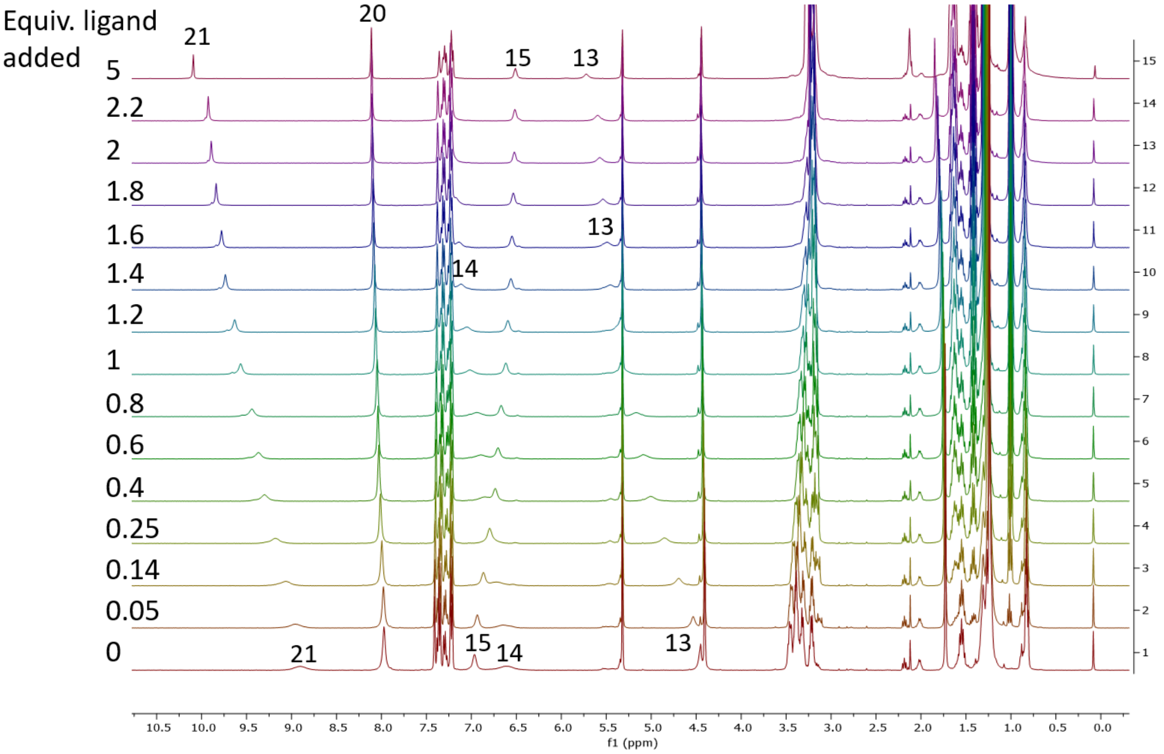

Supplementary Figure 74. Overlay of ^1H NMR spectra of compound **3** (14.8 mM in CD_2Cl_2) in the presence of increasing amounts of tetrabutylammonium bromide from 0 to 5 equivalents at 25 °C (400 MHz).

Supplementary Table 6. Chemical shifts of ^1H NH signals recorded during the NMR titration of **3** (14.8 mM in CD_2Cl_2) with increments of tetrabutylammonium bromide from 0 to 5 equivalents at 25 °C (400 MHz).

Concentration of host (mol/L)	Equivalents of bromide ligand	Concentration of bromide ligand (mol/L)	Chemical shift NH^{21} (ppm)	Chemical shift NH^{14} (ppm)	Chemical shift NH^{15} (ppm)	Chemical shift NH^{13} (ppm)
0.0148	0	0	8.9	6.61	6.97	4.46
0.0148	0.05	0.00074	8.95	6.64	6.94	4.53
0.0148	0.14	0.002072	9.06	6.72	6.86	4.69
0.0148	0.25	0.0037	9.18		6.79	4.85
0.0148	0.4	0.00592	9.3	6.85	6.73	5
0.0148	0.6	0.00888		6.89	6.7	5.09
0.0148	0.8	0.01184	9.44	6.94	6.67	5.16
0.0148	1	0.0148	9.57	7.01	6.62	
0.0148	1.2	0.01776	9.63	7.05	6.59	
0.0148	1.4	0.02072	9.74	7.11	6.56	5.46
0.0148	1.6	0.02368	9.78	7.14	6.55	5.49
0.0148	1.8	0.02664	9.84	7.17	6.53	5.54
0.0148	2	0.0296	9.89		6.52	5.57
0.0148	2.2	0.03256	9.93		6.51	5.59
0.0148	5	0.074	10.09		6.51	5.72
**CIS (ppm)**			**1.19**		**-0.46**	**1.26**

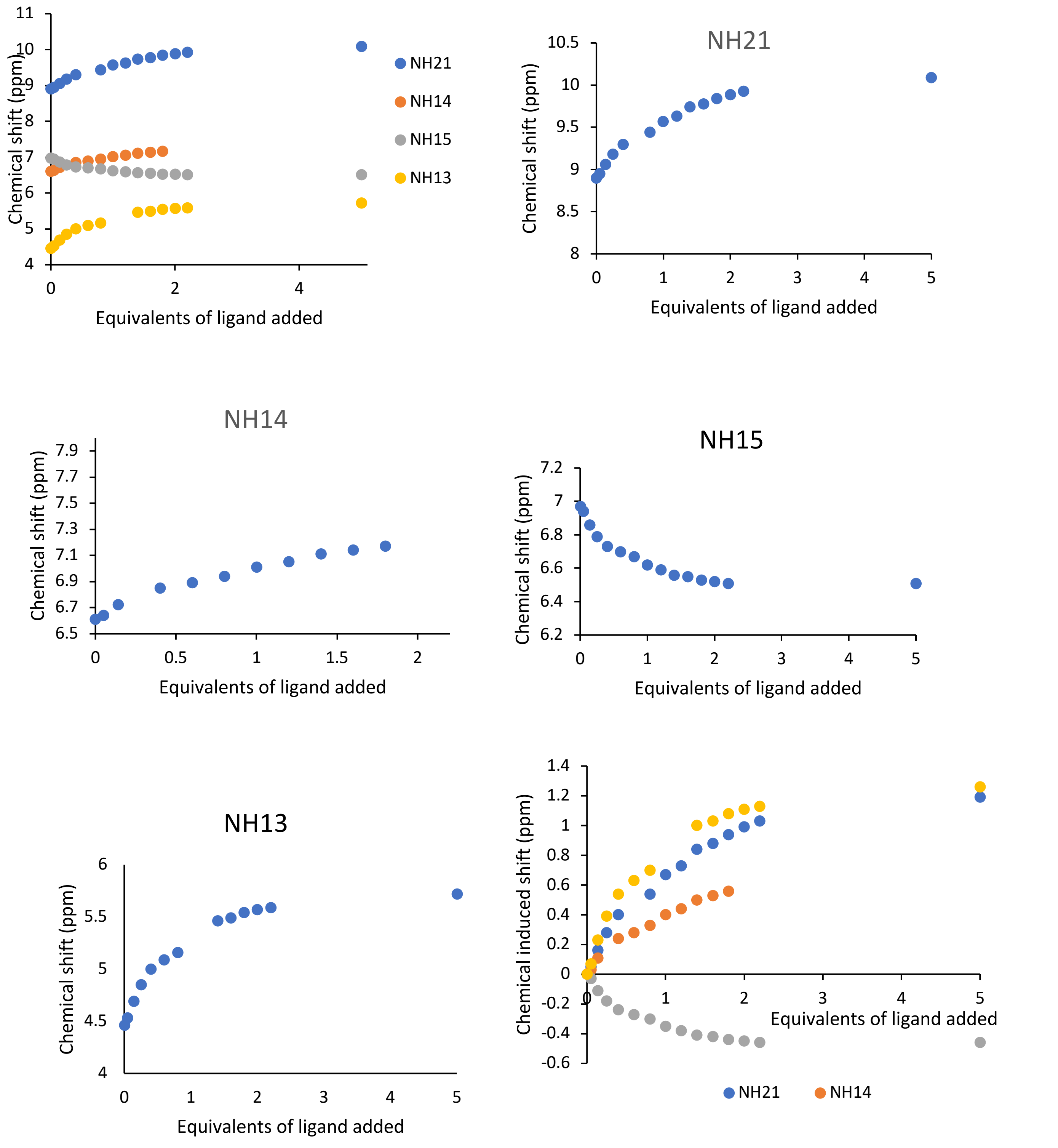

Supplementary Figure 75. Plots of variation of chemical shifts of NH signals of compound **3** (14.8 mM in CD_2Cl_2), and plot of variation of chemical induced shifts of NH signals upon addition of increments of tetrabutylammonium bromide from 0 to 5 equivalents, recorded by ^{1}H NMR at 25 °C (400 MHz).

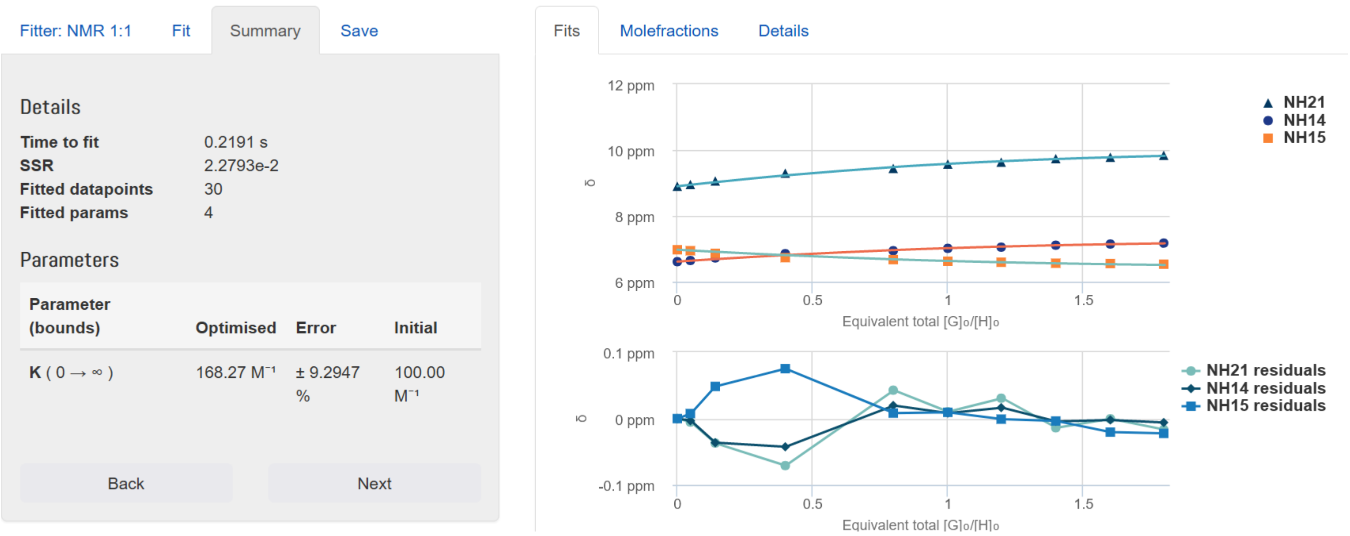

Supplementary Figure 76. Non-linear curve fitting analysis of the experimental titration data of compound **3** (14.8 mM in CD_2Cl_2) with increments of tetrabutylammonium bromide recorded at 25 °C (400 MHz) using a theoretical binding isotherm for 1:1 binding. Source data are provided as a Source Data file. http://app.supramolecular.org/bindfit/view/a53fb236-bc01-445e-a386-765a6e563791

5.3.6. Titration of compound 3 with tetrabutylammonium nitrate in CD_2Cl_2 at 25 °C

(5)

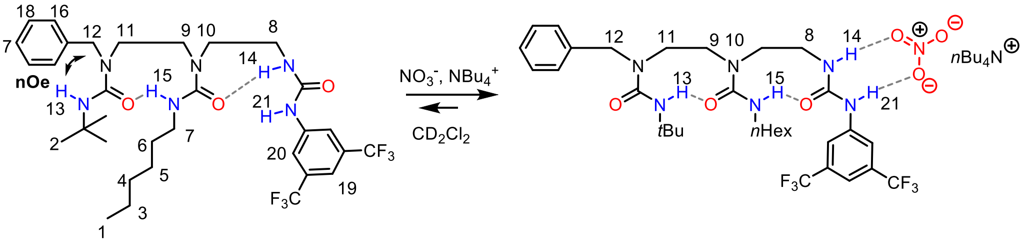

Titration of compound **3** (14.8 mM) in CD_2Cl_2 at 25 °C with increasing amounts of tetrabutylammonium nitrate (from 0 to 5 equivalents) was monitored by ^1H NMR (Supplementary Figure 77). The ligand was added as a solid so no variation of concentration occurs during the titration. The values of chemical shifts of the NH of compound **3** upon addition of tetrabutylammonium nitrate are collected in Supplementary Table 7 and plotted in a graph as {chemical shift of NH = f(ligand added)} (Supplementary Figure 78).

Progressive downfield shifts of both NH^{14} (CIS 0.87 ppm) and NH^{21} (CIS 0.88 ppm) signals are observed from 0 to 5 equivalent of ligand added. The observation is coherent with the formation of intermolecular hydrogen bonds between the ligand and the host at those positions. The signal for NH^{13} moves downfield (CIS 1.28 ppm), coherent with a change of global directionality of the hydrogen bond chain controlled by the binding of iodide at the terminus of the chain.

An association constant for the binding event of chloride ligand to NH^{21} was estimated by non-linear curve fitting analysis of the experimental titration curves and comparing the results with theoretical binding isotherms for 1:1, 1:2, 2:1 binding modes using supramolecular.org (Supplementary Figure 79). The best fit is obtained for a 1:1 binding mode with K = 500 ± 100 M^{-1}. Titration at lower concentrations to determine more accurately a binding constant value was not possible as NH signals become difficult to see at lower concentrations.

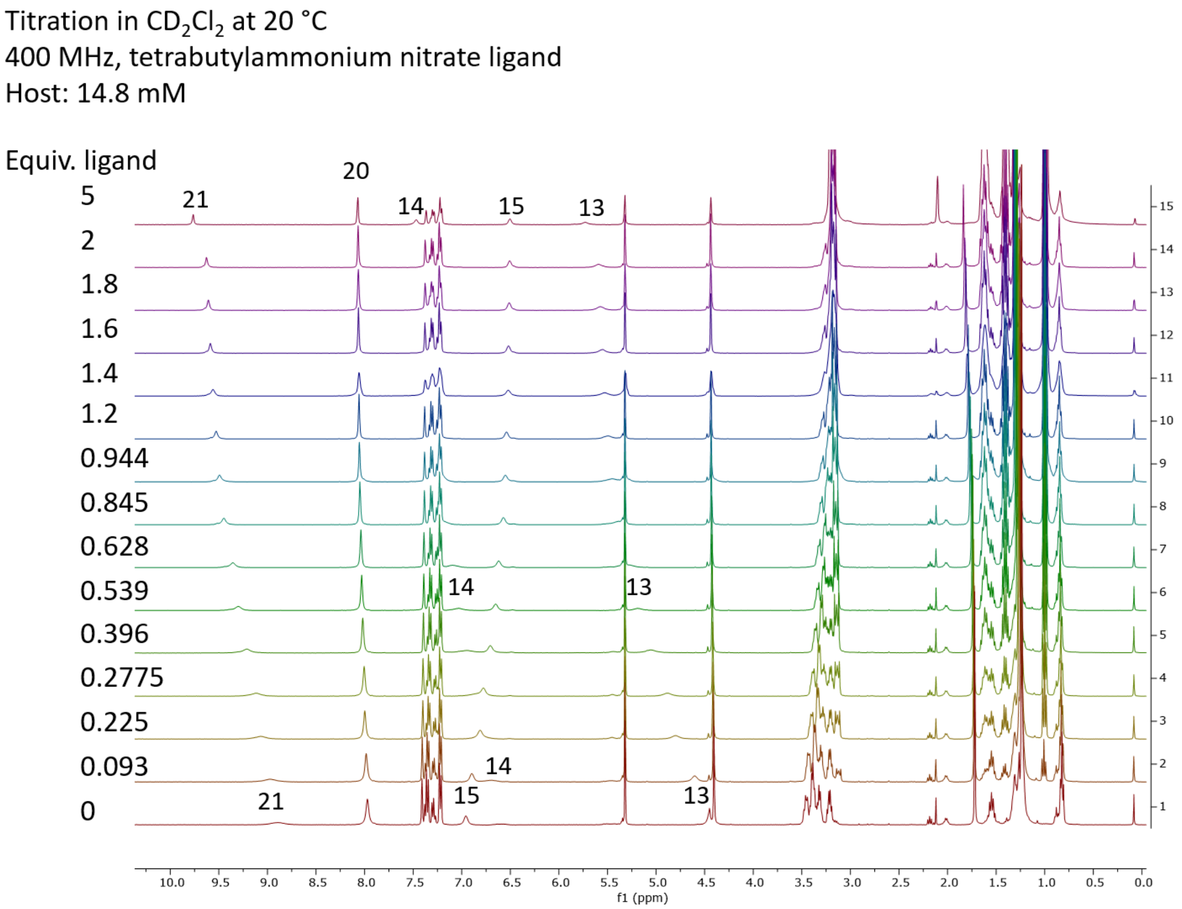

Supplementary Figure 77. Overlay of ^{1}H NMR spectra of compound **3** (14.8 mM in CD_2Cl_2) in the presence of increasing amounts of tetrabutylammonium nitrate from 0 to 5 equivalents at 25 °C (400 MHz).

Supplementary Table 7. Chemical shifts of ^{1}H NH signals recorded during the NMR titration of **3** (14.8 mM in CD_2Cl_2) with increments of tetrabutylammonium nitrate from 0 to 5 equivalents at 25 °C (400 MHz).

Concentration of host (mol/L)	Equivalents of nitrate ligand	Concentration of nitrate ligand (mol/L)	Chemical shift NH^{21} (ppm)	Chemical shift NH^{14} (ppm)	Chemical shift NH^{15} (ppm)	Chemical shift NH^{13} (ppm)
0.0148	0	0	8.89	6.6	6.96	4.45
0.0148	0.092567568	0.00137	8.97	6.7	6.9	4.6
0.0148	0.225	0.00333	9.07		6.81	4.8
0.0148	0.277027027	0.0041	9.11		6.78	4.89
0.0148	0.395945946	0.00586	9.21	6.94	6.7	5.05
0.0148	0.539189189	0.00798	9.3	7.03	6.66	5.19
0.0148	0.628378378	0.0093	9.35	7.1	6.62	5.28
0.0148	0.844594595	0.0125	9.45		6.57	5.39
0.0148	0.945945946	0.014	9.49		6.55	5.45
0.0148	1.2	0.01776	9.53		6.54	5.49
0.0148	1.398648649	0.0207	9.56		6.53	5.53
0.0148	1.6	0.02368	9.59		6.52	5.55
0.0148	1.8	0.02664	9.61		6.51	5.57
0.0148	2	0.0296	9.63		6.51	5.59
0.0148	5	0.074	9.77	7.47	6.51	5.73
**CIS (ppm)**			**0.88**	**0.87**	**-0.32**	1.28

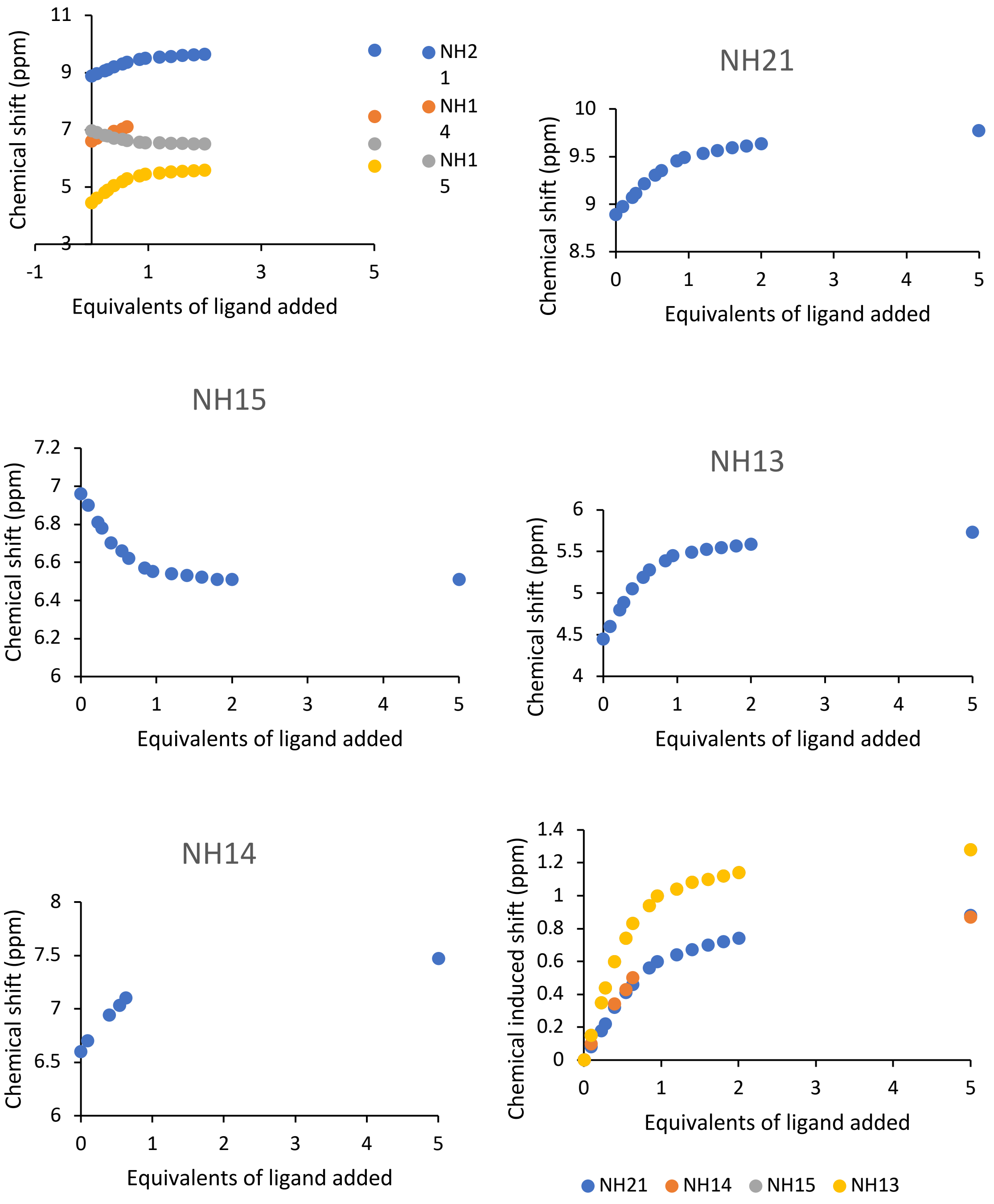

Supplementary Figure 78. Plots of variation of chemical shifts of NH signals of compound **3** (14.8 mM in CD_2Cl_2), and plot of variation of chemical induced shifts of NH signals upon addition of increments of tetrabutylammonium nitrate from 0 to 5 equivalents, recorded by ^{1}H NMR at 25 °C (400 MHz).

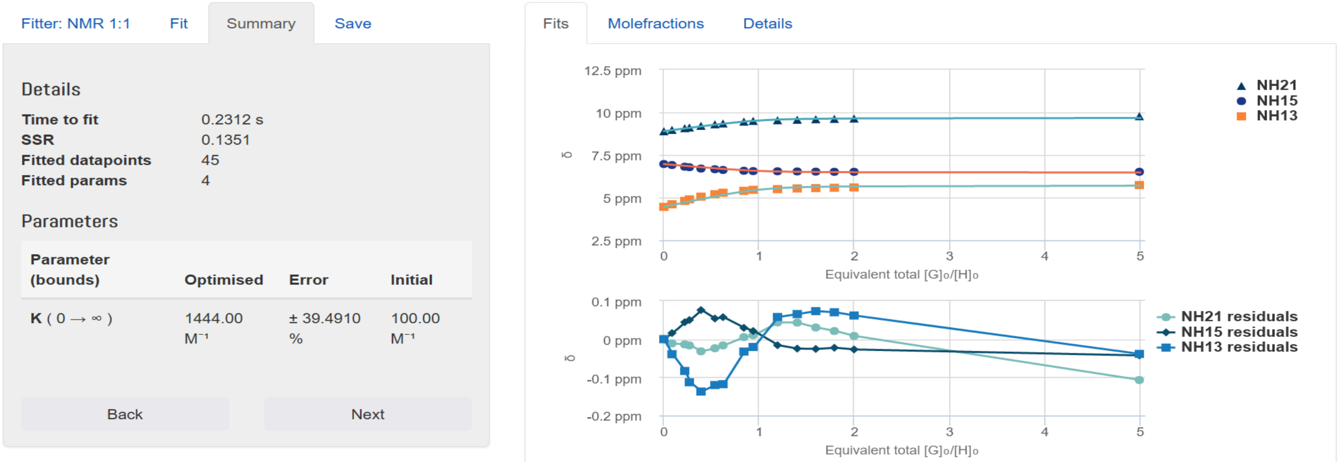

Supplementary Figure 79. Non-linear curve fitting analysis of the experimental titration data of compound **3** (14.8 mM in CD_2Cl_2) with increments of tetrabutylammonium nitrate recorded at 25 °C (400 MHz) using a theoretical binding isotherm for 1:1 binding). Source data are provided as a Source Data file. http://app.supramolecular.org/bindfit/view/3943732a-8e62-47a4-a8a5-17759c335ae2

5.4. Control NMR experiments on diurea **SI5** and tris(urea) **SI6**

5.4.1. Titration of diurea **SI5** with tetrabutylammonium chloride in CD_2Cl_2 at 25 °C

^{1}H NMR spectrum of compound **SI5** (27.6 mM) in CD_2Cl_2 at 25 °C displays one signal at 5.36 ppm for the two ureido NH, the chemical shift value is the weighted average of the chemical shifts of ureido NH in hydrogen bonding and in non-hydrogen-bonding modes (rapid equilibrium between directionalities on the NMR timescale, Supplementary Figure 80).

A titration of that solution with increasing amounts of tetrabutylammonium chloride (from 0 to 3.6 equivalents) was monitored by ^{1}H NMR analysis (Supplementary Figure 80). The ligand was added as a solid so no variation of concentration occurs during the titration. Addition of tetrabutylammonium chloride causes only minimal variation of the chemical shift values of the ureido NH signals compared to the chemical shift variation observed during the titrations of compound **3** (complexation induced shift CIS = 0.16 ppm for **SI5** at 1.4 equivalents of ligand, whereas downfield shifts of 1.5 ppm observed for the terminal ureido NH during the titrations of **3** with the same quantity of ligand). Thus, the interaction between tetrabutylammonium chloride and **SI5** under the titration conditions, is weak.

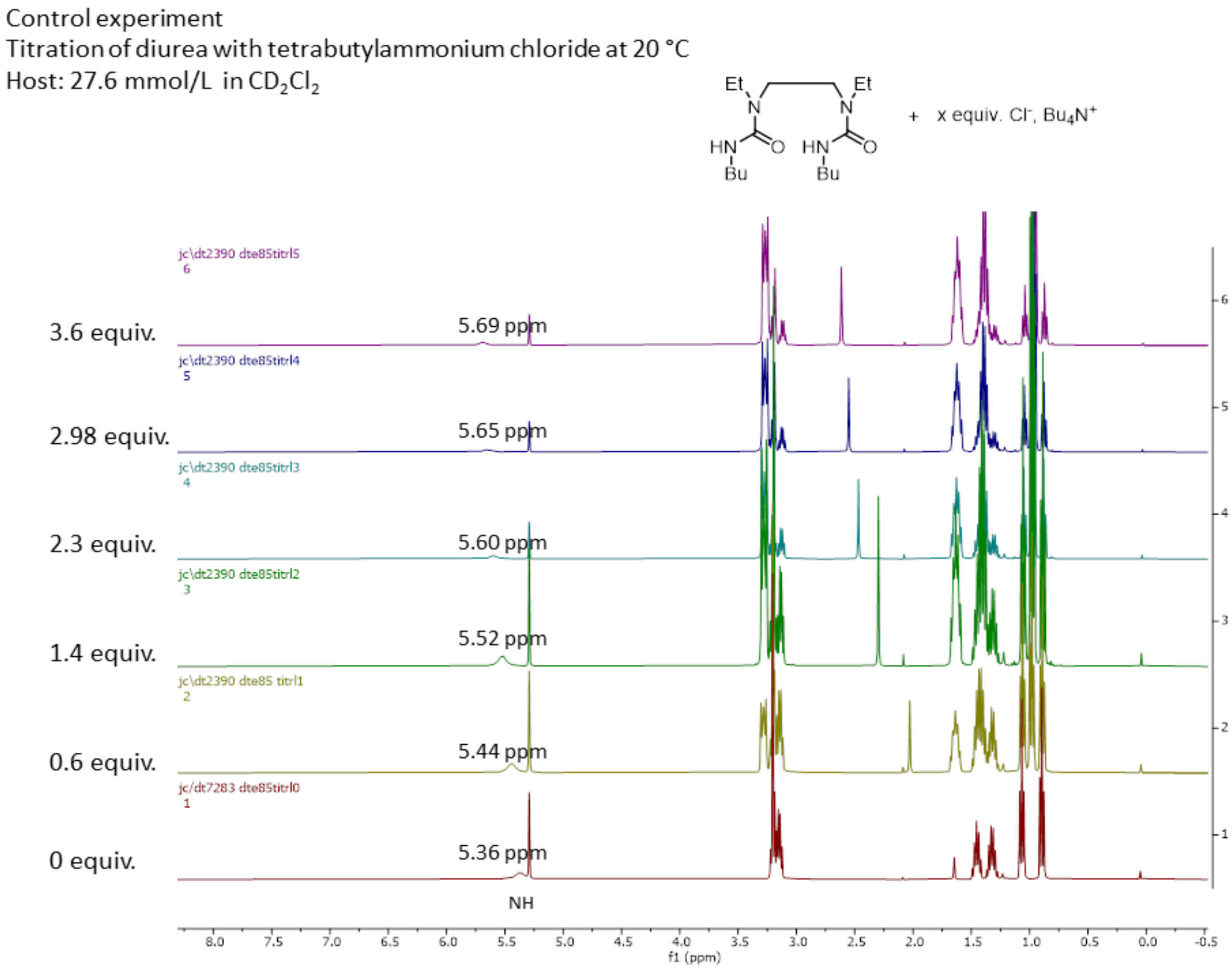

Supplementary Figure 80. Overlay of ^{1}H NMR spectra of compound **SI5** (27.6 mM in CD_2Cl_2) in the presence of increasing amounts of tetrabutylammonium chloride from 0 to 3.6 equivalents at 25 °C (300 MHz).

5.4.2. Titration of compound SI6 with tetrabutylammonium chloride in CD_2Cl_2 at 25 °C

^{1}H NMR spectrum of compound compound **SI6** (17.2 mM) in CD_2Cl_2 at 25 °C (Supplementary Figure 81) displays one triplet signal for NH^a at 6.63 ppm, and a large signal centred at 5.44 ppm in the baseline for the ureido NH^b located at the termini of the hydrogen bond chain; that value of chemical shift is the weighted average of the chemical shifts of NH^b in hydrogen bonding and in non-hydrogen-bonding modes (rapid equilibria between hydrogen bond directionalities on the NMR timescale at ambient temperature). Titration of that solution with tetrabutylammonium chloride (from 0 to 1.6 equivalents) was monitored by ^{1}H NMR analysis. The ligand was added as a solid so no variation of concentration occurs during the titration. The addition of tetrabutylammonium chloride to the solution causes only minimal chemical shift variation of the NH^a signal (0.09 ppm at 1.6 equivalents of ligand added). The signal of NH^b stay broad in the baseline during the titration.

The chemical shift variation of NH^b signals when adding Bu_4NCl is weak compared to the shifts observed for the titrations of **3** (complexation induced shift (CIS) = 0.14 ppm for **SI6** at 0.9 equivalents of ligand, compared to downfield shifts in the order of 1.5 ppm for the terminal ureido NH during the titrations of **3** with the same quantity of ligand). Thus, the interaction between tetrabutylammonium chloride and **SI6** under the titration conditions, is weak.

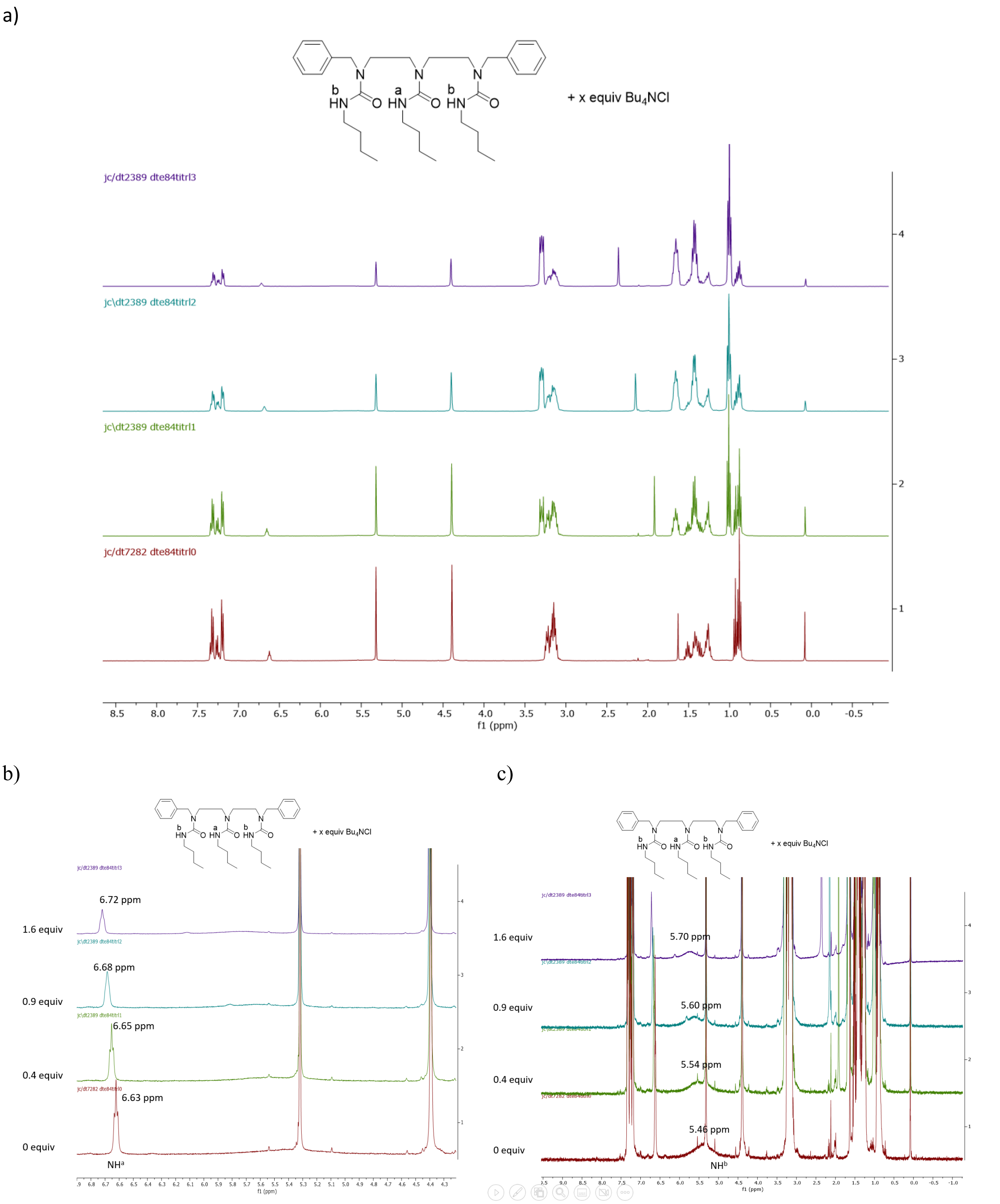

Supplementary Figure 81. Overlay of ^{1}H NMR spectra of compound **SI6** (17.2 mM in CD_2Cl_2) in the presence of increasing amounts of tetrabutylammonium chloride from 0 to 1.6 equivalents at 25 °C (300 MHz) (A) full spectra, B) expansion of NH^a, C) expansion for NH^b).

6. Deprotonation of 2,4-dinitrophenol

UV light absorbance spectra were recorded at room temperature on a JASCO V-660 spectrophotometer using the following parameters: bandwidth is 0.1 nm, response is set to medium, data pitch is 0.5 nm, scan speed is 1000 nm/min, baseline correction is applied using dry dichloromethane as the blank. The cuvette is 1 mm path length, its volume is 0.4 mL. The artifact at 370 nm in the spectra correspond to a change in the light source of the spectrophotometer.

6.1. Titration of 2,4-dinitrophenol with imidazole in dry dichloromethane

6.1.1. UV-visible spectroscopy titration of 2,4-dinitrophenol with imidazole

- A stock solution of 2,4-dinitrophenol 10 mM in dry dichloromethane was prepared by dissolving 2,4-dinitrophenol (0.92 mg, 0.005 mmol) in 0.5 mL dry dichloromethane.

- A stock solution of imidazole in dry dichloromethane (220.6 mM) was prepared by dissolving imidazole (60 mg, 0.882 mmol) in 4 mL of dry dichloromethane.

The titrated solution of 2,4-dinitrophenol (1.16 mM in the cuvette) was prepared by diluting 0.033 mL of 2,4-dinitrophenol stock solution (10 mM) with 0.25 mL of dry dichloromethane in the cuvette, resulting in a 1.16 mM solution of 2,4-dinitrophenol (0.00033 mmol of 2,4-dinitrophenol is present in the cuvette).

The titration proceeds by successive addition of 0.0015 mL of the imidazole 220.6 mM stock solution each time (0.0015 mL is 1 equivalent of imidazole, 0.00033 mmol) to the 2,4-dinitrophenol solution in the cuvette (1.16 mM), agitation of the resulting solution, and recording of the UV light absorbance spectrum 5 minute after each addition. UV light absorbance intensities shown in UV titration spectra are not corrected for changes in concentration - the substrate concentrations decreased from 1.16 mM at the start of the titration to 1.10 mM at the completion of the titration. No insoluble material was formed during the titration in the cuvette.

(7)

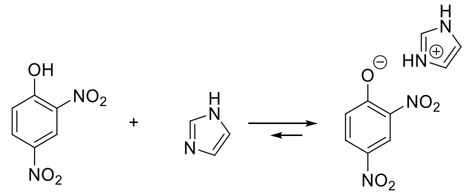

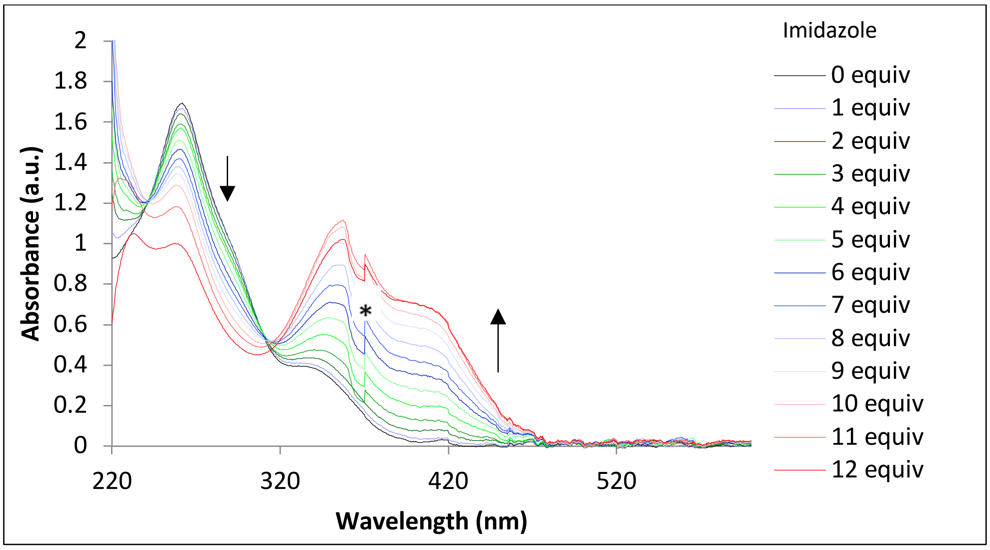

Supplementary Figure 82. Stacked UV light absorbance spectra recorded during the titration of 2,4-dinitrophenol (1.16 mM in dry dichloromethane) with increments of imidazole (from 0 to 12 equivalents), * indicates the bulb changeover artifact between 350-375 nm. Source data are provided as a Source Data file.

Upon incremental addition of imidazole from 0 to 12 equivalents to the 2,4-dinitrophenol (1.16 mM in dry dichloromethane), the colour of the solution in the cuvette visibly changes from colourless at 0 equivalent of imidazole to increasingly yellow, a colour characteristic of 2,4-dinitrophenolates. The stacked UV light absorbance spectra recorded during the titration show the presence of two isobestic points at 242 nm and 315 nm. The UV light absorbance progressively increases at wavelengths between 330 -440 nm (violet light) upon progressive addition of imidazole, while a progressive decrease in the UV light absorbance is observed simultaneously at wavelengths between 250-300 nm during the titration. Changes in UV light absorbance essentially occur between 0 and 10 equivalents of added imidazole: further addition of imidazole from 10 to 11 equivalents leads to no more change in the UV light absorbance spectra. Addition of one more equivalent of imidazole to 12 equivalents decreases the UV light absorbance at wavelengths between 330-440 nm (as opposed to the increase observed from 0 to 10 equivalents) and the UV light absorbance curve does not participate to the isobestic points, indicating a third species in equilibrium upon further addition of imidazole. Control experiments consisting of recording the UV light absorbance spectra of imidazole alone at concentrations of 1 mM and 10 mM in dry dichloromethane (Supplementary Figure 84) show that imidazole does not have any UV light absorbance at wavelengths between 250-520 nm at the concentrations of imidazole used during the titration. Another control experiment is the UV light absorbance spectrum of tetrabutylammonium 2,4-dinitrophenolate (1.25 mM in dry dichloromethane), maxima of UV light absorbance at wavelengths of 350-390 nm and 400-450 nm (Supplementary Figure 83) are identical to those observed during the titration (Supplementary Figure 82). Overall, the titration data are consistent with the progressive deprotonation of acidic 2,4-dinitrophenol by imidazole to form imidazolium 2,4-dinitrophenolate reaching completion of equilibrium displacement at 10 equivalents of added imidazole.

6.1.2. Control experiments

- UV light absorbance of tetrabutylammonium 2,4-dinitrophenolate (1.25 mM in dry dichloromethane)

A 5 mM stock solution of tetrabutylammonium 2,4-dinitrophenolate was obtained by mixing 0.412 mg (0.002 mmol) of sodium 2,4-dinitrophenolate with tetrabutylammonium chloride (0.556 mg, 0.002 mmol) in dry dichloromethane (0.4 mL) and agitating the solution for 5 minutes.

A 1.25 mM tetrabutylammonium 2,4-dinitrophenolate solution in dry dichloromethane was obtained by diluting 0.075 mL (0.000375 mmol) of the 5 mM stock solution with 0.225 mL of dry dichloromethane in the cuvette then agitation. To note, sodium 2,4-dinitrophenolate is not soluble in dry dichloromethane at the concentration, the tetrabutylammonium counterion contributes to the solubility of the ion pair.

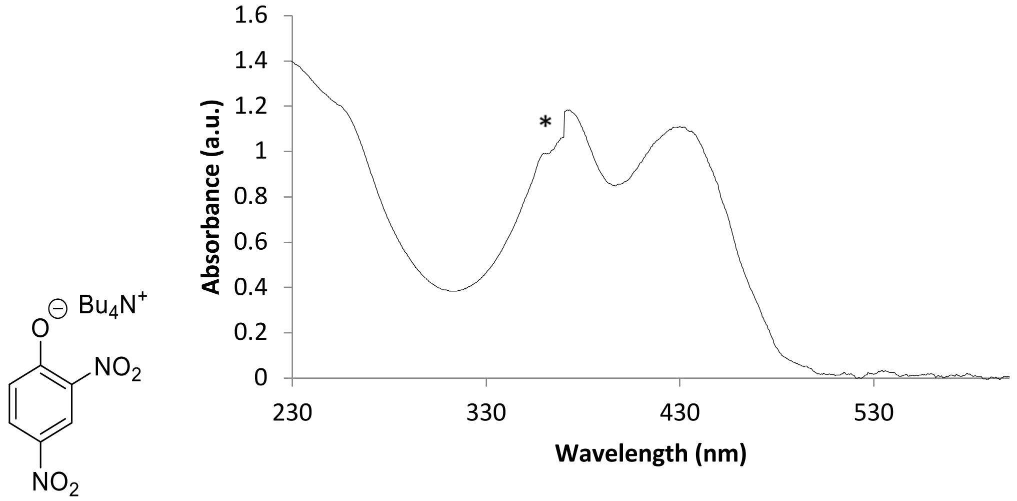

Supplementary Figure 83. UV light absorbance spectrum of tetrabutylammonium 2,4-dinitrophenolate (0.25 mM in dry dichloromethane), * indicates the bulb changeover artifact between 350-375 nm. Source data are provided as a Source Data file.

- **UV light absorbance spectrum of imidazole (1 mM and 10 mM in dry dichloromethane)**

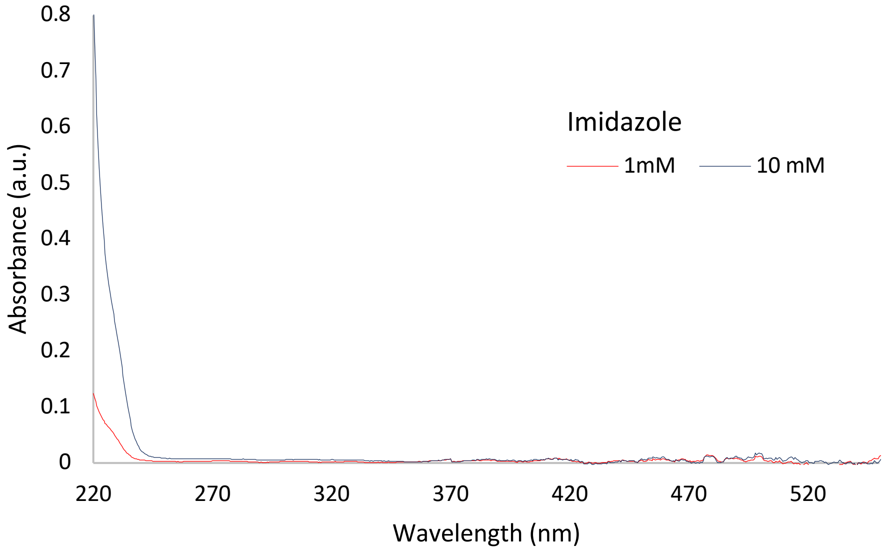

Supplementary Figure 84: UV light absorbance spectra of imidazole (1 mM and 10 mM in dry dichloromethane). Imidazole does not lead to UV light absorbance at wavelengths between 250-520 nm at the concentrations of the studies. Source data are provided as a Source Data file.

6.2. Titration of 2,4-dinitrophenol with compound **1** in dry dichloromethane

6.2.1. UV-visible spectroscopy titration of 2,4-dinitrophenol with compound **1**

- A stock solution of **1** (81 mM in dry dichloromethane) was prepared by dissolving 32.45 mg (0.0417 mmol) of compound **1** in 0.515 mL of dry dichloromethane.

The titrated solution of 2,4-dinitrophenol 1.16 mM was prepared by diluting 0.033 mL of 2,4-dinitrophenol stock solution (10 mM) with 0.25 mL of dry dichloromethane in the cuvette, resulting in a 1.16 mM solution of 2,4-dinitrophenol (0.00033 mmol of 2,4-dinitrophenol in the cuvette).

Titration with compound **1** proceeds by successive addition of 0.004 mL of the compound **1** stock solution (81 mM) each time (0.004 mL is 1 equivalent of compound **1,** 0.00033 mmol) to the 2,4-dinitrophenol solution (1.16 mM) in the cuvette, agitation of the resulting solution, and recording of the UV light absorbance spectrum 5 minutes after each addition. UV light absorbance intensities shown in UV titration spectra are not corrected for changes in concentration - the substrate concentrations decreased from 1.16 mM at the start of the titration to 1.0 mM at the completion of the titration. No insoluble material was formed during the titration in the cuvette.

(8)

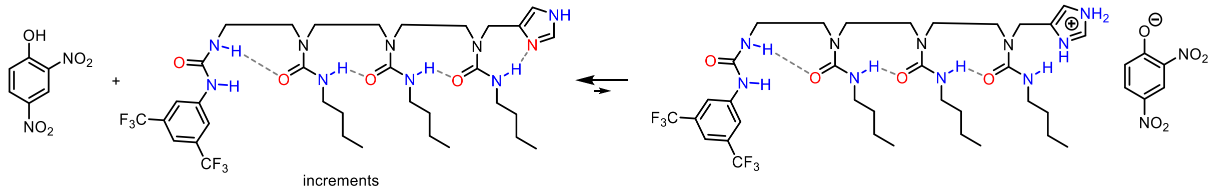

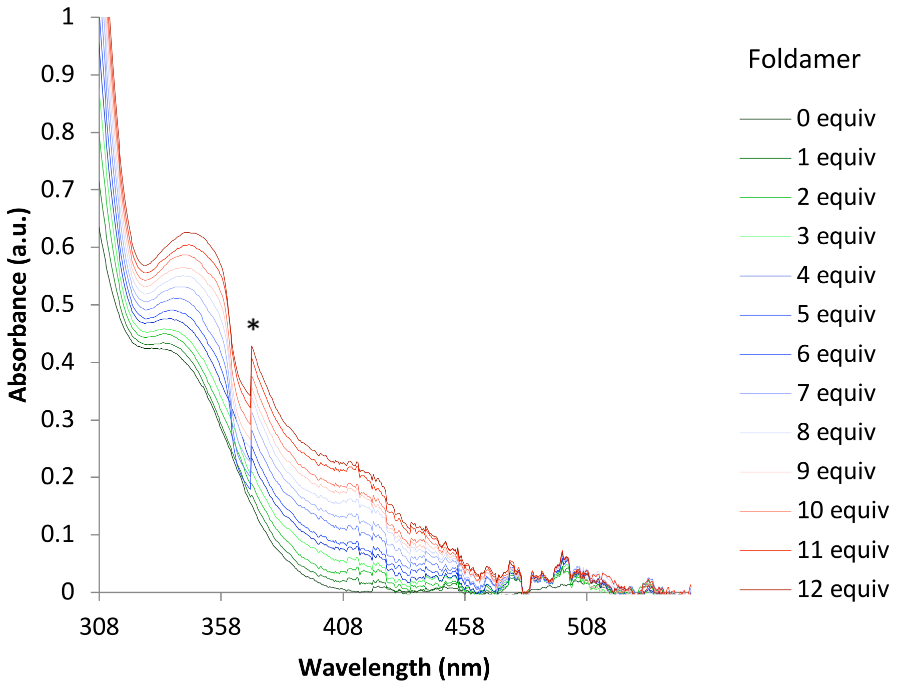

Supplementary Figure 85. Stacked UV light absorbance spectra recorded during the titration of 2,4-dinitrophenol (1.16 mM in dry dichloromethane) with increments of 1 from 0 to 12 equivalents, * indicates the bulb changeover artifact between 350-375 nm. Source data are provided as a Source Data file.Upon incremental addition of compound **1** (from 0 to 12 equivalents) to the 2,4-dinitrophenol (1.16 mM in dry dichloromethane), no colour change of the solution in the cuvette was visible; this observation contrasts with the colour change observed during the titration of 2,4-dinitrophenol at the same concentration with imidazole (a yellow colour developed upon addition of imidazole). It also contrasts with the visible yellow colour of tetrabutylammonium 2,4-dinitrophenolate solutions at the same concentrations in dichloromethane. The stacked UV light absorbance spectra recorded during the titration show continuous uniform progressive small increases in absorbance at all wavelengths between 450-220 nm upon progressive addition of increments of compound **1**; no isobestic point is observed. Strong UV light absorbance values (above 2 a.u.) are recorded at wavelengths below 300 nm upon addition of compound **1**. As a control experiment, the UV light absorbance spectrum of a solution of **1** 1.16 mM alone in dry dichloromethane (Supplementary Figure 86) shows strong absorbance at wavelengths below 300 nm. Since 1.16 mM concentration corresponds to 1 equivalent of added foldamer during the titration, the strong values of absorbance recorded below 300 nm during the titration are attributed to the absorbance of the foldamer only (12 equivalents of foldamer = 13.92 mM, Supplementary Figure 86). The uniform progressive small increases in absorbance recorded during the titration also are attributed to the absorbance of compound **1** only. The UV light absorbance spectrum of tetrabutylammonium 2,4-dinitrophenolate (1.25 mM in dry dichloromethane) shows maxima of UV light absorbance at wavelengths between 350-390 nm and 400-450 nm (Supplementary Figure 83), however no strong UV light absorbance at those wavelengths is observed during the titration using compound **1** (for example UV light absorbance values stays below 0.25 a.u. between 400-450 nm). Overall, the data indicate compound **1** does not deprotonate significantly 2,4-dinitrophenol under those conditions, this is in sharp contrast with the observed deprotonation of 2,4-dinitrophenol by imidazole in otherwise identical conditions.

6.2.2. Control experiments

- UV light absorbance spectrum of 1 (1.16 mM in dry dichloromethane) without then with tetrabutylammonium chloride (1 equivalent)

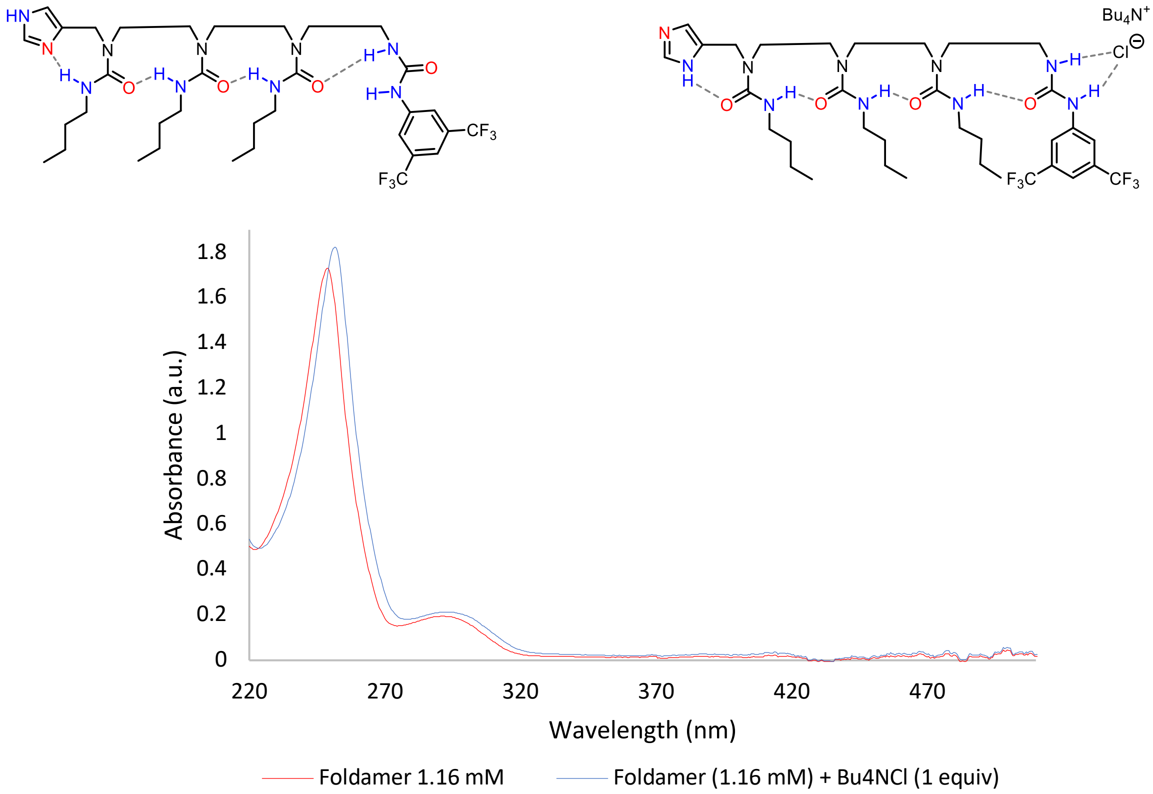

Supplementary Figure 86. Stacked UV light absorbance spectra of foldamer **1** (1.16 mM in dry dichloromethane) without and with tetrabutylammonium chloride (1 equivalent). Source data are provided as a Source Data file.Addition of tetrabutylammonium chloride (1 equivalent) to compound **1** (1.16 mM in dry dichloromethane) does not significantly alter the UV light absorbance of the resulting mixture.

6.3. Titration of 2,4-dinitrophenol with compound 2 in dry dichloromethane

6.3.1. UV-visible spectroscopy titration of 2,4-dinitrophenol with compound 2

- A stock solution of compound **2** (144 mM in dry dichloromethane) was prepared by dissolving 28.37 mg (0.072 mmol) of compound **2** in 0.5 mL of dry dichloromethane.

The titrated solution of 2,4-dinitrophenol 1.16 mM was prepared by diluting 0.033 mL of 2,4-dinitrophenol stock solution (10 mM) with 0.25 mL of dry dichloromethane in the cuvette, resulting in a 1.16 mM solution of 2,4-dinitrophenol (0.00033 mmol of 2,4-dinitrophenol in the cuvette).

Titration with compound **2** proceeds by successive addition of 0.0024 mL of the compound **2** stock solution (144 mM) each time (0.0024 mL is 1 equivalent of compound **2,** 0.00034 mmol) to the 2,4-dinitrophenol solution (1.16 mM) in the cuvette, agitation of the resulting solution, and recording of the UV light absorbance spectrum 5 minutes after each addition. UV light absorbance intensities shown in UV titration spectra are not corrected for changes in concentration - the substrate concentrations decreased from 1.16 mM at the start to 1.0 mM at the completion of the titration. No insoluble material was formed during the titration in the cuvette.

(9) 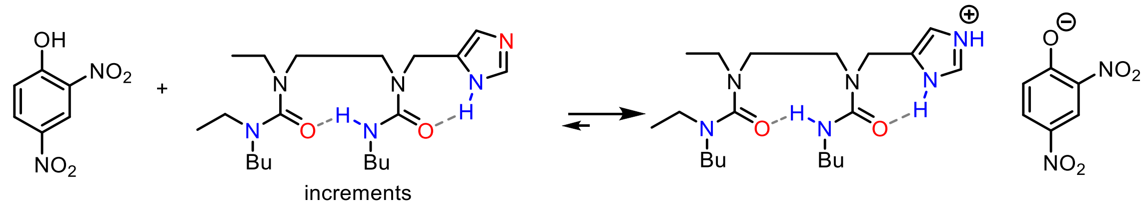

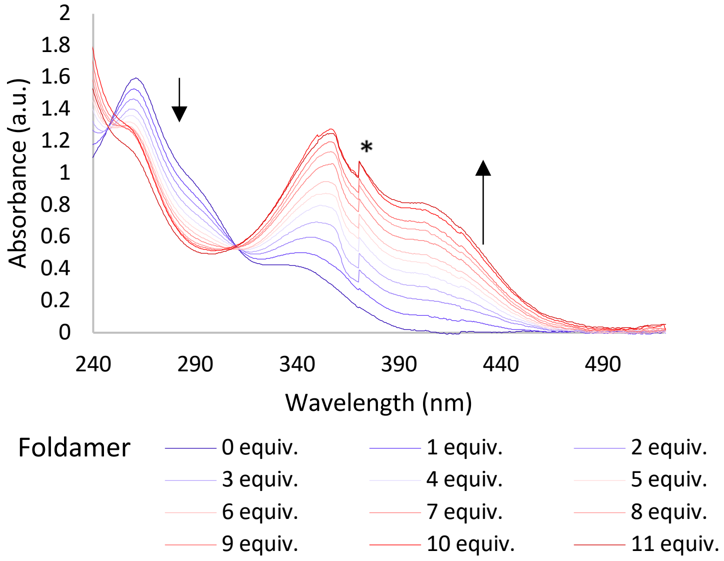

Supplementary Figure 87. Stacked UV light absorbance spectra recorded during the titration of 2,4-dinitrophenol (1.1 mM in dry dichloromethane) with compound **2**, * indicates the bulb changeover artifact between 350-375 nm. Source data are provided as a Source Data file.

Upon incremental addition of compound **2** from 0 to 11 equivalents to the 2,4-dinitrophenol solution (1.16 mM in dry dichloromethane), the colour of the solution in the cuvette visibly changes from colourless at 0 equivalent of compound **2** to increasingly yellow, a colour characteristic of 2,4-dinitrophenolates. The stacked UV light absorbance spectra recorded during the titration show the presence of two isobestic points at 247 nm and 310 nm (Supplementary Figure 87). The UV light absorbance progressively increases at wavelengths between 330 -440 nm (violet light) upon progressive addition of compound **2**. A decrease in the UV light absorbance is simultaneously observed at wavelengths between 250-300 nm during the titration. Changes in UV light absorbance essentially occur between 0 and 10 equivalents of added compound **2**: further addition of compound **2** from 10 to 11 equivalents decreases UV light absorbance at wavelengths between 330-440 nm and the absorbance curve does not participate to the isobestic points anymore, indicating a third species in equilibrium upon further addition of compound **2**. The UV light absorbance spectra of **2** alone at concentrations of 1.1 mM in dry dichloromethane (Supplementary Figure 88) shows that **2** does not feature absorbance maxima at wavelengths between 250-520 nm at the concentrations of compound **2** used during the titration. UV light absorbance spectrum of tetrabutylammonium 2,4-dinitrophenolate (1.25 mM in dry dichloromethane) show maxima of UV light absorbance at wavelengths between 350-390 nm and 400-450 nm (Supplementary Figure 83), those wavelengths for maximum UV light absorbance are identical to those observed during the titration. Overall, the titration data are consistent with the progressive deprotonation of acidic 2,4-dinitrophenol by compound **2** to form **2**:2,4-dinitrophenolate salt reaching completion of equilibrium displacement at 10 equivalents of added compound **2**. The observation is similar to the stacked UV light absorbance spectra recorded during the titration of 2,4-dinitrophenol with imidazole in otherwise similar conditions. In contrast, the stacked UV light absorbance spectra recorded during the titration of 2,4-dinitrophenol with compound **1** under otherwise similar conditions differ sharply. The difference in basicity reactivity of compound **1** and compound **2** towards 2,4-dinitrophenol correlates with the designed opposite hydrogen bond directionality of the linear chain of hydrogen bonds of compound **1** and compound **2**. Overall, the

results are consistent with a controlled basicity switch of imidazole through the switch of hydrogen bond chain directionality of the ligated foldamer.

6.3.2. Control experiment: UV absorbance of compound **2**

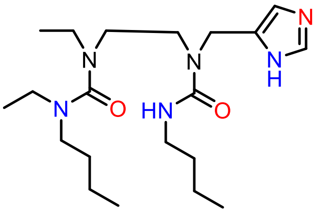

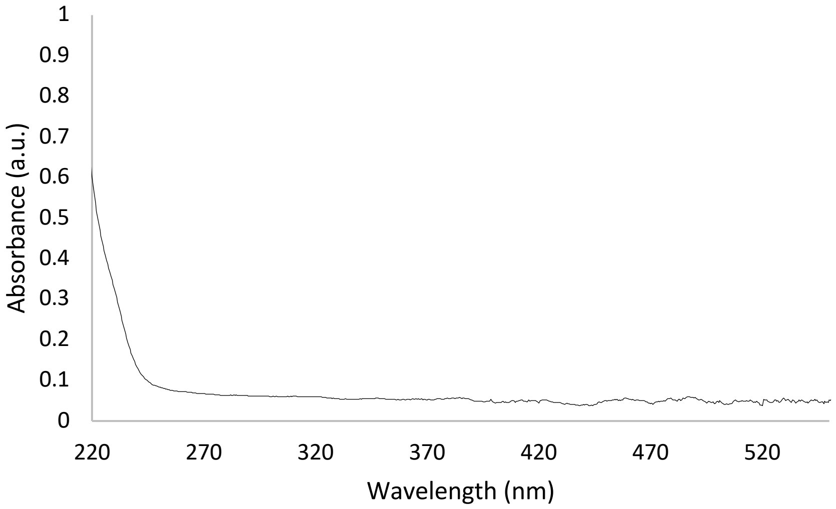

Supplementary Figure 88. UV light absorbance spectrum of foldamer **2** (1.16 mM) in dry dichloromethane**.** Source data are provided as a Source Data file.

6.4. Titration of 2,4-dinitrophenol and compound **1** with tetrabutylammonium chloride

6.4.1. UV-visible spectroscopy titration

- A stock solution of tetrabutylammonium chloride (165.5 mM in dry dichloromethane) was prepared by dissolving 23 mg (0.0827 mmol) of tetrabutylammonium chloride in 0.5 mL of dry dichloromethane.

The titrated solution was prepared by diluting 0.033 mL of 2,4-dinitrophenol stock solution (10 mM) with 0.25 mL of dry dichloromethane in the cuvette, resulting in a 1.16 mM solution of 2,4-dinitrophenol (0.00033 mmol of 2,4-dinitrophenol in the cuvette), then adding to it 0.048 mL of 81 mM stock solution of compound **1** (0.00396 mmol, 12 equivalents).

Titration with tetrabutylammonium chloride proceeds by successive addition of 0.002 mL of the tetrabutylammonium chloride stock solution (165.5 mM) each time (0.002 mL is 1 equivalent of Bu_4NCl, 0.00033 mmol) to the solution in the cuvette, agitation of the resulting solution, and recording of the UV spectrum 5 minutes after each addition. UV light absorbance intensities in titration spectra are not corrected for changes in concentration - the 2,4-dinitrophenol concentration decreased from 1.1 mM at the start to 1.0 mM at the completion of the titration. No insoluble material was formed during the titration in the cuvette.

(10) 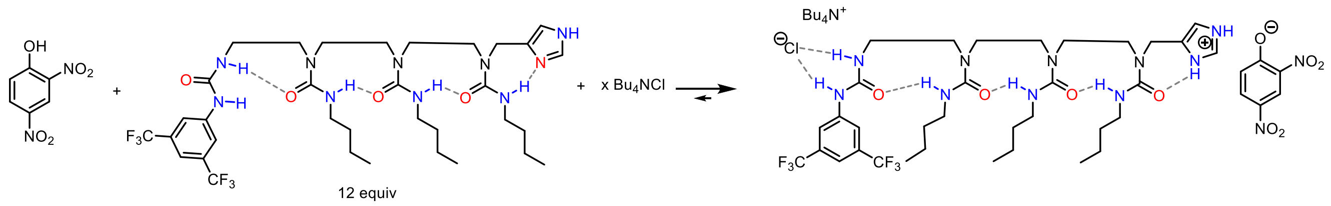

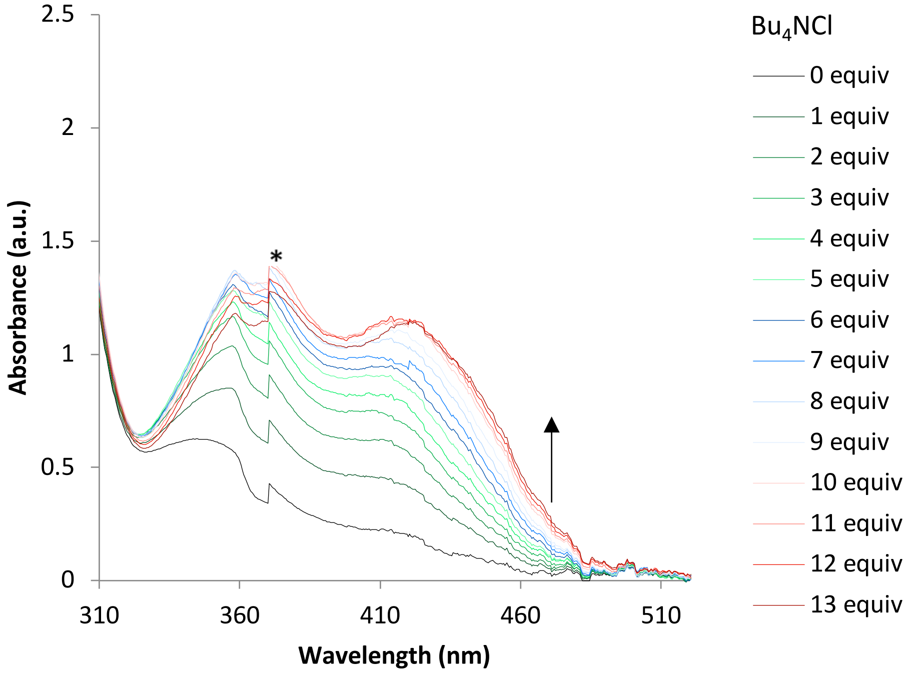

Supplementary Figure 89. Stacked UV light absorbance spectra recorded during the titration of 2,4-dinitrophenol (1.1 mM in dry dichloromethane) and **1** (12 equivalents) with increments of tetrabutylammonium chloride, ***** indicates the bulb changeover artifact between 350-375 nm. Source data are provided as a Source Data file.

Upon incremental addition of tetrabutylammonium chloride from 0 to 13 equivalents to a solution of 2,4-dinitrophenol (1.1 mM in dry dichloromethane) and compound **1** (12 equivalents), the colour of the solution visibly changed from colourless to increasingly yellow, a colour characteristic of 2,4-dinitrophenolates. The stacked UV light absorbance spectra recorded during the titration show the presence of an isobestic point at 310 nm, a progressive increase in UV light absorbance at wavelengths between 330-470 nm (violet light) occurs upon progressive addition of tetrabutylammonium chloride (Supplementary Figure 89, the UV light absorbance below 300 nm is high due to the absorbance by the 12 equivalents of foldamer). Changes in UV light absorbance at 330-470 nm occur between 0 and 10 equivalents of tetrabutylammonium chloride added, the addition of another equivalent to 11 equivalents of tetrabutylammonium chloride leads to no change in the UV light absorbance spectrum. Further addition to reach 13 equivalents decreases UV light absorbance at wavelengths between 330-440 nm with the UV light absorbance curve not participating to the isobestic point anymore, an indication of a third species in equilibrium at that stage. As control experiment, the UV light absorbance spectrum of tetrabutylammonium chloride (1 mM in dry dichloromethane) alone shows no absorbance at wavelengths between 250-520 nm (Supplementary Figure 90). Also, UV spectra of 2,4-dinitrophenol (0.25 mM in dry dichloromethane) were recorded without and with 4 equivalents of tetrabutylammonium chloride: the presence of tetrabutylamminium chloride does not alter significantly the UV light absorbance signals between 240-540 nm (Supplementary Figure 91). Control experiments indicate the UV light absorbance changes observed during the titration cannot be attributed to the direct interaction of tetrabutylammonium chloride with 2,4-dinitrophenol. The UV light absorbance spectrum of tetrabutylammonium 2,4-dinitrophenolate (1.25 mM in dry dichloromethane) show maxima of UV light absorbance at wavelengths between 350-390 nm and 400-450 nm, wavelengths of

maximum UV light absorbance that are identical to those observed during the titration with imidazole (Supplementary Figure 82). Overall, the titration data are consistent with the progressive deprotonation of acidic 2,4-dinitrophenol by {**1** : Bu_4NCl} to form 2,4-dinitrophenolate species, the full displacement of deprotonation equilibrium is reached at 10 equivalents of tetrabutylammonium chloride. The stacked spectra of this titration are very similar to those recorded during the titration of 2,4-dinitrophenol with either imidazole, or compound **2**, at similar concentration. The stacked spectra also are in sharp contrast with those recorded during the titration of 2,4-dinitrophenol with compound **1** in the absence of tetrabutylammonium chloride. The restored basicity of compound **1** in the presence of Bu_4NCl correlates with the induced switch of directionality of the hydrogen bond chain in compound **1** as shown during NMR titrations.

6.4.2. Control experiments

- UV light absorbance spectrum of tetrabutylammonium salts (1 mM in dry dichloromethane).

The 1 mM tetrabutylammonium chloride/bromide/iodide/nitrate solution was prepared by diluting 0.018 mL of tetrabutylammonium chloride 165.5 mM stock solution with 0.282 mL dry dichloromethane in the cuvette.

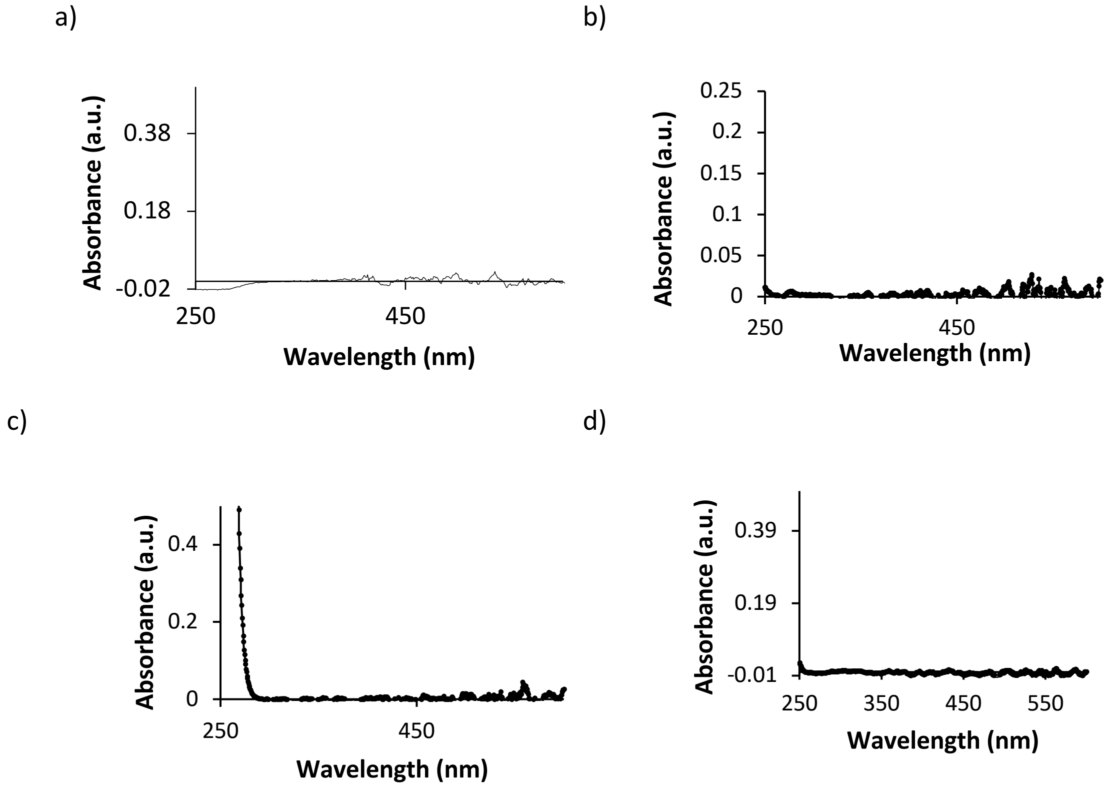

Supplementary Figure 90. UV light absorbance spectrum of a) tetrabutylammonium chloride, b) tetrabutylammonium bromide, c) tetrabutylammonium iodide, d) tetrabutylammonium nitrate (1 mM in dry dichloromethane). Source data are provided as a Source Data file.The UV light absorbance spectra show no significant absorbance at the wavelengths and concentrations used during the titrations.

- UV light absorbance spectra of 2,4-dinitrophenol (0.25 mM in dry dichloromethane) without and with 4 equivalents of tetrabutylammonium chloride

The 0.25 mM solution of 2,4-dinitrophenol in dry dichloromethane was prepared by diluting 0.0075 mL of 2,4-dinitrophenol 10 mM stock solution with 0.3 mL dichloromethane in the cuvette. After recording the UV light absorbance spectrum of it, 0.0018 mL of the tetrabutylammonium chloride 165.5 mM stock solution (4 equivalents) was added to the cuvette and the UV light absorbance spectrum of the resulting solution was recorded.

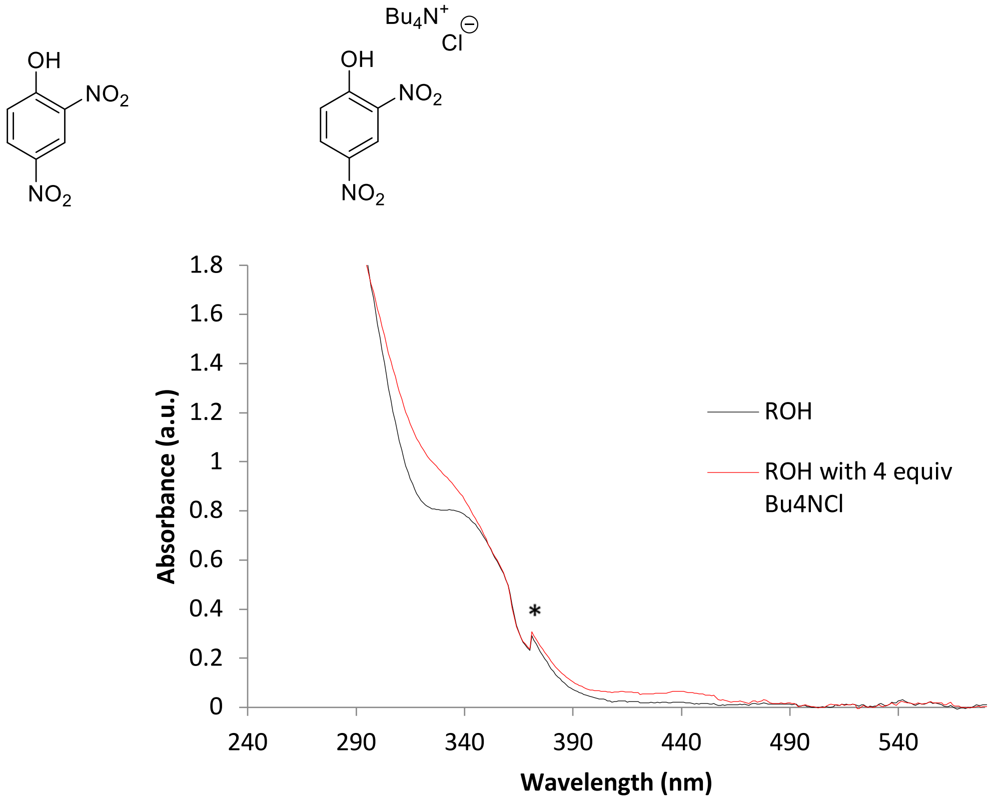

Supplementary Figure 91. Stacked UV light absorbance spectra of 2,4-dinitrophenol (0.25 mM in dry dichloromethane) without and with 4 equivalents of tetrabutylammonium chloride, * indicates the bulb changeover artifact between 350-375 nm. Source data are provided as a Source Data file.Not unexpectedly considering pKa, addition of tetrabutylammonium chloride to a solution of 2,4-dinitrophenol results in no deprotonation of the alcohol that would have generated hydrochloric acid and tetrabutylammonium 2,4-dinitrophenolate.

6.5. Time course studies of 2,4-dinitrophenol deprotonation in dichloromethane

6.5.1. Between 2,4-dinitrophenol and imidazole

A solution of 2,4-dinitrophenol (1.16 mM) was prepared by diluting 0.033 mL of 2,4-dinitrophenol stock solution (10 mM) with 0.250 mL of dry dichloromethane in the cuvette (0.00033 mmol of 2,4-dinitrophenol in the cuvette). A volume of 0.0045 mL (0.001 mmol) of the imidazole stock solution (220.6 mM) was added to the 2,4-dinitrophenol solution, followed with agitation of the resulting solution. The time course started immediately thereafter. UV light absorbance of the solution was measured automatically by the spectrometer at a wavelength of 410 nm every 3 minutes for 600 minutes. The wavelength of 410 nm chosen for the study corresponds to wavelength at which the most intense changes of UV light absorbance maxima occur during the titration of 2,4-dinitrophenol with imidazole. No insoluble material was formed during the titration in the cuvette.

(11)

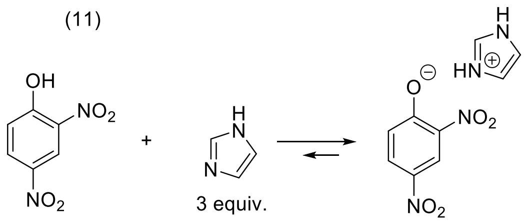

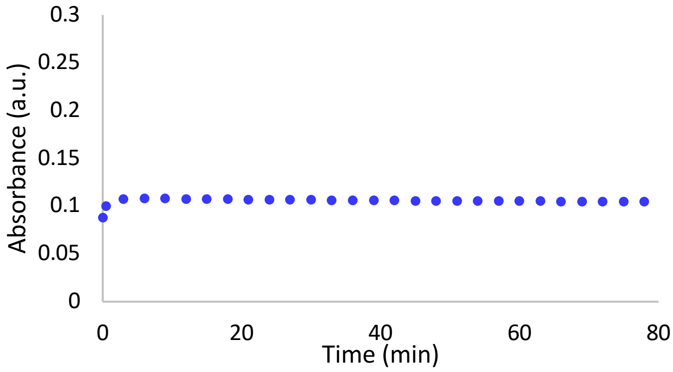

Supplementary Figure 92. Time course of the reaction between 2,4-dinitrophenol (1.16 mM in dichloromethane) and added imidazole (3 equivalents). Source data are provided as a Source Data file.The UV light absorbance at 410 nm reached a stable value (0.1 a.u.) within the 3 minutes after addition of imidazole, the absorbance value was identical 24h after the addition. This indicates the deprotonation equilibrium is reached within 3 minutes after addition of imidazole to 2,4-dinitrophenol.

6.5.2. Between 2,4-dinitrophenol and compound **1**

A solution of 2,4-dinitrophenol (1.16 mM) was prepared by diluting 0.033 mL of 2,4-dinitrophenol stock solution (10 mM) with 0.250 mL of dry dichloromethane in the cuvette (0.00033 mmol of 2,4-dinitrophenol in the cuvette). A volume of 0.012 mL (0.001 mmol) of a compound **1** stock solution (81 mM) was added to the 2,4-dinitrophenol solution in the cuvette, followed with agitation of the resulting solution. The time course started immediately thereafter. The UV light absorbance of the solution was measured automatically by the spectrometer at a wavelength of 410 nm, the measurement was recorded every 3 minutes for 600 minutes. The wavelength of 410 nm chosen for the study corresponds to wavelength at which the most intense changes of UV light absorbance maxima occur during the titration of 2,4-dinitrophenol with imidazole. No insoluble material was formed during the titration in the cuvette.

(12)

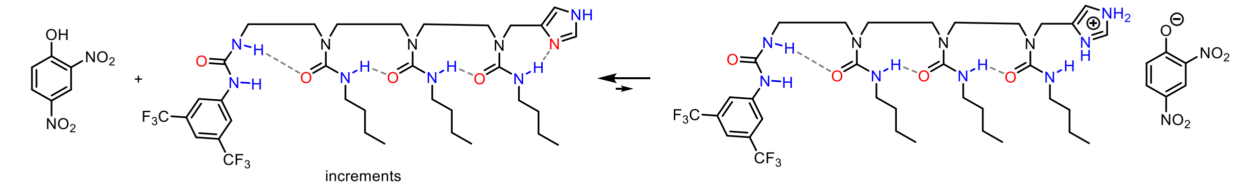

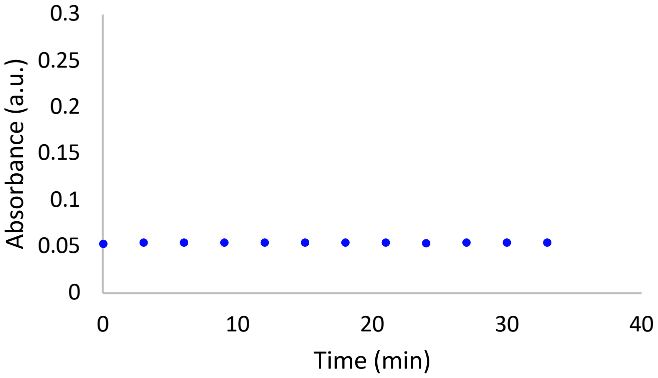

Supplementary Figure 93. Time course of the reaction between 2,4-dinitrophenol (1.16 mM) and compound **1** (3 equivalents). Source data are provided as a Source Data file.The UV light absorbance at 410 nm is stable (0.05 a.u.) within the 3 minutes after addition, the UV light absorbance value was identical 14 h after the addition. This indicates the deprotonation equilibrium is reached within 3 minutes after addition. The UV absorbance was half of the value obtained with imidazole under identical concentrations.

6.5.3. Between 2,4-dinitrophenol and compound **1** in the presence of tetrabutylammonium chloride

A solution of 2,4-dinitrophenol (1.16 mM) prepared by diluting 0.033 mL of 2,4-dinitrophenol stock solution (10 mM) with 0.250 mL of dry dichloromethane in the cuvette (0.00033 mmol of 2,4-dinitrophenol in the cuvette). A volume of 0.012 mL (0.001 mmol) of a compound **1** stock solution (81 mM) was added to the 2,4-dinitrophenol solution in the cuvette, followed with agitation of the resulting solution. A volume of 0.006 mL (0.001 mmol) of a stock solution of tetrabutylammonium chloride (165.5 mM) was added to the solution in the cuvette, followed with agitation of the solution. The Time course started immediately thereafter. UV light absorbance of the solution was measured automatically by the spectrometer at a wavelength of 410 nm, the measurement was made every 3 minutes for one hour. The wavelength of 410 nm chosen for the study corresponds to wavelength at which the most intense changes of UV light absorbance maxima occur during the titration of 2,4-dinitrophenol with imidazole. No insoluble material was formed during the titration in the cuvette.

(13)

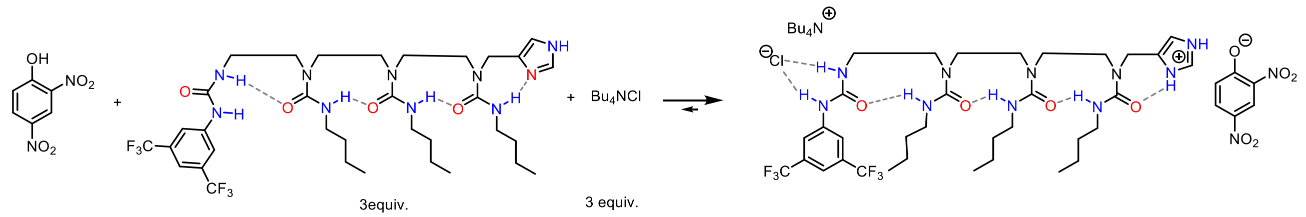

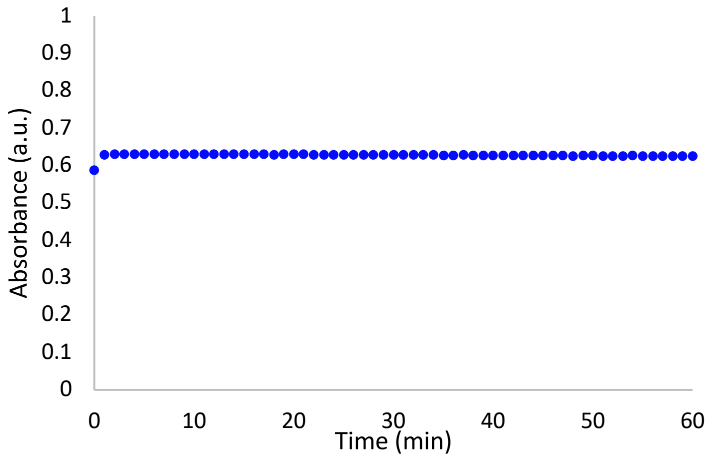

Supplementary Figure 94. Time course of the reaction between 2,4-dinitrophenol (1.16 mM) and 1 (3 equivalents) with tetrabutylammonium chloride (3 equivalents). Source data are provided as a Source Data file.One minute after the addition of tetrabutylammonium chloride to the solution in the cuvette, the UV light absorbance value had reached a stable value (0.63 a.u.) that did not change after 1 day. This indicates the deprotonation equilibrium is reached within 3 minutes after addition.

7. Nucleophilic additions to bis(4-nitrophenyl)carbonate

7.1. Reaction of bis(4-nitrophenyl)carbonate with imidazole in dry dichloromethane

7.1.1. Reaction of bis(4-nitrophenyl)carbonate with imidazole, monitored by UV visible spectrometry

- A stock solution of bis(4-nitrophenyl)carbonate **4** 32.9 mM in dry dichloromethane was prepared by dissolving bis(4-nitrophenyl)carbonate **4** (10 mg, 0.0329 mmol) in 1 mL dry dichloromethane.

The titrated solution of bis(4-nitrophenyl)carbonate **4** (0.37 mM) in dry dichloromethane was prepared by diluting 0.0033 mL of bis(4-nitrophenyl)carbonate stock solution (32.9 mM) with 0.297 mL of dry dichloromethane in the cuvette (0.00011 mmol of bis(4-nitrophenyl)carbonate in the cuvette).

Titration with imidazole proceeds by successive addition of 0.0005 mL of the imidazole stock solution (220.6 mM) each time (0.0005 mL is 1 equivalent of imidazole**,** 0.00011 mmol) to the bis(4-nitrophenyl)carbonate solution in the cuvette, agitation of the resulting solution, and recording of the UV light absorbance spectrum 5 minutes after each addition. UV light absorbance intensities shown in UV titration spectra are not corrected for changes in concentration - the substrate concentrations decreased from 0.37 mM at the start to 0.36 mM at the completion of the titration. No insoluble material was formed during the titration in the cuvette.

(14)

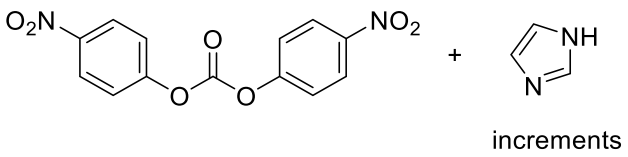

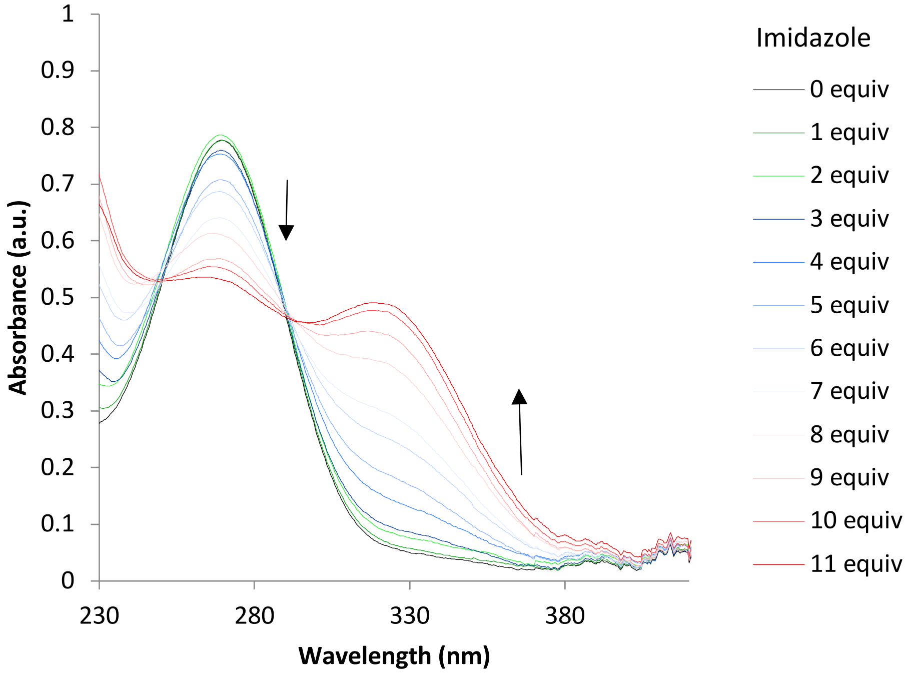

Supplementary Figure 95. Stacked UV light absorbance spectra recorded during the titration of bis(4-nitrophenyl)carbonate **4** (0.37 mM in dry dichloromethane) with increments of imidazole. Source data are provided as a Source Data file.

The stacked UV light absorbance spectra recorded during the titration show 2 isobestic points at 252 nm and 293 nm, a progressive increase in UV light absorbance at wavelengths between 300-370 nm, and a progressive decrease in UV light absorbance at wavelengths between 250-280 nm upon addition of up to 11 equivalents of imidazole. Addition of one more equivalent of imidazole to 12 equivalent decreases absorbance at all UV light

wavelengths and the absorbance curve does not participate to the isobestic points anymore, indicating a third species in equilibrium upon further addition of imidazole. The UV light absorbance spectra of imidazole alone at concentrations of 1 mM and 10 mM in dry dichloromethane (Supplementary Figure 90) show that imidazole does not lead to any absorbance at wavelengths between 250-520 nm at the concentrations of imidazole used during the titration. UV light absorbance spectrum of tetrabutylammonium 4-nitrophenolate (0.37 mM in dry dichloromethane) show maxima of UV light absorbance at wavelengths between 380-460 nm (Supplementary Figure 96) that is not present during the titration of bis(4-nitrophenyl)carbonate, indicating this species does not form. A control experiment established that 4-nitrophenol is not acidic enough to be deprotonated significantly by imidazole under the titration conditions (by UV spectrometry: Supplementary figures 97-99, by NMR spectrometry: Supplementary Figures 100-101). Overall, the titration data are consistent with the progressive nucleophilic addition of imidazole to bis(4-nitrophenyl)carbonate to form the nucleophilic adduct and 4-nitrophenol. The reaction was also monitored by NMR spectroscopy and mass spectrometry to characterise the nucleophilic adduct and 4-nitrophenol products formed (see part 7.1.3).

7.1.2. UV visible spectroscopy control experiments

- UV light absorbance spectrum of O_2NPhO^-, Bu_4N^+ (0.37 mM in dry dichloromethane).

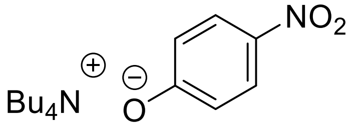

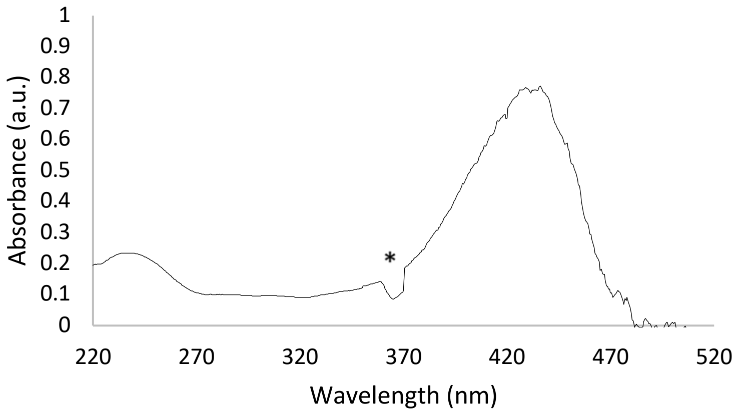

Supplementary Figure 96. UV light absorbance spectrum of O_2NPhO^-, Bu_4N^+ (0.37 mM in dry dichloromethane). The spectrum features UV light absorbance maxima at wavelengths between 380-460 nm, * indicates the bulb changeover artifact between 350-375 nm. Source data are provided as a Source Data file.

- UV light absorbance spectrum of O_2NPhOH (0.37 mM in dry dichloromethane).

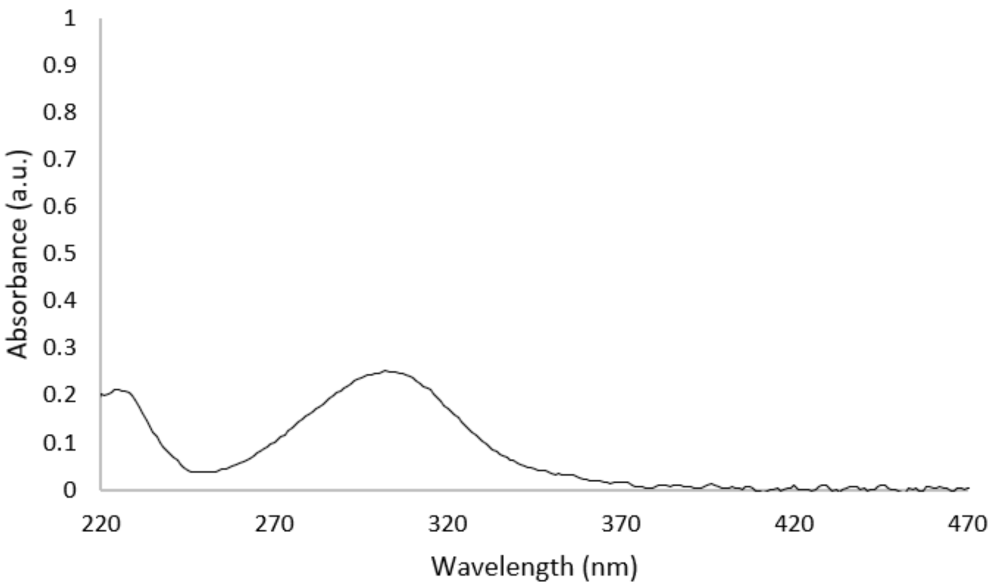

Supplementary Figure 97. UV light absorbance spectrum of 4-nitrophenol (0.37 mM in dry dichloromethane). Source data are provided as a Source Data file.

- UV light absorbance spectrum of 4-nitrophenol (0.37 mM in dry dichloromethane) in the presence of imidazole.

(15) 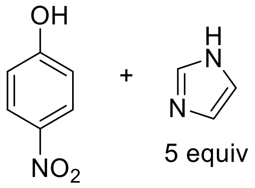

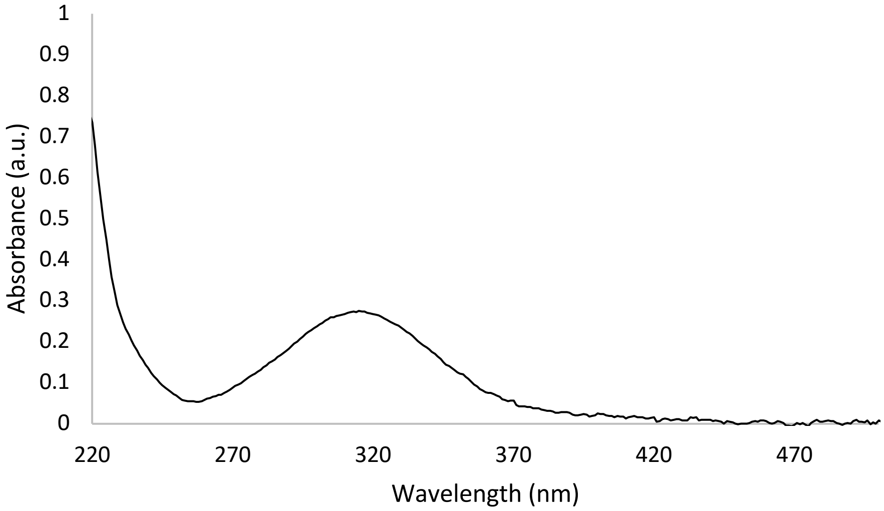

Supplementary Figure 98. UV light absorbance spectrum of 4-nitrophenol (0.37 mM in dry dichloromethane) in the presence of 5 equivalents of imidazole. Source data are provided as a Source Data file.

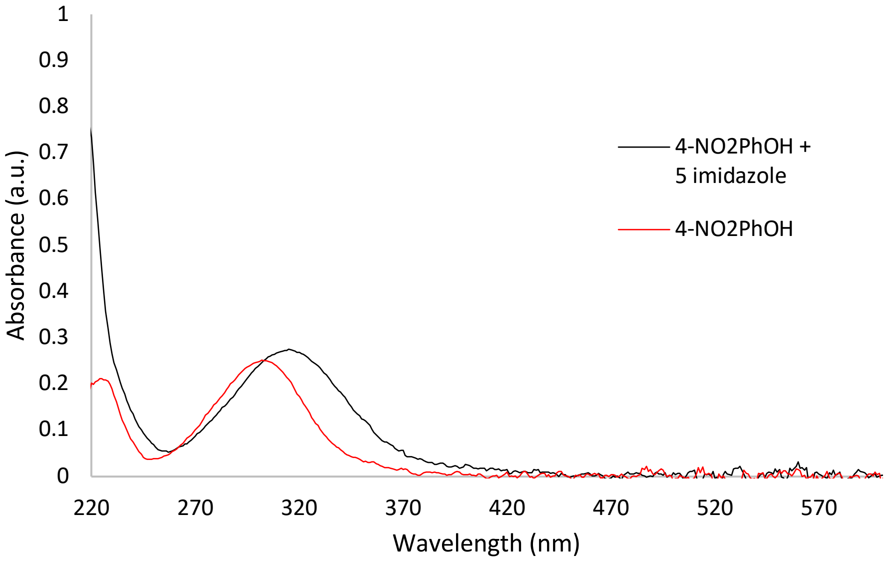

Supplementary Figure 99. Overlay of UV light absorbance spectra of 4-nitrophenol (0.37 mM in dry dichloromethane) without and in the presence of 5 equivalents of imidazole. Source data are provided as a Source Data file.The small shift in maximum absorbance is attributed to minor deprotonation of 4-nitrophenol as compared to the spectrum of tetrabutylammonium 4-nitrophenolate and correlated with ^{1}H NMR and ^{13}C NMR spectra of 4-nitrophenol without and in the presence of imidazole.

7.1.3. Reaction of bis(4-nitrophenyl)carbonate with imidazole monitored by NMR spectroscopy and mass spectrometry

The reaction of bis(4-nitrophenyl)carbonate **4** (10 mg, 0.0328 mmol) with imidazole (1 and 3 equivalents) in dry deuterated dichloromethane (0.5 mL) at ambient temperature was monitored by ^{1}H, COSY, ^{13}C NMR spectroscopy. Using one equivalent of imidazole (2.2 mg, 0.0328 mmol) after 13 hours of reaction, ^{1}H and ^{13}C NMR analyses showed that 4-nitrophenol and 4-nitrophenyl 1H-imidazole-1-carboxylate were formed as sole products of the reaction (Supplementary Figures 103-108), the observation was confirmed by measuring HRMS of 4-nitrophenyl 1H-imidazole-1-carboxylate (Supplementary Figure 109). Using three equivalents of imidazole (6.6 mg, 0.0984 mmol) under otherwise similar reaction conditions, NMR and mass analyses showed that 4-nitrophenol and 4-nitrophenyl 1H-imidazole-1-carboxylate were also the sole products of the reaction (the excess of imidazole did not react further with 4-nitrophenyl 1H-imidazole-1-carboxylate).

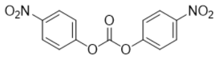

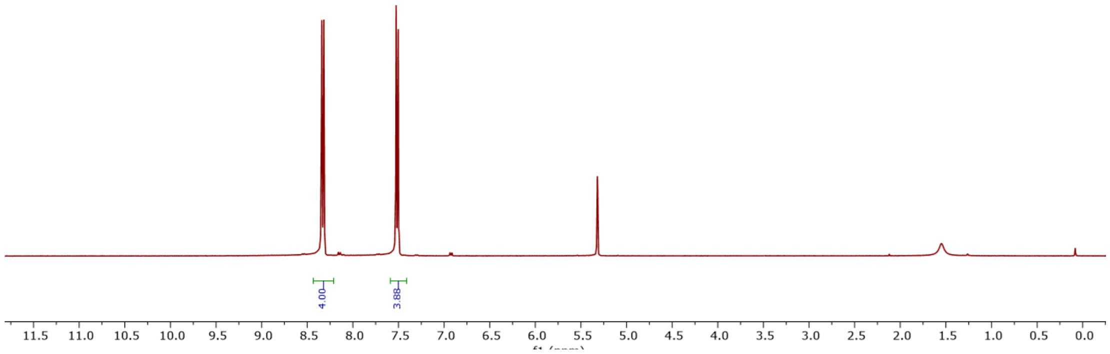

Supplementary Figure 100. ^{1}H NMR spectrum of bis(4-nitrophenyl)carbonate **4**. **^{1}H NMR** (400 MHz, CD_2Cl_2) δ 8.43 – 8.21 (m, 4H, 4 × CH_{Ph}), 7.59 – 7.41 (m, 4H, 4 × CH_{Ph}).

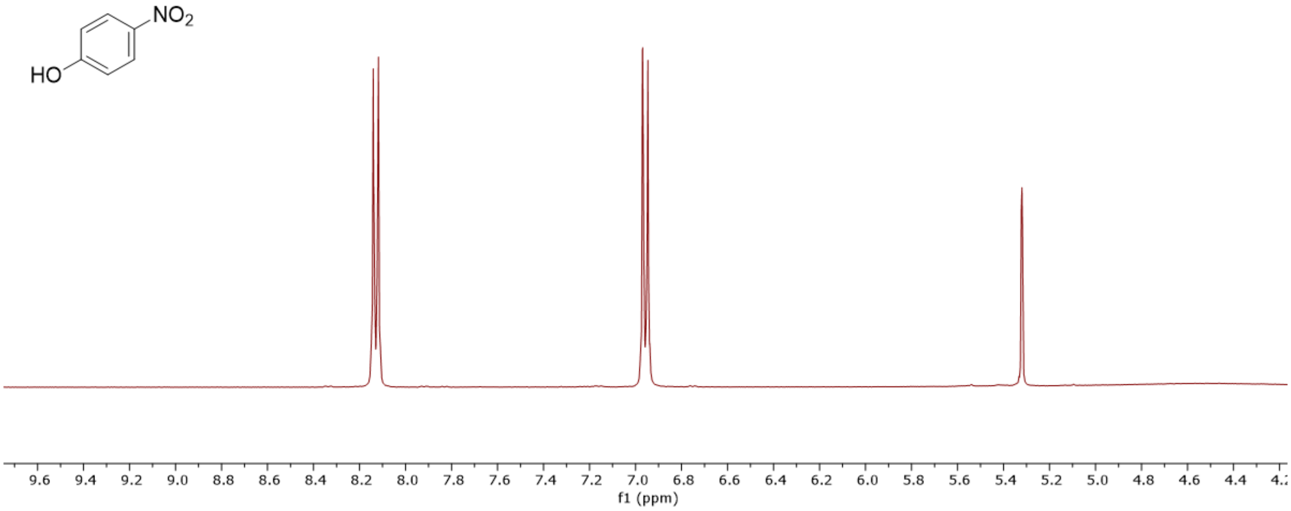

Supplementary Figure 101. ^{1}H NMR spectrum of 4-nitrophenol. **^{1}H NMR** (400 MHz, CD_2Cl_2) δ 8.13 (d, *J* = 9.2 Hz, 2H, 2 × CH_{Ph}), 6.96 (d, *J* = 9.2 Hz, 2H, 2 × CH_{Ph}).

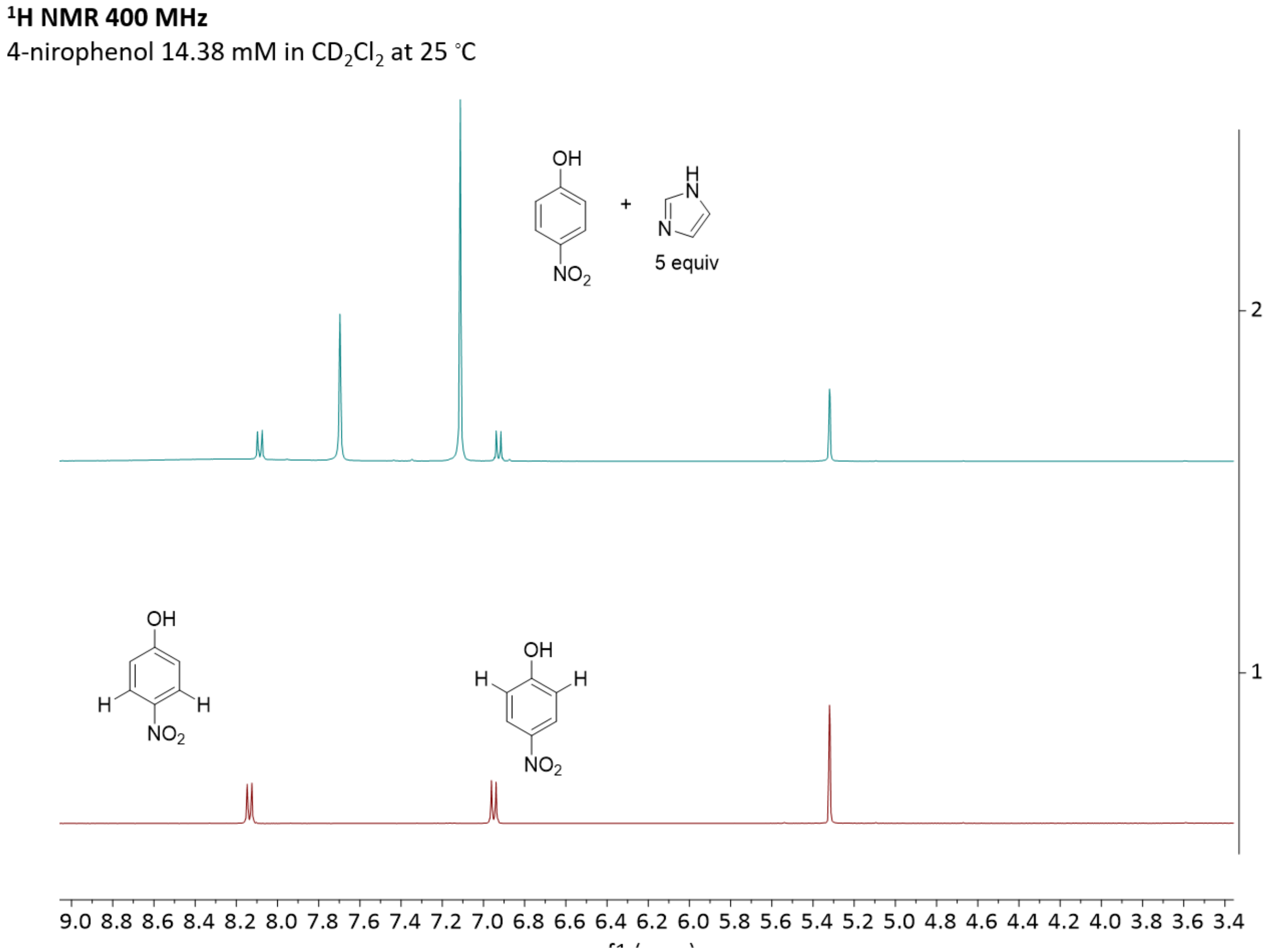

Supplementary Figure 102. Overlay of ^{1}H NMR spectra of 4-nitrophenol (14.38 mM in dichloromethane at 25 °C) without and in the presence of 5 equivalents of imidazole. Addition of imidazole to 4-nitrophenol in dichloromethane does not cause its deprotonation.

(16)

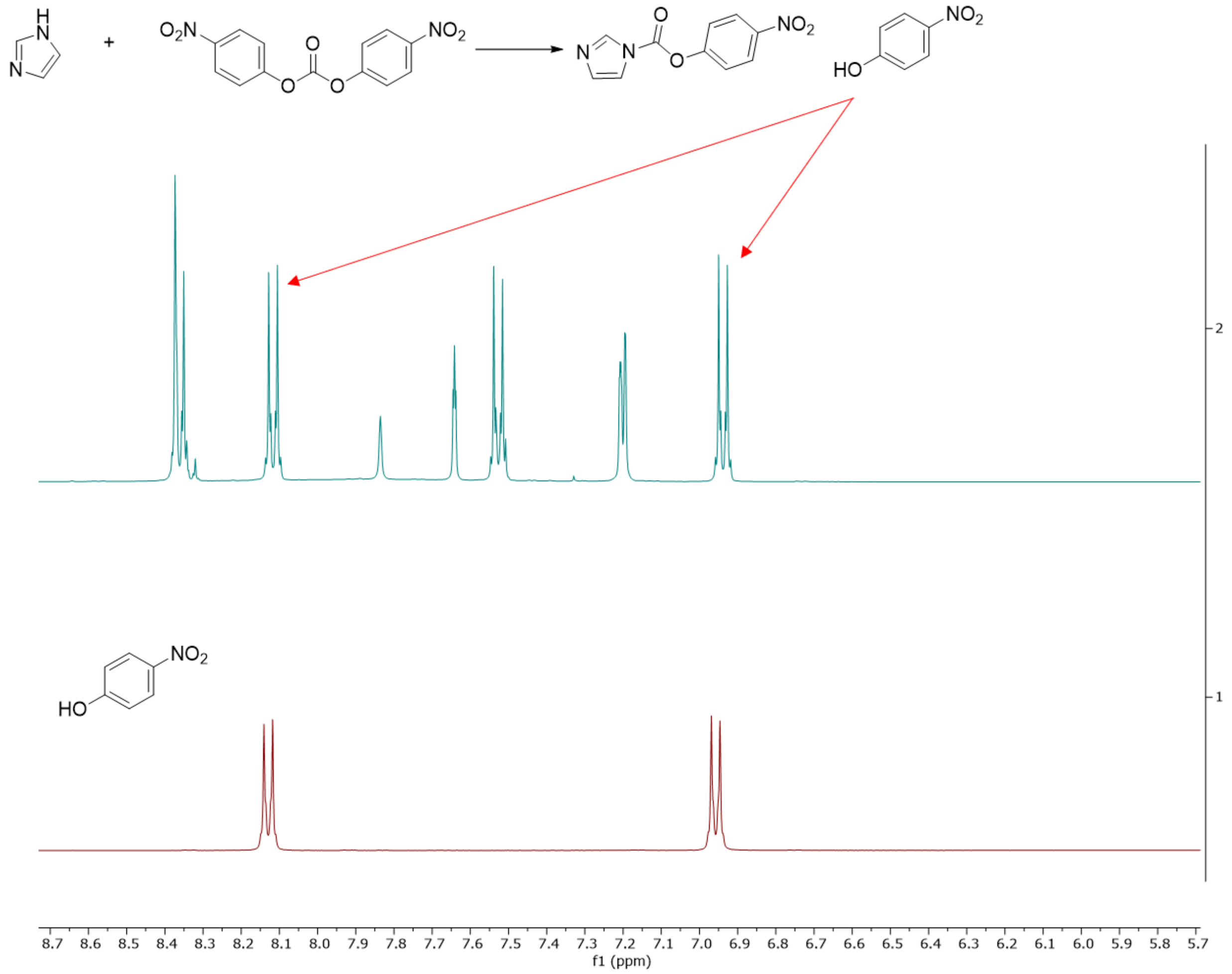

Supplementary Figure 103. Overlay of ^{1}H NMR spectra of 4-nitrophenol and of a mixture imidazole (65.6 mM in dichloromethane)+ bis(4-nitrophenyl)carbonate **4** (1 equivalent) after 13 hours at ambient temperature.

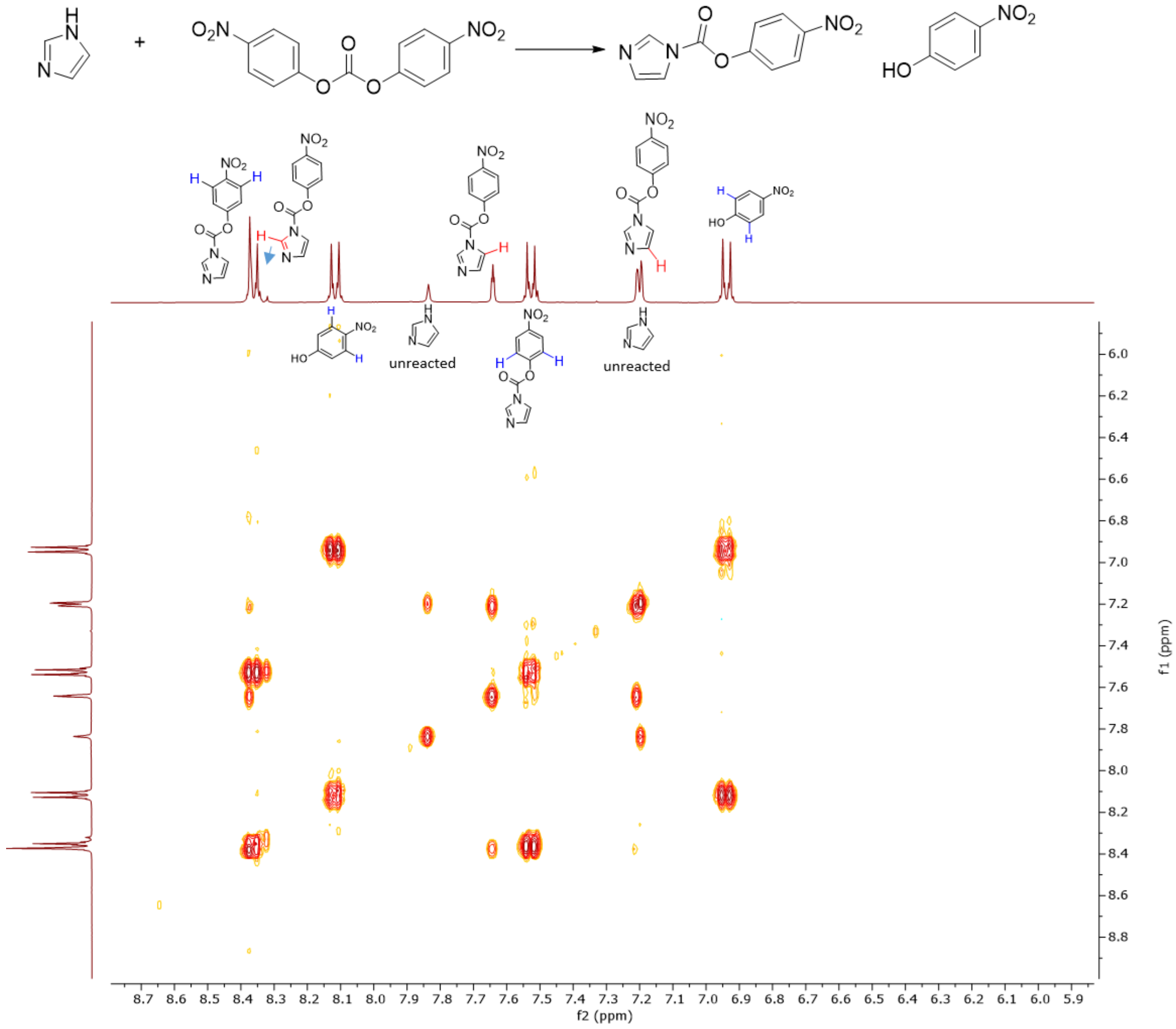

Supplementary Figure 104. COSY NMR spectrum of a mixture imidazole (65.6 mM in dichloromethane) + bis(4-nitrophenyl)carbonate **4** (1 equivalent) after 13 hours at ambient temperature.

3 equivalents of imidazole, 13 hours

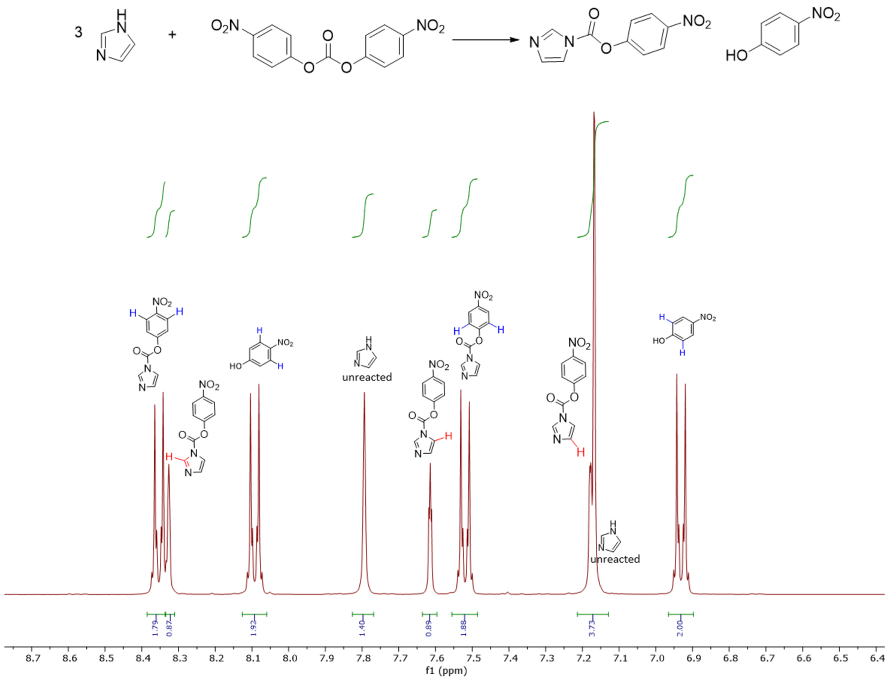

Supplementary Figure 105. ^{1}H NMR spectrum of a mixture 3 equivalents of imidazole+ bis(4-nitrophenyl)carbonate **4** (65.6 mM in dichloromethane) after 13 hours at ambient temperature.

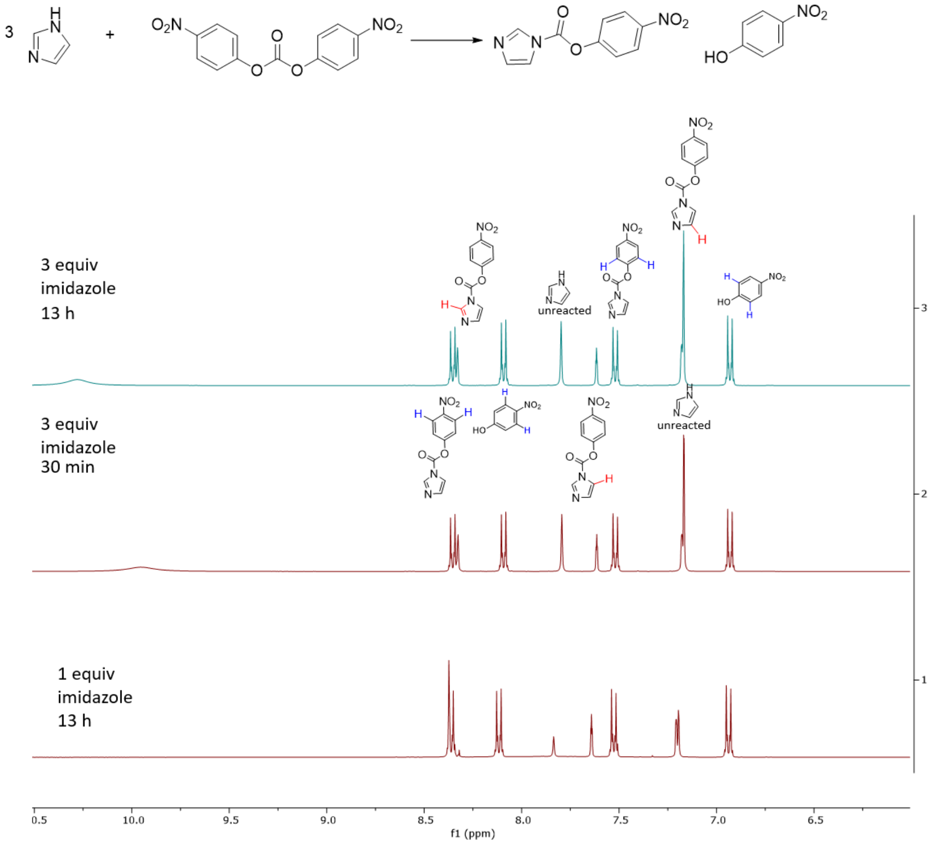

Supplementary Figure 106. Overlay of ^{1}H NMR spectra of a mixture imidazole (1 or 3 equivalents) + bis(4-nitrophenyl)carbonate **4** (65.6 mM in dichloromethane) after 13 hours at ambient temperature.

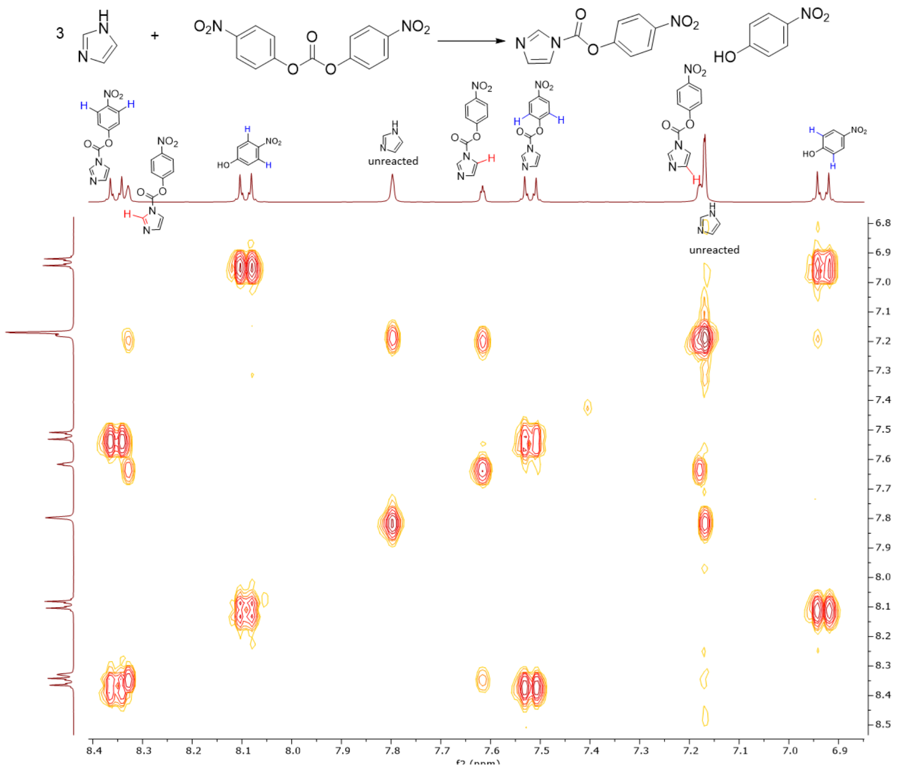

Supplementary Figure 107. COSY ^{1}H NMR spectrum of a mixture imidazole (3 equivalents) + bis(4-nitrophenyl)carbonate **4** (65.6 mM in dichloromethane) after 13 hours at ambient temperature.

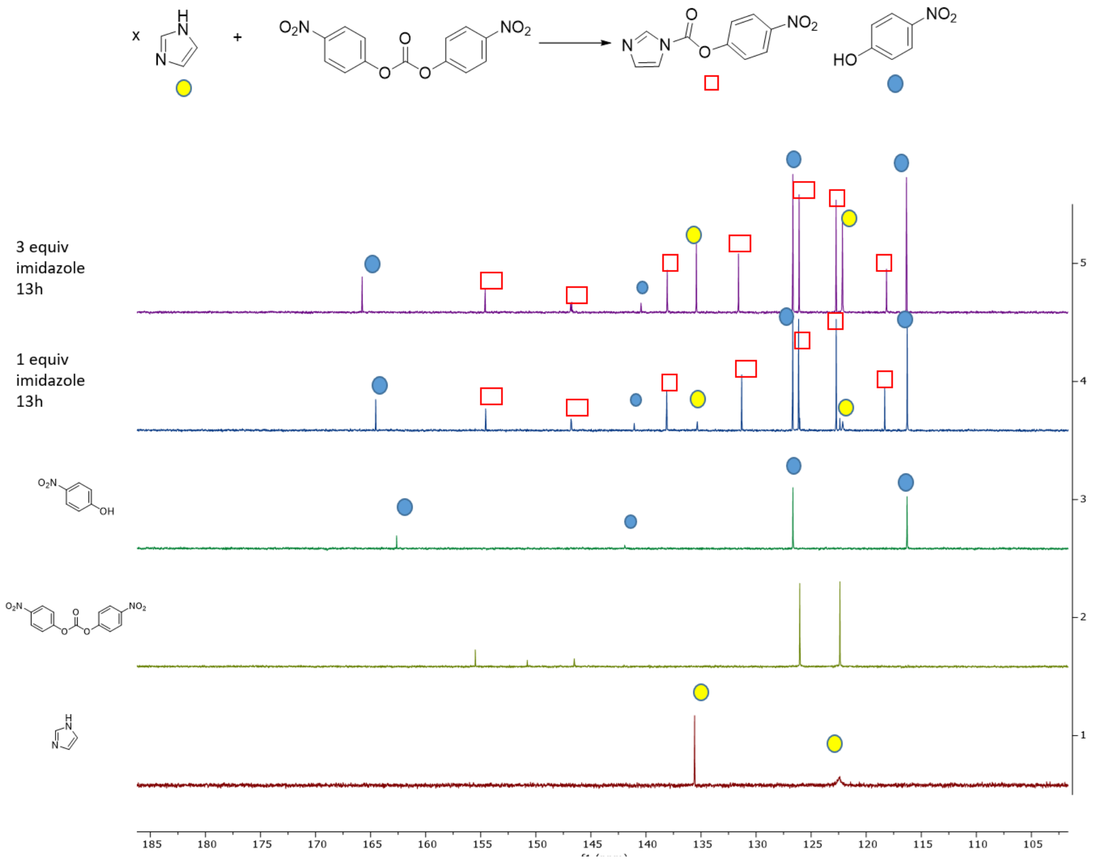

Supplementary Figure 108. Overlay of ^{13}C NMR spectra of imidazole, **4**, 4-nitrophenol, imidazole (1 equivalent) + **4** (65.6 mM in dichloromethane) after 13 hours at ambient temperature, imidazole (3 equivalent) + **4** (65.6 mM in dichloromethane) after 13 hours at ambient temperature.

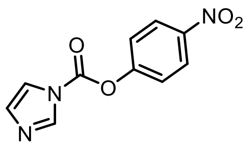

Thermo Q Exactive ASAP pos

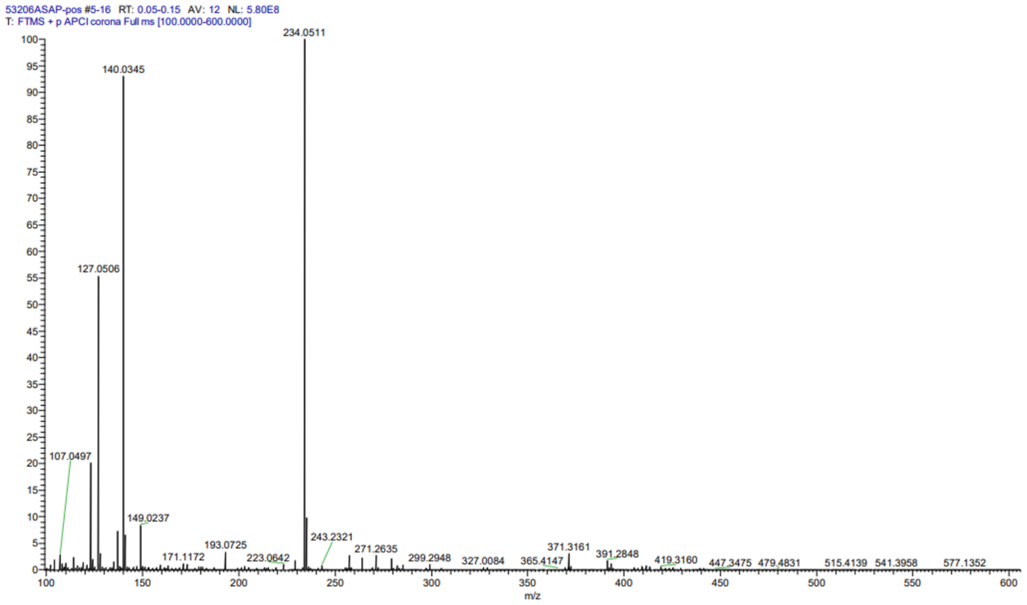

Thermo Q Exactive ASAP pos

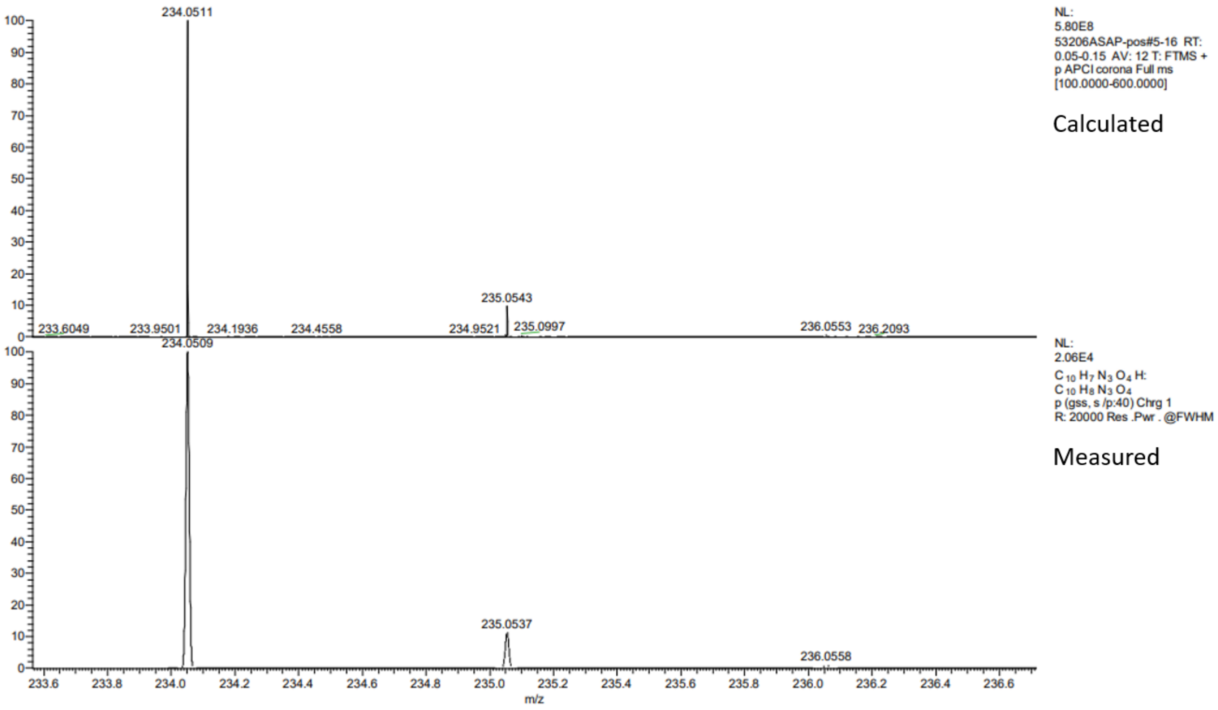

Supplementary Figure 109. HRMS spectra of adduct formed by reaction of imidazole (3 equivalent) with bis(4-nitrophenyl)carbonate **4** (1 equivalent) in dichloromethane after 13 hours at ambient temperature (calculated spectrum and measured spectrum illustrating the match of data). **HR – MS** (ESI, positive ion mode) – *m/z* for $[C_{10}H_8N_3O_4]^+$ calculated 234.0511, observed 234.0509.

7.2. Reaction of bis(4-nitrophenyl)carbonate with compound **1** monitored by UV visible spectroscopy

- A stock solution of **1** (34.8 mM in dry dichloromethane) was prepared by diluting 27.1 mg (0.0348 mmol) of **1** in 1 mL of dry dichloromethane.

The titrated solution of bis(4-nitrophenyl)carbonate 4 (0.37 mM) in dry dichloromethane was prepared by diluting 0.0033 mL of bis(4-nitrophenyl)carbonate stock solution (32.9 mM) with 0.297 mL of dry dichloromethane in the cuvette (0.00011 mmol of bis(4-nitrophenyl)carbonate in the cuvette).

Titration with compound **1** proceeds by successive addition of 0.0031 mL of the **1** stock solution (34.8 mM) each time (0.0031 mL is 1 equivalent of compound **1**, 0,00011 mmol) to the 2 bis(4-nitrophenyl)carbonate solution in the cuvette (0.37 mM), agitation of the resulting solution, and recording of the UV spectrum 5 minutes after each addition. UV light absorbance intensities shown in UV titration spectra are not corrected for changes in concentration - the substrate concentrations decreased from 0.37 mM at the start to 0.33 mM at the completion of the titration. No insoluble material was formed during the titration in the cuvette.

(17)

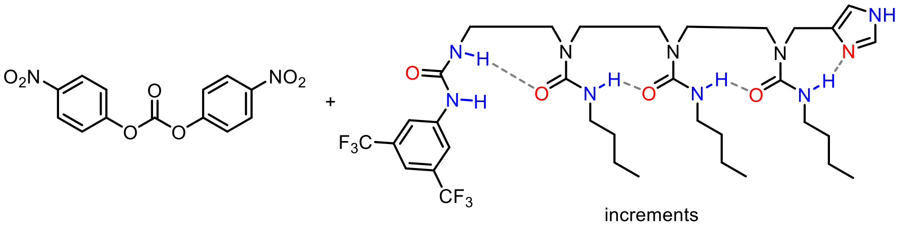

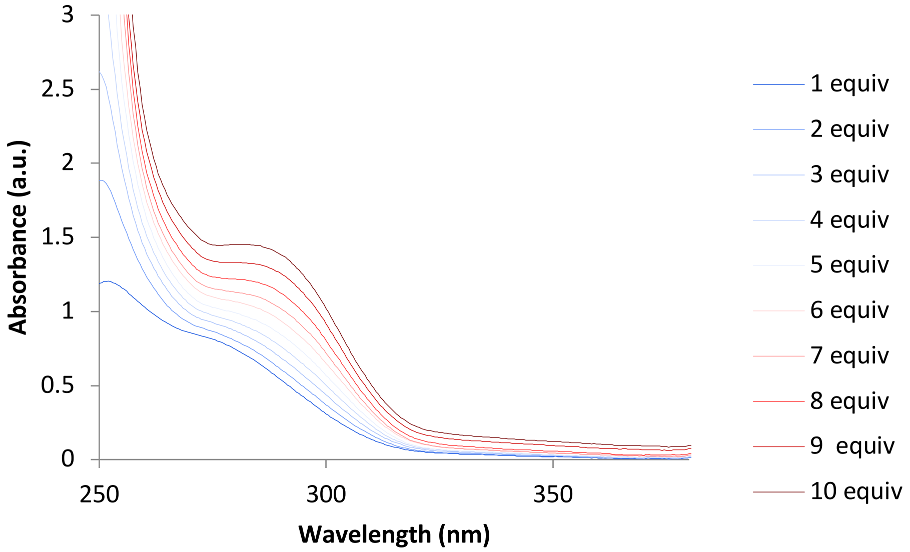

Supplementary Figure 110. Stacked UV light absorbance spectra recorded during the titration of bis(4-nitrophenyl)carbonate 4 (0.37 mM in dry dichloromethane) with increments of compound **1.** Source data are provided as a Source Data file.

Upon incremental addition of compound **1** from 0 to 10 equivalents to the bis(4-nitrophenyl)carbonate (0.37 mM in dry dichloromethane), the stacked UV light absorbance spectra show continuous uniform progressive small increases in absorbance at all wavelengths with no isobestic point between 220 nm and 600 nm. Strong UV light absorbance values (above 2 a.u.) are recorded below 300 nm. The UV light absorbance spectrum of a solution of compound **1** alone (1.16 mM in dry dichloromethane, Supplementary Figure 86) also shows strong absorbance at wavelengths below 300 nm, the strong values of absorbance recorded below 300 nm are attributed to the absorbance of the foldamer (10 equivalents of foldamer = 3.7 mM). The uniform progressive small increases in absorbance recorded during the titration are attributed only to the absorbance of increasing amount of compound

1 added during the titration (see UV light absorbance of compound **1** in dry dichloromethane). The stacked spectra contrast sharply with those obtained during the titration of bis(4-nitrophenyl)carbonate (0.37 mM in dry dichloromethane) with increments of imidazole in otherwise identical conditions (Supplementary Figure 95), indicating a difference of reactivity of imidazole when ligated to the foldamer in compound **1**. Overall, the data indicate compound **1** does not react significantly with bis(4-nitrophenyl)carbonate **4** under the experimental conditions.

7.3. Reaction of bis(4-nitrophenyl)carbonate with compound 2

7.3.1. Reaction of bis(4-nitrophenyl)carbonate with 2 monitored by UV-visible spectroscopy

- A stock solution of **2** (144 mM in dry dichloromethane) was prepared by diluting 28.4 mg (0.072 mmol) of compound **2** in 0.5 mL of dry dichloromethane.

The titrated solution of bis(4-nitrophenyl)carbonate **4** (0.37 mM) in dry dichloromethane was prepared by diluting 0.0036 mL of bis(4-nitrophenyl)carbonate stock solution (32.9 mM) with 0.297 mL of dry dichloromethane in the cuvette (0.00011 mmol of bis(4-nitrophenyl)carbonate in the cuvette).

Titration with compound **2** proceeds by successive addition of 0.00077 mL of the compound **2** stock solution (144 mM) each time (0.00077 mL is 1 equivalent of compound **2**, 0,00011 mmol) to the 2 bis(4-nitrophenyl)carbonate solution in the cuvette (0.37 mM), agitation of the resulting solution, and recording of the UV spectrum 5 minutes after each addition. UV light absorbance intensities shown in UV titration spectra are not corrected for changes in concentration - the substrate concentrations decreased from 0.37 mM at the start to 0.33 mM at the end of the titration. No insoluble material was formed during the titration in the cuvette.

(18)

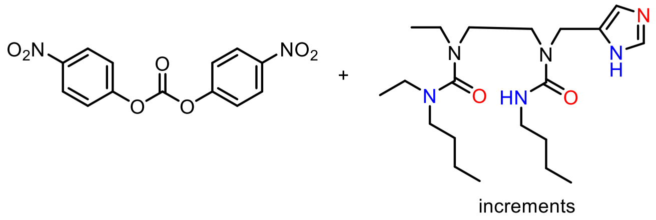

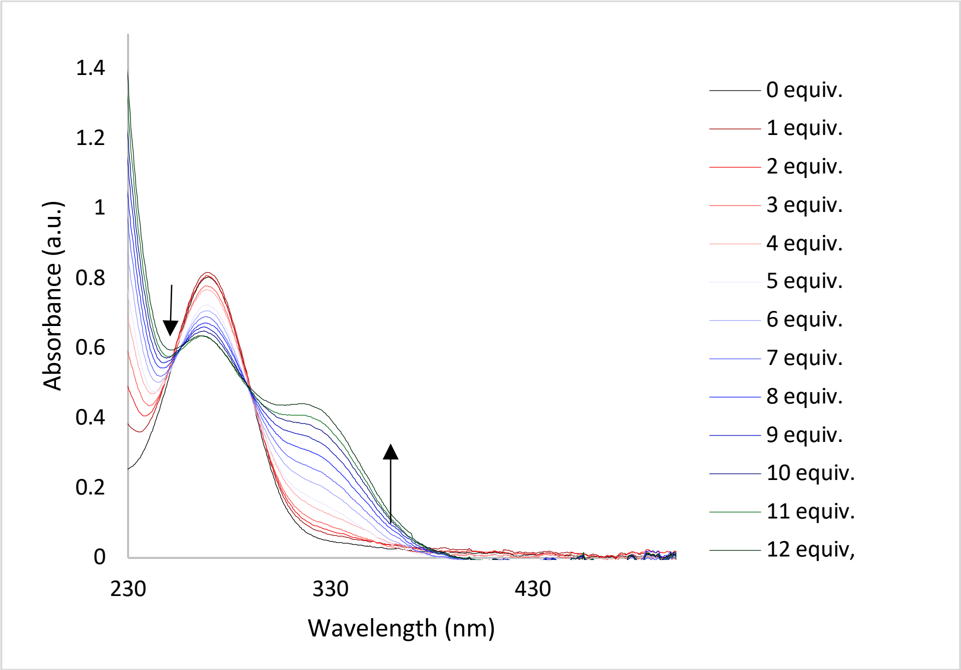

Supplementary Figure 111. Stacked UV light absorbance spectra recorded during the titration of bis(4-nitrophenyl)carbonate 4 (0.37 mM in dry dichloromethane) with increments of compound **2**. Source data are provided as a Source Data file.

Upon incremental addition of compound **2** from 0 to 12 equivalents to the bis(4-nitrophenyl)carbonate (0.37 mM in dry dichloromethane), the stacked UV light absorbance spectra show 2 isobestic points at 252 nm and 293 nm, a progressive increase in UV light absorbance at wavelengths between 300-370 nm, and a simultaneous progressive decrease in UV light absorbance at wavelengths between 250-280 nm. Addition of more than 12 equivalents of compound **2** leads to a UV light absorbance curve that does not participate anymore to the isobestic points, indicating a third species in equilibrium upon further addition of compound **2**. The UV light absorbance spectrum of compound **2** alone (1.16 mM in dry dichloromethane, control experiment, Supplementary Figure 88) shows no UV light absorbance maxima between 250 nm and 700 nm, thus the variations of UV light absorbance upon addition of increments of compound **2** cannot be attributed to it. The stacked spectra recorded during this titration are very similar to those obtained during the titration of bis(4-nitrophenyl)carbonate (0.37 mM in dry dichloromethane) with increments of imidazole (Supplementary Figure 95). Also, the stacked spectra are sharply contrasting with those obtained during the titration of bis(4-nitrophenyl)carbonate (0.37 mM in dry dichloromethane) with increments of compound **1** (Supplementary Figure 110). Overall, the titration data are consistent with the formation of the nucleophilic addition adduct. **2** of blocked hydrogen bond directionality has similar reactivity than imidazole towards the nucleophilic addition to bis(4-nitrophenyl)carbonate, its reactivity strongly differs from compound **1** that features an hydrogen bond chain of opposite hydrogen bond directionality.

7.3.2. Reaction of bis(4-nitrophenyl)carbonate with compound 2 monitored by NMR spectroscopy and mass spectrometry

The reaction of compound **2** (6.5 mg, 0.01649 mmol, 41.22 mM) with bis(4-nitrophenyl)carbonate **4** (10 mg, 0.032 mmol, 2 equivalent) in dry deuterated dichloromethane (0.4 mL) at ambient temperature was monitored by ^{1}H, COSY, ^{13}C NMR spectroscopy. After 13 hours of reaction, ^{1}H and ^{13}C NMR analyses showed that 4-nitrophenol and 4-nitrophenyl 1H-imidazole-1-carboxylate derivative of compound **2** were formed as sole products of the reaction (Supplementary Figures 112-115), the observation was confirmed by measuring HRMS of the **2** 4-nitrophenyl 1H-imidazole-1-carboxylate adduct (Supplementary Figure 116).

(19)

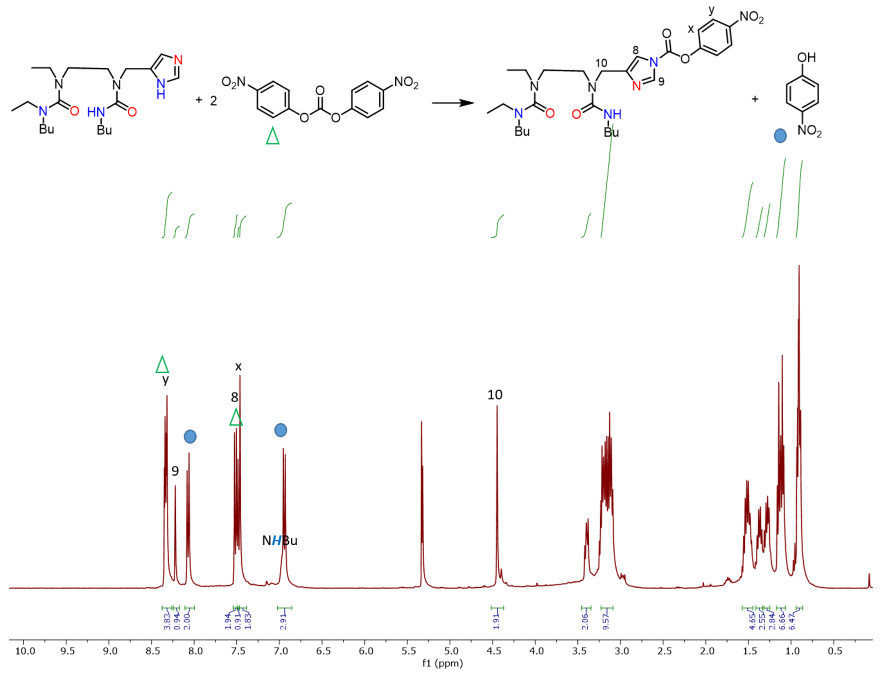

Supplementary Figure 112. ^{1}H NMR spectrum of a mixture of compound **2** (41.22 mM in CD_2Cl_2) + 2 equivalents of bis(4-nitrophenyl)carbonate **4** after 13 hours at ambient temperature.

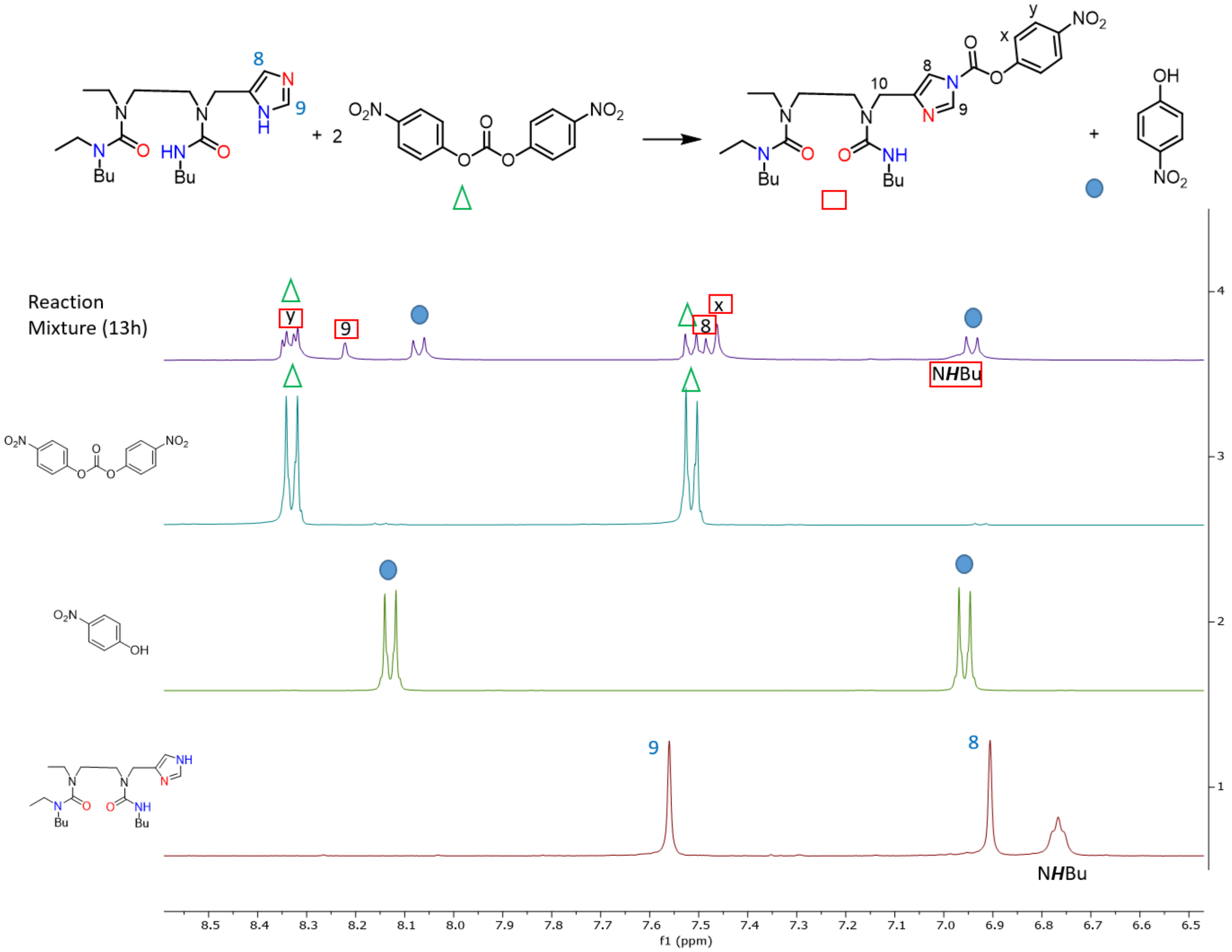

Supplementary Figure 113: Overlay of ^{1}H NMR spectra of compound **2** (41.22 mM in CD_2Cl_2) + bis(4-nitrophenyl)carbonate **4** (2 equivalents) after 13 hours at ambient temperature, bis(4-nitrophenyl)carbonate, 4-nitrophenol, **2**, all in dichloromethane.

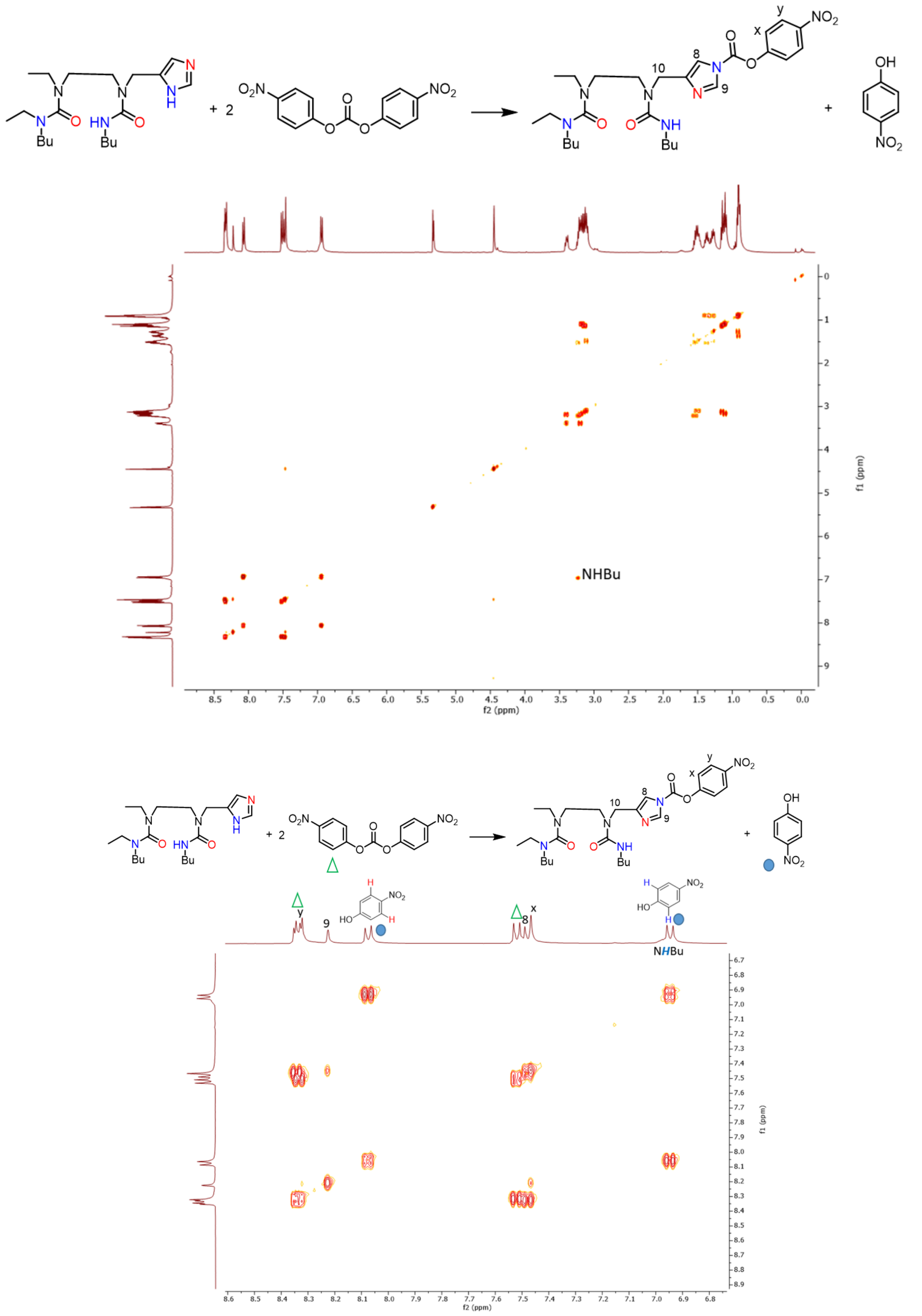

Supplementary Figure 114. COSY ^{1}H NMR spectra of a mixture imidazole (41.22 mM in CD_2Cl_2)+ bis(4-nitrophenyl)carbonate **4** (2 equivalents) after 13 hours at ambient temperature.

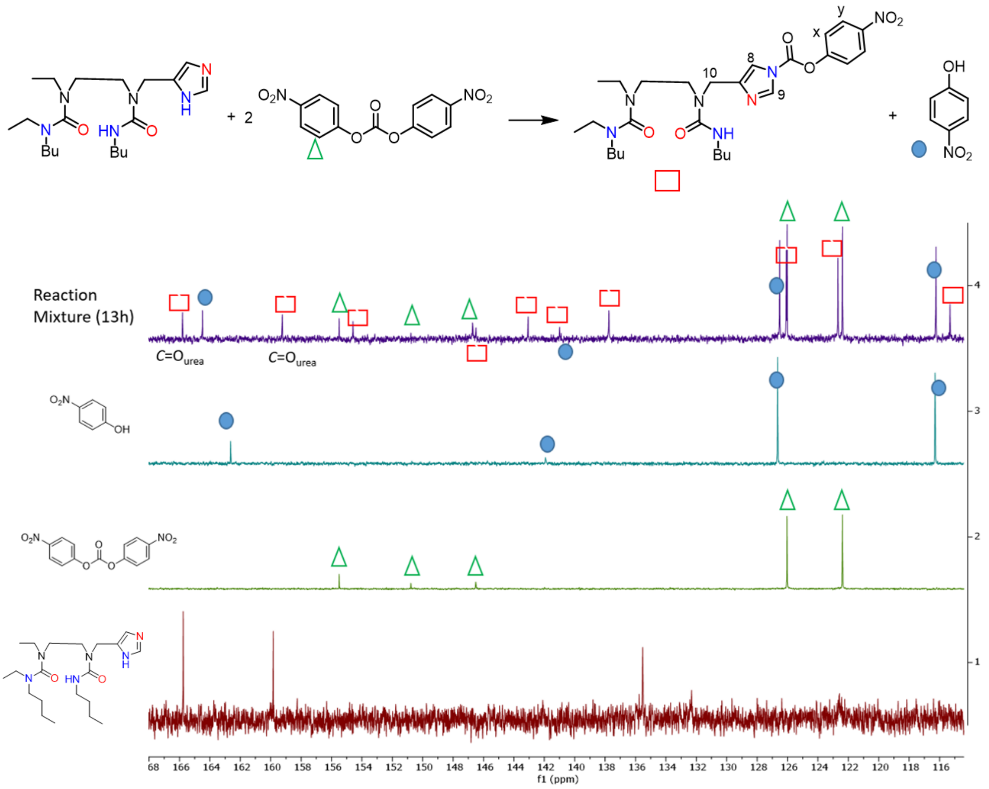

Supplementary Figure 115. Overlay of ^{13}C NMR spectra of **2** (1 equivalent) + bis(4-nitrophenyl)carbonate **4** (2 equivalents) in dichloromethane after 13 hours at ambient temperature, 4-nitrophenol, bis(4-nitrophenyl)carbonate, **2**.

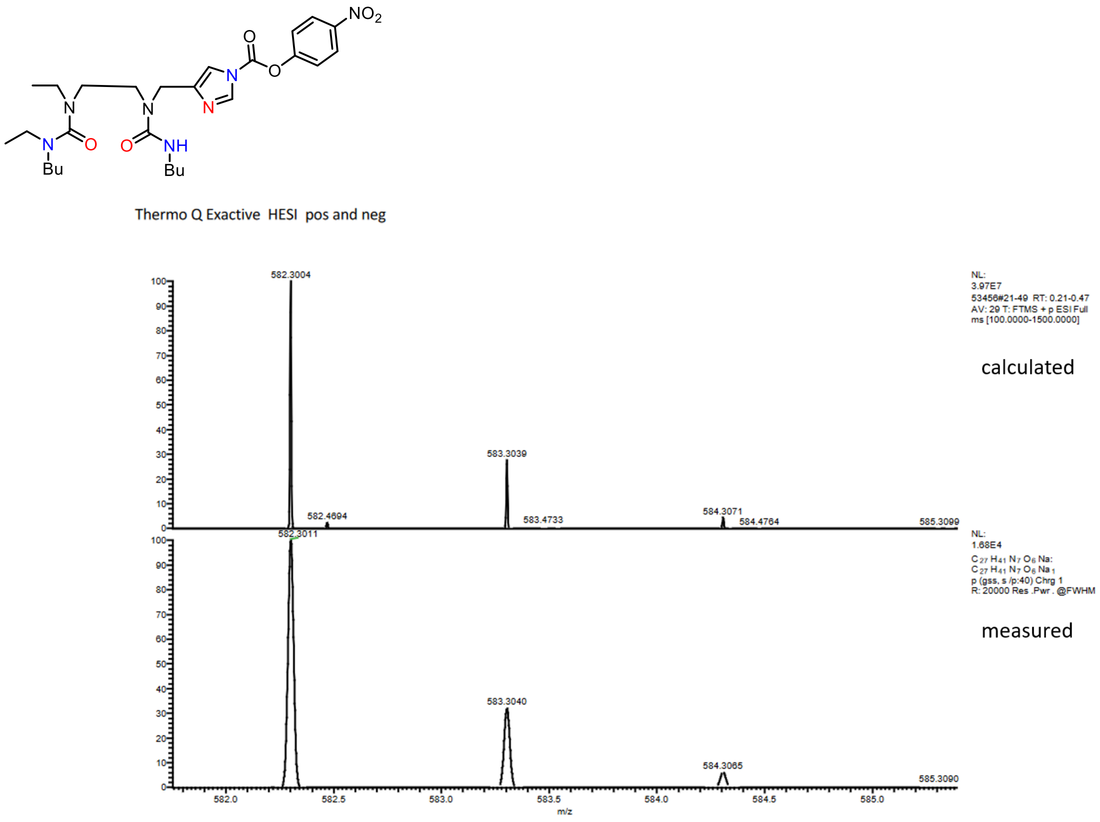

Supplementary Figure 116. HRMS spectra of adduct formed by reaction of **2** (1 equivalent) with bis(4-nitrophenyl)carbonate **4** (1 equivalent) in dichloromethane after 13 hours at ambient temperature (calculated spectrum and measured spectrum illustrating the match of data). **HR – MS** (ESI, positive ion mode) – *m/z* for $[C_{27}H_{41}O_6N_7Na]^+$ 582.3011, observed 582.3004.

7.4. Reaction of bis(4-nitrophenyl)carbonate and compound **1** in the presence of tetrabutylammonium chloride

7.4.1. UV-visible spectroscopy monitoring of bis(4-nitrophenyl)carbonate reacting with **1** in the presence of tetrabutylammonium chloride

- A stock solution of tetrabutylammonium chloride (54.95 mM in dry dichloromethane) was prepared by diluting 11 mg (0.0395 mmol) of tetrabutylammonium chloride in 0.720 mL of dry dichloromethane.

The titrated solution of bis(4-nitrophenyl)carbonate **4** (0.37 mM) and compound **1** (10 equivalents) in dry dichloromethane (0.3 mL) was prepared by diluting 0.0033 mL of bis(4-nitrophenyl)carbonate stock solution (32.9 mM) with 0.265 mL of dry dichloromethane in the cuvette (0.00011 mmol of bis(4-nitrophenyl)carbonate), then adding 0.031 mL of 34.8 mM stock solution of compound **1** (0.0011 mmol, 10 equivalents).

Titration with tetrabutylammonium chloride proceeds by successive addition of 0.006 mL of the tetrabutylammonium chloride stock solution (54.95 mM) each time (0.006 mL is 1 equivalent, 0.00011 mmol) to the titrated solution in the cuvette, agitation of the resulting solution, and recording of the UV light absorbance spectrum 5 minutes after each addition. UV light absorbance intensities shown in UV titration spectra are not corrected for changes in concentration - the substrate concentrations decreased from 0.37 mM at the start to 0.32 mM at the completion of the titration. No insoluble material was formed during the titration in the cuvette.

(20)

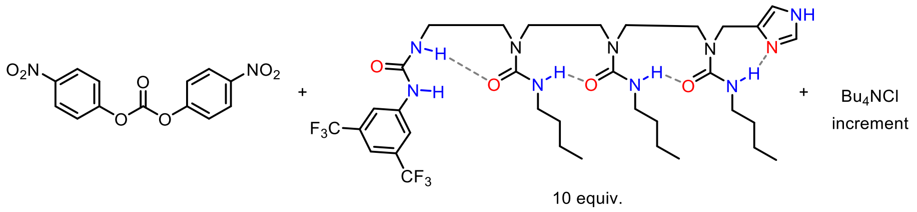

a)

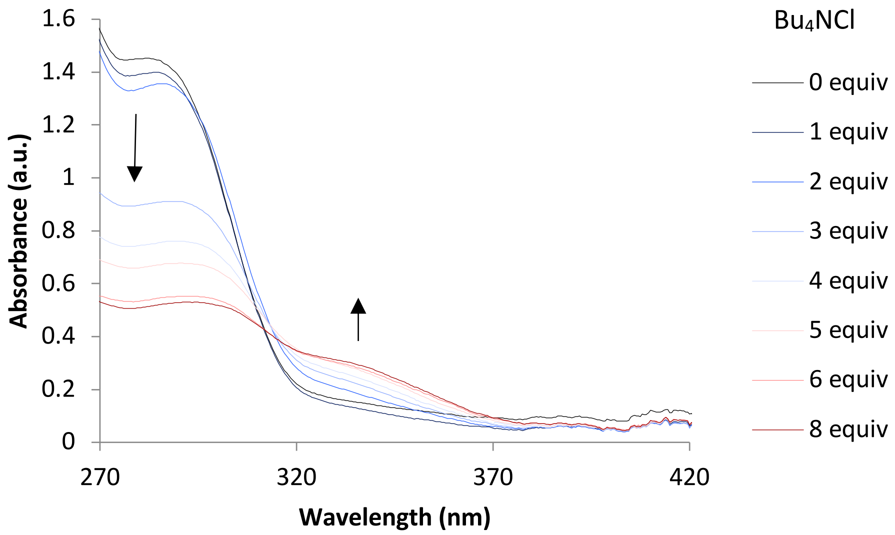

b)

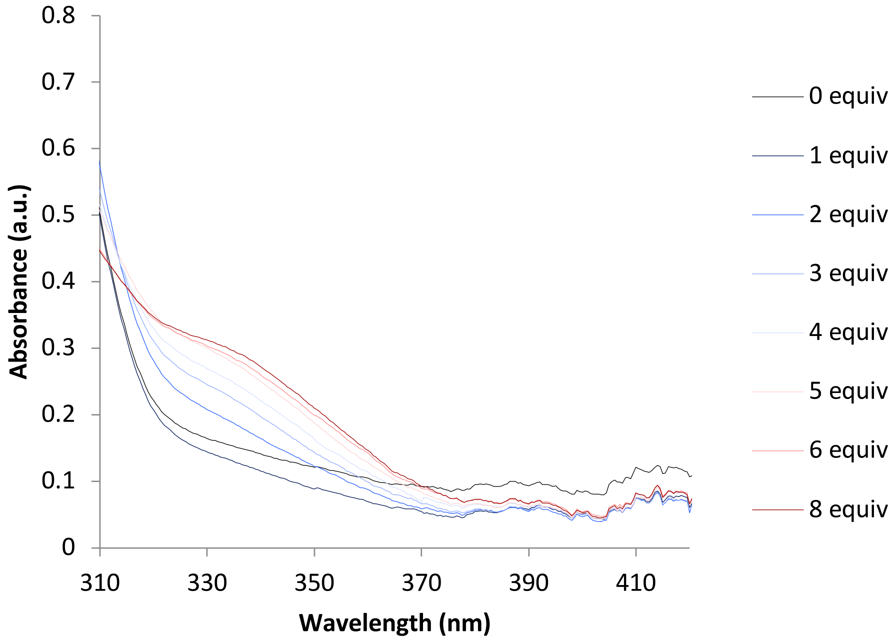

Supplementary Figure 117. Stacked UV light absorbance spectra recorded during the titration of a solution of bis(4-nitrophenyl)carbonate **4** (0.37 mM in dry dichloromethane) and **1** (10 equivalents) with increments of tetrabutylammonium chloride (a) full spectrum and b) expansion 310-410 nm)**.** Source data are provided as a Source Data file.

The stacked UV light absorbance spectra recorded during the titration show the presence of two isobestic points at 313 nm and 373 nm. Progressive increase in UV light absorbance at wavelengths between 313-360 nm and progressive decrease in UV light absorbance at wavelengths between 270-313 nm occurs upon addition of

tetrabutylammonium chloride. The UV light absorbance is high (above 1.2 a.u.) at wavelengths below 270 nm due to the absorbance of the 10 equivalents of foldamer in solution. Changes in UV light absorbance occur between 0 and 6 equivalents of tetrabutylammonium chloride added, the addition of more equivalents of tetrabutylammonium chloride leads to no change in the UV light absorbance spectrum. As control experiment, the UV light absorbance spectrum of tetrabutylammonium chloride (1 mM in dry dichloromethane) alone shows no absorbance at wavelengths between 250-520 nm (Supplementary Figure 90). In another control experiment, the UV light absorbance spectra of bis(4-nitrophenyl)carbonate (0.37 mM mM in dry dichloromethane) were recorded without and with tetrabutylammonium chloride (0, 3, 9 and 12 equivalents) (Supplementary Figure 122), the direct interaction of tetrabutylamminium chloride with bis(4-nitrophenyl)carbonate leads to small increase in UV light absorbance in the region of 320-360 nm (0.06 a.u. increase at 334 nm) that does account for the changes in UV light absorbance signals observed during this titration at those wavelengths (0.18 a.u. at 338 nm). Thus the changes in UV light absorbance observed during the titration cannot be attributed to the direct interaction of tetrabutylammonium chloride with bis(4-nitrophenyl)carbonate. The UV light absorbance spectrum of foldamer **1** (1.16 mM) in dry dichloromethane recorded in the presence of tetrabutylammonium chloride (1 equivalent) (Supplementary Figure 86) shows that the increased UV light absorbance cannot be attributed to the interaction of compound **1** (1.16 mM) with tetrabutylammonium chloride only. Finally, between 300-370 nm (where the absorbance of compound **1** does not overlap the adduct UV light absorbance) the titration spectra have similar absorbance values to those recorded during the titration of bis(4-nitrophenyl)carbonate with imidazole at similar concentration (Supplementary Figure 95). Overall, the titration data are consistent with the progressive nucleophilic addition to the bis(4-nitrophenyl)carbonate by {**1**:Bu_4NCl} to form the nucleophilic addition adduct in anhydrous solution. The titration spectra are in sharp contrast with those recorded in the absence of tetrabutylammonium chloride. The restored nucleophilicity of compound **1** in the presence of Bu_4NCl correlates with the induced switch of directionality of the hydrogen bond chain in compound **1** as shown during NMR titrations.

7.4.2. Mass spectrometric study to identify reaction products

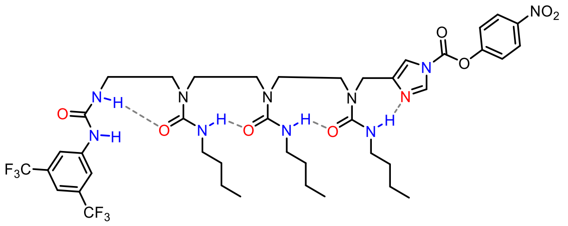

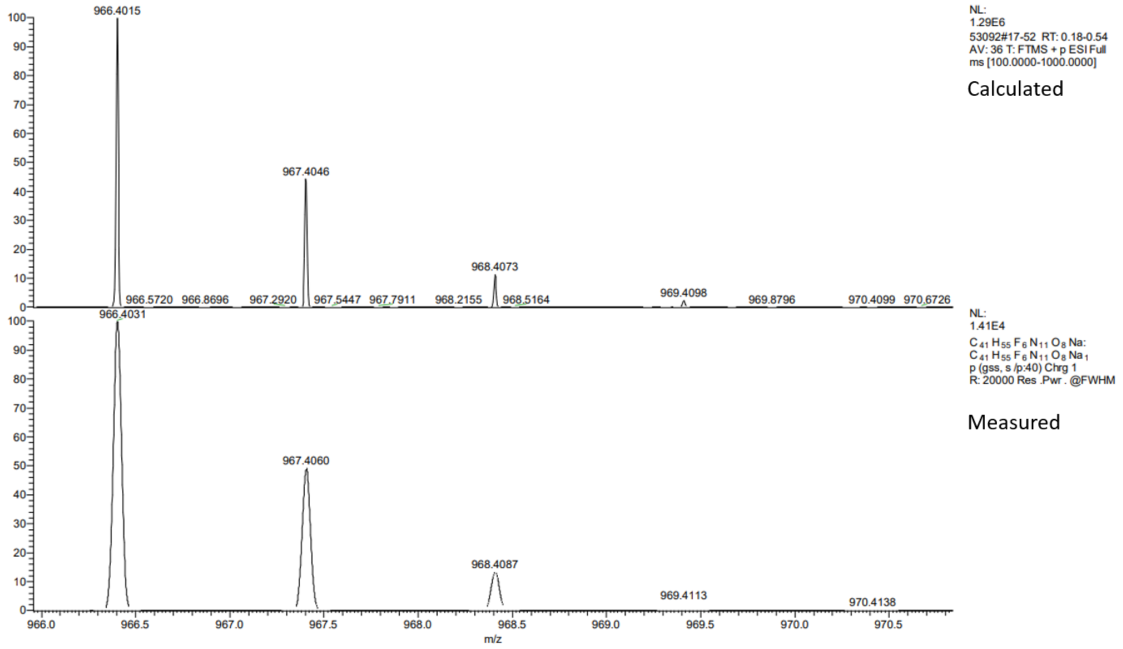

Supplementary Figure 118. HRMS spectra of adduct formed by reaction of **1** (3 equivalent) and Bu_4NCl (3 equivalents) with bis(4-nitrophenyl)carbonate **4** (1 equivalent) in dichloromethane after 13 hours at ambient temperature (calculated spectrum and measured spectrum illustrating the match of data). **HR – MS** (ESI, positive ion mode) – *m/z* for $[C_{41}H_{55}N_{11}O_8F_6Na]^+$ calculated 966.4015, observed 966.4031.

7.4.3. Control experiments

- NMR titration of compound 1 with bis(4-nitrophenyl)carbonate in CD_2Cl_2 at 25 °C

The titration establishes that bis(4-nitrophenyl)carbonate does not complex compound **1**, is not activated by **1** for subsequent nucleophilic addition.

(21) 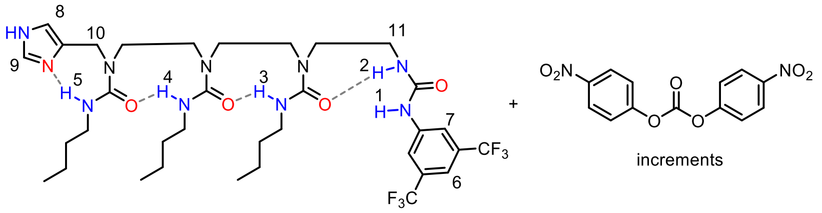

Titration of compound **1** (2.4 mg, 0.00308 mmol, 7.712 mM in 0.4 mL of CD_2Cl_2) at 25 °C with increasing amounts of bis(4-nitrophenyl)carbonate **4** (from 0 to 4 equivalents) was monitored by ^1H NMR (Supplementary Figure 119). The values of chemical shifts of the ureido NH of compound **1** upon addition of ligand are collected in Supplementary Table 8 and plotted in a graph as {chemical shift of NH = f(ligand added)} (Supplementary Figure 120). The chemical shift variation for all ureido NH signals was minimal during the titration. An association constant for the binding event of bis(4-nitrophenyl)carbonate **4** was estimated by non-linear curve fitting analysis of the titration curves with theoretical binding isotherms for 1:1, 1:2, 2:1 binding modes using supramolecular.org

(Supplementary Figure 121). The best fit using the chemical shifts of all NH signals is obtained for a 1:1 binding mode : $K = 16 \pm 1\ M^{-1}$. Bis(4-nitrophenyl)carbonate **4** does not bind compound **1**.

A

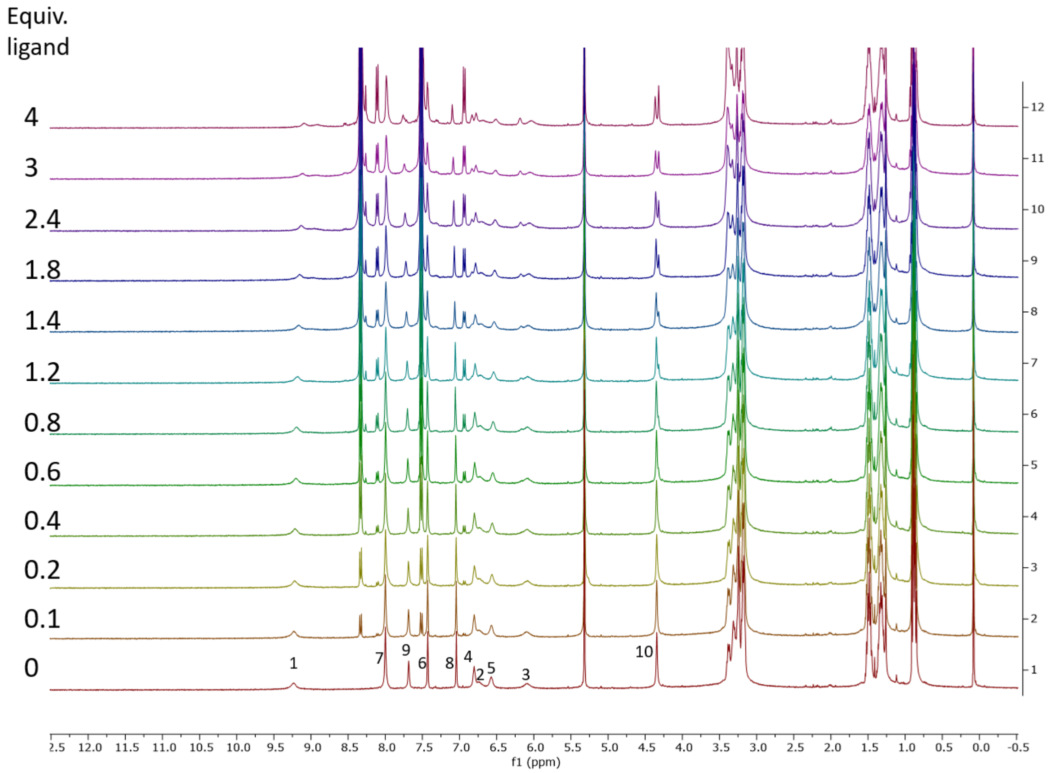

B

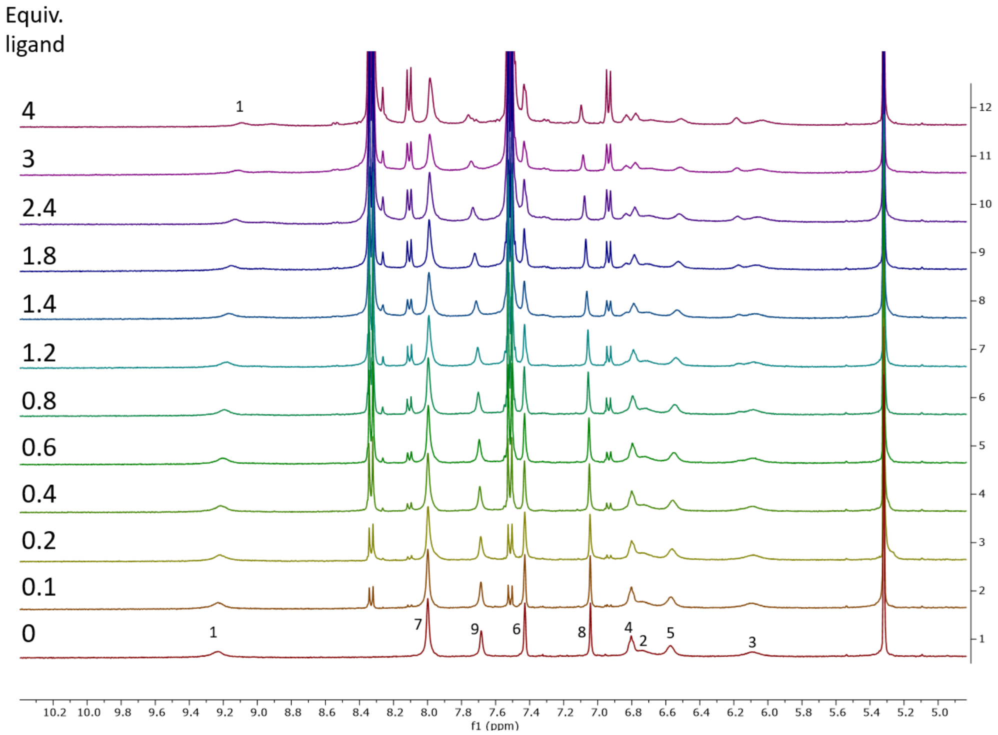

Supplementary Figure 119. Overlay of ^1H NMR spectra of **1** (7.712 mM in CD_2Cl_2, 400 MHz) in the presence of increasing amounts of bis(4-nitrophenyl)carbonate **4** from 0 to 4 equivalents (A: full spectrum, and B: zoom on the NH region).

Supplementary Table 8. chemical shifts of ^{1}H NMR NH signals recorded during the titration of **1** (7.712 mM in CD_2Cl_2) with increments of bis(4-nitrophenyl)carbonate **4** at 25 °C (400 MHz).

Concentration of host (mol/L)	Equivalents of ligand	Concentration ligand (mol/L)	NH ^{1} (ppm)	NH ^{4} (ppm)	NH ^{2} (ppm)	NH ^{5} (ppm)	NH ^{3} (ppm)
0.007712	0	0	9.23	6.8	6.74	6.57	6.09
0.007712	0.1	0.0007712	9.23	6.8	6.74	6.57	6.09
0.007712	0.2	0.0015424	9.22	6.8	6.74	6.56	6.09
0.007712	0.4	0.0030848	9.21	6.8	6.73	6.56	6.09
0.007712	0.6	0.0046272	9.2	6.79	6.73	6.55	6.08
0.007712	0.8	0.0061696	9.19	6.79	6.73	6.55	6.08
0.007712	1.2	0.0092544	9.18	6.79	6.72	6.54	6.08
0.007712	1.4	0.0107968	9.17	6.79	6.71	6.54	6.08
0.007712	1.8	0.0138816	9.16	6.79	6.71	6.53	6.07
0.007712	2.4	0.0185088	9.13	6.78	6.7	6.52	6.06
0.007712	3	0.023136	9.11	6.78	6.7	6.52	6.05
0.007712	4	0.030848	9.09	6.77	6.69	6.51	6.03
	CIS (ppm)		0.14	0.03	0.05	0.06	0.06

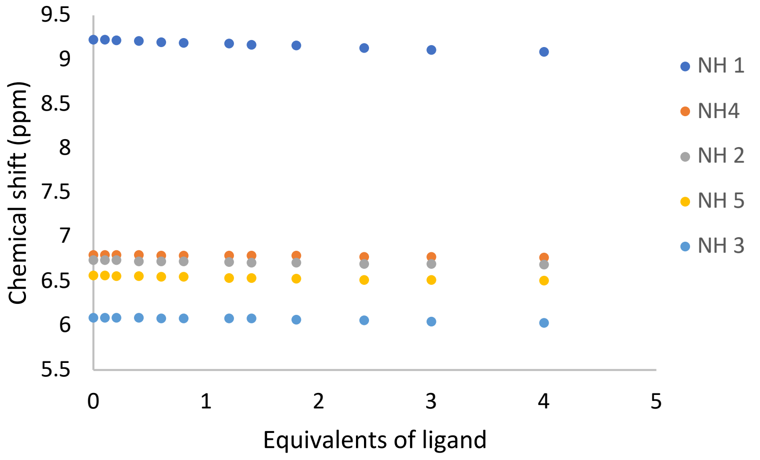

Supplementary Figure 120. Plots of variation of chemical shifts of NH signals of **1** (7.712 mM in CD_2Cl_2) upon addition of increments of bis(4-nitrophenyl)carbonate **4**, recorded by ^{1}H NMR at 25 °C (400 MHz).

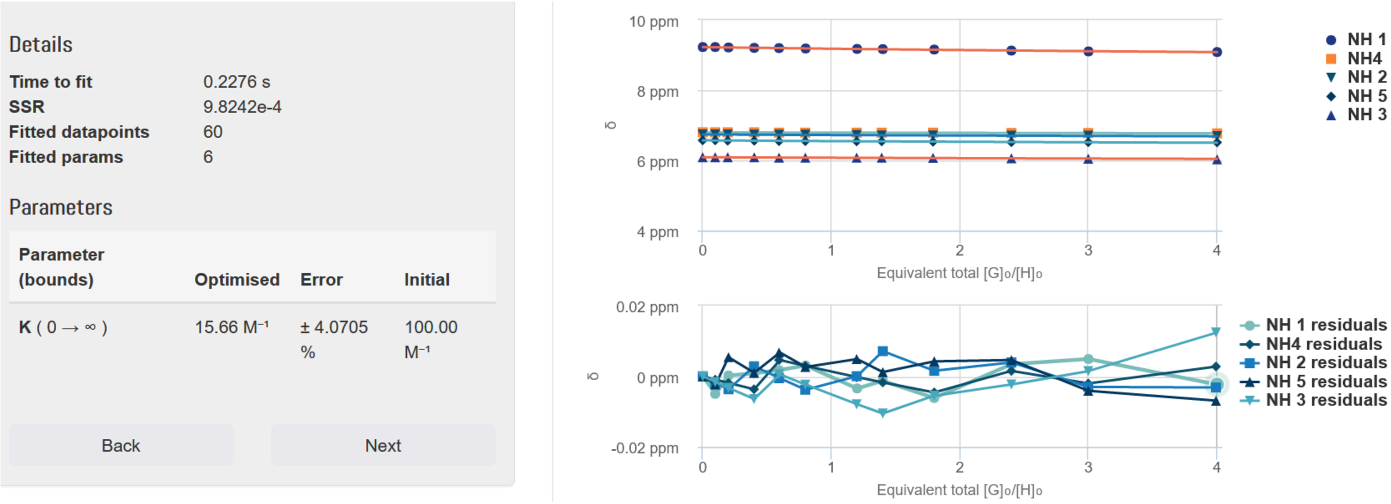

Supplementary Figure 121. Non-linear curve fitting analysis of variation of ^{1}H NMR chemical shifts of NH signals recorded during the titration of **1** (7.712 mM in CD_2Cl_2, 400 MHz) with bis(4-nitrophenyl)carbonate **4** from 0 to 4 equivalents using a theoretical binding isotherm for 1:1 binding. Source data are provided as a Source Data file. http://app.supramolecular.org/bindfit/view/57c79020-0083-4d08-b69a-c7d01c15e16e

Titration of bis(4-nitrophenyl)carbonate (0.37 mM in dry dichloromethane) with tetrabutylammonium chloride

The titration establishes no reaction occurs between bis(4-nitrophenyl)carbonate (0.37 mM in dry dichloromethane) and tetrabutylammonium chloride (12 equivalents). The titrated solution of bis(4-nitrophenyl)carbonate **4** (0.37 mM, 0.3 mL) was prepared by diluting 0.0033 mL of bis(4-nitrophenyl)carbonate stock solution (32.9 mM) with 0.29 mL of dry dichloromethane in the cuvette (0.00011 mmol of bis(4-nitrophenyl)carbonate in the cuvette).

Titration with tetrabutylammonium chloride proceeds by successive addition of 0.002 mL (3 equivalents, 0.00033 mmol) of the tetrabutylammonium chloride 165.5 mM stock solution each time to the cuvette, the UV light absorbance spectrum of the resulting solution was recorded 5 minutes after each addition. UV light absorbance intensities shown in UV titration spectra are not corrected for changes in concentration - the substrate concentrations decreased from 0.375 mM at the start to 0.366 mM at the completion of the titration. No insoluble material was formed during the titration in the cuvette.

(23) 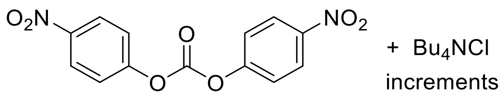

a)

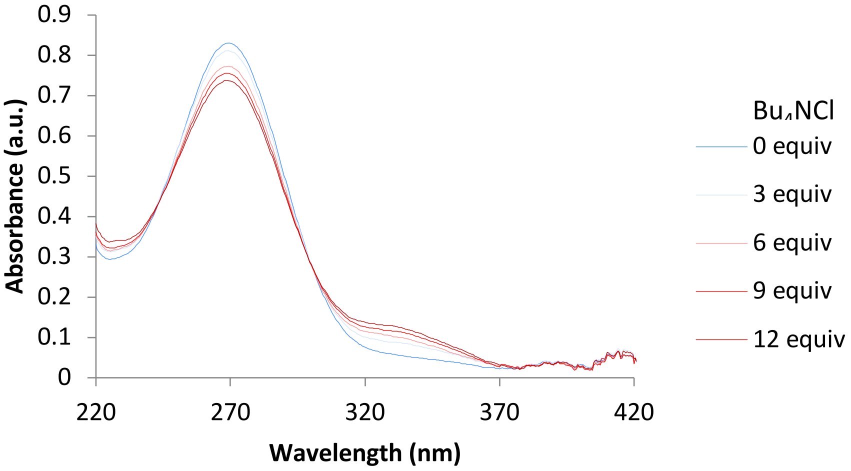

b)

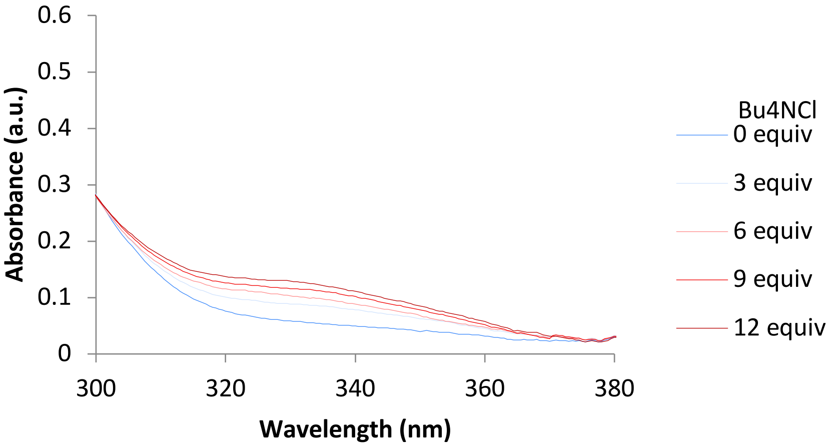

Supplementary Figure 122. Stacked UV light absorbance spectra recorded during the titration of a solution of bis(4-nitrophenyl)carbonate (0.37 mM in dry dichloromethane) with increments of tetrabutylammonium chloride (a) full spectra, b) expansion 300-380 nm). Source data are provided as a Source Data file.

7.4.4. Time course studies of the addition to bis(4-nitrophenyl)carbonate

Reaction of bis(4-nitrophenyl)carbonate (0.37 mM) in the presence of imidazole (3 equivalents)

A solution of bis(4-nitrophenyl)carbonate **4** (0.37 mM, 0.3 mL) was prepared by diluting 0.0033 mL of bis(4-nitrophenyl)carbonate stock solution (32.9 mM) with 0.3 mL of dry dichloromethane in the cuvette (0.00011 mmol of bis(4-nitrophenyl)carbonate in the cuvette). A volume of 0.0015 mL of an imidazole stock solution (220.6 mM) (3 equivalents of imidazole, 0.00033 mmol) was added to the bis(4-nitrophenyl)carbonate solution in the cuvette, followed with agitation of the resulting solution. The time course was started immediately thereafter. UV light absorbance of the solution was measured automatically by the spectrometer at a wavelength of 327 nm, a wavelength that corresponds to a local maximum of UV light absorbance observed during the titration of bis(4-nitrophenyl)carbonate (0.37 mM) with imidazole. The measurements were made every 30 minutes for 600 minutes.

The response of the spectrophotometer was set to 'medium', the UV band width of 1 nm was set as parameter. No insoluble material was formed in the cuvette during the time course.

Reaction of bis(4-nitrophenyl)carbonate 4 (0.37 mM) in the presence of 1 (3 equivalents)

A solution of bis(4-nitrophenyl)carbonate **4** (0.37 mM, 0.3 mL) prepared by diluting 0.0033 mL of bis(4-nitrophenyl)carbonate stock solution (32.9 mM) with 0.3 mL of dry dichloromethane (0.00011 mmol of bis(4-nitrophenyl)carbonate in the cuvette). A volume of 0.0093 mL (0.00032 mmol) of a **1** stock solution (34.8 mM) was added to the bis(4-nitrophenyl)carbonate solution in the cuvette, followed with agitation of the resulting solution. The time course was started immediately thereafter. UV light absorbance of the solution was measured automatically by the spectrometer at a wavelength of 327 nm, a wavelength that corresponds to a local maximum of UV light absorbance observed during the titration of bis(4-nitrophenyl)carbonate (0.37 mM) with imidazole. The measurement was made every 30 minutes for 600 minutes. The response was set to 'medium', the UV band width of 1 nm was set as parameter. No insoluble material was formed in the cuvette during the time course.

Reaction of bis(4-nitrophenyl)carbonate (0.37 mM) in the presence of 1 (3 equivalents) and tetrabutylammonium anions (either chloride, bromide, iodide, nitrate, tetraphenylborate) (3 equivalents)

A solution of bis(4-nitrophenyl)carbonate **4** (0.37 mM, 0.3 mL) prepared by diluting 0.0033 mL of bis(4-nitrophenyl)carbonate stock solution (32.9 mM) with 0.3 mL of dry dichloromethane (0.00011 mmol of bis(4-nitrophenyl)carbonate in the cuvette). A volume of 0.0093 mL (0.00032 mmol) of a **1** stock solution (34.8 mM) was added to the bis(4-nitrophenyl)carbonate solution in the cuvette, followed with agitation of the resulting solution. A volume of 0.006 mL (0.00033 mmol) of a tetrabutylammonium anion stock solution (54.9 mM) was added to the solution in the cuvette, followed with agitation of the resulting solution. The time course was started immediately thereafter. UV light absorbance of the solution was measured automatically by the spectrometer at a wavelength of 327 nm, a wavelength that corresponds to a local maximum of UV light absorbance observed during the titration of bis(4-nitrophenyl)carbonate (0.37 mM) with imidazole. The measurement was made every 30 minutes for 600 minutes. The response was set to 'medium', the UV band width of 1 nm was set as parameter. No insoluble material was formed in the cuvette during the time course.

Reaction of bis(4-nitrophenyl)carbonate 4 (0.37 mM) in the presence of 2 (3 equivalents)

A solution of bis(4-nitrophenyl)carbonate **4** (0.37 mM, 0.3 mL) prepared by diluting 0.0033 mL of bis(4-nitrophenyl)carbonate stock solution (32.9 mM) with 0.3 mL of dry dichloromethane (0.00011 mmol of bis(4-nitrophenyl)carbonate in the cuvette). A volume of 0.0024 mL (0.00032 mmol) of a **2** stock solution (144 mM) was added to the bis(4-nitrophenyl)carbonate solution in the cuvette, followed with agitation of the resulting solution. The time course was started immediately thereafter. UV light absorbance of the solution was measured automatically by the spectrometer at a wavelength of 327 nm, a wavelength that corresponds to a local maximum of UV light absorbance observed during the titration of bis(4-nitrophenyl)carbonate (0.37 mM) with imidazole. The measurement was made every 30 minutes for 600 minutes. The response was set to 'medium', the UV band width of 1 nm was set as parameter. No insoluble material was formed in the cuvette during the time course.

Reaction of bis(4-nitrophenyl)carbonate 4 (0.37 mM) in the presence of Bu_4NX (X = Cl^-. Br^-, I^-, NO_3^-, BPh_4^-) (3 equivalents)

A solution of bis(4-nitrophenyl)carbonate **4** (0.37 mM, 0.3 mL) prepared by diluting 0.0033 mL of bis(4-nitrophenyl)carbonate stock solution (32.9 mM) with 0.3 mL of dry dichloromethane (0.00011 mmol of bis(4-nitrophenyl)carbonate in the cuvette). A volume of 0.006 mL (0.00033 mmol) of a tetrabutylammonium anion stock solution (54.9 mM) was added to the solution in the cuvette, followed with agitation of the resulting solution. The time course was started immediately thereafter. UV light absorbance of the solution was measured automatically by the spectrometer at a wavelength of 327 nm, a wavelength that corresponds to a local maximum of UV light absorbance observed during the titration of bis(4-nitrophenyl)carbonate (0.37 mM) with imidazole. The

measurement was made every 30 minutes for 600 minutes. The response was set to 'medium', the UV band width of 1 nm was set as parameter. No insoluble material was formed in the cuvette during the time course.

Reaction of bis(4-nitrophenyl)carbonate 4 (0.37 mM) in the presence of 3 (3 equivalents) and tetrabutylammonium chloride (3 equivalents)

A solution of bis(4-nitrophenyl)carbonate **4** (0.37 mM, 0.3 mL) prepared by diluting 0.0033 mL of bis(4-nitrophenyl)carbonate stock solution (32.9 mM) with 0.3 mL of dry dichloromethane (0.00011 mmol of bis(4-nitrophenyl)carbonate in the cuvette). A stock solution of **3** (11.43 mM) was prepared by dissolving 2.9 mg (0.002857 mmol) of **3** in 0.25 mL of anhydrous dichloromethane. A volume of 0.039 mL (0.00033 mmol) of that stock solution was added to the bis(4-nitrophenyl)carbonate solution in the cuvette, followed with agitation of the resulting solution. A volume of 0.006 mL (0.00033 mmol) of a tetrabutylammonium chloride stock solution (54.9 mM) was added to the solution in the cuvette, followed with agitation of the resulting solution. The time course was started immediately thereafter. UV light absorbance of the solution was measured automatically by the spectrometer at a wavelength of 327 nm, a wavelength that corresponds to a local maximum of UV light absorbance observed during the titration of bis(4-nitrophenyl)carbonate (0.37 mM) with imidazole. The measurement was made every 30 minutes for 600 minutes. The response was set to 'medium', the UV band width of 1 nm was set as parameter. No insoluble material was formed in the cuvette during the time course.

Reaction of bis(4-nitrophenyl)carbonate (0.37 mM) in the presence of 1 (3 equivalents), and addition of tetrabutylammonium chloride (3 equivalents) 193 minutes after the start of the time course study

A solution of bis(4-nitrophenyl)carbonate **4** (0.37 mM, 0.4 mL) prepared by diluting 0.0045 mL of bis(4-nitrophenyl)carbonate stock solution (32.9 mM) with 0.38 mL of dry dichloromethane (0.000148 mmol of bis(4-nitrophenyl)carbonate in the cuvette). A stock solution of **1** (21.2 mM) was prepared by dissolving 16.5.9 mg (0.00212 mmol) of **1** in 1 mL of anhydrous dichloromethane. A volume of 0.0207 mL (0.00044 mmol) of that stock solution was added to the bis(4-nitrophenyl)carbonate solution in the cuvette, followed with agitation of the resulting solution. The time course was started immediately thereafter. At 193 minutes after the start of the time course, a volume of 0.0123 mL (0.00044 mmol) of a tetrabutylammonium chloride stock solution (35.9 mM) was added to the solution in the cuvette, followed with agitation of the resulting solution. UV light absorbance of the solution was measured automatically by the spectrometer at a wavelength of 327 nm, a wavelength that corresponds to a local maximum of UV light absorbance observed during the titration of bis(4-nitrophenyl)carbonate (0.37 mM) with imidazole. The measurement was made every 30 minutes for 800 minutes. The response was set to 'medium', the UV band width of 1 nm was set as parameters. No insoluble material was formed in the cuvette during the time course.

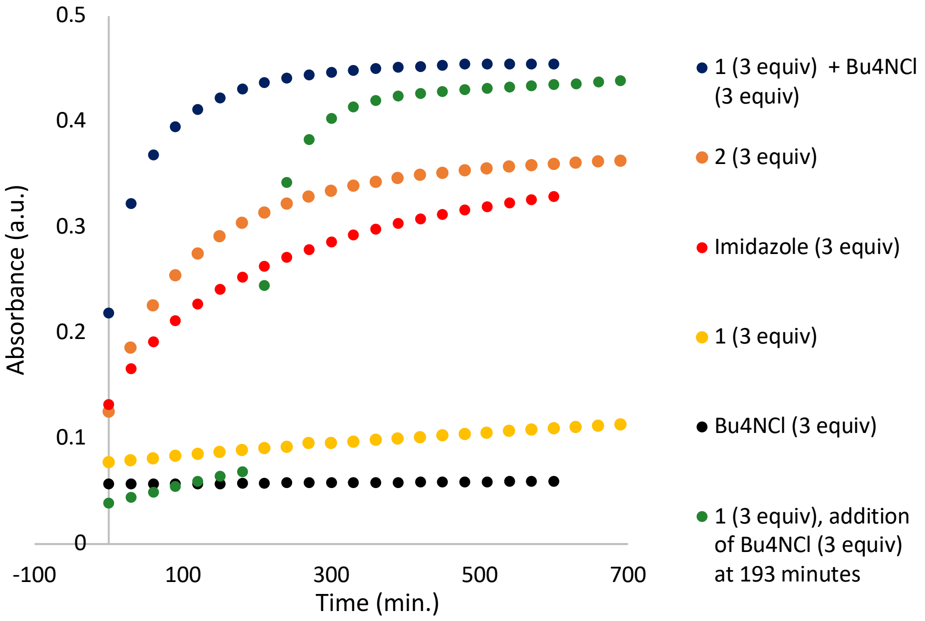

Supplementary Figure 123. UV light absorbance values recorded at 327 nm for the time course studies of bis(4-nitrophenyl)carbonate **4** (0.37 mM in dry dichloromethane) in the presence either imidazole (3 equivalents), **1** (3 equivalents), **1** (3 equivalents) with Bu_4NCl (3 equivalents), **2** (3 equivalents), **1** (3 equivalents) with addition of Bu_4NCl (3 equivalents) at 193 minutes from the start of the reaction. Source data are provided as a Source Data file.

In the presence of the foldamer **1** only (3 equivalents), the UV light absorbance stays relatively stable at 0.1 a.u. The UV light absorbance values recorded in the presence of imidazole (3 equivalents) under otherwise similar conditions evolve from 0.15 a.u. to 0.31 a.u. over 600 minutes, the change in UV absorbance value is attributed to the advance of nucleophilic addition of imidazole to bis(4-nitrophenyl)carbonate. The difference of absorbance evolution over time is interpreted as a decreased reactivity of imidazole **1** for the nucleophilic addition to bis(4-nitrophenyl)carbonate **4**.

The UV light absorbance values recorded in the presence of **2** (3 equivalents) in otherwise similar conditions evolve from 0.15 a.u. to 0.36 a.u. over 600 minutes, those values are slightly higher than the one obtained with imidazole. The data indicate a similar reactivity of **2** and imidazole, and a difference in reactivity of **2** compared to foldamer **1** for the nucleophilic addition to bis(4-nitrophenyl)carbonate. The difference in reactivity correlates with the opposed directionality of the hydrogen bond chain in the foldamer.

The UV light absorbance values recorded using **1** (3 equivalents) with tetrabutylammonium chloride (3 equivalents) in otherwise similar conditions evolve from 0.15 a.u. to 0.45 a.u over 600 minutes. This contrasts with the values recorded for **1** in the absence of Bu_4NCl; the values also are higher than those recorded with imidazole, or with **1** foldamer. The increase of reactivity of **1** in the presence of Bu_4NCl towards nucleophilic addition to bis(4-nitrophenyl)carbonate correlates with the switch of the foldamer hydrogen bond directionality.

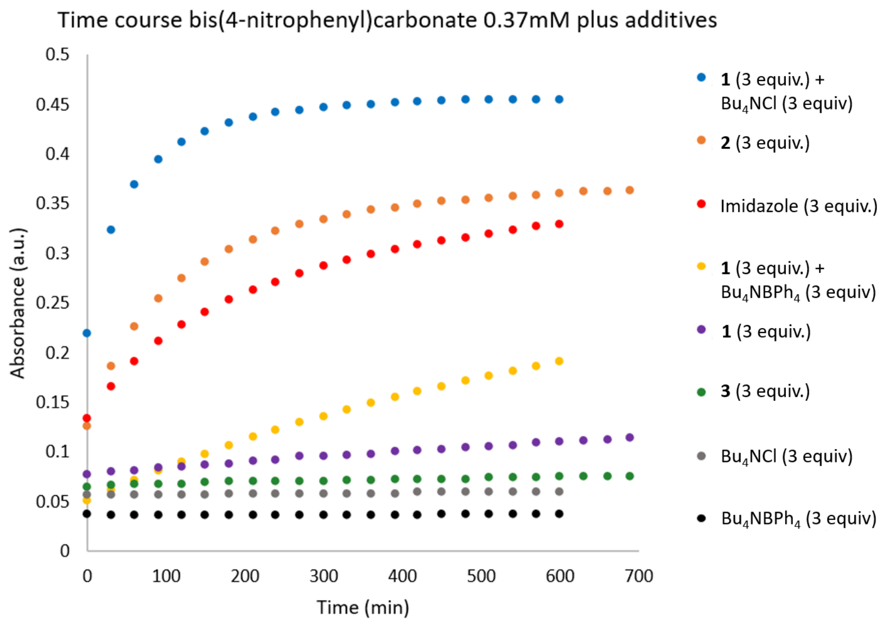

Supplementary Figure 124. Stacked UV light absorbance values recorded at 327 nm for the time course studies of bis(4-nitrophenyl)carbonate **4** (0.37 mM in dry dichloromethane) in the presence either imidazole (3 equivalents), **1** (3 equivalents), **1** (3 equivalents) with Bu_4NCl (3 equivalents), **2** (3 equivalents), **1** (3 equivalents) with Bu_4NBPh_4 (3 equivalents), Bu_4NBPh_4 (3 equivalents), Bu_4NCl (3 equivalents), **3** (3 equivalents) with Bu_4NCl (3 equivalents). Source data are provided as a Source Data file.

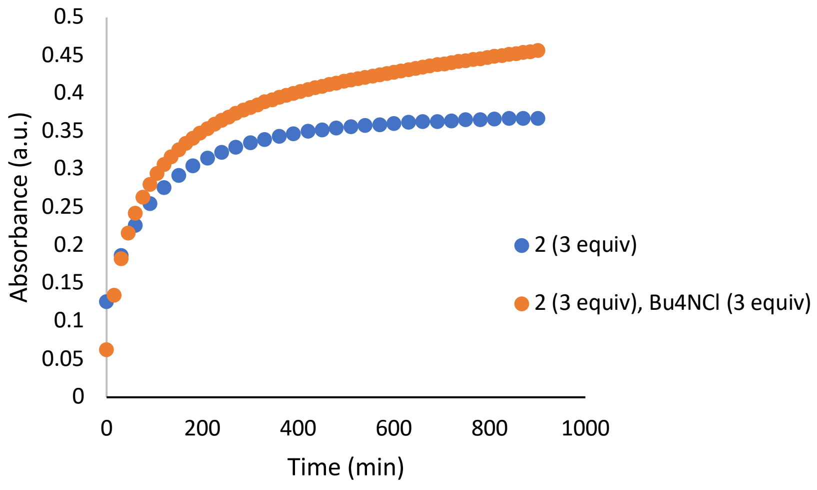

Supplementary Figure 125. Stacked UV light absorbance values recorded at 327 nm for the time course studies of bis(4-nitrophenyl)carbonate **4** (0.37 mM in dry dichloromethane) in the presence **2** (3 equivalents) with either no ligand, or Bu_4NCl (3 equivalents). Source data are provided as a Source Data file.

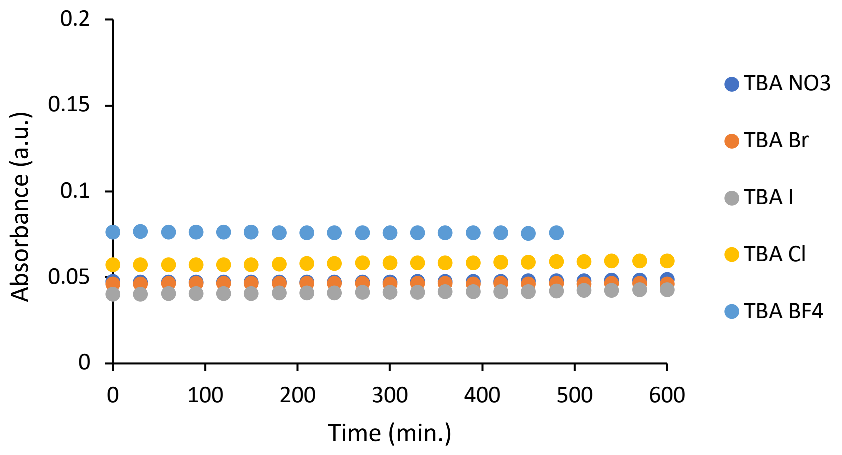

Supplementary Figure 126. Stacked UV light absorbance values (recorded at 327 nm) for the time course studies of bis(4-nitrophenyl)carbonate **4** (0.37 mM in dry dichloromethane) in the presence of various tetrabutylammonium anions (3 equivalents). No reaction occurs between the anions and bis(4-nitrophenyl)carbonate **4**. Source data are provided as a Source Data file.

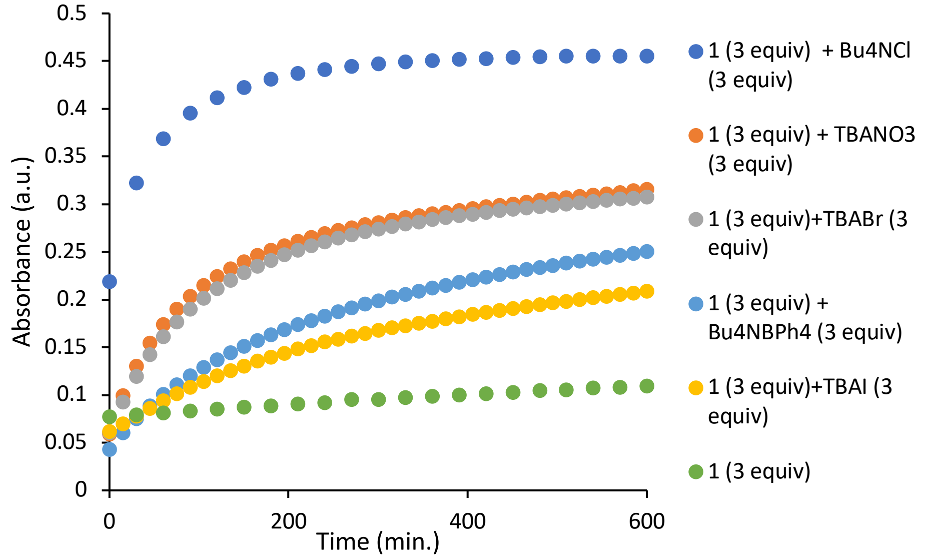

Supplementary Figure 127. Stacked UV light absorbance values (recorded at 327 nm) for the time course studies of bis(4-nitrophenyl)carbonate **4** (0.37 mM in dry dichloromethane) in the presence of compound **1** (3 equivalents) and various tetrabutylammonium anions (3 equivalents). The chloride anion provides the more activation among the anions tested. Source data are provided as a Source Data file.

Supplementary Table 9. Calculation of rate constants by non-linear curve fitting of the time course study UV-visible absorbance = f(reaction time) fitting to a first order equation making a pseudo-first order assumption, using GraphPad Prism 9.3.1 software.

**Conditions**	***k* / 10^{-6} s^{-1}**
Imidazole (3 equiv)	78.6
**1** (3 equiv)	13.0
**1**(3 equiv)+ Bu_4NCl (3 equiv)	260
**1** (3 equiv)+ Bu_4NO_3 (3 equiv)	141
**1** (3 equiv)+ Bu_4NBr (3 equiv)	126
**1** (3 equiv)+ Bu_4NI (3 equiv)	57.4
**1** (3 equiv)+ Bu_4NBF_4 (3 equiv)	50.7
**1**(3 equiv)+ Bu_4NBPh_4 (3 equiv)	23.6
**2**	126
**2**(3 equiv)+ Bu_4NCl (3 equiv)	177
**3** (3 equiv) + Bu_4NCl (3 equiv)	26.3
Bu_4NBF_4	37.7
Bu_4NCl (3 equiv)	0.0119
Bu_4NBPh_4 (3 equiv)	0.0200
Bu_4NNO_3 (3 equiv)	0.0456
Bu_4NI (3 equiv)	0.0468
Bu_4NBr (3 equiv)	0.0522

1 (3 equivalents) with Bu_4NCl (3 equivalents) reacts 20 times faster than without Bu_4NCl

1 (3 equivalents) with Bu_4NBPh_4 (3 equivalents) reacts 1.8 times faster than without Bu_4NBPh_4, attributed to the increase of polarity of the medium (non-polar dichloromethane) altering the stability of transition state.

1 reacts 6 times more slowly than imidazole in otherwise similar conditions.

2 reacts 9.75 times faster than **1** in otherwise similar conditions, and 3.3 times faster than imidazole.

2 reacts 1.8 times faster in the presence of 3 equivalents of Bu_4NCl in otherwise similar conditions.

Supplementary Table 10. Binding constants of compound **3** in dichloromethane at 25 °C with either tetrabutylammonium chloride, nitrate, bromide, iodide, and rate constants for the reaction of compound **1** with bis(4-nitrophenyl)carbonate **4** in the presence of the same ions.

	K / M^{-1} (1:1)	*k* / 10^{-6} s^{-1}
TBACl	300 ± 100	260
$TBANO_3$	1400 ± 600	141
TBABr	170 ± 20	126
TBAI	150 ± 20	57

V. Supplementary References

[1] Tilly, D. P., Zabka, M., Vitorica-Yrezabal, I., Sparkes, H. A., Pridmore, N., Clayden, J. *Chem. Sci*. **13**, 13153-13159 (2022).
[2] Bhaumik, J., Yao, Z., Borbas, K. E., Taniguchi, M., Lindsey, J.S. *J. Org. Chem*. **71,** 8807-8817 (2006).
[3] Morris, D. T. J., Wales, S. M., Tilly, D. P., Farrar, E. H. E., Grayson, M. N., Ward, J. W., Clayden, J. *Chem* **7**, 2460-2472 (2021).
[4] Frisch, M.J., Trucks, G.W., Schlegel, H.B., Scuseria, G. (2016). Gaussian16 Revision A. 03 (Wallingford, CT: Gaussian Inc.).
[5] Becke, A. D. *J. Chem. Phys.* **98,** 5648-5652 (1993).
[6] Stephens, P. J., Devlin, F. J., Chabalowski, C. F., Frisch, M. J. *J. Phys. Chem.* **98,** 11623-11627 (1994).
[7] Hehre, W. J., Ditchfield, R., Pople, J. A. *J. Chem. Phys.* **56,** 2257-2261 (1972).
[8] Sychrovsky, V., Grafenstein, J., Cremer, D. *J. Chem. Phys.* **113,** 3530-3547 (2000).
[9] Helgaker, T., Watson, M., Handy, N. C. *J. Chem. Phys.* **113,** 9402-9409 (2000).
[10] Ditchfield, R. *Mol. Phys.* **27,** 789-807 (1974).
[11] Wolinski, K., Hinton, J. F., Pulay, P. *J. Am. Chem. Soc.* **112,** 8251-8260 (1990).
[12] Hariharan, P. C., Pople, J. A. *Theor. Chim. Acta* **28,** 213-222 (1973).
[13] Chai, J.-D., Head-Gordon, M. *Phys. Chem. Chem. Phys.* **10,** 6615-6620 (2008).
